# TBC1D10C is a cytoskeletal functional linker that modulates cell spreading and phagocytosis in macrophages

**DOI:** 10.1038/s41598-021-00450-z

**Published:** 2021-10-22

**Authors:** Fabian R. Villagomez, Juan D. Diaz-Valencia, Erasmo Ovalle-García, Armando Antillón, Iván Ortega-Blake, Héctor Romero-Ramírez, Jorge F. Cerna-Cortes, Roberto Rosales-Reyes, Leopoldo Santos-Argumedo, Genaro Patiño-López

**Affiliations:** 1 Laboratorio de Investigación en Inmunología y Proteómica, Hospital Infantil de México, Federico Gómez, Ciudad de México, Mexico; 2 Laboratorio de Microbiología Molecular, Escuela Nacional de Ciencias Biológicas del Instituto Politécnico Nacional, Ciudad de México, Mexico; 3 Instituto de Ciencias Físicas, Universidad Nacional Autónoma de México, Cuernavaca, Mexico; 4 Departamento de Biomedicina Molecular, Centro de Investigación y de Estudios Avanzados del Instituto Politécnico Nacional, Ciudad De México, Mexico; 5 Laboratorio de Infectología, Microbiología e Inmunología Clínica, Unidad de Investigación en Medicina Experimental de la Universidad Nacional Autónoma de México, Ciudad de México, Mexico

**Keywords:** Monocytes and macrophages, Innate immune cells

## Abstract

Cell spreading and phagocytosis are notably regulated by small GTPases and GAP proteins. TBC1D10C is a dual inhibitory protein with GAP activity. In immune cells, TBC1D10C is one of the elements regulating lymphocyte activation. However, its specific role in macrophages remains unknown. Here, we show that TBC1D10C engages in functions dependent on the cytoskeleton and plasma membrane reorganization. Using ex vivo and in vitro assays, we found that elimination and overexpression of TBC1D10C modified the cytoskeletal architecture of macrophages by decreasing and increasing the spreading ability of these cells, respectively. In addition, TBC1D10C overexpression contributed to higher phagocytic activity against *Burkholderia cenocepacia* and to increased cell membrane tension. Furthermore, by performing in vitro and in silico analyses, we identified 27 TBC1D10C-interacting proteins, some of which were functionally classified as protein complexes involved in cytoskeletal dynamics. Interestingly, we identified one unreported TBC1D10C-intrinsically disordered region (IDR) with biological potential at the cytoskeleton level. Our results demonstrate that TBC1D10C shapes macrophage activity by inducing reorganization of the cytoskeleton-plasma membrane in cell spreading and phagocytosis. We anticipate our results will be the basis for further studies focused on TBC1D10C. For example, the specific molecular mechanism in *Burkholderia cenocepacia* phagocytosis and functional analysis of TBC1D10C-IDR are needed to further understand its role in health and disease.

## Introduction

TBC1D10C (also known as Epi64C or Carabin) is an abundant protein in spleen and peripheral blood leukocytes. It is a member of the EPI64 subfamily, a group of related proteins (TBC1D10A/EPI64A, TBC1D10B/EPI64B, and TBC1D10C) containing a TBC (Tre-2/Bud2/Cdc16) domain that confers GAP (GTPase-activating protein) activity^1,2^. GAP proteins inactivate small GTPases by accelerating the intrinsic capacity of the small GTPase to hydrolyze GTP (guanosine triphosphate). The aforementioned is a well-known mechanism whereby GAPs regulate the activity of small GTPases in cellular physiology^3^, including their participation in diverse aspects of the immune response^4^. TBC1D10C was originally identified as a dual inhibitory protein for Ras and calcineurin during T cell activation. Specifically, TBC1D10C’s inhibitory effect on Ras was attributed to the GAP domain (amino terminus; residues 89–294) and inhibition of calcineurin to the interacting calcineurin domain (carboxy terminus; residues 406–446)^2^. Later, we reported TBC1D10C as a specific GAP for Rab35 during immunological synapse formation, specifically in T cell receptor (TCR) recycling^5^. Subsequently, it was found that this GAP was also an endogenous inhibitor of B cell activation by a mechanism involving Ras inhibition^6^.

These studies described important roles for TBC1D10C in regulating the activity of lymphocytes through proteins such as calcineurin and some small GTPases^2,5,6^. Furthermore, it is known that small GTPases are among the most important modulators of cytoskeletal dynamics in both immune^4,7–11^ and nonimmune cells^1,12–17^. Likewise, these proteins coordinately and sometimes oppositely control different cellular processes. In particular, it is interesting that TBC1D10C has been functionally related to Arf6^12,13^ and Rab35^14,15^, which together, coordinately and oppositely, participate in diverse cellular functions in many cell types^18–21^. For example, these small GTPases modulate several aspects of the phagocytosis process in macrophages^20,22,23^. Nevertheless, the role of TBC1D10C in macrophage physiology has not been investigated. Therefore, we studied TBC1D10C using TBC1D10C knockout (KO) (BMDMs; bone marrow-derived macrophages) and stably transfected (RAW 264.7; GFP-TBC1D10C) macrophages as ex vivo and in vitro models, respectively. Here, we report that TBC1D10C participates in cellular pathways for cytoskeleton-plasma membrane reorganization that impact cell spreading and phagocytosis in macrophages.

## Results

### Immune cells show morphological defects in the cytoskeletal architecture upon changes in TBC1D10C expression

**Figure 1:**
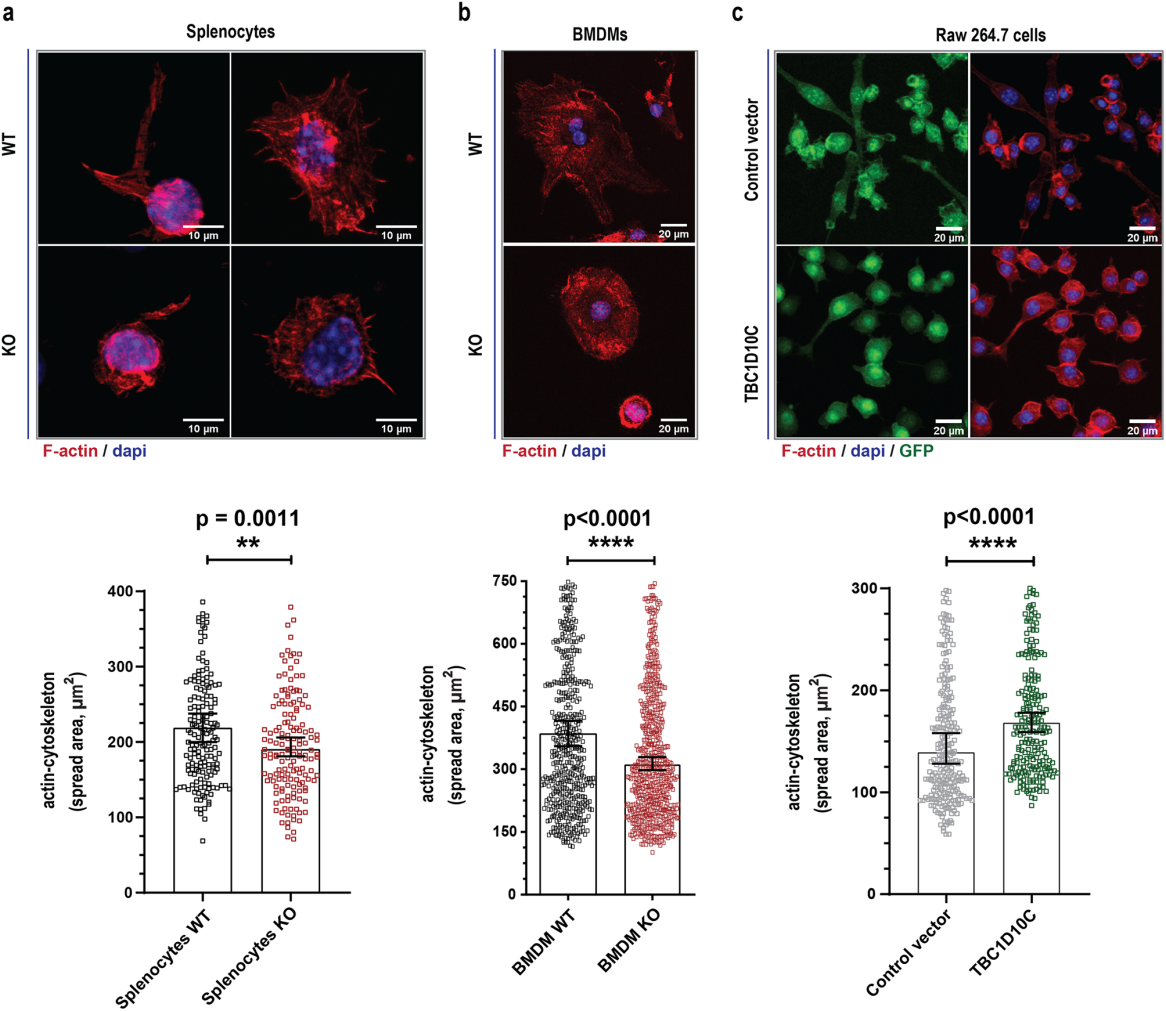
Quantification of cell spreading dependent on actin-cytoskeleton. Cells were stained with phalloidin, and the area of cell spread was calculated. (**a**) top: Confocal images of splenocytes, bottom: corresponding spread area data from confocal images (n = 4, mice; 185 WT, 158 KO cells); (**b**) top: confocal images of BMDM, bottom: corresponding spread area data from confocal images (n = 7 mice; WT BMDM, 572, KO BMDM, 623); (**c**) top: confocal images of Raw 264.7 transfected macrophages, corresponding spread area data from confocal images (254, Control vector and 221, TBC1D10C cells; 2 independent experiments). Confocal images are presented as the sum of slice projections. Data were analyzed by the Mann–Whitney test (P < 0.05), and graphs show SEM (standard error of the mean).

To investigate TBC1D10C, we generated TBC1D10C knockout (KO) mice by the Cre-Lox recombination system (Supplementary Fig. 1a–c). TBC1D10C knockout (KO) mice developed normally in the expected Mendelian ratio, and no abnormalities were detected, but they were more docile than WT (wild type) animals (data not shown). Initially, we studied immune cells from the spleen (splenocytes)^24,25^. Splenocytes were stained with phalloidin to label F-actin, and a morphometric examination was carried out. Our analysis showed that the spread area of KO splenocytes was reduced compared to that of their WT counterparts (Fig. 1a). Subsequently, we conducted the same morphometric analysis for immune cells from bone marrow, and we studied BMDMs (bone marrow-derived macrophages). We also observed that KO BMDMs showed a reduced spread area (Fig. 1b), thus corroborating that alterations related to the actin cytoskeleton of KO splenocytes (Fig. 1a) were not specific to immune cells obtained from the spleen. We focused our research on BMDMs because the role of TBC1D10C in macrophages has not been investigated. Consequently, we overexpressed TBC1D10C in RAW 264.7 cells to compare the effect of TBC1D10C elimination vs. overexpression (Supplementary Fig. 1d). In contrast to KO BMDMs, overexpression of TBC1D10C was associated with an increased spread area (Fig. 1c). Our initial data suggested that TBC1D10C influences morphological changes in macrophages through actin-cytoskeleton changes.

### TBC1D10C modifies cytoskeleton-plasma membrane dynamics

**Figure 2:**
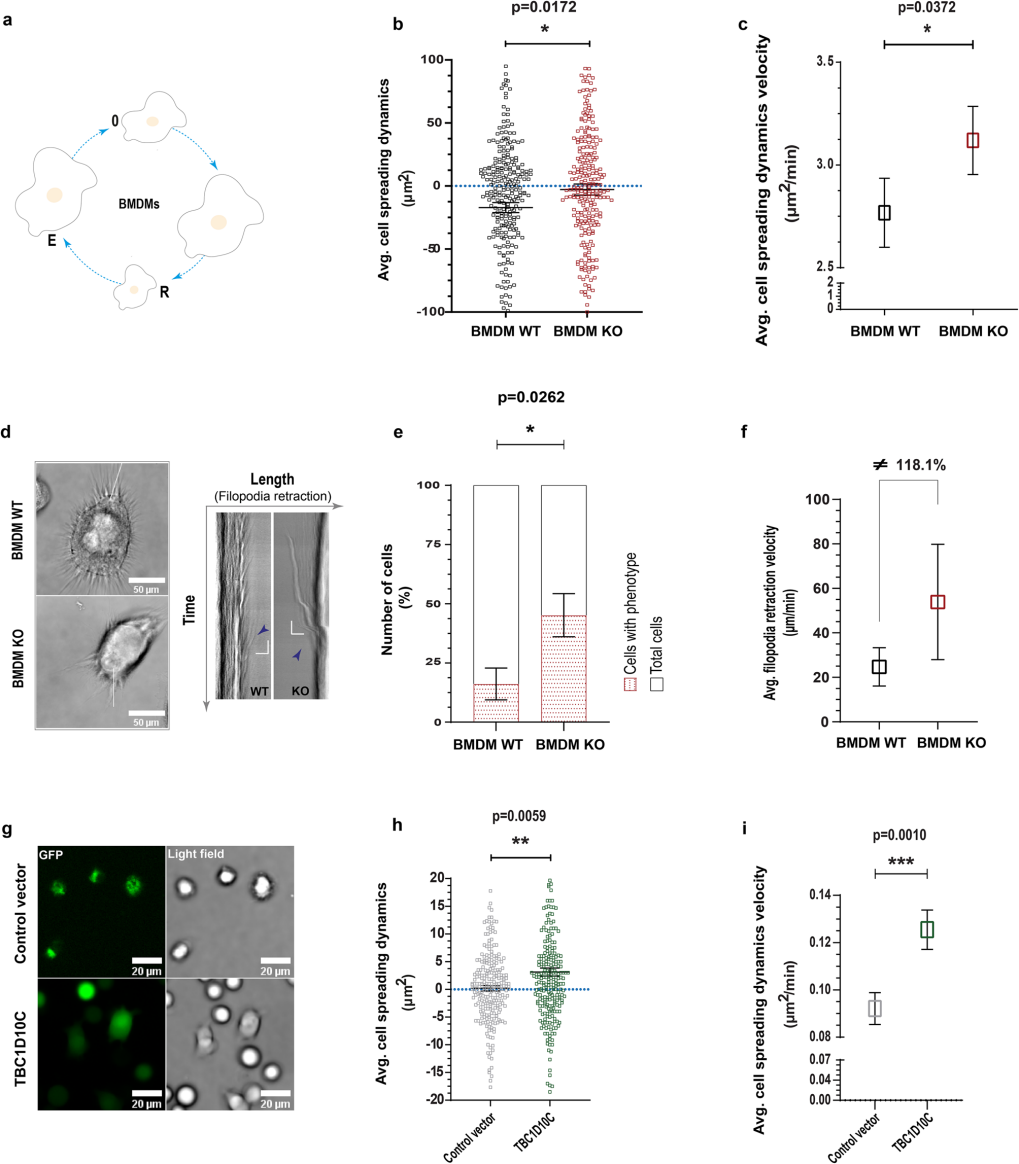
Analysis of cell spreading dynamics (aspect ratio dynamics). Living macrophages were imaged by time-lapse microscopy, and spreading was determined at multiple time points. (**a**) Drawing of cell spreading dynamics (0, initial cellular size; R, cellular body retraction; E, cellular body spreading); (**b**) average data for cell spreading dynamics (negative numbers are cellular retraction and positive numbers are cellular spreading; n = 31 BMDMs); (**c**) average data for velocity of cell spreading dynamics; (**d**), left: images of living BMDMs (sum of slices projection for xyt), right: kymographs showing filopodia abrupt retraction form selected ROIs (regions of interest, are indicated in while lines) in BMDMs; (**e**) percentage of BMDMs showing the abrupt retraction of filopodia; (**f**) average velocity of abrupt retraction of filopodia; **g**, confocal images of living Raw 264.7 transfected macrophages (28 Control vector and 29 TBC1D10C cells; sum of slices projection for xyzt) related to data for cell spreading dynamics and corresponding velocity in (**h**)and (**i**), respectively. Data were analyzed by the Mann–Whitney test (P < 0.05), and graphs show SEM.

Morphology and cytoskeleton dynamics are intimately related^26,27^. To gain more insights into the participation of TBC1D10C in the actin cytoskeleton, we measured cell spreading dynamics (aspect ratio dynamics) in macrophages by live cell imaging. This analysis indicated that KO BMDMs showed fewer cell spreading dynamics. Biologically, these KO cells were significantly less efficient at modifying their cellular size compared to WT BMDMs, which in turn tended to retract their cellular bodies during the analyzed time (Fig. 2a,b). Unexpectedly, despite the inefficiency of KO macrophages, the velocity at which these cells changed their cellular size was statistically greater than that of WT BMDMs (Fig. 2c). In the first instance, we expected that this velocity would also be lower. Why, then, was a greater velocity of aspect ratio dynamics recorded in KO BMDMs? To shed light on this effect, we analyzed multiple kymographs (through the cellular body of every cell; horizontal and vertical ROI every 1 µm). This analysis indicated that KO BMDMs frequently showed a phenotype consisting of detached filopodia that suddenly moved toward the cell body (Fig. 2d and Supplementary Movies 1–2). This phenotype was present in a significantly higher number of KO BMDMs than WT BMDMs (Fig. 2e) and seemed to affect cell spreading dynamics. In addition, quantification of filopodia retraction velocity showed more than double the velocity in KO cells (118.1% more; Fig. 2f), thus possibly increasing the quantification of the velocity of cell spreading dynamics (Fig. 2c). Afterward, we analyzed RAW 264.7 macrophages similarly and found that TBC1D10C overexpression was associated with an increase in both cell spreading dynamics and the corresponding velocity. In addition, macrophages overexpressing TBC1D10C showed a tendency to spread their cellular bodies (Fig. 2g,h,i). Together, our data strongly propose that TBC1D10C participates in macrophage spreading dynamics and that such participation correlates with defects of the cytoskeleton architecture.

### TBC1D10C contributes to *Burkholderia cenocepacia* phagocytosis by RAW 264.7 macrophages

**Figure 3:**
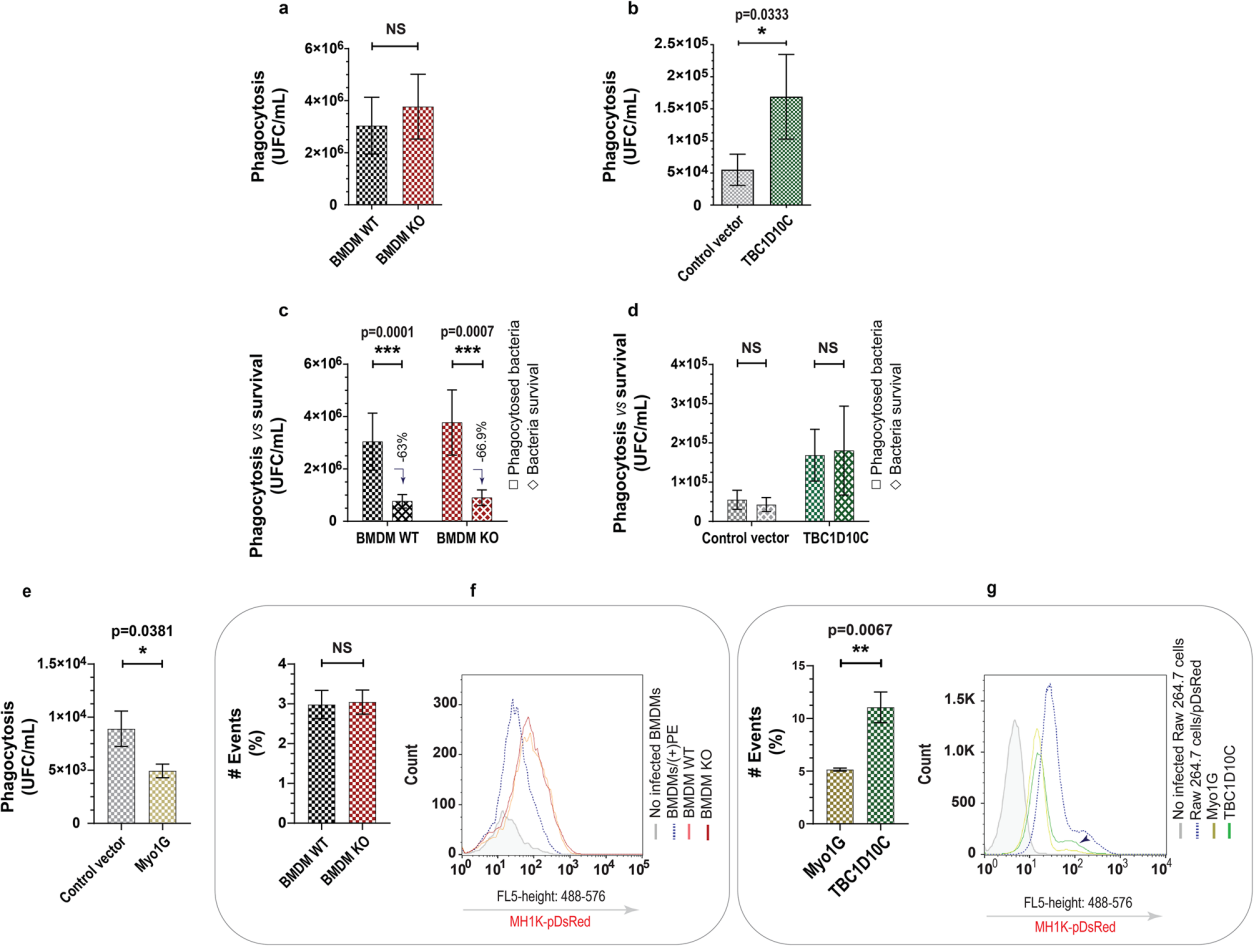
Evaluation of TBC1D10C’s effect on phagocytosis of *Burkholderia cenocepacia*. The effect of TBC1D10C elimination and overexpression on phagocytosis of *B. cenocepacia* was determined by CFU (colony forming unit) quantification in the gentamicine protection assay and flow cytometry at a MOI of 50 (multiplicity of infection). (**a**) Quantification of CFU (phagocytosed living bacteria at 1.5 h postinfection) in infected BMDMs (mice, n = 7); (**b**) quantification of CFU in infected Raw 264.7 transfected cells (control vector and TBC1D10C, respectively); (**c**) quantification of CFU in infected BMDMs at 5 h postinfection (comparison of phagocytosed, in (**a**) bacteria vs. bacteria survival); (**d**), quantification of CFU in infected Raw 264.7 transfected cells at 5 h-postinfection (control vector and TBC1D10C, respectively); (**e**) quantification of CFU in infected Raw 264.7 transfected cells (control vector and Myo1G; unrelated protein to TBC1D10C, respectively); (**f**) left: data of quantified events expressed as a percentage of BMDMs infected with transformed *B. cenocepacia* (MH1K-pDsRed) by flow cytometry, right, representative histogram of 2 independent experiments for (**f**)(BMDM/( +)PE is positive control for infection: MH1K-pDsRed); (**g**) left: data for quantified events expressed as a percentage of Raw 264.7 transfected macrophages infected with transformed *B. cenocepacia* (MH1K-pDsRed) by flow cytometry, right, representative histogram of 2 independent experiments for (**g**) (Raw 264.7 cells/pDsRed is positive control for infection; cells highly infected). Data were analyzed by the Mann–Whitney test (P < 0.05), and graphs show SEM.

The cytoskeleton participates in multiple cellular activities, including phagocytosis. In this process, macrophages must change shape through substantial membrane and cytoskeleton remodeling^28^. To determine whether TBC1D10C plays a role in this cellular function, we infected KO and WT BMDMs with *Burkholderia cenocepacia*^29^, an opportunistic pathogen that infects and survives in macrophages by altering actin polymerization^30^. To quantify only ingested bacteria, we used the gentamicin protection assay. TBC1D10C elimination in BMDMs was not associated with a significant increase or decrease in the amount of phagocytosed *B. cenocepacia* (Fig. 3a). However, its overexpression in RAW 264.7 macrophages correlated with a higher number of phagocytosed bacteria (Fig. 3b). Likewise, it has been reported that macropinocytosis contributes to the internalization of this bacterium by macrophages^31^; therefore, we evaluated this endocytic pathway using 10 kDa dextran (a fluid-phase marker ingested by macropinocytosis)^32^, but no significant differences were observed (Supplementary Fig. 2). Furthermore, by evaluating the capacity of macrophages to kill ingested bacteria, we determined that both WT and KO BMDMs eliminated intracellular bacteria similarly (Fig. 3c), whereas RAW 264.7 cells transfected with control vector and TBC1D10C were unable to efficiently kill phagocytosed microorganisms (Fig. 3d). This indicates a differential capacity to control *B. cenocepacia* by BMDMs and RAW 264.7 macrophages; consequently, the higher number of internalized bacteria upon TBC1D10C overexpression was not specifically related to differential elimination of bacteria or increased macropinocytosis. On the other hand, to validate the specific effect of TBC1D10C on *B. cenocepacia* phagocytosis, we transfected RAW 264.7 cells with Myosin 1G, an unrelated protein to TBC1D10c, which is not known to participate in the phagocytosis of *B. cenocepacia.* Again, we evaluated phagocytosis and found that Myo1g overexpression in RAW 264.7 macrophages was not associated with increased phagocytosis of this bacteria (Fig. 3e), thus indicating a specific contribution of TBC1D10C to *B. cenocepacia* phagocytosis. As further evidence, we evaluated phagocytosis by flow cytometry to more directly analyze the uptake of *B. cenocepacia*. With this analysis, we confirmed that elimination of TBC1D10C did not correlate with important changes in phagocytosis for *B. cenocepacia* (Fig. 3f), whereas TBC1D10C overexpression did so (Fig. 3g). Our results indicate that TBC1D10C contributes to the internalization of *B. cenocepacia* by RAW 264.7 macrophages.

### TBC1D10C is a protein intimately associated with cytoskeleton-plasma membrane reorganization and tension

**Figure 4:**
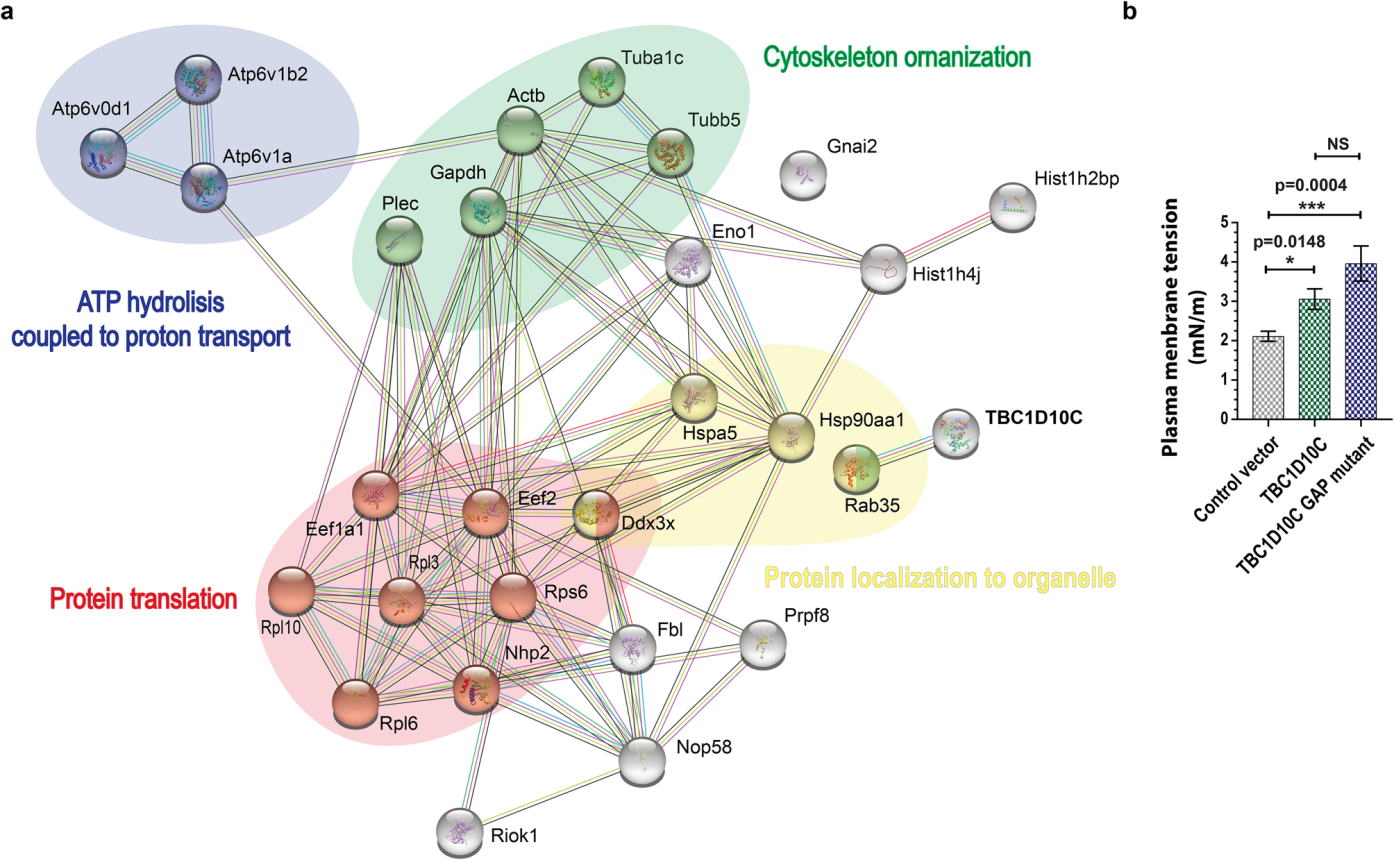
Protein–protein interaction (PPI) analysis of TBC1D10C-interacting proteins and measurement of cell membrane stiffness. (**a**) The TBC1D10C interaction network was built from the STRING database with a confidence score > 0.4. The network contains 28 nodes and 90 edges. PPI analysis indicated multiple TBC1D10C-associated complexes. TBC1D10C is highlighted in bold capital letters; (**b**) membrane stiffness of TBC1D10C-transfected Raw264.7 macrophages was determined by atomic force microscopy (AFM). (20, control vector; 30, TBC1D10C; 16 TBC1D10C GAP mutant). Data were analyzed by the Mann–Whitney test (P < 0.05), and the graph shows SEM.

Cell spreading and phagocytosis are related cellular processes that notably require cytoskeleton-plasma membrane reorganization as well as the participation of multiple proteins^28,33,34^; our data indicate that TBC1D10C participates in such cellular functions. To better understand the mechanistic contribution of TBC1D10C to cell spreading and phagocytosis, we carried out proteomic analysis using stably transfected RAW 264.7 cells (GFP tag-TBC1D10C). TBC1D10C was immunoprecipitated to identify proteins in the complex by mass spectrometry followed by bioinformatics analysis. This led us to identify 27 TBC1D10C-interacting proteins (Table 1), which were functionally grouped in cytoskeleton organization, protein localization to organelles, protein translation, and ATP hydrolysis coupled to proton transport (Fig. 4a), in addition to others (Supplementary Table 1). The proteins identified and functionally grouped in cytoskeletal organization are tubulin, plectin, Gapdh, and Rab35.

**Table 1:** List of TBC1D10C-interacting proteins in samples from RAW 264.7 macrophages.

UniProt ID	Protein name	Description	MW (kDa)	Scores (Mascot)	#Peptides	SC (%)
Q9QXS1	PLEC_MOUSE	Plectin	533.9	182.1	4	1.6
P47911	RL6_MOUSE	60S ribosomal protein L6	33.5	149.3	2	10.8
Q62167	DDX3X_MOUSE	ATP-dependent RNA helicase DDX3X	73.1	97.8	1	1.8
Q99PV0	PRP8_MOUSE	PremRNA-processing-splicing Factor 8	273.4	95.3	1	0.6
Q922Q2	RIOK1_MOUSE	Serine/threonine-protein kinase RIO1	64.9	177.4	4	7.9
P20029	GRP78_MOUSE	78 kDa glucose-regulated protein	72.4	167.8	3	5.8
Q6PHN9	RAB35_MOUSE	Ras-related protein Rab-35	23.0	212.7	4	26.9
P62814	VATB2_MOUSE	V-type proton ATPase subunit B, brain isoform	56.5	187.2	4	11.9
P51863	VA0D1_MOUSE	V-type proton ATPase subunit d 1	40.3	125.4	2	7.7
P50516	VATA_MOUSE	V-type proton ATPase catalytic subunit A	68.3	111.7	2	6.5
P08752	GNAI2_MOUSE	Guanine nucleotide-binding protein G(i) subunit alpha-2	40.5	89.4	2	7.9
P62806	H4_MOUSE	Histone H4	11.4	330.8	6	47.6
P62754	RS6_MOUSE	40S ribosomal protein S6	28.7	179.7	3	15.7
Q8CGP2	H2B1P_MOUSE	Histone H2B type 1-P	14.0	146.7	3	26.2
P35550	FBRL_MOUSE	rRNA 2'-O-methyltransferase fibrillarin	34.3	140.5	3	12.5
Q6ZWV3	RL10_MOUSE	60S ribosomal protein L10	24.6	138.3	2	11.2
Q9CRB2	NHP2_MOUSE	H/ACA ribonucleoprotein complex subunit 2	17.2	110.6	1	11.1
P27659	RL3_MOUSE	60S ribosomal protein L3	46.1	107.1	2	6.9
Q6DFW4	NOP58_MOUSE	Nucleolar protein 58	60.3	94.5	1	2.4
P10126	EF1A1_MOUSE	Elongation Factor 1-alpha 1	50.1	202.1	2	9.5
P68373	TBA1C_MOUSE	Tubulin alpha-1C chain	49.9	187.0	3	12.2
P17182	ENOA_MOUSE	Alpha-enolase	47.1	167.9	3	12.7
P60710	ACTB_MOUSE	Actin, cytoplasmic 1	41.7	134.5	2	11.7
P07901	HS90A_MOUSE	Heat shock protein HSP 90-alpha	84.7	119.5	4	8.6
P99024	TBB5_MOUSE	Tubulin beta-5 chain	49.6	112.5	2	7.9
P58252	EF2_MOUSE	Elongation Factor 2	95.3	86.9	3	7.6
P16858	G3P_MOUSE	Glyceraldehyde-3-phosphate dehydrogenase	35.8	84.0	1	7.2

Conserved proteins identified between the GFP-tagged control vector and GFP-tagged TBC1D10C were discarded (results from 4 independent experiments). SC: Sequence coverage. TBC1D10C was identified but not included in the table.

The identification of Rab35 as a TBC1D10C-interacting protein in macrophages, in principle, suggests a specific role for TBC1D10C GAP activity in cell spreading and phagocytosis. Therefore, we performed experiments to evaluate the contribution of this GAP activity as well as its biological effect on Rab35. This activity was represented by the use of the TBC1D10C-GAP mutant (R141K) and Rab35 DN (Rab35 dominant negative, S22N, which theoretically could represent the biological effect of TBC1D10C’s GAP activity) to modulate such cellular functions. These experiments indicated that functional TBC1D10C’s GAP domain is dispensable for macrophage spreading dynamics (Supplementary Fig. 3) but appears necessary for internalization of *B. cenocepacia* in the phagocytic process in RAW 264.7 cells (Supplementary Fig. 4). To shed light on these differences, we conducted another set of proteomic analyses for cells expressing the TBC1D10C-GAP mutant and Rab35 DN. We only identified Rab35, Histone H2A.V, and Histone H4 as TBC1D10C-R141K-interacting proteins in macrophages (TBC1D10C itself was also identified; 3 different experiments), thus indicating that the functional GAP domain in TBC1D10C is not essential for the TBC1D10C interaction with Rab35 in macrophages but is somehow important for the interaction with other TBC1D10C-interacting proteins. In the case of Rab35 DN, we identified 41 Rab35-S22N-interacting proteins in RAW 264.7 macrophages (Supplementary Table 2) and grouped them functionally (Supplementary Table 3). Two of the Rab35-S22N interactions we identified were particularly interesting: CD14 and Myo1G (Supplementary Fig. 5). Previously, we reported that Myo1G elimination in lymphocytes affected cell spreading and phagocytosis via a mechanism involving membrane tension, a physical property generated by the interaction between the actin cytoskeleton and the plasma membrane^35^. Furthermore, some of the TBC1D10C-interacting proteins (plectin, actin, tubulin) are known linkers of the plasma membrane to the cytoskeleton^26,36–39^, constituting a mechanism that influences plasma membrane tension. Accordingly, to demonstrate whether membrane tension was influenced by TBC1D10C, we measured this physical characteristic in transfected cells by atomic force microscopy. TBC1D10C overexpression in RAW 264.7 macrophages was associated with increased membrane tension (Fig. 4b) and was significantly independent of functional GAP activity. Together, our results show that TBC1D10C is a functional link of the cytoskeleton-plasma membrane that impacts its tension.

### TBC1D10C has a predicted intrinsically disordered region with protein-binding properties at the cytoskeleton level

**Figure 5:**
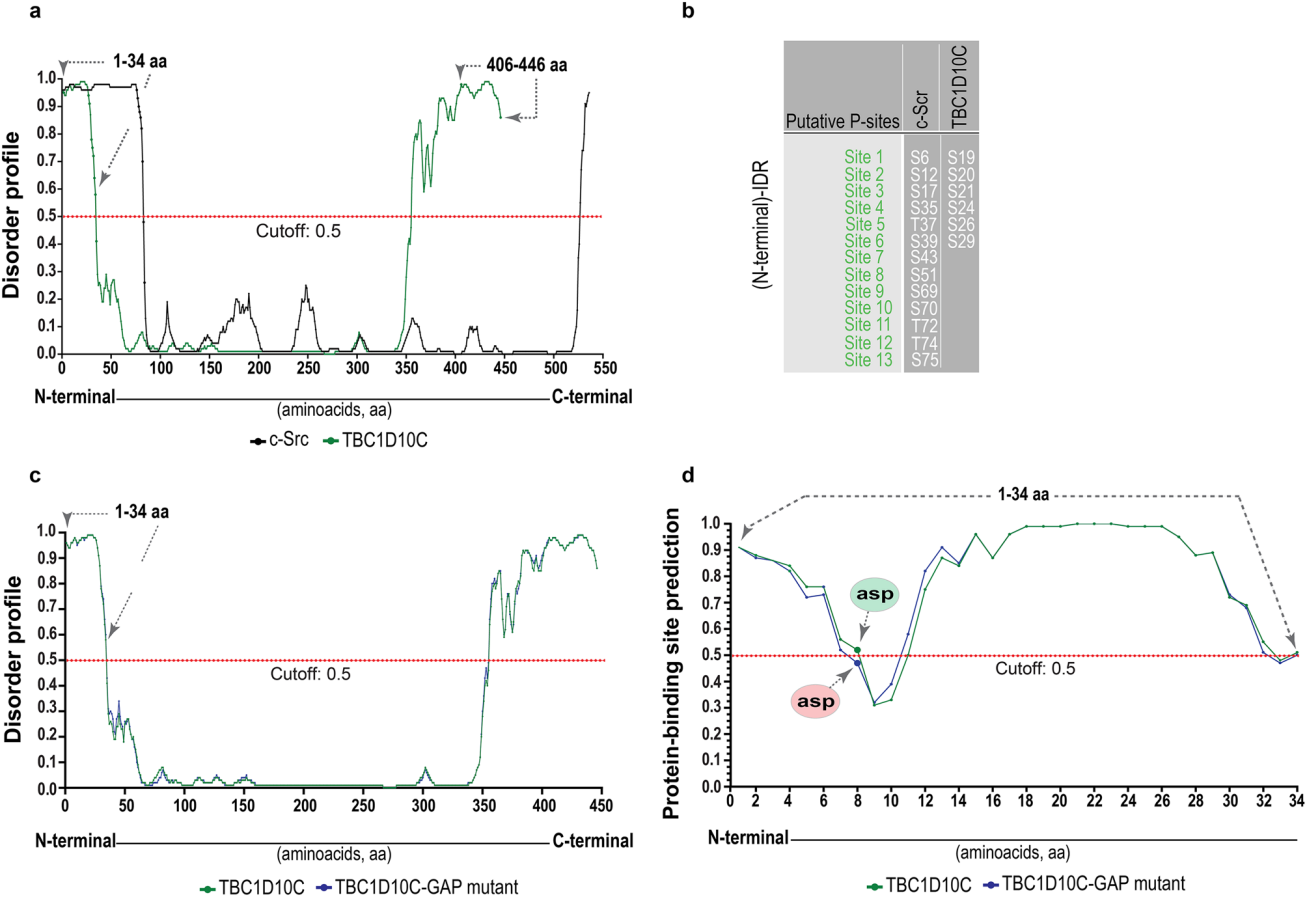
Prediction of intrinsically disordered regions (IDRs) and phosphorylation sites in TBC1D10C. (**a**) Intrinsic disorder profiles of the amino acid composition of human TBC1D10C (green dotted line) and human Src (black dotted line) are plotted. Regions of amino acids are considered disordered above the confidence score (cutoff: 0.5; gray dashed line). A (N-terminal)-IDR in TBC1D10C similar to the (N-terminal)-IDR in Src as well as a (C-terminal)-IDR in TBC1D10C that includes the calcineurin interacting region (406–446 aa); (**b**) 13 and 6 putative phosphorylation sites are shown for the (N-terminal)-IDR in Src and TBC1D10C, respectively; (**c**) intrinsic disorder profile of the amino acid composition of human TBC1D10C (green dotted line) and human TBC1D10C GAP mutant (blue dotted line) are plotted; (**d**) prediction of protein-binding sites in the (N-terminal)-IDR of TBC1D10C (green dotted line) and TBC1D10C GAP mutant (blue dotted line) (residue 8, asp, aspartic acid is significantly different predicted as a protein-binding site as a consequence of R141K mutation in TBC1D10C).

TBC1D10C has an interacting calcineurin domain and a GAP domain. This GAP domain is responsible for the inactivation of small GTPases such as Ras and Rab35^2,5^, therefore playing a significant role in the GAP and small GTPase interaction^40,41^. To obtain more insights into the interaction between TBC1D10C and TBC1D10C–interacting proteins, we searched for other domains or motives in TBC1D10C by bioinformatics analysis. We identified two intrinsically disordered regions (IDRs) in TBC1D10C (Fig. 5a): one on the C-terminus that coincided with the interacting calcineurin domain (residues 406–446) and another in the N-terminal region (residues 1–34). Interestingly, the (N-terminal)-IDR in TBC1D10C is similar to the recently reported (N-terminal)-IDR in Src kinase, which is essential to form Src dimers as a mechanism for autophosphorylation and phosphorylation of Src substrates, as well as for protein and/or cytoskeletal macromolecule interactions^42^. In addition, similar to the (N-terminal)-IDR in Src kinase, the (N-terminal)-IDR in TBC1D10C has multiple putative phosphorylation sites (Fig. 5b), thus reinforcing the notion of a biological role of the (N-terminal)-IDR in TBC1D10C. Importantly, we found that the disorder profile for TBC1D10C was similar to that for the TBC1D10C GAP mutant (Fig. 5c), but one of the sites in the (N-terminal)-IDR was predicted to be significantly different as a consequence of the GAP mutation (R141K) (Fig. 5d). These results suggest that the functional GAP domain in TBC1D10C is important for the interaction with TBC1D10C–interacting proteins in RAW 264.7 macrophages.

## Discussion

TBC1D10C is functionally related to the oncogenic proteins Ras and Rab35^2,5,15^; however, little is known about the function of this GAP in cellular physiology; in particular, its role in macrophages is not known. Here, we analyzed the participation of TBC1D10C in the cellular physiology of macrophages and identified TBC1D10C as a functional linker for cytoskeleton-plasma membrane reorganization in cellular pathways for cell spreading and phagocytosis, thus modulating the activity of macrophages.

The first insights into the role of TBC1D10C in macrophage physiology came from important differences observed in cytoskeletal architecture. Specifically, the spreading area (size) of macrophages visualized by F-actin is one of the main components of the cytoskeleton^36^. These differences under TBC1D10C elimination and overexpression correlated with less and more cell spreading in these immune cells, respectively (Fig. 1). We inferred that TBC1D10C positively influenced the spreading ability of macrophages in a manner related to cytoskeletal dynamics. However, how can TBC1D10C positively regulate cell spreading? In this process, cytoskeletal and plasma membrane modifications are essential and impact morphology^43–45^. By studying cytoskeleton-plasma membrane dynamics by live cell imaging in macrophages, we found that TBC1D10C KO cells were deficient in cell spreading dynamics. Unlike WT BMDMs, KO BMDMs had defects when dynamically reshaping their morphology, either toward cell spreading or cell retraction (Fig. 1a,b). These functions are in balance and are opposite cellular processes. Specifically, cell spreading is characterized by the formation of filopodia and actin polymerization^45^. KO BMDMs were associated with a significant phenotype consisting of filopodia that abruptly moved toward the cell body (Fig. 2d,e and Supplementary Movies 1–2). Such defects observed in KO cells seemed to affect the balance between cell spreading and cell retraction (Fig. 2b). In addition, it is possible that the phenotype described above may have contributed to an increase in the velocity of cell spreading dynamics in BMDM KO (Fig. 2c), perhaps by adding acceleration (Fig. 2f) derived from abrupt filopodia retraction to the quantified velocity^46^. Furthermore, TBC1D10C overexpression in RAW 264.7 cells was associated with the opposite outcome in the same cellular process; transfected macrophages were more dynamic in modifying their morphology, especially toward cell spreading (Fig. 2g,h). Furthermore, our proteomic analysis allowed us to identify multiple TBC1D10C-interacting proteins (Table 1) that were functionally grouped by cytoskeleton organization (Fig. 4), including Rab35 and actin, thus highlighting the role of TBC1D10C in cytoskeleton-plasma membrane dynamics. Additionally, identification of Rab35 suggested that, as in lymphocytes^5^, this small GTPase and cellular processes in which it participates could also be modified by TBC1D10C’s GAP activity in macrophages. A role for Rab35 in cell spreading has been documented; for example, when this function (also referred to as frustrated phagocytosis; in IgG-coated coverslips) was evaluated in BMDMs transfected with Rab35 DN and Rab35 CA (Rab35 constitutively active), transfected BMDMs-Rab35 DN showed decreased spreading ability^20^. Our results showed that Rab35 DN was not associated with increased cell spreading in RAW 264.7 macrophages and that the effect of TBC1D10C in promoting cell spreading was independent of TBC1D10C’s GAP activity (Supplementary Fig. 3). Additionally, there is an antagonistic operating relationship between Rab35 and Arf6 in many cell types that regulates diverse cellular processes^18–21,23,47–52^. TBC1D10C is functionally related to Arf6, seemingly as an effector protein (meaning a protein that can interact with active small GTPases, bound to GTP, and promote and/or participate in cellular pathways)^13,14^, and Arf6 also participates in cell spreading. It was reported that ectopic expression of Arf6 DN (dominant negative) on fibroblasts was able to block spreading, but activation of Arf6 by QS11 (ARF GAP inhibitor used to stimulate Arf6 activation) restored cell spreading and behaved similarly to control cells (on integrin ligands)^53^. Moreover, regardless of functional (TBC1D10C) or inactive (TBC1D10C-R141K) GAP activity, Arf6 was not identified as one of the TBC1D10C-interacting proteins in macrophages, thus suggesting other possible protein targets. In conclusion, TBC1D10C promotes cell spreading in macrophages in a manner closely associated with actin-cytoskeleton dynamics but significantly independent of GAP activity.

On the other hand, in the phagocytosis process, we determined that elimination of TBC1D10C in BMDMs did not affect the phagocytosis of *B. cenocepacia* (Fig. 3a), perhaps by genetic compensatory mechanisms^54^ or by important differences in phagocytosis between BMDMs and RAW 264.7 cells, where BMDMs are described to be more phagocytic in terms of both internalization and phagosome maturation^55^. This agrees with our results showing that BMDMs phagocytosed more bacteria and eliminated the studied microorganisms more efficiently than RAW 264.7 cells (Fig. 3a–d). Conversely, regarding TBC1D10C elimination, the overexpression of TBC1D10C increased the phagocytic activity of RAW 264.7 macrophages for *B. cenocepacia* (Fig. 3b,e,g), more specifically its internalization (Fig. 3b,g); in this regard, the GAP activity of TBC1D10C seemed to be required (Supplementary Fig. 4). Together, these results suggest a specific contribution of TBC1D10C in a cellular pathway involved in *B. cenocepacia* internalization by RAW 264.7 macrophages. Moreover, cell spreading and phagocytosis are intimately related processes that require cytoskeleton-plasma membrane remodeling as well as a determinant component of membrane tension^28,33,34^. TBC1D10C interacts with proteins associated with cytoskeletal reorganization (Fig. 4a), and specifically, independent of functional GAP activity, TBC1D10C interacts with Rab35 in RAW 264.7 macrophages. We inferred that TBC1D10C could be related to cellular pathways associated with Rab35. One of the Rab35-S22N-interacting proteins we identified is Myo1G (Supplementary Table 2), and we previously reported that membrane tension was affected in Myo1G knockout lymphocytes. Therefore, we analyzed transfected macrophages expressing TBC1D10C to demonstrate whether membrane tension was altered. We determined that TBC1D10C overexpression changes the plasma membrane tension of macrophages (Fig. 4b). This correlation was not impeded by a mutation (R141K) that inactivates the GAP activity of TBC1D10C. Furthermore, it has been reported in RAW 264.7 cells that, derived from actin polymerization during filopodia extension, there is an increase in membrane tension and cell spreading as the phagosome forms^28,33,34^. The above is consistent with our results that relate TBC1D10C with increased membrane tension and increased cell spreading (Figs. 1c, 2h) as well as with increased phagocytosis (Fig. 3b), strongly suggesting the contribution of the TBC1D10C effect on membrane tension to cellular processes where this physical characteristic is a master regulator, including cell spreading and phagocytosis. Additionally, our results suggest that other (than membrane tension) important elements related to TBC1D10C contribute to the phagocytosis of *B. cenocepacia*, given the significant differences observed in this process when evaluating the functional GAP activity (Supplementary Fig. 4).

For example, the effect of TBC1D10C on phagocytosis of *B. cenocepacia* seems to occur at the level of initial phases of phagocytosis (macropinocytosis as well as later phases of phagocytosis for bacterial killing were not significantly involved, Supplementary Figs. 2 and 3d). Many works have reported the contribution of Rab35 to phagocytosis; for instance, this small GTPase participates in the early stages of phagosome formation during the uptake of diverse particles in RAW 264.7 cells^20,23,48,55^. Another interesting Rab35-S22N interacting protein we identified is CD14, which was functionally grouped in the innate immune response (Supplementary Fig. 4) and, in turn, is a pattern recognition receptor (PRR)^56^ that contributes to phagocytosis of gram-negative bacteria^57,58^. Specifically, it has been reported that part of the immune response against *B. cenocepacia* is mediated by the association of lipopolysaccharide (LPS) components with CD14 in macrophages^59,60^. These results allow us to infer that the participation of TBC1D10C in phagocytosis of *B. cenocepacia* could be, at least in part, a cellular pathway associated with bacterial recognition. However, defining the upstream and downstream participation of CD14 in the increased phagocytic activity of *B. cenocepacia* in macrophages overexpressing TBC1D10C is a future objective.

**Figure 6:**
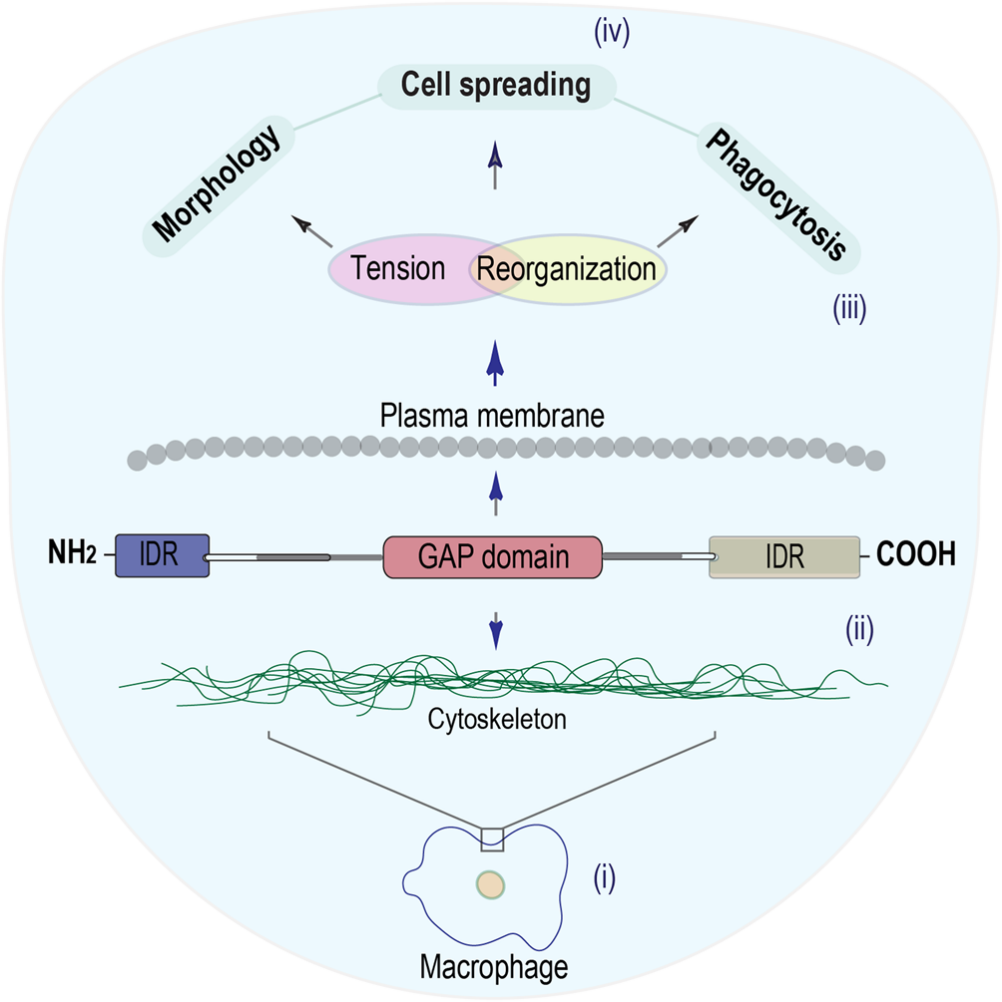
Model of the TBC1D10C’s role in macrophages. We propose, according to our results, that TBC1D10C is a functional linker for the cytoskeleton-plasma membrane (possibly contributing the N-terminal IDR of TBC1D10C), to cytoskeleton-plasma membrane tension and reorganization for cellular processes such as cell spreading, morphology, and phagocytosis.

Finally, we carried out bioinformatics analysis to gain insights into the interaction of TBC1D10C and TBC1D10C-interacting proteins, which could provide more information about the role of TBC1D10C in cell spreading and phagocytosis. We identified two intrinsically disordered regions (IDRs) in TBC1D10C: one TBC1D10C-IDR was identified on the C-terminus and coincided with the interacting calcineurin domain (carboxy terminus; residues 406–446), and the other was an unreported (N-terminal)-IDR (residues 1–34) (Fig. 5a). It has been reported that IDRs play important roles in modulating membrane properties as well as cytoskeleton-related functions^61–64^. In addition, a biological role for an IDR in Src kinase was recently revealed. Specifically, this IDR was located in the amino terminus (N-terminal), and it was described to be essential for Src dimerization as a mechanism of regulation. Additionally, it was suggested that this IDR could contribute, at the macromolecular level in the plasma membrane, as a signaling hub for protein–protein interactions^42^. Interestingly, the (N-terminal)-IDR in TBC1D10C is similar to the (N-terminal)-IDR in Src (Fig. 5a). In addition, as the (N-terminal)-IDR in Src kinase, the (N-terminal)-IDR in TBC1D10C has putative phosphorylation sites (Fig. 5b), suggesting a possible biological role for the (N-terminal)-IDR in TBC1D10C^65,66^. Furthermore, the disorder profiles for TBC1D10C and the TBC1D10C GAP mutant were similar (Fig. 5c), but in the (N-terminal)-IDR, a predicted protein-binding site was shown to be significantly different as a consequence of the R141K mutation in TBC1D10C (Fig. 5d); this is possibly related to the difference in the number of TBC1D10C-interacting proteins before the mutation. This result may be due to changes in the complex conformation between the GAP protein and the small GTPase^40,41^. Together, it is possible that the (N-terminal)-IDR in TBC1D10C plays a biological role in the interaction between TBC1D10C and TBC1D10C-interacting proteins and contributes to the functional link between TBC1D10C and the cytoskeletal membrane during spreading and phagocytosis. To summarize, TBC1D10C participates in macrophage physiology through a functional link with the cytoskeleton-plasma membrane that impacts its tension and reorganization for cell spreading and phagocytosis (Fig. 6).

## Materials and methods

### Mice

Mice were maintained in pathogen-free conditions at UPEAL (Unit for Production and Experimentation of Animals of Laboratory) from CINVESTAV. Their manipulation was performed according to the standard and operating protocols approved by the Animal Care Use Committee, Bioethics and Research of CINVESTAV (Protocol **145–15**). The general recommendations in the ARRIVE guidelines 2.0 (https://arriveguidelines.org/) were followed in our study. Our sample size was small. We did not use any method to generate the randomization sequence. All methods were performed in accordance with the relevant guidelines and regulations. We used an equal number of animals in the WT and KO groups for this study. We used TBC1D10C floxed and knockout (KO) mice generated on the C57BL/6 background. To obtain TBC1D10C KO mice, TBC1D10C floxed mice were crossed with β actin-cre mice (Jackson Laboratories, Bar Harbor, ME, USA) to generate full conventional KO. For genotyping, tail DNA was extracted by the HotShot protocol, and the *TBC1D10C* gene was amplified by PCR using specific primers: **floxed mice**—5’ loxP side: TBC lxpF, ccttcccctaaagctgaaca and TBC lxpR, tttggctgtcccctaaaaca; 3’ side: TBC FrtgtF, tgacagagtgtcaggagagca and TBC FrtgtR, gcacattaccagcccaaact. **KO mice**—TBC lxpF, ccttcccctaaagctgaaca and TBC FrtgtR, gcacattaccagcccaaact (to detect WT allele band, we used the primer TBC lxpR, tttggctgtcccctaaaaca).

### Reagents and antibodies

RPMI 1640 medium (with and without phenol red) supplemented with 10% FBS and 4 g/L glucose (unless otherwise indicated); dextran, Texas red™, 10,000 MW (D1828); Alexa phalloidin 488 (Cat. A12379), dil. (PBS) 1:625 for BMDMs; ActinRed™ 555 ReadyProbes™ Reagent (Cat. R37112); Halt protease inhibitor cocktail™ (Cat. 78,429); all from Thermo Fisher Scientific, Rockford, IL, USA; HotStar Taq master mix kit (Qiagen, Valencia, CA, USA, Cat. 203,446); trypsin gold, mass spectrometry grade (Promega, Madison WI, USA, Cat. V5280); goat polyclonal anti-carabin (Abcam; ab77625), 1 µg/ml; beta actin monoclonal antibody (Sigma, St. Louis, MO, USA, Cat. 15G5A11/E2), dil. 1:5000; Alexa 680 anti-mouse (Thermo Fisher Scientific, A28183); Alexa 680 anti-rabbit (Thermo Fisher Scientific, A27042).

### Macrophages and infection (gentamicin protection assay and flow cytometry)

**In vitro:** The RAW 264.7 (ATCC® TIB-71™) macrophage cell line was stably transfected with human TBC1D10C (wild type), TBC1D10C GAP mutant (R141K), Rab35 DN (dominant negative; S22N), Myo1g WT, and control empty vector (henceforth, control vector) (pDest732, N-terminal GFP *tag*)^5^ using Lipofectamine. Transfected cells with similar GFP expression were sorted (by FL1 mean fluorescence intensity) and cultured in RPMI 1640 supplemented medium with G418 at 600 μg/mL (atmosphere, oxygen, 95%; CO_2_, 5%; 37 °C). **Ex vivo:** Bone marrow-derived macrophages were obtained from bone marrow from C57BL/6 KO mice treated with medium for differentiation (V/V, supernatant medium of L929 cells, 30%; RPMI 1640 medium supplemented with 10% FBS, 70%), as previously described^67^. For the gentamicin protection assay, we used *Burkholderia cenocepacia* MH1K^29^, kindly donated by Dr. Miguel A. Valvano (Queens University, Belfast, UK), which is susceptible to gentamicin (this antibiotic does not penetrate eukaryotic cells). To infect RAW 264.7 cells (3.5 × 10^5^; seeded the day prior to infection), we used 12-well plates and 6-well plates for BMDMs (2 × 10^6^; differentiated as earlier). Macrophages were infected with this bacterium (cultured in LB broth medium with constant shaking; 37 °C, 16 h/180 r.p.m.) at a multiplicity of infection (MOI) of 50 for 2 h (atmosphere, oxygen, 95%; CO_2_, 5%; 37 °C) in RPMI medium without antibiotics (800 µL or 3 mL for 12- and 6-well plates, respectively). Infection was synchronized by centrifugation (1200 r.p.m/2 min). After 2 h of incubation, the cells were washed (twice with sterile PBS; 1 ml and 2 ml according to the well plate), and the medium was changed to RPMI medium plus gentamicin (60 µg/mL) for 1.5 h (to kill extracellular bacteria; same atmosphere). Finally, cells were washed once (same as above) and lysed using sterile water (10 min and pipetting); this time point (1.5 h post infection) was considered phagocytosis/bacteria invasion, while 5 h post infection was considered microbicidal macrophage ability/bacteria survival (with respect to 1.5 h post infection). The number of bacteria (CFU; colony forming unit) was determined by serial dilutions. For flow cytometry, after 2 h of incubation (as mentioned above), cells were washed (twice with sterile PBS), detached (4 °C PBS-EDTA, 2 mM; 10 min, to cover the cell monolayer), and fixed (PBS- paraformaldehyde 2%) until analysis.

### Triparental mating

This technique is a form of transformation by bacterial conjugation consisting of using donor, helper, and receiving strains (*E. coli*-pRK2013, conjugative plasmid, helper, km-resistant; *E. coli*-pDsRed; donor, Cm-resistant; *B. cenocepacia*, receiving strain, sensitive to km and Cm, resistant to polymyxin B). Bacteria were grown overnight (180 r.p.m/16 h, 37 °C) in the presence of appropriate antibiotics (*E. coli-*pDsRed, Cm, 100 µg/mL; *E. coli*-pRK2013, km, 100 µg/mL; *B. cenocepacia,* polymyxin B, 600 µg/mL). A pellet of bacteria was collected by centrifugation (3500 r.p.m/5 min) and washed with sterile PBS (resuspension and centrifugation as above). Bacteria were quantified by optical density to 600 nm (OD_600 nm_), and similar amounts of bacteria (*E. coli-*pDsRed, 0.45; *E. coli*-pRK2013, 045; *B. cenocepacia*, 0.5) were resuspended in 100 µL of LB medium, inoculated with a glass spreader in agar plates, and incubated (24 h, 37 °C). Subsequently, many bacterial colonies were obtained, resuspended in LB medium, and inoculated using serial dilutions (60–80 µL, as indicated above) in selection agar plates (Cm, 100 µg/mL + polymyxin B, 600 µg/mL). Then, selection agar plates were incubated (48 h, 37 °C). Afterward, colonies were observed by confocal microscopy.

### Cell spreading and cell spreading dynamics (aspect ratio dynamics)

**Cell spreading**: RAW 264.7-transfected cells (1 × 10^6^) were seeded onto coverslips in 6-well plates the day before the assay. For BMDMs, we seeded bone marrow cells (1.5 × 10^6^) for differentiation as previously described. For both types of cells, the medium was discarded, and monolayers were washed (once with PBS). Macrophages were fixed (paraformaldehyde 4%; 10 min), permeabilized (PBS-Tween 0.05%; 5 min) and F-actin stained using phalloidin (ready to use ActinRed™ 555 ReadyProbes™ Reagent; and Alexa phalloidin 488 at dil. 1:625 in PBS) following the manufacturer’s instructions. Coverslips were mounted onto slices to acquire confocal images (using a LEICA TCS SP8X confocal microscope; xyz planes), of which we generated projections (sum of slices). Later, using ImageJ software, we defined regions of interest (ROIs) for the F-actin perimeter of each cell to calculate the spreading area. **Cell spreading dynamics (aspect ratio dynamics):** Similar to previous reports^68–70^, we used two related assays to study the effect of TBC1D10C on cell spreading dynamics in living BMDMs and RAW 264.7-transfected cells. Bone marrow cells (1.5 × 10^6^) were seeded onto 35 × 10 mm glass bottom (0.17 mm) plates with 2.5 mL of medium for differentiation. Once differentiated, the medium was discarded, the cells were washed (sterile PBS, 1 mL), and new medium was added (37 °C RPMI with supplementation and without phenol red, 2 mL). On the other hand, RAW264.7-transfected cells were detached as described earlier and placed onto the same glass bottom plates (10,000 cells) (37 °C RPMI with supplementation and without phenol red plus G418, 2 mL). BMDMs and RAW 264.7 macrophages were time-lapsed by confocal microscopy (BMDMs, 37 °C; 40x, xyt planes, acquisition every 2 s for 30 min; 37 °C, 40x, xyzt planes, acquisition ≈ 53 s for ≈ 111 min). Aspect ratio dynamics were calculated for each individual BMDM and RAW 264.7 cell by defining ROIs (cell perimeter) at different time points; an average cell area change was estimated. Additionally, by using ImageJ software, multiple kymographs were generated for each cell (left to right and top to bottom; 1 µm output spacing) to cover the entire BMDM.

### Measurement of cell membrane stiffness (cell membrane elasticity)

To measure the cell membrane stiffness (elasticity of the cell membrane), we used an atomic force microscope (Model XE-Bio, Park Systems) equipped with a liquid cell. Si_3_N_4_ probes (MSNL-10, Bruker Nano Inc.) with a nominal pyramidal tip radius of 2 nm were used. Before measurements, all probes were calibrated using the thermal noise method to obtain an accurate value of elastic force constant for each cantilever (e.g., k = 62 mN/m for cantilever MSNL-10-D). All measurements were performed in a liquid environment (RPMI medium without supplementation and phenol red) at 22 °C. For each cell, a group of at least 10 force vs. distance (FvsD) curves with 1024 ordered pairs (distance, force) were acquired. To achieve optimal FvsD curves, the vertical probe displacement was adjusted, and the linear force vs. displacement was recorded. The vertical probe displacement was 300 nm at a vertical speed of 10 µm/s. The average of the 10 curves was used to obtain the membrane stiffness for each cell. Data adjustment was performed with a homemade MATLAB routine. The elastic model used to estimate the elastic force constant for cell membranes was the mechanical model for two springs in series given by Hooke’s law:

$$F=-[km*kc/(km+kc)](z-z0)$$

where F is the probe applied force, km is the membrane elastic constant, kc is the cantilever constant, z is the vertical probe displacement, and z0 is the point of contact between the cell membrane and probe tip. All measurements were performed at the highest point of the cell, that is, on the nucleus of the cell.

### Immunoprecipitation, gel digestion, and mass spectrometry

***Immunoprecipitation:*** Transfected RAW 264.7 cells (10 × 10^7^; the day prior to the assay; control vector, TBC1D10C, TBC1D10C-R141K, and Rab35 S22N) were cultured in round plates (9 cm) with RPMI medium (9 mL) supplemented with G418 and incubated. On the day of the assay, the medium was discarded, and a monolayer of cells was washed once (PBS; 5 mL). Cells were lysed by adding 1 mL of lysis buffer (150 mM NaCl, 1% Triton X-100, 50 mM Tris HCl, pH 8/protease inhibitors, 1.5x) and detached using a cell scraper. The supernatant was collected and incubated for 30 min on ice (2 mL microtubes), and proteins were obtained from that supernatant by centrifugation (10,000 r.p.m./10 min/4 °C). Immunoprecipitation was carried out using anti-GFP coupled to magnetic beads (MACS, 130–091–125) and microcolumns (MACS, 130–042–701) coupled to a magnetic separator (MACS, 130–042–303). From that procedure, we obtained an eluate or immunoprecipitated proteins (proteins in Laemmli buffer), which were later separated by SDS–PAGE**. In gel digestion**, bands were excised and cut into small pieces (2 mm) and washed with 1:1 (v/v) 50 mM NH_4_HCO_3_ (ammonium bicarbonate) and ACN (acetonitrile) to cover pieces of gel. Then, the proteins were reduced (10 mM DTT, dithiothreitol, in 50 mM NH_4_HCO_3_; 45 min, 56 °C) and later alkylated (50 mM IAA, iodoacetamide, in 50 mM NH_4_HCO_3_ for thirty minutes in a dark environment at room temperature, RT). Protein digestion was carried out by incubating (37 °C, overnight) pieces of the gel with trypsin (25 ng/µL in 50 mM NH_4_HCO_3_), and then peptide extraction was performed using incubation for 30 min (RT) with different concentrations of extraction solution, v/v (extraction solutions were as follows: extraction 1, 100 µL of formic acid 0.1% solution in Milli-Q water and 25 µL of ACN; extraction 2, 75 µL of formic acid 0.1% solution in Milli-Q water and 25 µL of ACN; extraction 3, 50 µL of formic acid 0.1% solution in Milli-Q water and 50 µL of ACN). Supernatants from extractions were mixed, dried (vacuum centrifuge; 65 °C/1200 r.p.m), and stored (-86 °C) until use. **Mass spectrometry:** Dried peptides were resuspended (20 µL of 1% formic acid in Milli-Q water; vortexed for 5 min) and injected (5 µL) in a nanoHPLC (fluid phase: A solution, TFA, trifluoroacetic acid; B solution, formic acid, 0.1%) coupled to a mass spectrometer (Amazon Speed Bruker or Impact II; electrospray ionization) to allow ionization and fragmentation of peptides (MS/MS). Finally, we used Protein Scape software to identify corresponding proteins to the obtained mass spectrum (m/z) in a search in the SwissProt database (organism: Mus musculus) and considered carbamidomethyl and oxidation modifications.

### Bioinformatics

The protein–protein interaction (PPI) network for TBC1D10C-interacting proteins was built using STRING (Search Tool for the Retrieval of Interacting Genes/Proteins; http://www.string-db.org). The PPI was generated with confidence score ≥ 0.4. Likewise, grouping of TBC1D10C-interacting proteins into biological processes was carried out using Gene Ontology (GO) annotation (http://www.geneontology.org), as reported previously^71^. Prediction of intrinsically disordered regions in TBC1D10C was carried out using the bioinformatics tools DISOPRED (http://bioinf.cs.ucl.ac.uk/psipred/) and IUPred2A-ANCHOR2 (https://iupred2a.elte.hu/). Prediction was accomplished by submitting the TBC1D10C protein sequence (FASTA format) as well as the Src protein sequence for comparison. Prediction of putative P-sites was performed with the phosphonet server (http://www.phosphonet.ca). For statistical analysis, we used GraphPad Prism 8.0.

### Statistical analysis

Assays were performed with at least 3 independent experiments unless otherwise indicated. We used the D’Agostino-Pearson omnibus normality test to analyze parametric and nonparametric distributions of data. The statistical analyses used were unpaired t-tests or Mann–Whitney tests (two-tailed; statistical significance P < 0.05) for two groups of data with parametric or nonparametric distributions, respectively. In the case of multiple groups, we used Kruskal–Wallis test and ordinary one-way ANOVA followed by Dunn's multiple comparisons test and Tukey's multiple comparisons test, respectively.

## Supplementary Information

Supplementary Information 1.

Supplementary Information 2.

Supplementary Information 3.

Supplementary Information 4.

Supplementary Video 1.

Supplementary Video 2.

## Abbreviations

BMDM: Bone marrow-derived macrophage
GAP: GTPase-activating protein
KO: Knockout
WT: Wild type
*Burkholderia cenocepacia*: *B. cenocepacia*
IDR: Intrinsically disordered region
PRR: Pattern recognition receptor
DN: Dominant negative
CA: Constitutively active
average: Avg
