# Supplementary material for: TBC1D10C is a cytoskeletal functional linker that modulates cell spreading and phagocytosis in macrophages
Source: Sci Rep. 2021 Oct 22;11:20946. doi: 10.1038/s41598-021-00450-z (PMC8536695; doi:10.1038/s41598-021-00450-z)
Supplement: Supplementary file 1 — Supplementary Information 1. [file 41598_2021_450_MOESM1_ESM.docx]

**Supplementary information**

**Supplementary Movies legend. Video-microscopy for BMDMs. Movie 1, BMDM WT; Movie 2, BMDM KO. BMDMs were time-lapsed for kymograph analysis. It can be noticed that filopodia in KO BMDMs retracts abruptly towards the cell body (black arrow heads).**

**
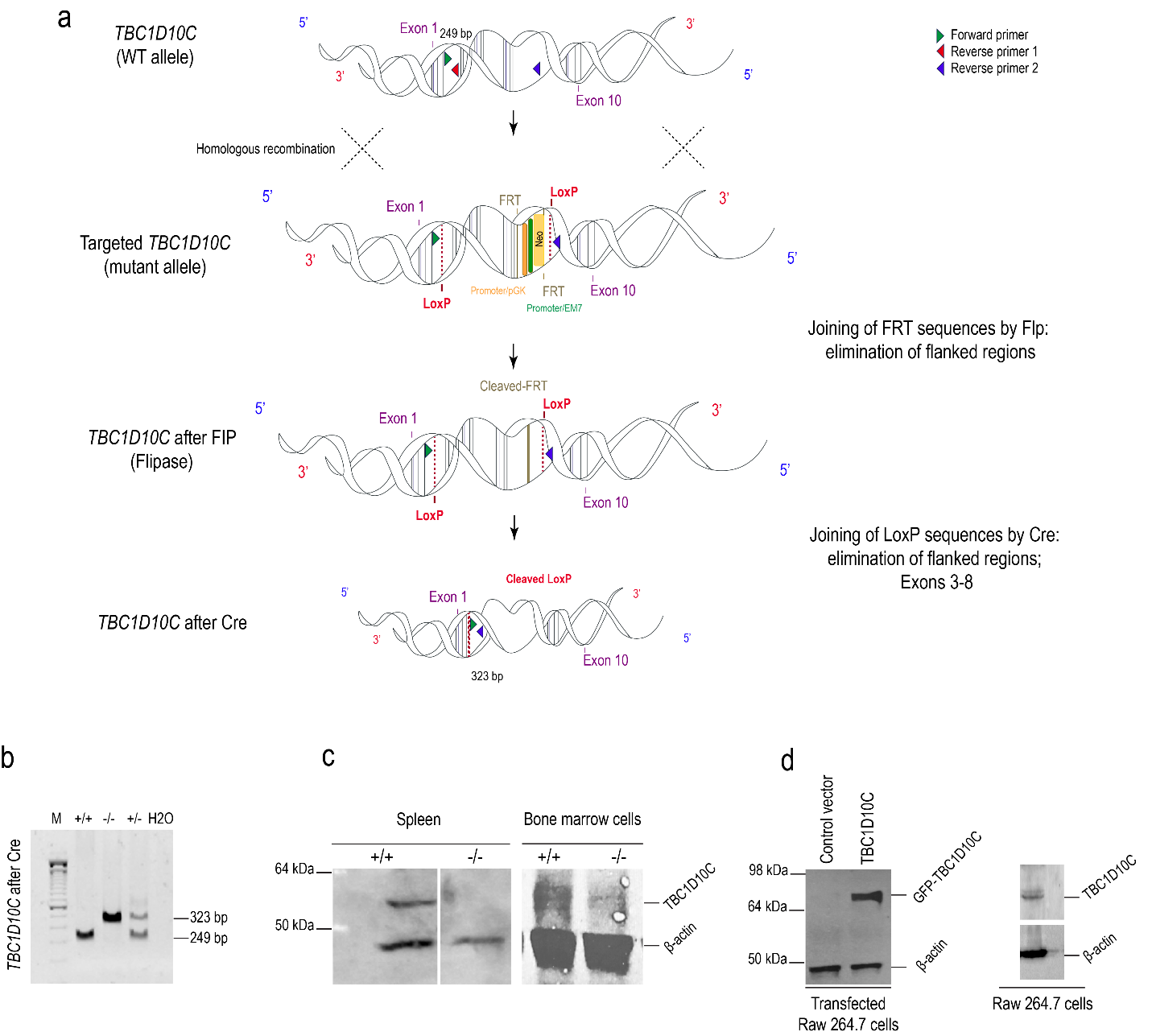
**

**Supplementary Figure 1.** Generation of TBC1D10C knockout mice by Cre-Lox recombination system. **a**, shows a schematic abstract for TBC1D10C knockout mice generation, starting at targeting the WT allele with mutant allele­­ by homologous recombination; elimination of FRT-flanked regions by mice crossing (flip mice); and elimination of lox-flanked regions (exons 3–8) by crossing with cre (β-actin) mice; **b**, mouse genotyping using specific primers after crossing flip and cre (β-actin) mice, expected bands are shown (Only shown WT, 249 bp; KO, 323 pb after crossing with Cre mice); **c**, western blot corroborating the elimination of TBC1D10C in different tissues from KO mice (left, spleen; right, bone marrow cells, right); **d**, western blot corroborating specifically the TBC1D10C (GFP tag) over-expression in Raw 264.7 transfected cells (left) as well as basal expression in same cells (right).

Suppl Fig 1 Continuation

Below are the original blots, some with different exposures, blue squares highlight the regions selected for display in the figure


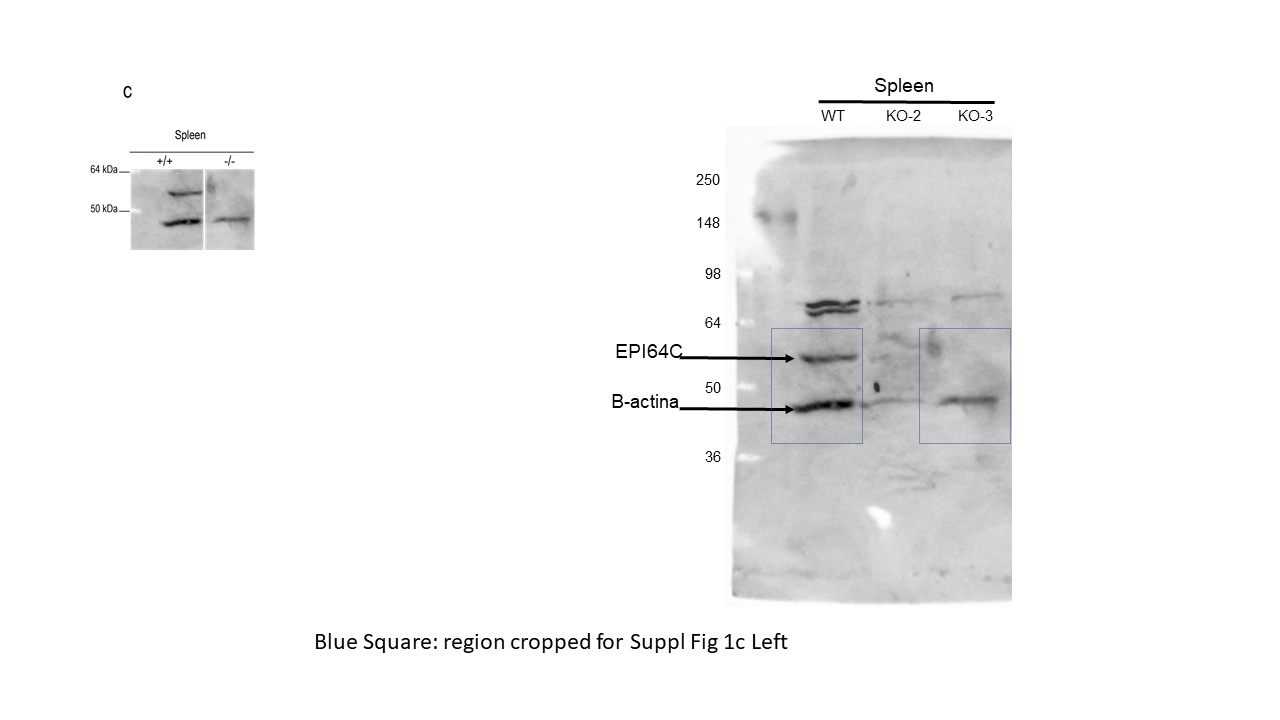

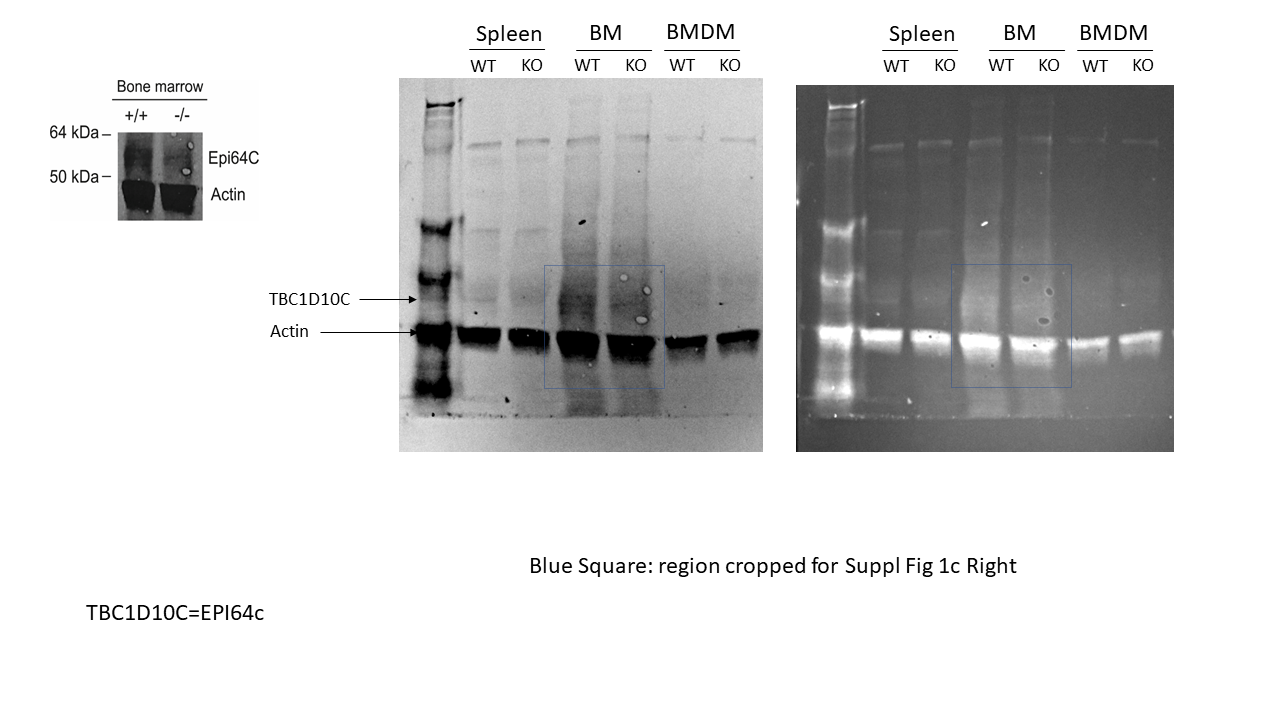


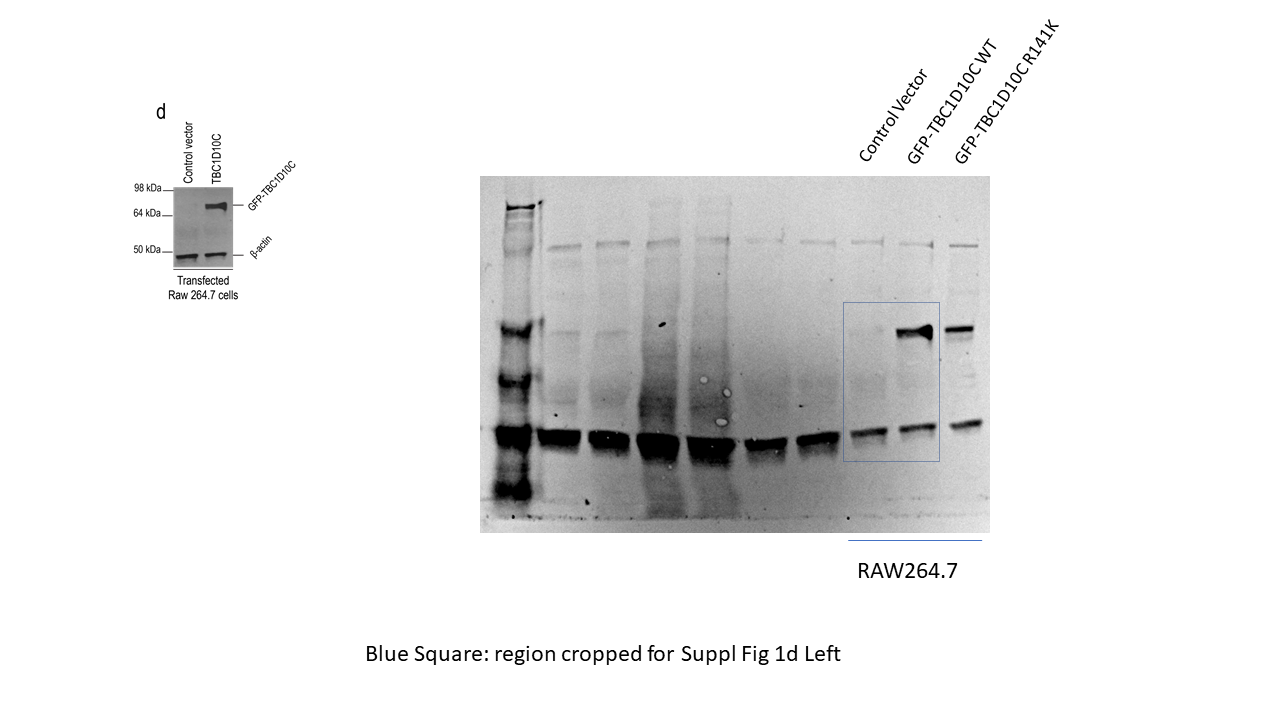


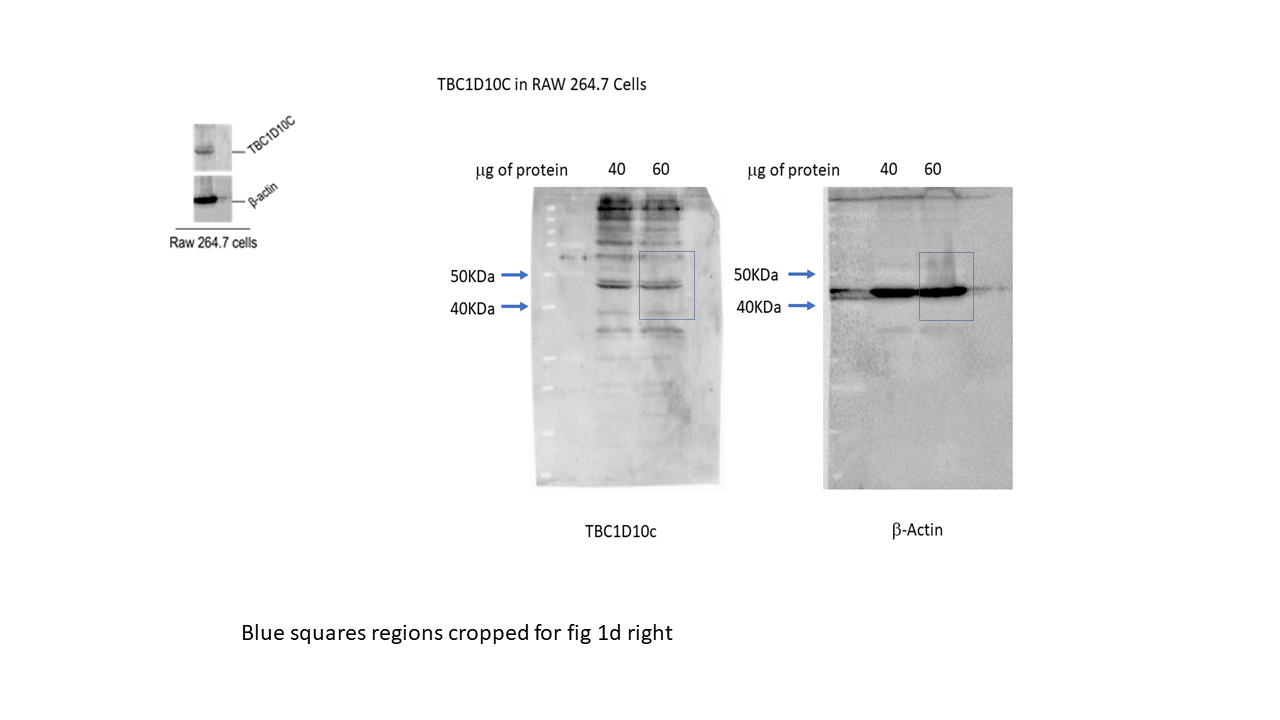


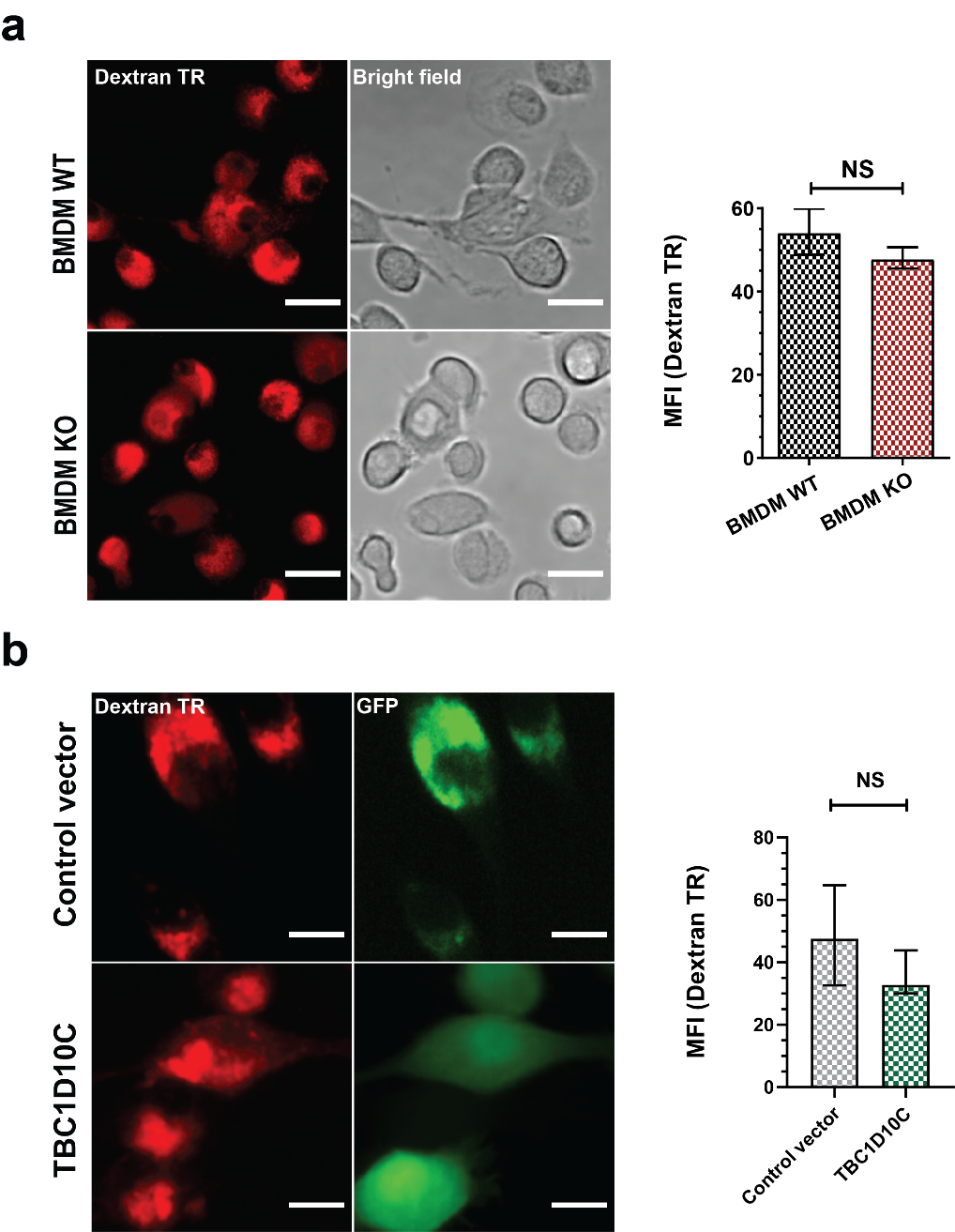


**Supplementary Figure 2**. Evaluation of TBC1D10C’s effect on macropinocytosis. Macropinocytosis was estimated by MFI (median fluorescence intensity) for dextran (TR, 10,000 Da). **a**, left, representative confocal images of BMDMs treated with dextran Texas red (TR, 10 kDa), and right, corresponding MFI of dextran TR (n = 3 mice; 151, WT and 157, KO). **b**, left, representative confocal images of Raw 264.7 macrophages treated with dextran Texas red, ), and right, corresponding MFI of dextran TR (58, control vector control and 62, TBC1D10C; 3 independent experiments). Data were analyzed by two-tailed Mann-Whitney test. Statistical significance p < 0.05 Graphs show median with 95% CI (confidence interval). Scale bar = 50 µm and 20 µm for BMDMs and Raw 264.7 cells, respectively.


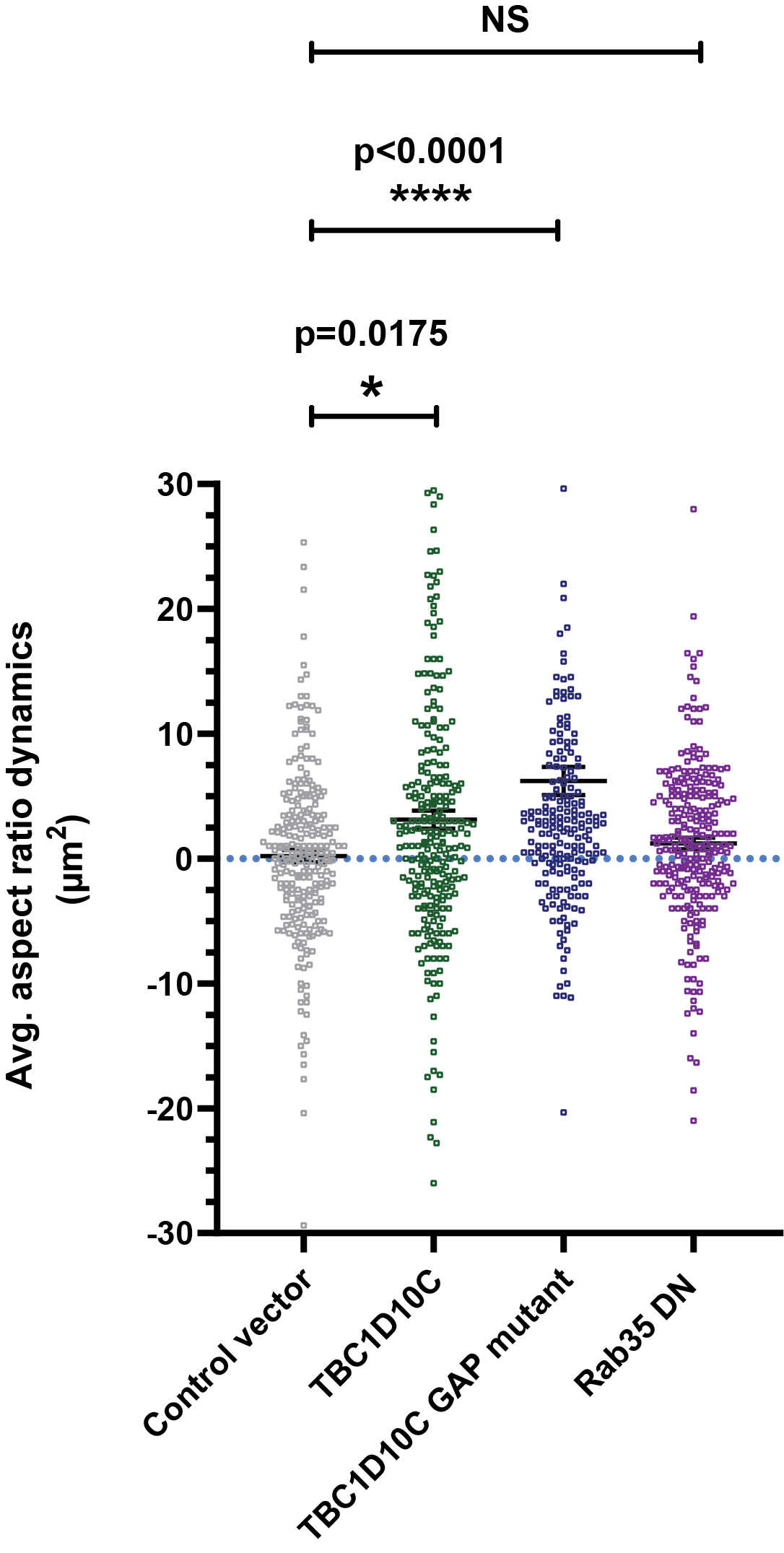


**Supplementary Figure 3.** Measurements for cell spreading dynamics. Cell spreading dynamics was calculated for each individual Raw 264.7 cell by defining ROIs (cell perimeter) at different time points; an average of cell area change was estimated. Raw 264.7 macrophages were time-lapsed by confocal microscopy (37°C; 40x, xyzt planes, acquisition ≈ 53 seconds for ≈ 111 min) (3 independent experiments; 28 Control vector, 29 TBC1D10C, 22 TBC1D10C-R141K cells, and 29 Rab35 DN). Data were analyzed by Krustal-Wallis test and Dunn's multiple comparisons test. Statistical significance p < 0.05. Scatter plot shows mean with SEM.


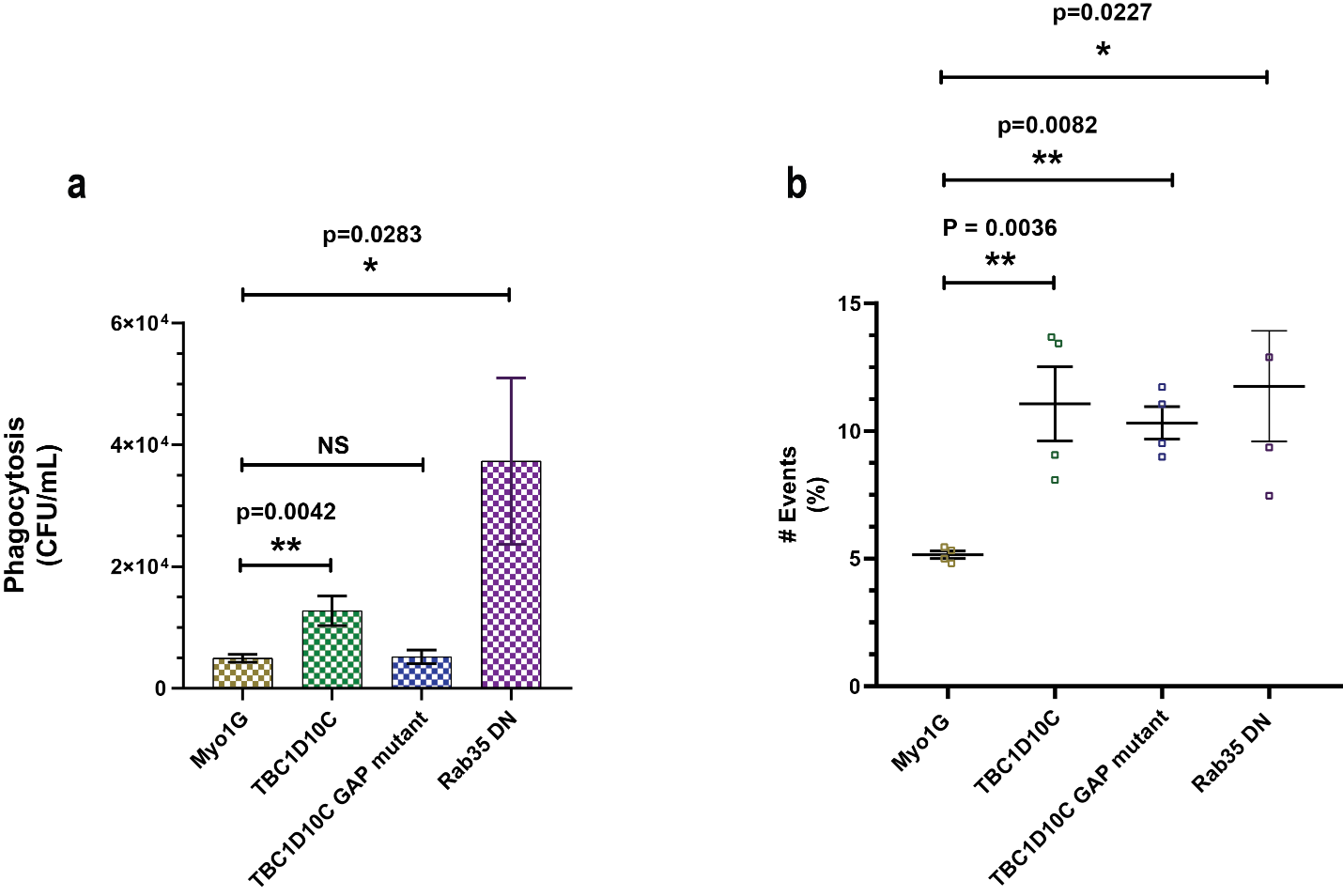


**Supplementary Figure 4.** Analysis of TBC1D10C’s GAP activity participation in phagocytosis of *B.* cenocepacia. Phagocytosis was analyzed by UFC and flow cytometry. **a**, quantification of CFU (phagocytosed living bacteria at 1.5 h post-infection) in infected Raw 264.7 transfected macrophages (as control we used Myo1G; unrelated protein to TBC1D10C; 5 independent experiments); **b**, data of quantified events expresses in percentage of Raw 264.7 transfected macrophages infected with transformed *B. cenocepacia* (MH1K-pDsRed) by flow cytometry (2 independent experiments). Data (Myo1G, TBC1D10C, and TBC1D10C GAP mutant) were analyzed by ordinary one-way ANOVA and Tukey's multiple comparisons test (data for Myo1G vs Rab35 DN were analyzed by two-tailed unpaired t test. Statistical significance p < 0.05., Graphs show mean with SEM.


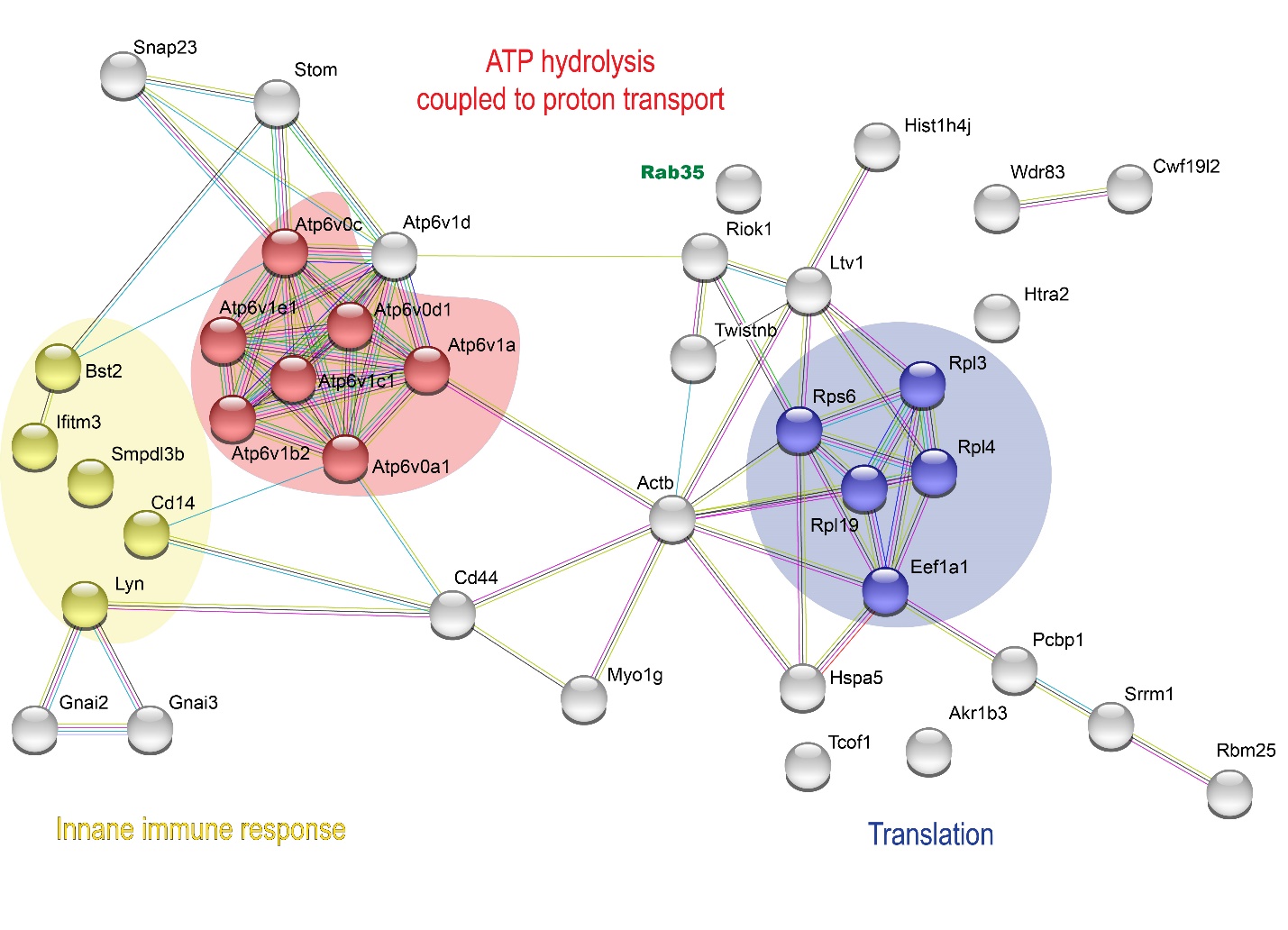


**Supplementary Figure 5.** The protein–protein interaction (PPI) network for Rab35-S22N-interacting proteins was built using STRING (Search Tool for the Retrieval of Interacting Genes/Proteins; http://www. string-db.org). The PPI was generated with confidence score ≥ 0.4. The network contains 39 nodes and 78 edges. Likewise, grouping of Rab35-S22N -interacting proteins into biological processes was carried out using Gene Ontology (GO) annotation (http:// [www.geneontology.org](http://www.geneontology.org)).

**Supplementary Movies.** Video-microscopy for BMDMs. Movie 1, BMDM WT; Movie 2, BMDM KO. BMDMs were time-lapse for kymograph analysis. It can be notice that filopodia retract abruptly towards the cell body (black arrow heads).

**Supplementary Tables**

| number of nodes: 28  number of edges: 90  average node degree: 6.43  avg. local clustering coefficient: 0.729 |
| --- |

**Network Stats**

| expected number of edges: 32  PPI enrichment p-value: < 1.0e-16 |
| --- |

**Supplementary Table 1.** Functional enrichments in TBC1D10C-interacting protein network

**Biological Process (GO)**

| *GO-term* | *description* | *count in gene set* | *false discovery rate* |
| --- | --- | --- | --- |
| [GO:0022613](http://amigo.geneontology.org/amigo/term/GO:0022613) | ribonucleoprotein complex biogenesis | 11 of 380 | 9.07e-10 |
| [GO:0042254](http://amigo.geneontology.org/amigo/term/GO:0042254) | ribosome biogenesis | 9 of 252 | 1.32e-08 |
| [GO:0044085](http://amigo.geneontology.org/amigo/term/GO:0044085) | cellular component biogenesis | 17 of 2213 | 3.19e-08 |
| [GO:0071840](http://amigo.geneontology.org/amigo/term/GO:0071840) | cellular component organization or biogenesis | 21 of 4730 | 6.67e-07 |
| [GO:0006412](http://amigo.geneontology.org/amigo/term/GO:0006412) | translation | 8 of 313 | 1.05e-06 |
| [GO:0006518](http://amigo.geneontology.org/amigo/term/GO:0006518) | peptide metabolic process | 8 of 440 | 8.70e-06 |
| [GO:0022618](http://amigo.geneontology.org/amigo/term/GO:0022618) | ribonucleoprotein complex assembly | 6 of 192 | 2.03e-05 |
| [GO:0034641](http://amigo.geneontology.org/amigo/term/GO:0034641) | cellular nitrogen compound metabolic process | 18 of 4247 | 2.59e-05 |
| [GO:1901566](http://amigo.geneontology.org/amigo/term/GO:1901566) | organonitrogen compound biosynthetic process | 10 of 1122 | 6.70e-05 |
| [GO:0042255](http://amigo.geneontology.org/amigo/term/GO:0042255) | ribosome assembly | 4 of 59 | 9.90e-05 |
| [GO:0034622](http://amigo.geneontology.org/amigo/term/GO:0034622) | cellular protein-containing complex assembly | 8 of 687 | 0.00013 |
| [GO:0042273](http://amigo.geneontology.org/amigo/term/GO:0042273) | ribosomal large subunit biogenesis | 4 of 68 | 0.00015 |
| [GO:0006996](http://amigo.geneontology.org/amigo/term/GO:0006996) | organelle organization | 14 of 2805 | 0.00015 |
| [GO:0022607](http://amigo.geneontology.org/amigo/term/GO:0022607) | cellular component assembly | 12 of 2015 | 0.00017 |
| [GO:0009987](http://amigo.geneontology.org/amigo/term/GO:0009987) | cellular process | 27 of 12459 | 0.00017 |
| [GO:0006364](http://amigo.geneontology.org/amigo/term/GO:0006364) | rRNA processing | 5 of 176 | 0.00017 |
| [GO:0016043](http://amigo.geneontology.org/amigo/term/GO:0016043) | cellular component organization | 17 of 4560 | 0.00026 |
| [GO:0015991](http://amigo.geneontology.org/amigo/term/GO:0015991) | ATP hydrolysis coupled proton transport | 3 of 25 | 0.00030 |
| [GO:0000154](http://amigo.geneontology.org/amigo/term/GO:0000154) | rRNA modification | 3 of 29 | 0.00042 |
| [GO:0000027](http://amigo.geneontology.org/amigo/term/GO:0000027) | ribosomal large subunit assembly | 3 of 29 | 0.00042 |
| [GO:0070887](http://amigo.geneontology.org/amigo/term/GO:0070887) | cellular response to chemical stimulus | 12 of 2287 | 0.00043 |
| [GO:1901564](http://amigo.geneontology.org/amigo/term/GO:1901564) | organonitrogen compound metabolic process | 16 of 4480 | 0.00088 |
| [GO:0065003](http://amigo.geneontology.org/amigo/term/GO:0065003) | protein-containing complex assembly | 9 of 1292 | 0.00088 |
| [GO:0048254](http://amigo.geneontology.org/amigo/term/GO:0048254) | snoRNA localization | 2 of 4 | 0.00091 |
| [GO:0044267](http://amigo.geneontology.org/amigo/term/GO:0044267) | cellular protein metabolic process | 13 of 2995 | 0.0010 |
| [GO:0042221](http://amigo.geneontology.org/amigo/term/GO:0042221) | response to chemical | 14 of 3532 | 0.0011 |
| [GO:0010467](http://amigo.geneontology.org/amigo/term/GO:0010467) | gene expression | 13 of 3013 | 0.0011 |
| [GO:1990090](http://amigo.geneontology.org/amigo/term/GO:1990090) | cellular response to nerve growth factor stimulus | 3 of 47 | 0.0012 |
| [GO:0046034](http://amigo.geneontology.org/amigo/term/GO:0046034) | ATP metabolic process | 4 of 162 | 0.0017 |
| [GO:0070925](http://amigo.geneontology.org/amigo/term/GO:0070925) | organelle assembly | 6 of 576 | 0.0021 |
| [GO:0044271](http://amigo.geneontology.org/amigo/term/GO:0044271) | cellular nitrogen compound biosynthetic process | 12 of 2781 | 0.0021 |
| [GO:0006807](http://amigo.geneontology.org/amigo/term/GO:0006807) | nitrogen compound metabolic process | 19 of 6983 | 0.0025 |
| [GO:0009167](http://amigo.geneontology.org/amigo/term/GO:0009167) | purine ribonucleoside monophosphate metabolic process | 4 of 196 | 0.0028 |
| [GO:0036295](http://amigo.geneontology.org/amigo/term/GO:0036295) | cellular response to increased oxygen levels | 2 of 12 | 0.0032 |
| [GO:0044237](http://amigo.geneontology.org/amigo/term/GO:0044237) | cellular metabolic process | 19 of 7348 | 0.0042 |
| [GO:0044238](http://amigo.geneontology.org/amigo/term/GO:0044238) | primary metabolic process | 19 of 7426 | 0.0048 |
| [GO:0006396](http://amigo.geneontology.org/amigo/term/GO:0006396) | RNA processing | 6 of 715 | 0.0049 |
| [GO:0006139](http://amigo.geneontology.org/amigo/term/GO:0006139) | nucleobase-containing compound metabolic process | 13 of 3702 | 0.0051 |
| [GO:0071310](http://amigo.geneontology.org/amigo/term/GO:0071310) | cellular response to organic substance | 9 of 1858 | 0.0067 |
| [GO:0017144](http://amigo.geneontology.org/amigo/term/GO:0017144) | drug metabolic process | 5 of 494 | 0.0071 |
| [GO:0071704](http://amigo.geneontology.org/amigo/term/GO:0071704) | organic substance metabolic process | 19 of 7733 | 0.0078 |
| [GO:0060359](http://amigo.geneontology.org/amigo/term/GO:0060359) | response to ammonium ion | 3 of 122 | 0.0092 |
| [GO:0006417](http://amigo.geneontology.org/amigo/term/GO:0006417) | regulation of translation | 4 of 307 | 0.0105 |
| [GO:0071353](http://amigo.geneontology.org/amigo/term/GO:0071353) | cellular response to interleukin-4 | 2 of 27 | 0.0106 |
| [GO:0006414](http://amigo.geneontology.org/amigo/term/GO:0006414) | translational elongation | 2 of 27 | 0.0106 |
| [GO:0010033](http://amigo.geneontology.org/amigo/term/GO:0010033) | response to organic substance | 10 of 2553 | 0.0125 |
| [GO:1901576](http://amigo.geneontology.org/amigo/term/GO:1901576) | organic substance biosynthetic process | 12 of 3651 | 0.0145 |
| [GO:0006757](http://amigo.geneontology.org/amigo/term/GO:0006757) | ATP generation from ADP | 2 of 37 | 0.0163 |
| [GO:0006096](http://amigo.geneontology.org/amigo/term/GO:0006096) | glycolytic process | 2 of 37 | 0.0163 |
| [GO:0043170](http://amigo.geneontology.org/amigo/term/GO:0043170) | macromolecule metabolic process | 16 of 6225 | 0.0177 |
| [GO:0042866](http://amigo.geneontology.org/amigo/term/GO:0042866) | pyruvate biosynthetic process | 2 of 41 | 0.0184 |
| [GO:0071495](http://amigo.geneontology.org/amigo/term/GO:0071495) | cellular response to endogenous stimulus | 6 of 997 | 0.0186 |
| [GO:0034645](http://amigo.geneontology.org/amigo/term/GO:0034645) | cellular macromolecule biosynthetic process | 10 of 2778 | 0.0201 |
| [GO:2000377](http://amigo.geneontology.org/amigo/term/GO:2000377) | regulation of reactive oxygen species metabolic process | 3 of 184 | 0.0215 |
| [GO:0051402](http://amigo.geneontology.org/amigo/term/GO:0051402) | neuron apoptotic process | 2 of 48 | 0.0228 |
| [GO:0009628](http://amigo.geneontology.org/amigo/term/GO:0009628) | response to abiotic stimulus | 6 of 1063 | 0.0236 |
| [GO:0071103](http://amigo.geneontology.org/amigo/term/GO:0071103) | DNA conformation change | 3 of 198 | 0.0251 |
| [GO:0071363](http://amigo.geneontology.org/amigo/term/GO:0071363) | cellular response to growth factor stimulus | 4 of 437 | 0.0266 |
| [GO:0044260](http://amigo.geneontology.org/amigo/term/GO:0044260) | cellular macromolecule metabolic process | 14 of 5233 | 0.0268 |
| [GO:0019359](http://amigo.geneontology.org/amigo/term/GO:0019359) | nicotinamide nucleotide biosynthetic process | 2 of 54 | 0.0268 |
| [GO:0032268](http://amigo.geneontology.org/amigo/term/GO:0032268) | regulation of cellular protein metabolic process | 9 of 2443 | 0.0278 |
| [GO:0006879](http://amigo.geneontology.org/amigo/term/GO:0006879) | cellular iron ion homeostasis | 2 of 56 | 0.0278 |
| [GO:0006090](http://amigo.geneontology.org/amigo/term/GO:0006090) | pyruvate metabolic process | 2 of 62 | 0.0315 |
| [GO:0042274](http://amigo.geneontology.org/amigo/term/GO:0042274) | ribosomal small subunit biogenesis | 2 of 70 | 0.0384 |
| [GO:0010941](http://amigo.geneontology.org/amigo/term/GO:0010941) | regulation of cell death | 7 of 1640 | 0.0384 |
| [GO:0006754](http://amigo.geneontology.org/amigo/term/GO:0006754) | ATP biosynthetic process | 2 of 70 | 0.0384 |
| [GO:0033365](http://amigo.geneontology.org/amigo/term/GO:0033365) | protein localization to organelle | 4 of 515 | 0.0393 |
| [GO:0051276](http://amigo.geneontology.org/amigo/term/GO:0051276) | chromosome organization | 5 of 883 | 0.0463 |
| [GO:0043066](http://amigo.geneontology.org/amigo/term/GO:0043066) | negative regulation of apoptotic process | 5 of 884 | 0.0463 |
| [GO:0071214](http://amigo.geneontology.org/amigo/term/GO:0071214) | cellular response to abiotic stimulus | 3 of 272 | 0.0465 |
| [GO:0006334](http://amigo.geneontology.org/amigo/term/GO:0006334) | nucleosome assembly | 2 of 80 | 0.0465 |
| [GO:0007010](http://amigo.geneontology.org/amigo/term/GO:0007010) | cytoskeleton organization | 5 of 916 | 0.0490 |
| [GO:0006986](http://amigo.geneontology.org/amigo/term/GO:0006986) | response to unfolded protein | 2 of 86 | 0.0490 |
| [GO:1903426](http://amigo.geneontology.org/amigo/term/GO:1903426) | regulation of reactive oxygen species biosynthetic process | 2 of 87 | 0.0494 |

**Molecular Function (GO)**

| *GO-term* | *description* | *count in gene set* | *false discovery rate* |
| --- | --- | --- | --- |
| [GO:1901363](http://amigo.geneontology.org/amigo/term/GO:1901363) | heterocyclic compound binding | 25 of 4748 | 9.26e-12 |
| [GO:0097159](http://amigo.geneontology.org/amigo/term/GO:0097159) | organic cyclic compound binding | 25 of 4818 | 9.26e-12 |
| [GO:0003723](http://amigo.geneontology.org/amigo/term/GO:0003723) | RNA binding | 13 of 986 | 5.18e-09 |
| [GO:0017111](http://amigo.geneontology.org/amigo/term/GO:0017111) | nucleoside-triphosphatase activity | 11 of 714 | 3.63e-08 |
| [GO:0005488](http://amigo.geneontology.org/amigo/term/GO:0005488) | binding | 28 of 10884 | 8.39e-08 |
| [GO:0003924](http://amigo.geneontology.org/amigo/term/GO:0003924) | GTPase activity | 7 of 255 | 8.23e-07 |
| [GO:0000166](http://amigo.geneontology.org/amigo/term/GO:0000166) | nucleotide binding | 14 of 2006 | 8.23e-07 |
| [GO:0035639](http://amigo.geneontology.org/amigo/term/GO:0035639) | purine ribonucleoside triphosphate binding | 13 of 1697 | 9.03e-07 |
| [GO:0032555](http://amigo.geneontology.org/amigo/term/GO:0032555) | purine ribonucleotide binding | 13 of 1766 | 1.33e-06 |
| [GO:0005525](http://amigo.geneontology.org/amigo/term/GO:0005525) | GTP binding | 7 of 338 | 3.02e-06 |
| [GO:0005198](http://amigo.geneontology.org/amigo/term/GO:0005198) | structural molecule activity | 8 of 546 | 3.31e-06 |
| [GO:0003676](http://amigo.geneontology.org/amigo/term/GO:0003676) | nucleic acid binding | 15 of 2868 | 3.74e-06 |
| [GO:0016787](http://amigo.geneontology.org/amigo/term/GO:0016787) | hydrolase activity | 13 of 2259 | 1.11e-05 |
| [GO:0005200](http://amigo.geneontology.org/amigo/term/GO:0005200) | structural constituent of cytoskeleton | 4 of 67 | 1.71e-05 |
| [GO:0019899](http://amigo.geneontology.org/amigo/term/GO:0019899) | enzyme binding | 12 of 2175 | 4.80e-05 |
| [GO:0003729](http://amigo.geneontology.org/amigo/term/GO:0003729) | mRNA binding | 5 of 202 | 4.80e-05 |
| [GO:0003824](http://amigo.geneontology.org/amigo/term/GO:0003824) | catalytic activity | 17 of 5239 | 0.00026 |
| [GO:0003735](http://amigo.geneontology.org/amigo/term/GO:0003735) | structural constituent of ribosome | 4 of 153 | 0.00034 |
| [GO:0044877](http://amigo.geneontology.org/amigo/term/GO:0044877) | protein-containing complex binding | 8 of 1094 | 0.00036 |
| [GO:0001094](http://amigo.geneontology.org/amigo/term/GO:0001094) | TFIID-class transcription factor complex binding | 2 of 9 | 0.00061 |
| [GO:0019843](http://amigo.geneontology.org/amigo/term/GO:0019843) | rRNA binding | 3 of 67 | 0.00066 |
| [GO:0016887](http://amigo.geneontology.org/amigo/term/GO:0016887) | ATPase activity | 5 of 372 | 0.00069 |
| [GO:0005515](http://amigo.geneontology.org/amigo/term/GO:0005515) | protein binding | 18 of 6454 | 0.00087 |
| [GO:0008097](http://amigo.geneontology.org/amigo/term/GO:0008097) | 5S rRNA binding | 2 of 13 | 0.0010 |
| [GO:0003746](http://amigo.geneontology.org/amigo/term/GO:0003746) | translation elongation factor activity | 2 of 15 | 0.0013 |
| [GO:0046961](http://amigo.geneontology.org/amigo/term/GO:0046961) | proton-transporting ATPase activity, rotational mechanism | 2 of 18 | 0.0018 |
| [GO:0045182](http://amigo.geneontology.org/amigo/term/GO:0045182) | translation regulator activity | 3 of 117 | 0.0027 |
| [GO:0043021](http://amigo.geneontology.org/amigo/term/GO:0043021) | ribonucleoprotein complex binding | 3 of 136 | 0.0039 |
| [GO:0030515](http://amigo.geneontology.org/amigo/term/GO:0030515) | snoRNA binding | 2 of 29 | 0.0039 |
| [GO:0008144](http://amigo.geneontology.org/amigo/term/GO:0008144) | drug binding | 8 of 1630 | 0.0039 |
| [GO:0097718](http://amigo.geneontology.org/amigo/term/GO:0097718) | disordered domain specific binding | 2 of 34 | 0.0049 |
| [GO:0019901](http://amigo.geneontology.org/amigo/term/GO:0019901) | protein kinase binding | 5 of 659 | 0.0068 |
| [GO:0005524](http://amigo.geneontology.org/amigo/term/GO:0005524) | ATP binding | 7 of 1389 | 0.0071 |
| [GO:0000049](http://amigo.geneontology.org/amigo/term/GO:0000049) | tRNA binding | 2 of 54 | 0.0100 |
| [GO:0043167](http://amigo.geneontology.org/amigo/term/GO:0043167) | ion binding | 14 of 5302 | 0.0109 |
| [GO:0043022](http://amigo.geneontology.org/amigo/term/GO:0043022) | ribosome binding | 2 of 57 | 0.0109 |
| [GO:0019904](http://amigo.geneontology.org/amigo/term/GO:0019904) | protein domain specific binding | 5 of 775 | 0.0112 |
| [GO:0051082](http://amigo.geneontology.org/amigo/term/GO:0051082) | unfolded protein binding | 2 of 77 | 0.0175 |
| [GO:0008092](http://amigo.geneontology.org/amigo/term/GO:0008092) | cytoskeletal protein binding | 5 of 877 | 0.0175 |
| [GO:0051117](http://amigo.geneontology.org/amigo/term/GO:0051117) | ATPase binding | 2 of 91 | 0.0230 |
| [GO:0042623](http://amigo.geneontology.org/amigo/term/GO:0042623) | ATPase activity, coupled | 3 of 301 | 0.0237 |
| [GO:0140098](http://amigo.geneontology.org/amigo/term/GO:0140098) | catalytic activity, acting on RNA | 3 of 317 | 0.0263 |
| [GO:0031625](http://amigo.geneontology.org/amigo/term/GO:0031625) | ubiquitin protein ligase binding | 3 of 316 | 0.0263 |
| [GO:0008134](http://amigo.geneontology.org/amigo/term/GO:0008134) | transcription factor binding | 4 of 644 | 0.0286 |
| [GO:0001085](http://amigo.geneontology.org/amigo/term/GO:0001085) | RNA polymerase II transcription factor binding | 2 of 130 | 0.0383 |

**Cellular Component (GO)**

| *GO-term* | *description* | *count in gene set* | *false discovery rate* |
| --- | --- | --- | --- |
| [GO:1990904](http://amigo.geneontology.org/amigo/term/GO:1990904) | ribonucleoprotein complex | 14 of 765 | 3.04e-11 |
| [GO:0032991](http://amigo.geneontology.org/amigo/term/GO:0032991) | protein-containing complex | 23 of 4701 | 1.73e-09 |
| [GO:0005829](http://amigo.geneontology.org/amigo/term/GO:0005829) | cytosol | 20 of 3326 | 3.40e-09 |
| [GO:0043209](http://amigo.geneontology.org/amigo/term/GO:0043209) | myelin sheath | 8 of 212 | 1.63e-08 |
| [GO:0043232](http://amigo.geneontology.org/amigo/term/GO:0043232) | intracellular non-membrane-bounded organelle | 19 of 3809 | 2.69e-07 |
| [GO:0044444](http://amigo.geneontology.org/amigo/term/GO:0044444) | cytoplasmic part | 24 of 7673 | 1.70e-06 |
| [GO:0044424](http://amigo.geneontology.org/amigo/term/GO:0044424) | intracellular part | 28 of 12219 | 2.45e-06 |
| [GO:0044428](http://amigo.geneontology.org/amigo/term/GO:0044428) | nuclear part | 16 of 3798 | 6.55e-05 |
| [GO:0098805](http://amigo.geneontology.org/amigo/term/GO:0098805) | whole membrane | 10 of 1300 | 6.76e-05 |
| [GO:0044422](http://amigo.geneontology.org/amigo/term/GO:0044422) | organelle part | 22 of 7665 | 6.76e-05 |
| [GO:0036464](http://amigo.geneontology.org/amigo/term/GO:0036464) | cytoplasmic ribonucleoprotein granule | 5 of 182 | 7.00e-05 |
| [GO:0033176](http://amigo.geneontology.org/amigo/term/GO:0033176) | proton-transporting V-type ATPase complex | 3 of 22 | 7.48e-05 |
| [GO:0005732](http://amigo.geneontology.org/amigo/term/GO:0005732) | small nucleolar ribonucleoprotein complex | 3 of 22 | 7.48e-05 |
| [GO:0043229](http://amigo.geneontology.org/amigo/term/GO:0043229) | intracellular organelle | 25 of 10645 | 9.71e-05 |
| [GO:0045121](http://amigo.geneontology.org/amigo/term/GO:0045121) | membrane raft | 6 of 374 | 9.91e-05 |
| [GO:0005840](http://amigo.geneontology.org/amigo/term/GO:0005840) | ribosome | 5 of 215 | 0.00010 |
| [GO:0044446](http://amigo.geneontology.org/amigo/term/GO:0044446) | intracellular organelle part | 21 of 7416 | 0.00011 |
| [GO:0044445](http://amigo.geneontology.org/amigo/term/GO:0044445) | cytosolic part | 5 of 228 | 0.00011 |
| [GO:0022626](http://amigo.geneontology.org/amigo/term/GO:0022626) | cytosolic ribosome | 4 of 107 | 0.00011 |
| [GO:0005730](http://amigo.geneontology.org/amigo/term/GO:0005730) | nucleolus | 8 of 878 | 0.00011 |
| [GO:0005634](http://amigo.geneontology.org/amigo/term/GO:0005634) | nucleus | 19 of 6086 | 0.00011 |
| [GO:0070013](http://amigo.geneontology.org/amigo/term/GO:0070013) | intracellular organelle lumen | 15 of 3882 | 0.00017 |
| [GO:0120025](http://amigo.geneontology.org/amigo/term/GO:0120025) | plasma membrane bounded cell projection | 11 of 2172 | 0.00031 |
| [GO:0015030](http://amigo.geneontology.org/amigo/term/GO:0015030) | Cajal body | 3 of 51 | 0.00035 |
| [GO:0043227](http://amigo.geneontology.org/amigo/term/GO:0043227) | membrane-bounded organelle | 23 of 9775 | 0.00037 |
| [GO:0098590](http://amigo.geneontology.org/amigo/term/GO:0098590) | plasma membrane region | 8 of 1115 | 0.00041 |
| [GO:0033180](http://amigo.geneontology.org/amigo/term/GO:0033180) | proton-transporting V-type ATPase, V1 domain | 2 of 7 | 0.00041 |
| [GO:0043231](http://amigo.geneontology.org/amigo/term/GO:0043231) | intracellular membrane-bounded organelle | 22 of 9088 | 0.00046 |
| [GO:0022625](http://amigo.geneontology.org/amigo/term/GO:0022625) | cytosolic large ribosomal subunit | 3 of 59 | 0.00046 |
| [GO:0031428](http://amigo.geneontology.org/amigo/term/GO:0031428) | box C/D snoRNP complex | 2 of 8 | 0.00047 |
| [GO:0044391](http://amigo.geneontology.org/amigo/term/GO:0044391) | ribosomal subunit | 4 of 181 | 0.00055 |
| [GO:0031981](http://amigo.geneontology.org/amigo/term/GO:0031981) | nuclear lumen | 13 of 3386 | 0.00065 |
| [GO:0005844](http://amigo.geneontology.org/amigo/term/GO:0005844) | polysome | 3 of 71 | 0.00070 |
| [GO:0030684](http://amigo.geneontology.org/amigo/term/GO:0030684) | preribosome | 3 of 72 | 0.00072 |
| [GO:0005886](http://amigo.geneontology.org/amigo/term/GO:0005886) | plasma membrane | 14 of 4328 | 0.0018 |
| [GO:0042470](http://amigo.geneontology.org/amigo/term/GO:0042470) | melanosome | 3 of 103 | 0.0019 |
| [GO:0120038](http://amigo.geneontology.org/amigo/term/GO:0120038) | plasma membrane bounded cell projection part | 8 of 1532 | 0.0026 |
| [GO:0031253](http://amigo.geneontology.org/amigo/term/GO:0031253) | cell projection membrane | 4 of 316 | 0.0035 |
| [GO:0044448](http://amigo.geneontology.org/amigo/term/GO:0044448) | cell cortex part | 3 of 136 | 0.0036 |
| [GO:0042788](http://amigo.geneontology.org/amigo/term/GO:0042788) | polysomal ribosome | 2 of 30 | 0.0038 |
| [GO:0032040](http://amigo.geneontology.org/amigo/term/GO:0032040) | small-subunit processome | 2 of 33 | 0.0043 |
| [GO:0016324](http://amigo.geneontology.org/amigo/term/GO:0016324) | apical plasma membrane | 4 of 339 | 0.0043 |
| [GO:0001726](http://amigo.geneontology.org/amigo/term/GO:0001726) | ruffle | 3 of 168 | 0.0062 |
| [GO:0044452](http://amigo.geneontology.org/amigo/term/GO:0044452) | nucleolar part | 3 of 175 | 0.0068 |
| [GO:0044451](http://amigo.geneontology.org/amigo/term/GO:0044451) | nucleoplasm part | 6 of 1023 | 0.0072 |
| [GO:0016604](http://amigo.geneontology.org/amigo/term/GO:0016604) | nuclear body | 5 of 709 | 0.0084 |
| [GO:0044459](http://amigo.geneontology.org/amigo/term/GO:0044459) | plasma membrane part | 9 of 2362 | 0.0086 |
| [GO:0044425](http://amigo.geneontology.org/amigo/term/GO:0044425) | membrane part | 15 of 5857 | 0.0095 |
| [GO:0005856](http://amigo.geneontology.org/amigo/term/GO:0005856) | cytoskeleton | 8 of 1933 | 0.0095 |
| [GO:0098858](http://amigo.geneontology.org/amigo/term/GO:0098858) | actin-based cell projection | 3 of 216 | 0.0110 |
| [GO:0016020](http://amigo.geneontology.org/amigo/term/GO:0016020) | membrane | 17 of 7460 | 0.0135 |
| [GO:0000786](http://amigo.geneontology.org/amigo/term/GO:0000786) | nucleosome | 2 of 67 | 0.0141 |
| [GO:0005654](http://amigo.geneontology.org/amigo/term/GO:0005654) | nucleoplasm | 9 of 2648 | 0.0168 |
| [GO:1902494](http://amigo.geneontology.org/amigo/term/GO:1902494) | catalytic complex | 6 of 1266 | 0.0176 |
| [GO:0097458](http://amigo.geneontology.org/amigo/term/GO:0097458) | neuron part | 7 of 1732 | 0.0192 |
| [GO:0032587](http://amigo.geneontology.org/amigo/term/GO:0032587) | ruffle membrane | 2 of 85 | 0.0204 |
| [GO:0030863](http://amigo.geneontology.org/amigo/term/GO:0030863) | cortical cytoskeleton | 2 of 85 | 0.0204 |
| [GO:0005902](http://amigo.geneontology.org/amigo/term/GO:0005902) | microvillus | 2 of 95 | 0.0246 |
| [GO:0043005](http://amigo.geneontology.org/amigo/term/GO:0043005) | neuron projection | 6 of 1429 | 0.0296 |
| [GO:0044430](http://amigo.geneontology.org/amigo/term/GO:0044430) | cytoskeletal part | 6 of 1460 | 0.0323 |
| [GO:0150034](http://amigo.geneontology.org/amigo/term/GO:0150034) | distal axon | 3 of 378 | 0.0416 |
| [GO:0030424](http://amigo.geneontology.org/amigo/term/GO:0030424) | axon | 4 of 712 | 0.0416 |
| [GO:0015630](http://amigo.geneontology.org/amigo/term/GO:0015630) | microtubule cytoskeleton | 5 of 1106 | 0.0416 |
| [GO:0012505](http://amigo.geneontology.org/amigo/term/GO:0012505) | endomembrane system | 10 of 3670 | 0.0416 |
| [GO:0001650](http://amigo.geneontology.org/amigo/term/GO:0001650) | fibrillar center | 2 of 128 | 0.0416 |
| [GO:0005903](http://amigo.geneontology.org/amigo/term/GO:0005903) | brush border | 2 of 133 | 0.0422 |

**Reference publications**

| *publication* | *(year) title* | *count in gene set* | *false discovery rate* |
| --- | --- | --- | --- |
| [PMID:28736500](https://www.ncbi.nlm.nih.gov/pubmed/28736500) | (2017) Comparative Proteomic Analysis of Three Chinese Hamster Ovary (CHO) Host Cells. | 7 of 50 | 1.19e-07 |
| [PMID:23696868](https://www.ncbi.nlm.nih.gov/pubmed/23696868) | (2013) Enhanced translation of mRNAs encoding proteins involved in mRNA translation during recovery from heat shock. | 6 of 28 | 2.96e-07 |
| [PMID:16822315](https://www.ncbi.nlm.nih.gov/pubmed/16822315) | (2006) Gene identification and analysis of transcripts differentially regulated in fracture healing by EST sequencing in the domestic sheep. | 7 of 64 | 2.96e-07 |
| [PMID:29986939](https://www.ncbi.nlm.nih.gov/pubmed/29986939) | (2018) MSC exosome works through a protein-based mechanism of action. | 6 of 33 | 3.74e-07 |
| [PMID:26399832](https://www.ncbi.nlm.nih.gov/pubmed/26399832) | (2015) Cell-fate determination by ubiquitin-dependent regulation of translation. | 6 of 44 | 1.44e-06 |
| [PMID:25993305](https://www.ncbi.nlm.nih.gov/pubmed/25993305) | (2015) MALDI-Mass Spectrometric Imaging Revealing Hypoxia-Driven Lipids and Proteins in a Breast Tumor Model. | 6 of 44 | 1.44e-06 |
| [PMID:31151297](https://www.ncbi.nlm.nih.gov/pubmed/31151297) | (2019) Identification of Proteins Differentially Expressed by Adipose-derived Mesenchymal Stem Cells Isolated from Immunodeficient Mice. | 8 of 180 | 1.84e-06 |
| [PMID:31033440](https://www.ncbi.nlm.nih.gov/pubmed/31033440) | (2019) HRI coordinates translation necessary for protein homeostasis and mitochondrial function in erythropoiesis. | 6 of 53 | 2.36e-06 |
| [PMID:30260431](https://www.ncbi.nlm.nih.gov/pubmed/30260431) | (2018) Protein Syndesmos is a novel RNA-binding protein that regulates primary cilia formation. | 6 of 54 | 2.36e-06 |
| [PMID:20701774](https://www.ncbi.nlm.nih.gov/pubmed/20701774) | (2010) A novel function for vimentin: the potential biomarker for predicting melanoma hematogenous metastasis. | 5 of 19 | 2.36e-06 |
| [PMID:24809507](https://www.ncbi.nlm.nih.gov/pubmed/24809507) | (2014) Multi-faceted proteomic characterization of host protein complement of Rift Valley fever virus virions and identification of specific heat shock proteins, including HSP90, as important viral host factors. | 6 of 59 | 3.36e-06 |
| [PMID:31698554](https://www.ncbi.nlm.nih.gov/pubmed/31698554) | (2019) LGR5 and Downstream Intracellular Signaling Proteins Play Critical Roles in the Cell Proliferation of Neuroblastoma, Meningioma and Pituitary Adenoma. | 5 of 24 | 4.38e-06 |
| [PMID:31460127](https://www.ncbi.nlm.nih.gov/pubmed/31460127) | (2019) Targeting Translation Activity at the Ribosome Interface with UV-Active Small Molecules. | 5 of 24 | 4.38e-06 |
| [PMID:26544960](https://www.ncbi.nlm.nih.gov/pubmed/26544960) | (2015) Lysine Methylation of the Valosin-Containing Protein (VCP) Is Dispensable for Development and Survival of Mice. | 5 of 24 | 4.38e-06 |
| [PMID:24113185](https://www.ncbi.nlm.nih.gov/pubmed/24113185) | (2013) Translational control in the stress adaptive response of cancer cells: a novel role for the heat shock protein TRAP1. | 5 of 24 | 4.38e-06 |
| [PMID:28611246](https://www.ncbi.nlm.nih.gov/pubmed/28611246) | (2017) Inhibition of Avian Influenza A Virus Replication in Human Cells by Host Restriction Factor TUFM Is Correlated with Autophagy. | 5 of 27 | 5.56e-06 |
| [PMID:23690912](https://www.ncbi.nlm.nih.gov/pubmed/23690912) | (2013) New model of action for mood stabilizers: phosphoproteome from rat pre-frontal cortex synaptoneurosomal preparations. | 5 of 27 | 5.56e-06 |
| [PMID:30891164](https://www.ncbi.nlm.nih.gov/pubmed/30891164) | (2019) Extracellular vesicles derived from natural killer cells use multiple cytotoxic proteins and killing mechanisms to target cancer cells. | 5 of 28 | 5.82e-06 |
| [PMID:30295850](https://www.ncbi.nlm.nih.gov/pubmed/30295850) | (2018) RNA-binding protein DDX1 is responsible for fatty acid-mediated repression of insulin translation. | 5 of 28 | 5.82e-06 |
| [PMID:23555724](https://www.ncbi.nlm.nih.gov/pubmed/23555724) | (2013) Oxidative stress induces monocyte necrosis with enrichment of cell-bound albumin and overexpression of endoplasmic reticulum and mitochondrial chaperones. | 5 of 29 | 5.82e-06 |
| [PMID:22809209](https://www.ncbi.nlm.nih.gov/pubmed/22809209) | (2012) Insights into the virulence of oral biofilms: discoveries from proteomics. | 5 of 28 | 5.82e-06 |
| [PMID:20529248](https://www.ncbi.nlm.nih.gov/pubmed/20529248) | (2010) Proteomic analysis of primary duck hepatocytes infected with duck hepatitis B virus. | 5 of 28 | 5.82e-06 |
| [PMID:28747643](https://www.ncbi.nlm.nih.gov/pubmed/28747643) | (2017) Generation of ribosome imprinted polymers for sensitive detection of translational responses. | 4 of 7 | 5.93e-06 |
| [PMID:22483619](https://www.ncbi.nlm.nih.gov/pubmed/22483619) | (2012) mRNA decapping factors and the exonuclease Xrn2 function in widespread premature termination of RNA polymerase II transcription. | 5 of 30 | 5.96e-06 |
| [PMID:26340096](https://www.ncbi.nlm.nih.gov/pubmed/26340096) | (2015) Ribosomal Biogenesis and Translational Flux Inhibition by the Selective Inhibitor of Nuclear Export (SINE) XPO1 Antagonist KPT-185. | 5 of 34 | 1.01e-05 |
| [PMID:26630129](https://www.ncbi.nlm.nih.gov/pubmed/26630129) | (2015) Transcriptional Profiling Identifies Location-Specific and Breed-Specific Differentially Expressed Genes in Embryonic Myogenesis in Anas Platyrhynchos. | 5 of 35 | 1.11e-05 |
| [PMID:27512079](https://www.ncbi.nlm.nih.gov/pubmed/27512079) | (2016) Quantitative Non-canonical Amino Acid Tagging (QuaNCAT) Proteomics Identifies Distinct Patterns of Protein Synthesis Rapidly Induced by Hypertrophic Agents in Cardiomyocytes, Revealing New Aspects of Metabolic Remodeling. | 5 of 37 | 1.38e-05 |
| [PMID:30862090](https://www.ncbi.nlm.nih.gov/pubmed/30862090) | (2019) Emerging Role of Eukaryote Ribosomes in Translational Control. | 5 of 38 | 1.47e-05 |
| [PMID:22537006](https://www.ncbi.nlm.nih.gov/pubmed/22537006) | (2012) PIntron: a fast method for detecting the gene structure due to alternative splicing via maximal pairings of a pattern and a text. | 4 of 10 | 1.47e-05 |
| [PMID:30302013](https://www.ncbi.nlm.nih.gov/pubmed/30302013) | (2018) Porcine Reproductive and Respiratory Syndrome Virus strains with Higher Virulence Cause Marked Protein Profile Changes in MARC-145 Cells. | 7 of 179 | 1.73e-05 |
| [PMID:24278208](https://www.ncbi.nlm.nih.gov/pubmed/24278208) | (2013) Differential phosphoproteome regulation of nucleus accumbens in environmentally enriched and isolated rats in response to acute stress. | 5 of 40 | 1.73e-05 |
| [PMID:20140087](https://www.ncbi.nlm.nih.gov/pubmed/20140087) | (2010) Comprehensive identification and modified-site mapping of S-nitrosylated targets in prostate epithelial cells. | 6 of 94 | 1.73e-05 |
| [PMID:28884116](https://www.ncbi.nlm.nih.gov/pubmed/28884116) | (2017) The Role of Pontin and Reptin in Cellular Physiology and Cancer Etiology. | 6 of 99 | 2.03e-05 |
| [PMID:23861804](https://www.ncbi.nlm.nih.gov/pubmed/23861804) | (2013) Proteomic profiling of rabbit embryonic stem cells derived from parthenotes and fertilized embryos. | 5 of 42 | 2.03e-05 |
| [PMID:19811410](https://www.ncbi.nlm.nih.gov/pubmed/19811410) | (2009) Proteomic analyses of the effects of drugs of abuse on monocyte-derived mature dendritic cells. | 5 of 42 | 2.03e-05 |
| [PMID:26939752](https://www.ncbi.nlm.nih.gov/pubmed/26939752) | (2016) Long noncoding RNAs (lncRNAs) dynamics evidence immunomodulation during ISAV-Infected Atlantic salmon (Salmo salar). | 4 of 12 | 2.07e-05 |
| [PMID:25307283](https://www.ncbi.nlm.nih.gov/pubmed/25307283) | (2015) The effect of sulforaphane on histone deacetylase activity in keratinocytes: Differences between in vitro and in vivo analyses. | 4 of 12 | 2.07e-05 |
| [PMID:20000738](https://www.ncbi.nlm.nih.gov/pubmed/20000738) | (2010) Proteomic dissection of cell type-specific H2AX-interacting protein complex associated with hepatocellular carcinoma. | 5 of 44 | 2.07e-05 |
| [PMID:19427311](https://www.ncbi.nlm.nih.gov/pubmed/19427311) | (2009) Molecular mechanisms of pancreatic dysfunction induced by protein malnutrition. | 4 of 12 | 2.07e-05 |
| [PMID:18302751](https://www.ncbi.nlm.nih.gov/pubmed/18302751) | (2008) Characterization of human mesenchymal stem cell secretome at early steps of adipocyte and osteoblast differentiation. | 5 of 43 | 2.07e-05 |
| [PMID:27152104](https://www.ncbi.nlm.nih.gov/pubmed/27152104) | (2016) Clinical proteomics of enervated neurons. | 5 of 45 | 2.25e-05 |
| [PMID:30852271](https://www.ncbi.nlm.nih.gov/pubmed/30852271) | (2019) N-Linked glycosylation of the membrane protein ectodomain regulates infectious bronchitis virus-induced ER stress response, apoptosis and pathogenesis. | 4 of 13 | 2.33e-05 |
| [PMID:29850612](https://www.ncbi.nlm.nih.gov/pubmed/29850612) | (2018) Proteomic Analysis of Hippocampus and Cortex in Streptozotocin-Induced Diabetic Model Mice Showing Dementia. | 5 of 46 | 2.33e-05 |
| [PMID:29337352](https://www.ncbi.nlm.nih.gov/pubmed/29337352) | (2018) Genetic removal of eIF2Alfa kinase PERK in mice enables hippocampal L-LTP independent of mTORC1 activity. | 4 of 13 | 2.33e-05 |
| [PMID:18596936](https://www.ncbi.nlm.nih.gov/pubmed/18596936) | (2008) Sex-specific expression of the X-linked histone demethylase gene Jarid1c in brain. | 4 of 13 | 2.33e-05 |
| [PMID:25923296](https://www.ncbi.nlm.nih.gov/pubmed/25923296) | (2015) Use of both cumulus cells' transcriptomic markers and zona pellucida birefringence to select developmentally competent oocytes in human assisted reproductive technologies. | 5 of 47 | 2.45e-05 |
| [PMID:25407680](https://www.ncbi.nlm.nih.gov/pubmed/25407680) | (2014) The dyskerin ribonucleoprotein complex as an OCT4SOX2 coactivator in embryonic stem cells. | 5 of 47 | 2.45e-05 |
| [PMID:29499948](https://www.ncbi.nlm.nih.gov/pubmed/29499948) | (2018) Endogenous Cellular MicroRNAs Mediate Antiviral Defense against Influenza A Virus. | 4 of 14 | 2.62e-05 |
| [PMID:26304123](https://www.ncbi.nlm.nih.gov/pubmed/26304123) | (2015) mRNA Targeting to Endoplasmic Reticulum Precedes Ago Protein Interaction and MicroRNA (miRNA)-mediated Translation Repression in Mammalian Cells. | 4 of 14 | 2.62e-05 |
| [PMID:24213167](https://www.ncbi.nlm.nih.gov/pubmed/24213167) | (2014) Parallel measurement of dynamic changes in translation rates in single cells. | 4 of 14 | 2.62e-05 |
| [PMID:21986946](https://www.ncbi.nlm.nih.gov/pubmed/21986946) | (2012) Small nucleolar RNA 42 acts as an oncogene in lung tumorigenesis. | 4 of 14 | 2.62e-05 |
| [PMID:28536652](https://www.ncbi.nlm.nih.gov/pubmed/28536652) | (2017) Mesencephalic astrocyte-derived neurotrophic factor reduces cell apoptosis via upregulating HSP70 in SHSY-5Y cells. | 4 of 15 | 3.06e-05 |
| [PMID:25579851](https://www.ncbi.nlm.nih.gov/pubmed/25579851) | (2016) Moderate Alcohol Drinking and the Amygdala Proteome: Identification and Validation of CalciumCalmodulin Dependent Kinase II and AMPA Receptor Activity as Novel Molecular Mechanisms of the Positive Reinforcing Effects of Alcohol. | 4 of 15 | 3.06e-05 |
| [PMID:25664316](https://www.ncbi.nlm.nih.gov/pubmed/25664316) | (2015) Isoform composition and gene expression of thick and thin filament proteins in striated muscles of mice after 30-day space flight. | 4 of 15 | 3.06e-05 |
| [PMID:23407963](https://www.ncbi.nlm.nih.gov/pubmed/23407963) | (2013) A novel role of cytosolic protein synthesis inhibition in aminoglycoside ototoxicity. | 4 of 15 | 3.06e-05 |
| [PMID:31987042](https://www.ncbi.nlm.nih.gov/pubmed/31987042) | (2020) The role of the redoxmiR-6855-3pPRDX5A axis in reversing SLUG-mediated BRCA2 silencing in breast cancer cells. | 4 of 16 | 3.53e-05 |
| [PMID:27694897](https://www.ncbi.nlm.nih.gov/pubmed/27694897) | (2017) Systematic and functional characterization of novel androgen receptor variants arising from alternative splicing in the ligand-binding domain. | 4 of 16 | 3.53e-05 |
| [PMID:27110132](https://www.ncbi.nlm.nih.gov/pubmed/27110132) | (2016) Identification of HSPA8 as a candidate biomarker for endometrial carcinoma by using iTRAQ-based proteomic analysis. | 4 of 16 | 3.53e-05 |
| [PMID:26102067](https://www.ncbi.nlm.nih.gov/pubmed/26102067) | (2015) Comparative Label-Free Mass Spectrometric Analysis of Mildly versus Severely Affected mdx Mouse Skeletal Muscles Identifies Annexin, Lamin, and Vimentin as Universal Dystrophic Markers. | 5 of 53 | 3.53e-05 |
| [PMID:23874968](https://www.ncbi.nlm.nih.gov/pubmed/23874968) | (2013) Inhibition of inducible heat shock protein-70 (hsp72) enhances bortezomib-induced cell death in human bladder cancer cells. | 4 of 16 | 3.53e-05 |
| [PMID:21480387](https://www.ncbi.nlm.nih.gov/pubmed/21480387) | (2011) Dyskerin is required for tumor cell growth through mechanisms that are independent of its role in telomerase and only partially related to its function in precursor rRNA processing. | 4 of 16 | 3.53e-05 |
| [PMID:20537126](https://www.ncbi.nlm.nih.gov/pubmed/20537126) | (2010) Epigallocatechin-3-gallate suppresses the expression of HSP70 and HSP90 and exhibits anti-tumor activity in vitro and in vivo. | 4 of 16 | 3.53e-05 |
| [PMID:20423331](https://www.ncbi.nlm.nih.gov/pubmed/20423331) | (2010) SnoRNA microarray analysis reveals changes in HACA and CD RNA levels caused by dyskerin ablation in mouse liver. | 4 of 16 | 3.53e-05 |
| [PMID:16526958](https://www.ncbi.nlm.nih.gov/pubmed/16526958) | (2006) Molecular phenotype of zebrafish ovarian follicle by serial analysis of gene expression and proteomic profiling, and comparison with the transcriptomes of other animals. | 5 of 54 | 3.53e-05 |
| [PMID:30890939](https://www.ncbi.nlm.nih.gov/pubmed/30890939) | (2019) Biochemical Pathways Triggered by Antipsychotics in Human [corrected] Oligodendrocytes: Potential of Discovering New Treatment Targets. | 5 of 55 | 3.62e-05 |
| [PMID:30894069](https://www.ncbi.nlm.nih.gov/pubmed/30894069) | (2019) Impaired TFEB-mediated lysosomal biogenesis promotes the development of pancreatitis in mice and is associated with human pancreatitis. | 4 of 17 | 3.72e-05 |
| [PMID:30805011](https://www.ncbi.nlm.nih.gov/pubmed/30805011) | (2019) A Liquid Chromatography with Tandem Mass Spectrometry-Based Proteomic Analysis of Primary Cultured Cells and Subcultured Cells Using Mouse Adipose-Derived Mesenchymal Stem Cells. | 6 of 124 | 3.72e-05 |
| [PMID:28704482](https://www.ncbi.nlm.nih.gov/pubmed/28704482) | (2017) Proteomic identification of proteins differentially expressed following overexpression of hTERT (human telomerase reverse transcriptase) in cancer cells. | 4 of 17 | 3.72e-05 |
| [PMID:28630480](https://www.ncbi.nlm.nih.gov/pubmed/28630480) | (2017) Pro-invasive stimuli and the interacting protein Hsp70 favour the route of alpha-enolase to the cell surface. | 4 of 17 | 3.72e-05 |
| [PMID:27852045](https://www.ncbi.nlm.nih.gov/pubmed/27852045) | (2016) Different BCRAbl protein suppression patterns as a converging trait of chronic myeloid leukemia cell adaptation to energy restriction. | 4 of 17 | 3.72e-05 |
| [PMID:26647757](https://www.ncbi.nlm.nih.gov/pubmed/26647757) | (2016) Valproate attenuates diabetic nephropathy through inhibition of endoplasmic reticulum stress-induced apoptosis. | 4 of 17 | 3.72e-05 |
| [PMID:26871690](https://www.ncbi.nlm.nih.gov/pubmed/26871690) | (2016) Effect of Dietary Restriction and Subsequent Re-Alimentation on the Transcriptional Profile of Bovine Skeletal Muscle. | 5 of 57 | 3.87e-05 |
| [PMID:32102213](https://www.ncbi.nlm.nih.gov/pubmed/32102213) | (2020) Augmenting Vacuolar H+-ATPase Function Prevents Cardiomyocytes from Lipid-Overload Induced Dysfunction. | 4 of 18 | 4.10e-05 |
| [PMID:31363116](https://www.ncbi.nlm.nih.gov/pubmed/31363116) | (2019) Serum anti-EIF3A autoantibody as a potential diagnostic marker for hepatocellular carcinoma. | 4 of 18 | 4.10e-05 |
| [PMID:28333953](https://www.ncbi.nlm.nih.gov/pubmed/28333953) | (2017) Involvement of Beta- and Gamma-actin isoforms in actin cytoskeleton organization and migration abilities of bleb-forming human colon cancer cells. | 4 of 18 | 4.10e-05 |
| [PMID:28057907](https://www.ncbi.nlm.nih.gov/pubmed/28057907) | (2017) Molecular cloning and characterization of porcine ribosomal protein L21. | 4 of 18 | 4.10e-05 |
| [PMID:24816817](https://www.ncbi.nlm.nih.gov/pubmed/24816817) | (2014) Massively parallel sequencing of human urinary exosomemicrovesicle RNA reveals a predominance of non-coding RNA. | 4 of 18 | 4.10e-05 |
| [PMID:23690862](https://www.ncbi.nlm.nih.gov/pubmed/23690862) | (2013) Melatonin Suppresses the Expression of 45S Preribosomal RNA and Upstream Binding Factor and Enhances the Antitumor Activity of Puromycin in MDA-MB-231 Breast Cancer Cells. | 4 of 18 | 4.10e-05 |
| [PMID:20137074](https://www.ncbi.nlm.nih.gov/pubmed/20137074) | (2010) Identification of arginine- and lysine-methylation in the proteome of Saccharomyces cerevisiae and its functional implications. | 4 of 18 | 4.10e-05 |
| [PMID:19271284](https://www.ncbi.nlm.nih.gov/pubmed/19271284) | (2009) Hop proanthocyanidins induce apoptosis, protein carbonylation, and cytoskeleton disorganization in human colorectal adenocarcinoma cells via reactive oxygen species. | 4 of 18 | 4.10e-05 |
| [PMID:18644987](https://www.ncbi.nlm.nih.gov/pubmed/18644987) | (2008) Destabilization of ERBB2 transcripts by targeting 3' untranslated region messenger RNA associated HuR and histone deacetylase-6. | 4 of 18 | 4.10e-05 |
| [PMID:29066441](https://www.ncbi.nlm.nih.gov/pubmed/29066441) | (2017) Endoplasmic Reticulum Stress Is Associated With Autophagy and Cardiomyocyte Remodeling in Experimental and Human Atrial Fibrillation. | 4 of 19 | 4.42e-05 |
| [PMID:28275690](https://www.ncbi.nlm.nih.gov/pubmed/28275690) | (2017) Intestinal Epithelial-Specific mTORC1 Activation Enhances Intestinal Adaptation After Small Bowel Resection. | 4 of 19 | 4.42e-05 |
| [PMID:25152840](https://www.ncbi.nlm.nih.gov/pubmed/25152840) | (2014) Induction of Apoptosis in Pancreatic Cancer Cells by CDDO-Me Involves Repression of Telomerase through Epigenetic Pathways. | 4 of 19 | 4.42e-05 |
| [PMID:24651535](https://www.ncbi.nlm.nih.gov/pubmed/24651535) | (2014) Towards decrypting cryptobiosis--analyzing anhydrobiosis in the tardigrade Milnesium tardigradum using transcriptome sequencing. | 4 of 19 | 4.42e-05 |
| [PMID:20931991](https://www.ncbi.nlm.nih.gov/pubmed/20931991) | (2010) Paraoxon-induced protein expression changes to SH-SY5Y cells. | 4 of 19 | 4.42e-05 |
| [PMID:20667471](https://www.ncbi.nlm.nih.gov/pubmed/20667471) | (2010) Acute hyperglycemia rapidly stimulates VEGF mRNA translation in the kidney. Role of angiotensin type 2 receptor (AT2). | 4 of 19 | 4.42e-05 |
| [PMID:20219969](https://www.ncbi.nlm.nih.gov/pubmed/20219969) | (2010) Analysis of human small nucleolar RNAs (snoRNA) and the development of snoRNA modulator of gene expression vectors. | 4 of 19 | 4.42e-05 |
| [PMID:32267851](https://www.ncbi.nlm.nih.gov/pubmed/32267851) | (2020) Proteomic analysis of the processes leading to Madurella mycetomatis grain formation in Galleria mellonella larvae. | 5 of 62 | 4.65e-05 |
| [PMID:29396395](https://www.ncbi.nlm.nih.gov/pubmed/29396395) | (2018) Replication confers Beta cell immaturity. | 5 of 62 | 4.65e-05 |
| [PMID:32194992](https://www.ncbi.nlm.nih.gov/pubmed/32194992) | (2020) TFEB-mediated lysosomal biogenesis and lysosomal drug sequestration confer resistance to MEK inhibition in pancreatic cancer. | 4 of 20 | 4.77e-05 |
| [PMID:30951670](https://www.ncbi.nlm.nih.gov/pubmed/30951670) | (2019) Reassessment of Exosome Composition. | 5 of 63 | 4.77e-05 |
| [PMID:27158675](https://www.ncbi.nlm.nih.gov/pubmed/27158675) | (2016) Eosinophilic esophagitis-linked calpain 14 is an IL-13-induced protease that mediates esophageal epithelial barrier impairment. | 4 of 20 | 4.77e-05 |
| [PMID:26554841](https://www.ncbi.nlm.nih.gov/pubmed/26554841) | (2015) Proteome Differences in Placenta and Endometrium between Normal and Intrauterine Growth Restricted Pig Fetuses. | 5 of 63 | 4.77e-05 |
| [PMID:25723318](https://www.ncbi.nlm.nih.gov/pubmed/25723318) | (2014) Proteomic analysis of the regenerating liver following 23 partial hepatectomy in rats. | 5 of 63 | 4.77e-05 |
| [PMID:21208456](https://www.ncbi.nlm.nih.gov/pubmed/21208456) | (2011) Induction of HSPA4 and HSPA14 by NBS1 overexpression contributes to NBS1-induced in vitro metastatic and transformation activity. | 4 of 20 | 4.77e-05 |
| [PMID:21364676](https://www.ncbi.nlm.nih.gov/pubmed/21364676) | (2010) Androgens modulate autophagy and cell death via regulation of the endoplasmic reticulum chaperone glucose-regulated protein 78BiP in prostate cancer cells. | 4 of 20 | 4.77e-05 |
| [PMID:19480393](https://www.ncbi.nlm.nih.gov/pubmed/19480393) | (2009) Proteomic analysis of DNA-protein cross-linking by antitumor nitrogen mustards. | 4 of 20 | 4.77e-05 |
| [PMID:31828325](https://www.ncbi.nlm.nih.gov/pubmed/31828325) | (2020) Functional diversity of small nucleolar RNAs. | 5 of 64 | 4.86e-05 |
| [PMID:31026227](https://www.ncbi.nlm.nih.gov/pubmed/31026227) | (2019) Impaired ribosome biogenesis: mechanisms and relevance to cancer and aging. | 5 of 64 | 4.86e-05 |

**KEGG Pathways**

| *pathway* | *description* | *count in gene set* | *false discovery rate* |
| --- | --- | --- | --- |
| [mmu04145](https://www.kegg.jp/kegg-bin/show_pathway?mmu04145) | Phagosome | 6 of 165 | 6.02e-06 |
| [mmu03008](https://www.kegg.jp/kegg-bin/show_pathway?mmu03008) | Ribosome biogenesis in eukaryotes | 4 of 76 | 0.00014 |
| [mmu04966](https://www.kegg.jp/kegg-bin/show_pathway?mmu04966) | Collecting duct acid secretion | 3 of 27 | 0.00022 |
| [mmu04721](https://www.kegg.jp/kegg-bin/show_pathway?mmu04721) | Synaptic vesicle cycle | 3 of 62 | 0.0018 |
| [mmu05323](https://www.kegg.jp/kegg-bin/show_pathway?mmu05323) | Rheumatoid arthritis | 3 of 81 | 0.0030 |
| [mmu04540](https://www.kegg.jp/kegg-bin/show_pathway?mmu04540) | Gap junction | 3 of 85 | 0.0030 |
| [mmu04066](https://www.kegg.jp/kegg-bin/show_pathway?mmu04066) | HIF-1 signaling pathway | 3 of 102 | 0.0042 |
| [mmu03010](https://www.kegg.jp/kegg-bin/show_pathway?mmu03010) | Ribosome | 3 of 128 | 0.0070 |
| [mmu00190](https://www.kegg.jp/kegg-bin/show_pathway?mmu00190) | Oxidative phosphorylation | 3 of 129 | 0.0070 |
| [mmu04921](https://www.kegg.jp/kegg-bin/show_pathway?mmu04921) | Oxytocin signaling pathway | 3 of 149 | 0.0086 |
| [mmu04150](https://www.kegg.jp/kegg-bin/show_pathway?mmu04150) | mTOR signaling pathway | 3 of 152 | 0.0086 |
| [mmu05203](https://www.kegg.jp/kegg-bin/show_pathway?mmu05203) | Viral carcinogenesis | 3 of 199 | 0.0162 |
| [mmu00010](https://www.kegg.jp/kegg-bin/show_pathway?mmu00010) | Glycolysis / Gluconeogenesis | 2 of 65 | 0.0233 |
| [mmu04971](https://www.kegg.jp/kegg-bin/show_pathway?mmu04971) | Gastric acid secretion | 2 of 72 | 0.0263 |
| [mmu01230](https://www.kegg.jp/kegg-bin/show_pathway?mmu01230) | Biosynthesis of amino acids | 2 of 75 | 0.0265 |
| [mmu04612](https://www.kegg.jp/kegg-bin/show_pathway?mmu04612) | Antigen processing and presentation | 2 of 78 | 0.0268 |
| [mmu04914](https://www.kegg.jp/kegg-bin/show_pathway?mmu04914) | Progesterone-mediated oocyte maturation | 2 of 90 | 0.0331 |
| [mmu04670](https://www.kegg.jp/kegg-bin/show_pathway?mmu04670) | Leukocyte transendothelial migration | 2 of 115 | 0.0497 |
| [mmu01200](https://www.kegg.jp/kegg-bin/show_pathway?mmu01200) | Carbon metabolism | 2 of 118 | 0.0497 |

**Reactome Pathways**

| *pathway* | *description* | *count in gene set* | *false discovery rate* |
| --- | --- | --- | --- |
| [MMU-168256](https://reactome.org/content/detail/R-MMU-168256) | Immune System | 11 of 1523 | 0.00016 |
| [MMU-168249](https://reactome.org/content/detail/R-MMU-168249) | Innate Immune System | 9 of 879 | 0.00016 |
| [MMU-917977](https://reactome.org/content/detail/R-MMU-917977) | Transferrin endocytosis and recycling | 3 of 30 | 0.00043 |
| [MMU-77387](https://reactome.org/content/detail/R-MMU-77387) | Insulin receptor recycling | 3 of 27 | 0.00043 |
| [MMU-1222556](https://reactome.org/content/detail/R-MMU-1222556) | ROS, RNS production in phagocytes | 3 of 32 | 0.00043 |
| [MMU-917937](https://reactome.org/content/detail/R-MMU-917937) | Iron uptake and transport | 3 of 50 | 0.0012 |
| [MMU-9006934](https://reactome.org/content/detail/R-MMU-9006934) | Signaling by Receptor Tyrosine Kinases | 5 of 360 | 0.0022 |
| [MMU-74752](https://reactome.org/content/detail/R-MMU-74752) | Signaling by Insulin receptor | 3 of 66 | 0.0022 |
| [MMU-380320](https://reactome.org/content/detail/R-MMU-380320) | Recruitment of NuMA to mitotic centrosomes | 3 of 82 | 0.0033 |
| [MMU-8868773](https://reactome.org/content/detail/R-MMU-8868773) | rRNA processing in the nucleus and cytosol | 3 of 102 | 0.0056 |
| [MMU-72312](https://reactome.org/content/detail/R-MMU-72312) | rRNA processing | 3 of 102 | 0.0056 |
| [MMU-6798695](https://reactome.org/content/detail/R-MMU-6798695) | Neutrophil degranulation | 5 of 476 | 0.0056 |
| [MMU-6791226](https://reactome.org/content/detail/R-MMU-6791226) | Major pathway of rRNA processing in the nucleolus and cytosol | 3 of 102 | 0.0056 |
| [MMU-5626467](https://reactome.org/content/detail/R-MMU-5626467) | RHO GTPases activate IQGAPs | 2 of 25 | 0.0068 |
| [MMU-8854214](https://reactome.org/content/detail/R-MMU-8854214) | TBC/RABGAPs | 2 of 29 | 0.0084 |
| [MMU-437239](https://reactome.org/content/detail/R-MMU-437239) | Recycling pathway of L1 | 2 of 33 | 0.0101 |
| [MMU-3928662](https://reactome.org/content/detail/R-MMU-3928662) | EPHB-mediated forward signaling | 2 of 34 | 0.0101 |
| [MMU-983712](https://reactome.org/content/detail/R-MMU-983712) | Ion channel transport | 3 of 159 | 0.0110 |
| [MMU-190828](https://reactome.org/content/detail/R-MMU-190828) | Gap junction trafficking | 2 of 37 | 0.0110 |
| [MMU-157858](https://reactome.org/content/detail/R-MMU-157858) | Gap junction trafficking and regulation | 2 of 39 | 0.0110 |
| [MMU-69275](https://reactome.org/content/detail/R-MMU-69275) | G2/M Transition | 3 of 169 | 0.0112 |
| [MMU-5617833](https://reactome.org/content/detail/R-MMU-5617833) | Cilium Assembly | 3 of 174 | 0.0112 |
| [MMU-453274](https://reactome.org/content/detail/R-MMU-453274) | Mitotic G2-G2/M phases | 3 of 171 | 0.0112 |
| [MMU-68877](https://reactome.org/content/detail/R-MMU-68877) | Mitotic Prometaphase | 3 of 179 | 0.0116 |
| [MMU-3371497](https://reactome.org/content/detail/R-MMU-3371497) | HSP90 chaperone cycle for steroid hormone receptors (SHR) | 2 of 48 | 0.0131 |
| [MMU-1852241](https://reactome.org/content/detail/R-MMU-1852241) | Organelle biogenesis and maintenance | 3 of 199 | 0.0144 |
| [MMU-2029482](https://reactome.org/content/detail/R-MMU-2029482) | Regulation of actin dynamics for phagocytic cup formation | 2 of 54 | 0.0152 |
| [MMU-8953854](https://reactome.org/content/detail/R-MMU-8953854) | Metabolism of RNA | 4 of 448 | 0.0154 |
| [MMU-8854518](https://reactome.org/content/detail/R-MMU-8854518) | AURKA Activation by TPX2 | 2 of 67 | 0.0190 |
| [MMU-8852276](https://reactome.org/content/detail/R-MMU-8852276) | The role of GTSE1 in G2/M progression after G2 checkpoint | 2 of 68 | 0.0190 |
| [MMU-446728](https://reactome.org/content/detail/R-MMU-446728) | Cell junction organization | 2 of 63 | 0.0190 |
| [MMU-380284](https://reactome.org/content/detail/R-MMU-380284) | Loss of proteins required for interphase microtubule organization from the centrosome | 2 of 64 | 0.0190 |
| [MMU-380259](https://reactome.org/content/detail/R-MMU-380259) | Loss of Nlp from mitotic centrosomes | 2 of 64 | 0.0190 |
| [MMU-373760](https://reactome.org/content/detail/R-MMU-373760) | L1CAM interactions | 2 of 64 | 0.0190 |
| [MMU-2682334](https://reactome.org/content/detail/R-MMU-2682334) | EPH-Ephrin signaling | 2 of 63 | 0.0190 |
| [MMU-422475](https://reactome.org/content/detail/R-MMU-422475) | Axon guidance | 3 of 248 | 0.0192 |
| [MMU-380287](https://reactome.org/content/detail/R-MMU-380287) | Centrosome maturation | 2 of 73 | 0.0197 |
| [MMU-380270](https://reactome.org/content/detail/R-MMU-380270) | Recruitment of mitotic centrosome proteins and complexes | 2 of 73 | 0.0197 |
| [MMU-2029480](https://reactome.org/content/detail/R-MMU-2029480) | Fcgamma receptor (FCGR) dependent phagocytosis | 2 of 74 | 0.0197 |
| [MMU-199991](https://reactome.org/content/detail/R-MMU-199991) | Membrane Trafficking | 4 of 523 | 0.0197 |
| [MMU-3371556](https://reactome.org/content/detail/R-MMU-3371556) | Cellular response to heat stress | 2 of 78 | 0.0201 |
| [MMU-2565942](https://reactome.org/content/detail/R-MMU-2565942) | Regulation of PLK1 Activity at G2/M Transition | 2 of 80 | 0.0206 |
| [MMU-5653656](https://reactome.org/content/detail/R-MMU-5653656) | Vesicle-mediated transport | 4 of 553 | 0.0211 |
| [MMU-1500931](https://reactome.org/content/detail/R-MMU-1500931) | Cell-Cell communication | 2 of 84 | 0.0216 |
| [MMU-5620912](https://reactome.org/content/detail/R-MMU-5620912) | Anchoring of the basal body to the plasma membrane | 2 of 88 | 0.0226 |
| [MMU-4420097](https://reactome.org/content/detail/R-MMU-4420097) | VEGFA-VEGFR2 Pathway | 2 of 87 | 0.0226 |
| [MMU-194138](https://reactome.org/content/detail/R-MMU-194138) | Signaling by VEGF | 2 of 94 | 0.0250 |
| [MMU-9007101](https://reactome.org/content/detail/R-MMU-9007101) | Rab regulation of trafficking | 2 of 101 | 0.0281 |
| [MMU-68886](https://reactome.org/content/detail/R-MMU-68886) | M Phase | 3 of 317 | 0.0281 |
| [MMU-2262752](https://reactome.org/content/detail/R-MMU-2262752) | Cellular responses to stress | 3 of 327 | 0.0294 |
| [MMU-392499](https://reactome.org/content/detail/R-MMU-392499) | Metabolism of proteins | 6 of 1497 | 0.0356 |
| [MMU-5663220](https://reactome.org/content/detail/R-MMU-5663220) | RHO GTPases Activate Formins | 2 of 125 | 0.0387 |
| [MMU-8953897](https://reactome.org/content/detail/R-MMU-8953897) | Cellular responses to external stimuli | 3 of 382 | 0.0422 |

**UniProt Keywords**

| *keyword* | *description* | *count in gene set* | *false discovery rate* |
| --- | --- | --- | --- |
| [KW-0832](https://www.uniprot.org/keywords/KW-0832) | Ubl conjugation | 16 of 2091 | 3.62e-08 |
| [KW-0488](https://www.uniprot.org/keywords/KW-0488) | Methylation | 12 of 922 | 3.62e-08 |
| [KW-1017](https://www.uniprot.org/keywords/KW-1017) | Isopeptide bond | 13 of 1442 | 1.67e-07 |
| [KW-0687](https://www.uniprot.org/keywords/KW-0687) | Ribonucleoprotein | 7 of 281 | 1.13e-06 |
| [KW-0007](https://www.uniprot.org/keywords/KW-0007) | Acetylation | 16 of 3060 | 1.80e-06 |
| [KW-0547](https://www.uniprot.org/keywords/KW-0547) | Nucleotide-binding | 12 of 1738 | 7.33e-06 |
| [KW-0597](https://www.uniprot.org/keywords/KW-0597) | Phosphoprotein | 22 of 7545 | 2.35e-05 |
| [KW-0690](https://www.uniprot.org/keywords/KW-0690) | Ribosome biogenesis | 4 of 74 | 2.80e-05 |
| [KW-0342](https://www.uniprot.org/keywords/KW-0342) | GTP-binding | 6 of 324 | 2.80e-05 |
| [KW-0963](https://www.uniprot.org/keywords/KW-0963) | Cytoplasm | 16 of 4690 | 0.00030 |
| [KW-0375](https://www.uniprot.org/keywords/KW-0375) | Hydrogen ion transport | 3 of 48 | 0.00030 |
| [KW-0251](https://www.uniprot.org/keywords/KW-0251) | Elongation factor | 2 of 15 | 0.0014 |
| [KW-0324](https://www.uniprot.org/keywords/KW-0324) | Glycolysis | 2 of 32 | 0.0053 |
| [KW-0944](https://www.uniprot.org/keywords/KW-0944) | Nitration | 2 of 42 | 0.0082 |
| [KW-0689](https://www.uniprot.org/keywords/KW-0689) | Ribosomal protein | 3 of 182 | 0.0088 |
| [KW-0702](https://www.uniprot.org/keywords/KW-0702) | S-nitrosylation | 2 of 52 | 0.0108 |
| [KW-0206](https://www.uniprot.org/keywords/KW-0206) | Cytoskeleton | 6 of 1159 | 0.0141 |
| [KW-0698](https://www.uniprot.org/keywords/KW-0698) | rRNA processing | 2 of 84 | 0.0239 |
| [KW-0067](https://www.uniprot.org/keywords/KW-0067) | ATP-binding | 6 of 1352 | 0.0267 |
| [KW-0539](https://www.uniprot.org/keywords/KW-0539) | Nucleus | 12 of 4624 | 0.0301 |
| [KW-0810](https://www.uniprot.org/keywords/KW-0810) | Translation regulation | 2 of 110 | 0.0337 |
| [KW-0694](https://www.uniprot.org/keywords/KW-0694) | RNA-binding | 4 of 647 | 0.0337 |
| [KW-0379](https://www.uniprot.org/keywords/KW-0379) | Hydroxylation | 2 of 120 | 0.0367 |

**PFAM Protein Domains**

| *domain* | *description* | *count in gene set* | *false discovery rate* |
| --- | --- | --- | --- |
| [PF02874](https://pfam.xfam.org/family/PF02874) | ATP synthase alpha/beta family, beta-barrel domain | 2 of 5 | 0.0016 |
| [PF00006](https://pfam.xfam.org/family/PF00006) | ATP synthase alpha/beta family, nucleotide-binding domain | 2 of 5 | 0.0016 |
| [PF03144](https://pfam.xfam.org/family/PF03144) | Elongation factor Tu domain 2 | 2 of 14 | 0.0030 |
| [PF03953](https://pfam.xfam.org/family/PF03953) | Tubulin C-terminal domain | 2 of 20 | 0.0043 |
| [PF00091](https://pfam.xfam.org/family/PF00091) | Tubulin/FtsZ family, GTPase domain | 2 of 22 | 0.0043 |
| [PF00009](https://pfam.xfam.org/family/PF00009) | Elongation factor Tu GTP binding domain | 2 of 23 | 0.0043 |

**INTERPRO Protein Domains and Features**

| *domain* | *description* | *count in gene set* | *false discovery rate* |
| --- | --- | --- | --- |
| [IPR009000](https://www.ebi.ac.uk/interpro/entry/IPR009000) | Translation protein, beta-barrel domain superfamily | 3 of 30 | 0.0010 |
| [IPR031157](https://www.ebi.ac.uk/interpro/entry/IPR031157) | Tr-type G domain, conserved site | 2 of 9 | 0.0018 |
| [IPR027417](https://www.ebi.ac.uk/interpro/entry/IPR027417) | P-loop containing nucleoside triphosphate hydrolase | 7 of 877 | 0.0018 |
| [IPR020003](https://www.ebi.ac.uk/interpro/entry/IPR020003) | ATPase, alpha/beta subunit, nucleotide-binding domain, active site | 2 of 5 | 0.0018 |
| [IPR004100](https://www.ebi.ac.uk/interpro/entry/IPR004100) | ATPase, F1/V1/A1 complex, alpha/beta subunit, N-terminal domain | 2 of 5 | 0.0018 |
| [IPR000194](https://www.ebi.ac.uk/interpro/entry/IPR000194) | ATPase, F1/V1/A1 complex, alpha/beta subunit, nucleotide-binding domain | 2 of 5 | 0.0018 |
| [IPR004161](https://www.ebi.ac.uk/interpro/entry/IPR004161) | Translation elongation factor EFTu-like, domain 2 | 2 of 14 | 0.0028 |
| [IPR037103](https://www.ebi.ac.uk/interpro/entry/IPR037103) | Tubulin/FtsZ, C-terminal domain superfamily | 2 of 17 | 0.0035 |
| [IPR036525](https://www.ebi.ac.uk/interpro/entry/IPR036525) | Tubulin/FtsZ, GTPase domain superfamily | 2 of 20 | 0.0035 |
| [IPR023123](https://www.ebi.ac.uk/interpro/entry/IPR023123) | Tubulin, C-terminal | 2 of 19 | 0.0035 |
| [IPR018316](https://www.ebi.ac.uk/interpro/entry/IPR018316) | Tubulin/FtsZ, 2-layer sandwich domain | 2 of 18 | 0.0035 |
| [IPR017975](https://www.ebi.ac.uk/interpro/entry/IPR017975) | Tubulin, conserved site | 2 of 19 | 0.0035 |
| [IPR008280](https://www.ebi.ac.uk/interpro/entry/IPR008280) | Tubulin/FtsZ, C-terminal | 2 of 19 | 0.0035 |
| [IPR003008](https://www.ebi.ac.uk/interpro/entry/IPR003008) | Tubulin/FtsZ, GTPase domain | 2 of 19 | 0.0035 |
| [IPR000795](https://www.ebi.ac.uk/interpro/entry/IPR000795) | Transcription factor, GTP-binding domain | 2 of 19 | 0.0035 |
| [IPR000217](https://www.ebi.ac.uk/interpro/entry/IPR000217) | Tubulin | 2 of 19 | 0.0035 |
| [IPR020568](https://www.ebi.ac.uk/interpro/entry/IPR020568) | Ribosomal protein S5 domain 2-type fold | 2 of 34 | 0.0060 |
| [IPR005225](https://www.ebi.ac.uk/interpro/entry/IPR005225) | Small GTP-binding protein domain | 3 of 171 | 0.0082 |

**Supplementary Table 2.** List of Rab35-interacting proteins in samples from Raw 264.7 macrophages.

| UniProt ID | Protein name | Description | MW (kDa) | Scores  (Mascot) | #Peptides | SC (%) |
| --- | --- | --- | --- | --- | --- | --- |
| **P25911** | LYN_MOUSE | Tyrosine-protein kinase Lyn | 58.8 | 285.0 | 5 | 10.7 |
| **Q9DAJ4** | WDR83_MOUSE | WD repeat domain-containing protein 83 | 34.4 | 244.0 | 3 | 11.7 |
| **Q9DC51** | GNAI3_MOUSE | Guanine nucleotide-binding protein G(k) subunit alpha | 40.5 | 241.7 | 7 | 17.5 |
| **Q8BG79** | C19L2_MOUSE | CWF19-like protein 2 | 103.1 | 173.0 | 6 | 8.3 |
| **Q9Z1G4** | VPP1_MOUSE | V-type proton ATPase 116 kDa subunit a isoform 1 | 96.4 | 143.8 | 3 | 4.2 |
| **P58242** | ASM3B_MOUSE | Acid sphingomyelinase-like phosphodiesterase 3b | 51.6 | 133.7 | 3 | 9.6 |
| **Q8R2Q8** | BST2_MOUSE | Bone marrow stromal antigen 2 | 19.1 | 132.8 | 3 | 16.9 |
| **P60335** | PCBP1_MOUSE | Poly(rC)-binding protein 1 | 37.5 | 112.5 | 3 | 11.8 |
| **Q52KI8** | SRRM1_MOUSE | Serine/arginine repetitive matrix protein 1 | 106.8 | 104.2 | 2 | 3.0 |
| **O08602** | RAE1A_MOUSE | Retinoic acid early-inducible protein 1-alpha | 28.6 | 102.1 | 2 | 7.5 |
| **Q5SUA5** | MYO1G_MOUSE | Unconventional myosin-Ig | 117.2 | 99.8 | 3 | 3.5 |
| **O09044** | SNP23_MOUSE | Synaptosomal-associated protein 23 | 23.2 | 85.4 | 3 | 12.9 |
| **P15379** | CD44_MOUSE | CD44 antigen | 85.6 | 80.4 | 2 | 2.4 |
| **P27659** | RL3_MOUSE | 60S ribosomal protein L3 | 46.1 | 240.3 | 8 | 18.9 |
| **P62754** | RS6_MOUSE | 40S ribosomal protein S6 | 28.7 | 187.4 | 3 | 14.1 |
| **P10126** | EF1A1_MOUSE | Elongation factor 1-alpha 1 | 50.1 | 166.5 | 4 | 8.7 |
| **Q9D8E6** | RL4_MOUSE | 60S ribosomal protein L4 | 47.1 | 98.7 | 2 | 6.7 |
| **P84099** | RL19_MOUSE | 60S ribosomal protein L19 | 23.5 | 95.0 | 2 | 8.7 |
| **B2RY56** | RBM25_MOUSE | RNA-binding protein 25 | 99.5 | 395.5 | 7 | 11.2 |
| **Q6NSQ7** | LTV1_MOUSE | Protein LTV1 homolog | 54.0 | 237.1 | 4 | 11.5 |
| **O08784** | TCOF_MOUSE | Treacle protein | 134.9 | 224.4 | 7 | 6.7 |
| **Q9CQW9** | IFM3_MOUSE | Interferon-induced transmembrane protein 3 | 14.9 | 93.8 | 1 | 14.6 |
| **P50516** | VATA_MOUSE | V-type proton ATPase catalytic subunit A | 68.3 | 705.6 | 15 | 27.6 |
| **P10810** | CD14_MOUSE | Monocyte differentiation antigen CD14 | 39.2 | 553.6 | 9 | 31.7 |
| **P62814** | VATB2_MOUSE | V-type proton ATPase subunit B, brain isoform | 56.5 | 541.5 | 10 | 27.0 |
| **P08752** | GNAI2_MOUSE | Guanine nucleotide-binding protein G(i) subunit alpha-2 | 40.5 | 516.3 | 9 | 30.7 |
| **P51863** | VA0D1_MOUSE | V-type proton ATPase subunit d 1 | 40.3 | 512.8 | 9 | 33.6 |
| **P60710** | ACTB_MOUSE | Actin, cytoplasmic 1 | 41.7 | 237.4 | 5 | 17.3 |
| **P54116** | STOM_MOUSE | Erythrocyte band 7 integral membrane protein | 31.4 | 198.7 | 4 | 18.3 |
| **P50518** | VATE1_MOUSE | V-type proton ATPase subunit E 1 | 26.1 | 197.0 | 5 | 22.1 |
| **P63082** | VATL_MOUSE | V-type proton ATPase 16 kDa proteolipid subunit | 15.8 | 171.8 | 3 | 11.6 |
| **Q9JIY5** | HTRA2_MOUSE | Serine protease HTRA2, mitochondrial | 49.3 | 141.6 | 3 | 8.5 |
| **Q9Z1G3** | VATC1_MOUSE | V-type proton ATPase subunit C 1 | 43.9 | 125.3 | 2 | 6.8 |
| **P57746** | VATD_MOUSE | V-type proton ATPase subunit D | 28.4 | 107.5 | 2 | 11.3 |
| **P62806** | H4_MOUSE | Histone H4 | 11.4 | 105.4 | 3 | 26.2 |
| **Q922Q2** | RIOK1_MOUSE | Serine/threonine-protein kinase RIO1 | 64.9 | 101.7 | 2 | 3.5 |
| **Q78WZ7** | RPA43_MOUSE | DNA-directed RNA polymerase I subunit RPA43 | 36.7 | 96.4 | 2 | 7.9 |
| **P45376** | ALDR_MOUSE | Aldose reductase | 35.7 | 93.3 | 2 | 7.0 |
| **P62880** | GBB2_MOUSE | Guanine nucleotide-binding protein G(I)/G(S)/G(T) subunit beta-2 | 37.3 | 92.6 | 2 | 6.2 |
| **P20029** | GRP78_MOUSE | 78 kDa glucose-regulated protein | 72.4 | 309.9 | 6 | 11.3 |

Conserved proteins identified between GFP-tag control vector and GFP-tag Rab35 DN were discarded (results from 4 independent experiments). *SC: Sequence coverage. Rab35 was identified but not included in the table.

**Network Stats**

| number of nodes: 39  number of edges: 78  average node degree: 4  avg. local clustering coefficient: 0.61 |
| --- |

| expected number of edges: 28  PPI enrichment p-value: 6.77e-15 |
| --- |

**Supplementary Table 3.** Functional enrichments in Rab35 (DN)-interacting protein network

**Biological Process (GO)**

| *GO-term* | *description* | *count in gene set* | *false discovery rate* |
| --- | --- | --- | --- |
| [GO:0015991](http://amigo.geneontology.org/amigo/term/GO:0015991) | ATP hydrolysis coupled proton transport | 7 of 25 | 1.49e-10 |
| [GO:0070887](http://amigo.geneontology.org/amigo/term/GO:0070887) | cellular response to chemical stimulus | 15 of 2287 | 0.00084 |
| [GO:0007035](http://amigo.geneontology.org/amigo/term/GO:0007035) | vacuolar acidification | 3 of 18 | 0.0011 |
| [GO:0006812](http://amigo.geneontology.org/amigo/term/GO:0006812) | cation transport | 9 of 795 | 0.0013 |
| [GO:0071310](http://amigo.geneontology.org/amigo/term/GO:0071310) | cellular response to organic substance | 13 of 1858 | 0.0015 |
| [GO:0006810](http://amigo.geneontology.org/amigo/term/GO:0006810) | transport | 17 of 3187 | 0.0015 |
| [GO:0006811](http://amigo.geneontology.org/amigo/term/GO:0006811) | ion transport | 10 of 1156 | 0.0025 |
| [GO:0034641](http://amigo.geneontology.org/amigo/term/GO:0034641) | cellular nitrogen compound metabolic process | 19 of 4247 | 0.0029 |
| [GO:0051179](http://amigo.geneontology.org/amigo/term/GO:0051179) | localization | 19 of 4315 | 0.0034 |
| [GO:0009987](http://amigo.geneontology.org/amigo/term/GO:0009987) | cellular process | 34 of 12459 | 0.0034 |
| [GO:0035456](http://amigo.geneontology.org/amigo/term/GO:0035456) | response to interferon-beta | 3 of 37 | 0.0035 |
| [GO:0034097](http://amigo.geneontology.org/amigo/term/GO:0034097) | response to cytokine | 8 of 792 | 0.0044 |
| [GO:0033864](http://amigo.geneontology.org/amigo/term/GO:0033864) | positive regulation of NAD(P)H oxidase activity | 2 of 5 | 0.0044 |
| [GO:0010033](http://amigo.geneontology.org/amigo/term/GO:0010033) | response to organic substance | 14 of 2553 | 0.0044 |
| [GO:0002553](http://amigo.geneontology.org/amigo/term/GO:0002553) | histamine secretion by mast cell | 2 of 5 | 0.0044 |
| [GO:0002431](http://amigo.geneontology.org/amigo/term/GO:0002431) | Fc receptor mediated stimulatory signaling pathway | 2 of 5 | 0.0044 |
| [GO:0002252](http://amigo.geneontology.org/amigo/term/GO:0002252) | immune effector process | 6 of 395 | 0.0044 |
| [GO:0043603](http://amigo.geneontology.org/amigo/term/GO:0043603) | cellular amide metabolic process | 7 of 644 | 0.0060 |
| [GO:0071840](http://amigo.geneontology.org/amigo/term/GO:0071840) | cellular component organization or biogenesis | 19 of 4730 | 0.0069 |
| [GO:0042221](http://amigo.geneontology.org/amigo/term/GO:0042221) | response to chemical | 16 of 3532 | 0.0075 |
| [GO:0006955](http://amigo.geneontology.org/amigo/term/GO:0006955) | immune response | 8 of 914 | 0.0075 |
| [GO:0006412](http://amigo.geneontology.org/amigo/term/GO:0006412) | translation | 5 of 313 | 0.0094 |
| [GO:0036295](http://amigo.geneontology.org/amigo/term/GO:0036295) | cellular response to increased oxygen levels | 2 of 12 | 0.0106 |
| [GO:0045069](http://amigo.geneontology.org/amigo/term/GO:0045069) | regulation of viral genome replication | 3 of 72 | 0.0110 |
| [GO:0044085](http://amigo.geneontology.org/amigo/term/GO:0044085) | cellular component biogenesis | 12 of 2213 | 0.0110 |
| [GO:0042274](http://amigo.geneontology.org/amigo/term/GO:0042274) | ribosomal small subunit biogenesis | 3 of 70 | 0.0110 |
| [GO:0009205](http://amigo.geneontology.org/amigo/term/GO:0009205) | purine ribonucleoside triphosphate metabolic process | 4 of 187 | 0.0116 |
| [GO:0006807](http://amigo.geneontology.org/amigo/term/GO:0006807) | nitrogen compound metabolic process | 23 of 6983 | 0.0125 |
| [GO:0010941](http://amigo.geneontology.org/amigo/term/GO:0010941) | regulation of cell death | 10 of 1640 | 0.0133 |
| [GO:0009628](http://amigo.geneontology.org/amigo/term/GO:0009628) | response to abiotic stimulus | 8 of 1063 | 0.0139 |
| [GO:0055085](http://amigo.geneontology.org/amigo/term/GO:0055085) | transmembrane transport | 8 of 1073 | 0.0145 |
| [GO:0032930](http://amigo.geneontology.org/amigo/term/GO:0032930) | positive regulation of superoxide anion generation | 2 of 19 | 0.0160 |
| [GO:0009408](http://amigo.geneontology.org/amigo/term/GO:0009408) | response to heat | 3 of 94 | 0.0166 |
| [GO:0002902](http://amigo.geneontology.org/amigo/term/GO:0002902) | regulation of B cell apoptotic process | 2 of 20 | 0.0167 |
| [GO:0016241](http://amigo.geneontology.org/amigo/term/GO:0016241) | regulation of macroautophagy | 3 of 97 | 0.0175 |
| [GO:1901701](http://amigo.geneontology.org/amigo/term/GO:1901701) | cellular response to oxygen-containing compound | 7 of 870 | 0.0176 |
| [GO:0050776](http://amigo.geneontology.org/amigo/term/GO:0050776) | regulation of immune response | 6 of 635 | 0.0184 |
| [GO:0048661](http://amigo.geneontology.org/amigo/term/GO:0048661) | positive regulation of smooth muscle cell proliferation | 3 of 105 | 0.0189 |
| [GO:0044237](http://amigo.geneontology.org/amigo/term/GO:0044237) | cellular metabolic process | 23 of 7348 | 0.0189 |
| [GO:0042981](http://amigo.geneontology.org/amigo/term/GO:0042981) | regulation of apoptotic process | 9 of 1476 | 0.0189 |
| [GO:0035455](http://amigo.geneontology.org/amigo/term/GO:0035455) | response to interferon-alpha | 2 of 23 | 0.0189 |
| [GO:0002683](http://amigo.geneontology.org/amigo/term/GO:0002683) | negative regulation of immune system process | 5 of 421 | 0.0189 |
| [GO:0071363](http://amigo.geneontology.org/amigo/term/GO:0071363) | cellular response to growth factor stimulus | 5 of 437 | 0.0200 |
| [GO:0042254](http://amigo.geneontology.org/amigo/term/GO:0042254) | ribosome biogenesis | 4 of 252 | 0.0200 |
| [GO:0035809](http://amigo.geneontology.org/amigo/term/GO:0035809) | regulation of urine volume | 2 of 25 | 0.0200 |
| [GO:0006518](http://amigo.geneontology.org/amigo/term/GO:0006518) | peptide metabolic process | 5 of 440 | 0.0200 |
| [GO:0071345](http://amigo.geneontology.org/amigo/term/GO:0071345) | cellular response to cytokine stimulus | 6 of 676 | 0.0211 |
| [GO:0071353](http://amigo.geneontology.org/amigo/term/GO:0071353) | cellular response to interleukin-4 | 2 of 27 | 0.0213 |
| [GO:0006954](http://amigo.geneontology.org/amigo/term/GO:0006954) | inflammatory response | 5 of 454 | 0.0214 |
| [GO:0006360](http://amigo.geneontology.org/amigo/term/GO:0006360) | transcription by RNA polymerase I | 2 of 28 | 0.0223 |
| [GO:0009636](http://amigo.geneontology.org/amigo/term/GO:0009636) | response to toxic substance | 5 of 471 | 0.0238 |
| [GO:0051716](http://amigo.geneontology.org/amigo/term/GO:0051716) | cellular response to stimulus | 18 of 5142 | 0.0239 |
| [GO:0010467](http://amigo.geneontology.org/amigo/term/GO:0010467) | gene expression | 13 of 3013 | 0.0239 |
| [GO:0006396](http://amigo.geneontology.org/amigo/term/GO:0006396) | RNA processing | 6 of 715 | 0.0245 |
| [GO:0050896](http://amigo.geneontology.org/amigo/term/GO:0050896) | response to stimulus | 21 of 6616 | 0.0255 |
| [GO:1904707](http://amigo.geneontology.org/amigo/term/GO:1904707) | positive regulation of vascular smooth muscle cell proliferation | 2 of 32 | 0.0256 |
| [GO:0071495](http://amigo.geneontology.org/amigo/term/GO:0071495) | cellular response to endogenous stimulus | 7 of 997 | 0.0256 |
| [GO:0034122](http://amigo.geneontology.org/amigo/term/GO:0034122) | negative regulation of toll-like receptor signaling pathway | 2 of 32 | 0.0256 |
| [GO:0031663](http://amigo.geneontology.org/amigo/term/GO:0031663) | lipopolysaccharide-mediated signaling pathway | 2 of 32 | 0.0256 |
| [GO:0009719](http://amigo.geneontology.org/amigo/term/GO:0009719) | response to endogenous stimulus | 8 of 1289 | 0.0256 |
| [GO:0060548](http://amigo.geneontology.org/amigo/term/GO:0060548) | negative regulation of cell death | 7 of 1004 | 0.0258 |
| [GO:2000108](http://amigo.geneontology.org/amigo/term/GO:2000108) | positive regulation of leukocyte apoptotic process | 2 of 34 | 0.0268 |
| [GO:0016192](http://amigo.geneontology.org/amigo/term/GO:0016192) | vesicle-mediated transport | 7 of 1020 | 0.0276 |
| [GO:0071704](http://amigo.geneontology.org/amigo/term/GO:0071704) | organic substance metabolic process | 23 of 7733 | 0.0282 |
| [GO:1903707](http://amigo.geneontology.org/amigo/term/GO:1903707) | negative regulation of hemopoiesis | 3 of 143 | 0.0307 |
| [GO:0002366](http://amigo.geneontology.org/amigo/term/GO:0002366) | leukocyte activation involved in immune response | 3 of 147 | 0.0328 |
| [GO:0045087](http://amigo.geneontology.org/amigo/term/GO:0045087) | innate immune response | 5 of 534 | 0.0336 |
| [GO:0002376](http://amigo.geneontology.org/amigo/term/GO:0002376) | immune system process | 9 of 1703 | 0.0336 |
| [GO:1901564](http://amigo.geneontology.org/amigo/term/GO:1901564) | organonitrogen compound metabolic process | 16 of 4480 | 0.0346 |
| [GO:1901998](http://amigo.geneontology.org/amigo/term/GO:1901998) | toxin transport | 2 of 42 | 0.0348 |
| [GO:1901360](http://amigo.geneontology.org/amigo/term/GO:1901360) | organic cyclic compound metabolic process | 15 of 4057 | 0.0348 |
| [GO:0045071](http://amigo.geneontology.org/amigo/term/GO:0045071) | negative regulation of viral genome replication | 2 of 41 | 0.0348 |
| [GO:0035690](http://amigo.geneontology.org/amigo/term/GO:0035690) | cellular response to drug | 4 of 325 | 0.0348 |
| [GO:0006952](http://amigo.geneontology.org/amigo/term/GO:0006952) | defense response | 7 of 1079 | 0.0348 |
| [GO:0010976](http://amigo.geneontology.org/amigo/term/GO:0010976) | positive regulation of neuron projection development | 4 of 333 | 0.0355 |
| [GO:0002443](http://amigo.geneontology.org/amigo/term/GO:0002443) | leukocyte mediated immunity | 3 of 158 | 0.0355 |
| [GO:0065007](http://amigo.geneontology.org/amigo/term/GO:0065007) | biological regulation | 27 of 10168 | 0.0363 |
| [GO:0046034](http://amigo.geneontology.org/amigo/term/GO:0046034) | ATP metabolic process | 3 of 162 | 0.0363 |
| [GO:0044238](http://amigo.geneontology.org/amigo/term/GO:0044238) | primary metabolic process | 22 of 7426 | 0.0363 |
| [GO:0034605](http://amigo.geneontology.org/amigo/term/GO:0034605) | cellular response to heat | 2 of 44 | 0.0363 |
| [GO:0016043](http://amigo.geneontology.org/amigo/term/GO:0016043) | cellular component organization | 16 of 4560 | 0.0366 |
| [GO:1901566](http://amigo.geneontology.org/amigo/term/GO:1901566) | organonitrogen compound biosynthetic process | 7 of 1122 | 0.0379 |
| [GO:1990090](http://amigo.geneontology.org/amigo/term/GO:1990090) | cellular response to nerve growth factor stimulus | 2 of 47 | 0.0388 |
| [GO:0002757](http://amigo.geneontology.org/amigo/term/GO:0002757) | immune response-activating signal transduction | 3 of 168 | 0.0388 |
| [GO:0009150](http://amigo.geneontology.org/amigo/term/GO:0009150) | purine ribonucleotide metabolic process | 4 of 356 | 0.0408 |
| [GO:0007399](http://amigo.geneontology.org/amigo/term/GO:0007399) | nervous system development | 10 of 2181 | 0.0427 |
| [GO:0051602](http://amigo.geneontology.org/amigo/term/GO:0051602) | response to electrical stimulus | 2 of 51 | 0.0430 |
| [GO:0002682](http://amigo.geneontology.org/amigo/term/GO:0002682) | regulation of immune system process | 7 of 1165 | 0.0434 |
| [GO:0071216](http://amigo.geneontology.org/amigo/term/GO:0071216) | cellular response to biotic stimulus | 3 of 180 | 0.0437 |
| [GO:2000377](http://amigo.geneontology.org/amigo/term/GO:2000377) | regulation of reactive oxygen species metabolic process | 3 of 184 | 0.0457 |
| [GO:1901135](http://amigo.geneontology.org/amigo/term/GO:1901135) | carbohydrate derivative metabolic process | 6 of 882 | 0.0457 |
| [GO:0043066](http://amigo.geneontology.org/amigo/term/GO:0043066) | negative regulation of apoptotic process | 6 of 884 | 0.0457 |
| [GO:0017157](http://amigo.geneontology.org/amigo/term/GO:0017157) | regulation of exocytosis | 3 of 184 | 0.0457 |
| [GO:0002181](http://amigo.geneontology.org/amigo/term/GO:0002181) | cytoplasmic translation | 2 of 55 | 0.0460 |
| [GO:0006879](http://amigo.geneontology.org/amigo/term/GO:0006879) | cellular iron ion homeostasis | 2 of 56 | 0.0466 |
| [GO:0006397](http://amigo.geneontology.org/amigo/term/GO:0006397) | mRNA processing | 4 of 384 | 0.0466 |
| [GO:0071300](http://amigo.geneontology.org/amigo/term/GO:0071300) | cellular response to retinoic acid | 2 of 57 | 0.0473 |
| [GO:0052548](http://amigo.geneontology.org/amigo/term/GO:0052548) | regulation of endopeptidase activity | 4 of 387 | 0.0473 |
| [GO:0048699](http://amigo.geneontology.org/amigo/term/GO:0048699) | generation of neurons | 8 of 1538 | 0.0473 |
| [GO:0006725](http://amigo.geneontology.org/amigo/term/GO:0006725) | cellular aromatic compound metabolic process | 14 of 3879 | 0.0473 |
| [GO:0016072](http://amigo.geneontology.org/amigo/term/GO:0016072) | rRNA metabolic process | 3 of 196 | 0.0487 |
| [GO:0009167](http://amigo.geneontology.org/amigo/term/GO:0009167) | purine ribonucleoside monophosphate metabolic process | 3 of 196 | 0.0487 |

**Molecular Function (GO)**

| *GO-term* | *description* | *count in gene set* | *false discovery rate* |
| --- | --- | --- | --- |
| [GO:0046961](http://amigo.geneontology.org/amigo/term/GO:0046961) | proton-transporting ATPase activity, rotational mechanism | 6 of 18 | 5.43e-10 |
| [GO:0017111](http://amigo.geneontology.org/amigo/term/GO:0017111) | nucleoside-triphosphatase activity | 13 of 714 | 9.00e-09 |
| [GO:0042626](http://amigo.geneontology.org/amigo/term/GO:0042626) | ATPase activity, coupled to transmembrane movement of substances | 7 of 105 | 2.45e-08 |
| [GO:0008553](http://amigo.geneontology.org/amigo/term/GO:0008553) | proton-exporting ATPase activity, phosphorylative mechanism | 4 of 14 | 3.39e-07 |
| [GO:0016787](http://amigo.geneontology.org/amigo/term/GO:0016787) | hydrolase activity | 17 of 2259 | 1.04e-06 |
| [GO:0016887](http://amigo.geneontology.org/amigo/term/GO:0016887) | ATPase activity | 8 of 372 | 3.12e-06 |
| [GO:0097367](http://amigo.geneontology.org/amigo/term/GO:0097367) | carbohydrate derivative binding | 13 of 2051 | 0.00028 |
| [GO:0035639](http://amigo.geneontology.org/amigo/term/GO:0035639) | purine ribonucleoside triphosphate binding | 11 of 1697 | 0.0011 |
| [GO:1901363](http://amigo.geneontology.org/amigo/term/GO:1901363) | heterocyclic compound binding | 19 of 4748 | 0.0013 |
| [GO:0003735](http://amigo.geneontology.org/amigo/term/GO:0003735) | structural constituent of ribosome | 4 of 153 | 0.0013 |
| [GO:0097159](http://amigo.geneontology.org/amigo/term/GO:0097159) | organic cyclic compound binding | 19 of 4818 | 0.0014 |
| [GO:0032555](http://amigo.geneontology.org/amigo/term/GO:0032555) | purine ribonucleotide binding | 11 of 1766 | 0.0014 |
| [GO:0003824](http://amigo.geneontology.org/amigo/term/GO:0003824) | catalytic activity | 20 of 5239 | 0.0014 |
| [GO:0019843](http://amigo.geneontology.org/amigo/term/GO:0019843) | rRNA binding | 3 of 67 | 0.0016 |
| [GO:0008097](http://amigo.geneontology.org/amigo/term/GO:0008097) | 5S rRNA binding | 2 of 13 | 0.0019 |
| [GO:0005488](http://amigo.geneontology.org/amigo/term/GO:0005488) | binding | 30 of 10884 | 0.0024 |
| [GO:0003729](http://amigo.geneontology.org/amigo/term/GO:0003729) | mRNA binding | 4 of 202 | 0.0027 |
| [GO:0036094](http://amigo.geneontology.org/amigo/term/GO:0036094) | small molecule binding | 12 of 2364 | 0.0029 |
| [GO:0031683](http://amigo.geneontology.org/amigo/term/GO:0031683) | G-protein beta/gamma-subunit complex binding | 2 of 25 | 0.0052 |
| [GO:0019899](http://amigo.geneontology.org/amigo/term/GO:0019899) | enzyme binding | 11 of 2175 | 0.0052 |
| [GO:0003924](http://amigo.geneontology.org/amigo/term/GO:0003924) | GTPase activity | 4 of 255 | 0.0052 |
| [GO:0043168](http://amigo.geneontology.org/amigo/term/GO:0043168) | anion binding | 12 of 2578 | 0.0056 |
| [GO:0003723](http://amigo.geneontology.org/amigo/term/GO:0003723) | RNA binding | 7 of 986 | 0.0070 |
| [GO:0005198](http://amigo.geneontology.org/amigo/term/GO:0005198) | structural molecule activity | 5 of 546 | 0.0111 |
| [GO:0005525](http://amigo.geneontology.org/amigo/term/GO:0005525) | GTP binding | 4 of 338 | 0.0121 |
| [GO:0070063](http://amigo.geneontology.org/amigo/term/GO:0070063) | RNA polymerase binding | 2 of 52 | 0.0144 |
| [GO:0019003](http://amigo.geneontology.org/amigo/term/GO:0019003) | GDP binding | 2 of 62 | 0.0198 |
| [GO:0005546](http://amigo.geneontology.org/amigo/term/GO:0005546) | phosphatidylinositol-4,5-bisphosphate binding | 2 of 68 | 0.0231 |
| [GO:0008144](http://amigo.geneontology.org/amigo/term/GO:0008144) | drug binding | 8 of 1630 | 0.0233 |
| [GO:0005524](http://amigo.geneontology.org/amigo/term/GO:0005524) | ATP binding | 7 of 1389 | 0.0338 |
| [GO:0051117](http://amigo.geneontology.org/amigo/term/GO:0051117) | ATPase binding | 2 of 91 | 0.0380 |
| [GO:0090079](http://amigo.geneontology.org/amigo/term/GO:0090079) | translation regulator activity, nucleic acid binding | 2 of 92 | 0.0382 |
| [GO:0051219](http://amigo.geneontology.org/amigo/term/GO:0051219) | phosphoprotein binding | 2 of 98 | 0.0405 |

**Cellular Component (GO)**

| *GO-term* | *description* | *count in gene set* | *false discovery rate* |
| --- | --- | --- | --- |
| [GO:0033176](http://amigo.geneontology.org/amigo/term/GO:0033176) | proton-transporting V-type ATPase complex | 7 of 22 | 1.24e-11 |
| [GO:0016469](http://amigo.geneontology.org/amigo/term/GO:0016469) | proton-transporting two-sector ATPase complex | 8 of 46 | 1.24e-11 |
| [GO:0032991](http://amigo.geneontology.org/amigo/term/GO:0032991) | protein-containing complex | 30 of 4701 | 1.99e-11 |
| [GO:0098796](http://amigo.geneontology.org/amigo/term/GO:0098796) | membrane protein complex | 15 of 1009 | 5.63e-09 |
| [GO:0098805](http://amigo.geneontology.org/amigo/term/GO:0098805) | whole membrane | 15 of 1300 | 1.44e-07 |
| [GO:0043229](http://amigo.geneontology.org/amigo/term/GO:0043229) | intracellular organelle | 36 of 10645 | 2.77e-07 |
| [GO:0005773](http://amigo.geneontology.org/amigo/term/GO:0005773) | vacuole | 10 of 519 | 6.97e-07 |
| [GO:0033178](http://amigo.geneontology.org/amigo/term/GO:0033178) | proton-transporting two-sector ATPase complex, catalytic domain | 4 of 14 | 7.24e-07 |
| [GO:0005902](http://amigo.geneontology.org/amigo/term/GO:0005902) | microvillus | 6 of 95 | 7.24e-07 |
| [GO:0044446](http://amigo.geneontology.org/amigo/term/GO:0044446) | intracellular organelle part | 30 of 7416 | 1.03e-06 |
| [GO:0016471](http://amigo.geneontology.org/amigo/term/GO:0016471) | vacuolar proton-transporting V-type ATPase complex | 4 of 16 | 1.03e-06 |
| [GO:0044424](http://amigo.geneontology.org/amigo/term/GO:0044424) | intracellular part | 37 of 12219 | 1.16e-06 |
| [GO:1990904](http://amigo.geneontology.org/amigo/term/GO:1990904) | ribonucleoprotein complex | 11 of 765 | 1.31e-06 |
| [GO:0044444](http://amigo.geneontology.org/amigo/term/GO:0044444) | cytoplasmic part | 30 of 7673 | 1.80e-06 |
| [GO:0005774](http://amigo.geneontology.org/amigo/term/GO:0005774) | vacuolar membrane | 7 of 239 | 3.53e-06 |
| [GO:0043227](http://amigo.geneontology.org/amigo/term/GO:0043227) | membrane-bounded organelle | 33 of 9775 | 3.83e-06 |
| [GO:0005886](http://amigo.geneontology.org/amigo/term/GO:0005886) | plasma membrane | 22 of 4328 | 5.92e-06 |
| [GO:0044464](http://amigo.geneontology.org/amigo/term/GO:0044464) | cell part | 38 of 14017 | 6.60e-06 |
| [GO:0033180](http://amigo.geneontology.org/amigo/term/GO:0033180) | proton-transporting V-type ATPase, V1 domain | 3 of 7 | 7.24e-06 |
| [GO:0005764](http://amigo.geneontology.org/amigo/term/GO:0005764) | lysosome | 8 of 422 | 7.97e-06 |
| [GO:0005829](http://amigo.geneontology.org/amigo/term/GO:0005829) | cytosol | 19 of 3326 | 8.29e-06 |
| [GO:0044425](http://amigo.geneontology.org/amigo/term/GO:0044425) | membrane part | 25 of 5857 | 1.02e-05 |
| [GO:0033179](http://amigo.geneontology.org/amigo/term/GO:0033179) | proton-transporting V-type ATPase, V0 domain | 3 of 9 | 1.06e-05 |
| [GO:0043231](http://amigo.geneontology.org/amigo/term/GO:0043231) | intracellular membrane-bounded organelle | 31 of 9088 | 1.17e-05 |
| [GO:0120025](http://amigo.geneontology.org/amigo/term/GO:0120025) | plasma membrane bounded cell projection | 15 of 2172 | 1.84e-05 |
| [GO:0043209](http://amigo.geneontology.org/amigo/term/GO:0043209) | myelin sheath | 6 of 212 | 1.87e-05 |
| [GO:0005737](http://amigo.geneontology.org/amigo/term/GO:0005737) | cytoplasm | 32 of 9909 | 1.87e-05 |
| [GO:0045121](http://amigo.geneontology.org/amigo/term/GO:0045121) | membrane raft | 7 of 374 | 3.19e-05 |
| [GO:0070013](http://amigo.geneontology.org/amigo/term/GO:0070013) | intracellular organelle lumen | 19 of 3882 | 6.20e-05 |
| [GO:0045177](http://amigo.geneontology.org/amigo/term/GO:0045177) | apical part of cell | 7 of 423 | 6.20e-05 |
| [GO:0042629](http://amigo.geneontology.org/amigo/term/GO:0042629) | mast cell granule | 3 of 23 | 8.39e-05 |
| [GO:0012505](http://amigo.geneontology.org/amigo/term/GO:0012505) | endomembrane system | 18 of 3670 | 0.00011 |
| [GO:0044428](http://amigo.geneontology.org/amigo/term/GO:0044428) | nuclear part | 18 of 3798 | 0.00016 |
| [GO:0031410](http://amigo.geneontology.org/amigo/term/GO:0031410) | cytoplasmic vesicle | 12 of 1710 | 0.00016 |
| [GO:0044459](http://amigo.geneontology.org/amigo/term/GO:0044459) | plasma membrane part | 14 of 2362 | 0.00017 |
| [GO:0042470](http://amigo.geneontology.org/amigo/term/GO:0042470) | melanosome | 4 of 103 | 0.00021 |
| [GO:0005634](http://amigo.geneontology.org/amigo/term/GO:0005634) | nucleus | 23 of 6086 | 0.00022 |
| [GO:0022626](http://amigo.geneontology.org/amigo/term/GO:0022626) | cytosolic ribosome | 4 of 107 | 0.00023 |
| [GO:0000220](http://amigo.geneontology.org/amigo/term/GO:0000220) | vacuolar proton-transporting V-type ATPase, V0 domain | 2 of 5 | 0.00033 |
| [GO:0031981](http://amigo.geneontology.org/amigo/term/GO:0031981) | nuclear lumen | 16 of 3386 | 0.00053 |
| [GO:0043232](http://amigo.geneontology.org/amigo/term/GO:0043232) | intracellular non-membrane-bounded organelle | 17 of 3809 | 0.00056 |
| [GO:0022625](http://amigo.geneontology.org/amigo/term/GO:0022625) | cytosolic large ribosomal subunit | 3 of 59 | 0.00084 |
| [GO:0030688](http://amigo.geneontology.org/amigo/term/GO:0030688) | preribosome, small subunit precursor | 2 of 10 | 0.00092 |
| [GO:0031225](http://amigo.geneontology.org/amigo/term/GO:0031225) | anchored component of membrane | 4 of 163 | 0.00096 |
| [GO:0098552](http://amigo.geneontology.org/amigo/term/GO:0098552) | side of membrane | 6 of 514 | 0.0013 |
| [GO:0009898](http://amigo.geneontology.org/amigo/term/GO:0009898) | cytoplasmic side of plasma membrane | 4 of 177 | 0.0013 |
| [GO:0044391](http://amigo.geneontology.org/amigo/term/GO:0044391) | ribosomal subunit | 4 of 181 | 0.0014 |
| [GO:0016324](http://amigo.geneontology.org/amigo/term/GO:0016324) | apical plasma membrane | 5 of 339 | 0.0014 |
| [GO:0005765](http://amigo.geneontology.org/amigo/term/GO:0005765) | lysosomal membrane | 4 of 186 | 0.0015 |
| [GO:0031234](http://amigo.geneontology.org/amigo/term/GO:0031234) | extrinsic component of cytoplasmic side of plasma membrane | 3 of 111 | 0.0042 |
| [GO:0098588](http://amigo.geneontology.org/amigo/term/GO:0098588) | bounding membrane of organelle | 9 of 1513 | 0.0043 |
| [GO:0120038](http://amigo.geneontology.org/amigo/term/GO:0120038) | plasma membrane bounded cell projection part | 9 of 1532 | 0.0045 |
| [GO:0098563](http://amigo.geneontology.org/amigo/term/GO:0098563) | intrinsic component of synaptic vesicle membrane | 2 of 28 | 0.0047 |
| [GO:0005834](http://amigo.geneontology.org/amigo/term/GO:0005834) | heterotrimeric G-protein complex | 2 of 31 | 0.0056 |
| [GO:0043235](http://amigo.geneontology.org/amigo/term/GO:0043235) | receptor complex | 4 of 302 | 0.0072 |
| [GO:0031090](http://amigo.geneontology.org/amigo/term/GO:0031090) | organelle membrane | 12 of 2740 | 0.0073 |
| [GO:0099503](http://amigo.geneontology.org/amigo/term/GO:0099503) | secretory vesicle | 5 of 525 | 0.0079 |
| [GO:0005768](http://amigo.geneontology.org/amigo/term/GO:0005768) | endosome | 6 of 779 | 0.0081 |
| [GO:0044456](http://amigo.geneontology.org/amigo/term/GO:0044456) | synapse part | 6 of 809 | 0.0096 |
| [GO:0031256](http://amigo.geneontology.org/amigo/term/GO:0031256) | leading edge membrane | 3 of 161 | 0.0101 |
| [GO:0098590](http://amigo.geneontology.org/amigo/term/GO:0098590) | plasma membrane region | 7 of 1115 | 0.0104 |
| [GO:0030496](http://amigo.geneontology.org/amigo/term/GO:0030496) | midbody | 3 of 165 | 0.0104 |
| [GO:0016607](http://amigo.geneontology.org/amigo/term/GO:0016607) | nuclear speck | 4 of 362 | 0.0123 |
| [GO:0005681](http://amigo.geneontology.org/amigo/term/GO:0005681) | spliceosomal complex | 3 of 177 | 0.0124 |
| [GO:0036464](http://amigo.geneontology.org/amigo/term/GO:0036464) | cytoplasmic ribonucleoprotein granule | 3 of 182 | 0.0132 |
| [GO:0008021](http://amigo.geneontology.org/amigo/term/GO:0008021) | synaptic vesicle | 3 of 183 | 0.0132 |
| [GO:0005730](http://amigo.geneontology.org/amigo/term/GO:0005730) | nucleolus | 6 of 878 | 0.0132 |
| [GO:0043679](http://amigo.geneontology.org/amigo/term/GO:0043679) | axon terminus | 3 of 188 | 0.0140 |
| [GO:0031252](http://amigo.geneontology.org/amigo/term/GO:0031252) | cell leading edge | 4 of 393 | 0.0154 |
| [GO:0005856](http://amigo.geneontology.org/amigo/term/GO:0005856) | cytoskeleton | 9 of 1933 | 0.0167 |
| [GO:1902494](http://amigo.geneontology.org/amigo/term/GO:1902494) | catalytic complex | 7 of 1266 | 0.0179 |
| [GO:0098793](http://amigo.geneontology.org/amigo/term/GO:0098793) | presynapse | 4 of 429 | 0.0194 |
| [GO:0005844](http://amigo.geneontology.org/amigo/term/GO:0005844) | polysome | 2 of 71 | 0.0204 |
| [GO:0005794](http://amigo.geneontology.org/amigo/term/GO:0005794) | Golgi apparatus | 7 of 1313 | 0.0206 |
| [GO:0005770](http://amigo.geneontology.org/amigo/term/GO:0005770) | late endosome | 3 of 225 | 0.0206 |
| [GO:0098797](http://amigo.geneontology.org/amigo/term/GO:0098797) | plasma membrane protein complex | 4 of 461 | 0.0237 |
| [GO:0097458](http://amigo.geneontology.org/amigo/term/GO:0097458) | neuron part | 8 of 1732 | 0.0260 |
| [GO:0005758](http://amigo.geneontology.org/amigo/term/GO:0005758) | mitochondrial intermembrane space | 2 of 83 | 0.0260 |
| [GO:0005913](http://amigo.geneontology.org/amigo/term/GO:0005913) | cell-cell adherens junction | 2 of 84 | 0.0261 |
| [GO:0030863](http://amigo.geneontology.org/amigo/term/GO:0030863) | cortical cytoskeleton | 2 of 85 | 0.0264 |
| [GO:0005813](http://amigo.geneontology.org/amigo/term/GO:0005813) | centrosome | 4 of 481 | 0.0264 |
| [GO:0009986](http://amigo.geneontology.org/amigo/term/GO:0009986) | cell surface | 5 of 796 | 0.0315 |
| [GO:0005912](http://amigo.geneontology.org/amigo/term/GO:0005912) | adherens junction | 3 of 276 | 0.0316 |
| [GO:0044430](http://amigo.geneontology.org/amigo/term/GO:0044430) | cytoskeletal part | 7 of 1460 | 0.0317 |
| [GO:0005654](http://amigo.geneontology.org/amigo/term/GO:0005654) | nucleoplasm | 10 of 2648 | 0.0352 |
| [GO:0031902](http://amigo.geneontology.org/amigo/term/GO:0031902) | late endosome membrane | 2 of 115 | 0.0420 |
| [GO:0031300](http://amigo.geneontology.org/amigo/term/GO:0031300) | intrinsic component of organelle membrane | 3 of 312 | 0.0420 |
| [GO:0043195](http://amigo.geneontology.org/amigo/term/GO:0043195) | terminal bouton | 2 of 116 | 0.0423 |
| [GO:0031253](http://amigo.geneontology.org/amigo/term/GO:0031253) | cell projection membrane | 3 of 316 | 0.0423 |

**Reference publications**

| *publication* | *(year) title* | *count in gene set* | *false discovery rate* |
| --- | --- | --- | --- |
| [PMID:32194992](https://www.ncbi.nlm.nih.gov/pubmed/32194992) | (2020) TFEB-mediated lysosomal biogenesis and lysosomal drug sequestration confer resistance to MEK inhibition in pancreatic cancer. | 5 of 20 | 9.58e-05 |
| [PMID:30395881](https://www.ncbi.nlm.nih.gov/pubmed/30395881) | (2019) The protein interaction networks of mucolipins and two-pore channels. | 7 of 105 | 9.58e-05 |
| [PMID:30555553](https://www.ncbi.nlm.nih.gov/pubmed/30555553) | (2018) V-ATPases and osteoclasts: ambiguous future of V-ATPases inhibitors in osteoporosis. | 6 of 50 | 9.58e-05 |
| [PMID:27519690](https://www.ncbi.nlm.nih.gov/pubmed/27519690) | (2016) Systematic identification of genes involved in metabolic acid stress resistance in yeast and their potential as cancer targets. | 5 of 22 | 9.58e-05 |
| [PMID:26442671](https://www.ncbi.nlm.nih.gov/pubmed/26442671) | (2015) Mapping the H(+) (V)-ATPase interactome: identification of proteins involved in trafficking, folding, assembly and phosphorylation. | 6 of 51 | 9.58e-05 |
| [PMID:25948753](https://www.ncbi.nlm.nih.gov/pubmed/25948753) | (2015) Regulation of lipid droplet dynamics in Saccharomyces cerevisiae depends on the Rab7-like Ypt7p, HOPS complex and V1-ATPase. | 5 of 29 | 9.58e-05 |
| [PMID:24575049](https://www.ncbi.nlm.nih.gov/pubmed/24575049) | (2014) Role of the bicarbonate-responsive soluble adenylyl cyclase in pH sensing and metabolic regulation. | 5 of 29 | 9.58e-05 |
| [PMID:29900055](https://www.ncbi.nlm.nih.gov/pubmed/29900055) | (2018) pH regulators to target the tumor immune microenvironment in human hepatocellular carcinoma. | 5 of 33 | 0.00012 |
| [PMID:29422602](https://www.ncbi.nlm.nih.gov/pubmed/29422602) | (2018) Acidic organelles mediate TGF-Beta1-induced cellular fibrosis via (pro)renin receptor and vacuolar ATPase trafficking in human peritoneal mesothelial cells. | 5 of 34 | 0.00013 |
| [PMID:20418956](https://www.ncbi.nlm.nih.gov/pubmed/20418956) | (2010) High-content, image-based screening for drug targets in yeast. | 4 of 10 | 0.00013 |
| [PMID:23967163](https://www.ncbi.nlm.nih.gov/pubmed/23967163) | (2013) Elucidation of how cancer cells avoid acidosis through comparative transcriptomic data analysis. | 5 of 39 | 0.00019 |
| [PMID:30478388](https://www.ncbi.nlm.nih.gov/pubmed/30478388) | (2018) The interferon-inducible isoform of NCOA7 inhibits endosome-mediated viral entry. | 5 of 43 | 0.00028 |
| [PMID:29473670](https://www.ncbi.nlm.nih.gov/pubmed/29473670) | (2018) Some assembly required: Contributions of Tom Stevens' lab to the V-ATPase field. | 4 of 15 | 0.00038 |
| [PMID:24155661](https://www.ncbi.nlm.nih.gov/pubmed/24155661) | (2013) Silencing of atp6v1c1 prevents breast cancer growth and bone metastasis. | 4 of 15 | 0.00038 |
| [PMID:25866880](https://www.ncbi.nlm.nih.gov/pubmed/25866880) | (2015) Translational control of the cytosolic stress response by mitochondrial ribosomal protein L18. | 4 of 16 | 0.00041 |
| [PMID:24314139](https://www.ncbi.nlm.nih.gov/pubmed/24314139) | (2013) Reprogramming of lysosomal gene expression by interleukin-4 and Stat6. | 6 of 105 | 0.00041 |
| [PMID:32102213](https://www.ncbi.nlm.nih.gov/pubmed/32102213) | (2020) Augmenting Vacuolar H+-ATPase Function Prevents Cardiomyocytes from Lipid-Overload Induced Dysfunction. | 4 of 18 | 0.00043 |
| [PMID:31033440](https://www.ncbi.nlm.nih.gov/pubmed/31033440) | (2019) HRI coordinates translation necessary for protein homeostasis and mitochondrial function in erythropoiesis. | 5 of 53 | 0.00043 |
| [PMID:30952843](https://www.ncbi.nlm.nih.gov/pubmed/30952843) | (2019) FoxK1 and FoxK2 in insulin regulation of cellular and mitochondrial metabolism. | 6 of 113 | 0.00043 |
| [PMID:30894069](https://www.ncbi.nlm.nih.gov/pubmed/30894069) | (2019) Impaired TFEB-mediated lysosomal biogenesis promotes the development of pancreatitis in mice and is associated with human pancreatitis. | 4 of 17 | 0.00043 |
| [PMID:30647105](https://www.ncbi.nlm.nih.gov/pubmed/30647105) | (2019) Comparative Genomic Screen in Two Yeasts Reveals Conserved Pathways in the Response Network to Phenol Stress. | 4 of 18 | 0.00043 |
| [PMID:28594408](https://www.ncbi.nlm.nih.gov/pubmed/28594408) | (2017) Autophagy blockade and lysosomal membrane permeabilization contribute to lead-induced nephrotoxicity in primary rat proximal tubular cells. | 4 of 17 | 0.00043 |
| [PMID:28290485](https://www.ncbi.nlm.nih.gov/pubmed/28290485) | (2017) Complementary transcriptomic and proteomic analyses reveal regulatory mechanisms of milk protein production in dairy cows consuming different forages. | 5 of 51 | 0.00043 |
| [PMID:28053225](https://www.ncbi.nlm.nih.gov/pubmed/28053225) | (2017) The V-ATPase is expressed in the choroid plexus and mediates cAMP-induced intracellular pH alterations. | 4 of 17 | 0.00043 |
| [PMID:26586472](https://www.ncbi.nlm.nih.gov/pubmed/26586472) | (2016) V-type ATPase proton pump expression during enamel formation. | 4 of 19 | 0.00043 |
| [PMID:24223829](https://www.ncbi.nlm.nih.gov/pubmed/24223829) | (2013) The role of individual domains and the significance of shedding of ATP6AP2(pro)renin receptor in vacuolar H(+)-ATPase biogenesis. | 4 of 17 | 0.00043 |
| [PMID:23469216](https://www.ncbi.nlm.nih.gov/pubmed/23469216) | (2013) Distinct signal transduction pathways downstream of the (P)RR revealed by microarray and ChIP-chip analyses. | 5 of 58 | 0.00043 |
| [PMID:22467241](https://www.ncbi.nlm.nih.gov/pubmed/22467241) | (2012) V-ATPase subunit ATP6AP1 (Ac45) regulates osteoclast differentiation, extracellular acidification, lysosomal trafficking, and protease exocytosis in osteoclast-mediated bone resorption. | 4 of 19 | 0.00043 |
| [PMID:21804531](https://www.ncbi.nlm.nih.gov/pubmed/21804531) | (2011) Regulation of TFEB and V-ATPases by mTORC1. | 4 of 19 | 0.00043 |
| [PMID:20137074](https://www.ncbi.nlm.nih.gov/pubmed/20137074) | (2010) Identification of arginine- and lysine-methylation in the proteome of Saccharomyces cerevisiae and its functional implications. | 4 of 18 | 0.00043 |
| [PMID:11922865](https://www.ncbi.nlm.nih.gov/pubmed/11922865) | (2002) Nerve growth factor selectively regulates expression of transcripts encoding ribosomal proteins. | 4 of 19 | 0.00043 |
| [PMID:25133973](https://www.ncbi.nlm.nih.gov/pubmed/25133973) | (2014) Nuclear cytoplasmic trafficking of proteins is a major response of human fibroblasts to oxidative stress. | 6 of 123 | 0.00048 |
| [PMID:23724114](https://www.ncbi.nlm.nih.gov/pubmed/23724114) | (2013) Functional analyse of GLUT1 and GLUT12 in glucose uptake in goat mammary gland epithelial cells. | 4 of 21 | 0.00048 |
| [PMID:30875964](https://www.ncbi.nlm.nih.gov/pubmed/30875964) | (2019) Niclosamide Triggers Non-Canonical LC3 Lipidation. | 4 of 22 | 0.00055 |
| [PMID:31523176](https://www.ncbi.nlm.nih.gov/pubmed/31523176) | (2019) Ribosomal Protein L15 is involved in Colon Carcinogenesis. | 4 of 23 | 0.00063 |
| [PMID:25332393](https://www.ncbi.nlm.nih.gov/pubmed/25332393) | (2014) Ribosomal stress activates eEF2K-eEF2 pathway causing translation elongation inhibition and recruitment of terminal oligopyrimidine (TOP) mRNAs on polysomes. | 4 of 23 | 0.00063 |
| [PMID:30307711](https://www.ncbi.nlm.nih.gov/pubmed/30307711) | (2018) Quantitative proteomic analysis of intracerebral hemorrhage in rats with a focus on brain energy metabolism. | 4 of 26 | 0.00092 |
| [PMID:22194971](https://www.ncbi.nlm.nih.gov/pubmed/22194971) | (2011) Protein profile changes during porcine oocyte aging and effects of caffeine on protein expression patterns. | 4 of 26 | 0.00092 |
| [PMID:26319900](https://www.ncbi.nlm.nih.gov/pubmed/26319900) | (2015) Suppression of the GTPase-activating protein RGS10 increases Rheb-GTP and mTOR signaling in ovarian cancer cells. | 4 of 27 | 0.0010 |
| [PMID:23690912](https://www.ncbi.nlm.nih.gov/pubmed/23690912) | (2013) New model of action for mood stabilizers: phosphoproteome from rat pre-frontal cortex synaptoneurosomal preparations. | 4 of 27 | 0.0010 |
| [PMID:22829766](https://www.ncbi.nlm.nih.gov/pubmed/22829766) | (2012) A cytotoxic type III secretion effector of Vibrio parahaemolyticus targets vacuolar H+-ATPase subunit c and ruptures host cell lysosomes. | 4 of 27 | 0.0010 |
| [PMID:31189779](https://www.ncbi.nlm.nih.gov/pubmed/31189779) | (2019) Vacuolar-type ATPase: A proton pump to lysosomal trafficking. | 4 of 28 | 0.0011 |
| [PMID:29980615](https://www.ncbi.nlm.nih.gov/pubmed/29980615) | (2018) Influenza A Virus Induces Autophagosomal Targeting of Ribosomal Proteins. | 4 of 29 | 0.0011 |
| [PMID:25951193](https://www.ncbi.nlm.nih.gov/pubmed/25951193) | (2015) The integral membrane protein ITM2A, a transcriptional target of PKA-CREB, regulates autophagic flux via interaction with the vacuolar ATPase. | 4 of 28 | 0.0011 |
| [PMID:23696868](https://www.ncbi.nlm.nih.gov/pubmed/23696868) | (2013) Enhanced translation of mRNAs encoding proteins involved in mRNA translation during recovery from heat shock. | 4 of 28 | 0.0011 |
| [PMID:23077530](https://www.ncbi.nlm.nih.gov/pubmed/23077530) | (2012) Suppressed RNA-polymerase 1 pathway is associated with benign multiple sclerosis. | 4 of 30 | 0.0013 |
| [PMID:18667600](https://www.ncbi.nlm.nih.gov/pubmed/18667600) | (2008) V-ATPase expression in the mouse olfactory epithelium. | 3 of 6 | 0.0014 |
| [PMID:31285595](https://www.ncbi.nlm.nih.gov/pubmed/31285595) | (2019) Covalent targeting of the vacuolar H+-ATPase activates autophagy via mTORC1 inhibition. | 4 of 32 | 0.0015 |
| [PMID:30804932](https://www.ncbi.nlm.nih.gov/pubmed/30804932) | (2019) New Insights for RANKL as a Proinflammatory Modulator in Modeled Inflammatory Arthritis. | 4 of 32 | 0.0015 |
| [PMID:26151086](https://www.ncbi.nlm.nih.gov/pubmed/26151086) | (2015) Compensatory Islet Response to Insulin Resistance Revealed by Quantitative Proteomics. | 5 of 85 | 0.0016 |
| [PMID:31201651](https://www.ncbi.nlm.nih.gov/pubmed/31201651) | (2019) Altered Levels of Proteins and Phosphoproteins, in the Absence of Early Causative Transcriptional Changes, Shape the Molecular Pathogenesis in the Brain of Young Presymptomatic Ki91 SCA3MJD Mouse. | 7 of 277 | 0.0018 |
| [PMID:28253842](https://www.ncbi.nlm.nih.gov/pubmed/28253842) | (2017) Salicylic acid-related cotton (Gossypium arboreum) ribosomal protein GaRPL18 contributes to resistance to Verticillium dahliae. | 3 of 7 | 0.0018 |
| [PMID:26901847](https://www.ncbi.nlm.nih.gov/pubmed/26901847) | (2016) Radiotherapy diagnostic biomarkers in radioresistant human H460 lung cancer stem-like cells. | 4 of 34 | 0.0018 |
| [PMID:28994389](https://www.ncbi.nlm.nih.gov/pubmed/28994389) | (2017) Molecular architecture underlying fluid absorption by the developing inner ear. | 5 of 93 | 0.0023 |
| [PMID:25885223](https://www.ncbi.nlm.nih.gov/pubmed/25885223) | (2015) Endoplasmic reticulum chaperone GRP78 is involved in autophagy activation induced by ischemic preconditioning in neural cells. | 3 of 8 | 0.0023 |
| [PMID:25496664](https://www.ncbi.nlm.nih.gov/pubmed/25496664) | (2014) Molecular signatures that correlate with induction of lens regeneration in newts: lessons from proteomic analysis. | 5 of 93 | 0.0023 |
| [PMID:24284395](https://www.ncbi.nlm.nih.gov/pubmed/24284395) | (2013) Combination BMSC and Niaspan treatment of stroke enhances white matter remodeling and synaptic protein expression in diabetic rats. | 3 of 8 | 0.0023 |
| [PMID:31920959](https://www.ncbi.nlm.nih.gov/pubmed/31920959) | (2019) Multiomics-Based Signaling Pathway Network Alterations in Human Non-functional Pituitary Adenomas. | 6 of 186 | 0.0027 |
| [PMID:26792401](https://www.ncbi.nlm.nih.gov/pubmed/26792401) | (2016) CNS uptake of bortezomib is enhanced by P-glycoprotein inhibition: implications for spinal muscular atrophy. | 3 of 9 | 0.0029 |
| [PMID:24899231](https://www.ncbi.nlm.nih.gov/pubmed/24899231) | (2014) Conditional disruption of interactions between GAlfai2 and regulator of G protein signaling (RGS) proteins protects the heart from ischemic injury. | 3 of 9 | 0.0029 |
| [PMID:30120234](https://www.ncbi.nlm.nih.gov/pubmed/30120234) | (2018) Galectin-9 suppresses B cell receptor signaling and is regulated by I-branching of N-glycans. | 4 of 41 | 0.0030 |
| [PMID:19302708](https://www.ncbi.nlm.nih.gov/pubmed/19302708) | (2009) Transcriptional signatures of BALBc mouse macrophages housing multiplying Leishmania amazonensis amastigotes. | 5 of 102 | 0.0031 |
| [PMID:31296888](https://www.ncbi.nlm.nih.gov/pubmed/31296888) | (2019) A Differential Hypofunctionality of GAlfai Proteins Occurs in Adolescent Idiopathic Scoliosis and Correlates with the Risk of Disease Progression. | 3 of 10 | 0.0035 |
| [PMID:31149036](https://www.ncbi.nlm.nih.gov/pubmed/31149036) | (2019) GRP78-targeted ferritin nanocaged ultra-high dose of doxorubicin for hepatocellular carcinoma therapy. | 3 of 10 | 0.0035 |
| [PMID:30201806](https://www.ncbi.nlm.nih.gov/pubmed/30201806) | (2018) RACK1 Specifically Regulates Translation through Its Binding to Ribosomes. | 3 of 10 | 0.0035 |
| [PMID:30189184](https://www.ncbi.nlm.nih.gov/pubmed/30189184) | (2018) Role of hippocampal 5-HT1A receptors in the antidepressant-like phenotype of mice expressing RGS-insensitive GAlfai2 protein. | 3 of 10 | 0.0035 |
| [PMID:25993305](https://www.ncbi.nlm.nih.gov/pubmed/25993305) | (2015) MALDI-Mass Spectrometric Imaging Revealing Hypoxia-Driven Lipids and Proteins in a Breast Tumor Model. | 4 of 44 | 0.0035 |
| [PMID:24858945](https://www.ncbi.nlm.nih.gov/pubmed/24858945) | (2014) GAlfai2- and GAlfai3-deficient mice display opposite severity of myocardial ischemia reperfusion injury. | 3 of 10 | 0.0035 |
| [PMID:23434374](https://www.ncbi.nlm.nih.gov/pubmed/23434374) | (2013) ZKSCAN3 is a master transcriptional repressor of autophagy. | 4 of 43 | 0.0035 |
| [PMID:21524776](https://www.ncbi.nlm.nih.gov/pubmed/21524776) | (2011) Subcellular location and topology of severe acute respiratory syndrome coronavirus envelope protein. | 3 of 10 | 0.0035 |
| [PMID:21276872](https://www.ncbi.nlm.nih.gov/pubmed/21276872) | (2011) Uncovering the global host cell requirements for influenza virus replication via RNAi screening. | 4 of 45 | 0.0036 |
| [PMID:31223620](https://www.ncbi.nlm.nih.gov/pubmed/31223620) | (2019) Validation of Reference Genes for Gene Expression Normalization in RAW264.7 Cells under Different Conditions. | 3 of 11 | 0.0039 |
| [PMID:30442709](https://www.ncbi.nlm.nih.gov/pubmed/30442709) | (2019) PPT1 Promotes Tumor Growth and Is the Molecular Target of Chloroquine Derivatives in Cancer. | 3 of 11 | 0.0039 |
| [PMID:30009162](https://www.ncbi.nlm.nih.gov/pubmed/30009162) | (2018) Topological Characterization of Human and Mouse m5C Epitranscriptome Revealed by Bisulfite Sequencing. | 4 of 46 | 0.0039 |
| [PMID:29203145](https://www.ncbi.nlm.nih.gov/pubmed/29203145) | (2018) Global gene expression analysis of macrophage response induced by nonporous and porous silica nanoparticles. | 3 of 11 | 0.0039 |
| [PMID:27790248](https://www.ncbi.nlm.nih.gov/pubmed/27790248) | (2016) Large Scale Gene Expression Meta-Analysis Reveals Tissue-Specific, Sex-Biased Gene Expression in Humans. | 4 of 47 | 0.0039 |
| [PMID:27278128](https://www.ncbi.nlm.nih.gov/pubmed/27278128) | (2016) Phorbol ester-mediated re-expression of endogenous LAT adapter in J.CaM2 cells: a model for dissecting drivers and blockers of LAT transcription. | 3 of 11 | 0.0039 |
| [PMID:26075749](https://www.ncbi.nlm.nih.gov/pubmed/26075749) | (2015) Metformin induces ER stress-dependent apoptosis through miR-708-5pNNAT pathway in prostate cancer. | 3 of 11 | 0.0039 |
| [PMID:25050110](https://www.ncbi.nlm.nih.gov/pubmed/25050110) | (2014) GRP78 inhibits macrophage adhesion via SR-A. | 3 of 11 | 0.0039 |
| [PMID:19008951](https://www.ncbi.nlm.nih.gov/pubmed/19008951) | (2008) Key role of splenic myeloid DCs in the IFN-alphabeta response to adenoviruses in vivo. | 4 of 46 | 0.0039 |
| [PMID:18638456](https://www.ncbi.nlm.nih.gov/pubmed/18638456) | (2008) AMPK represses TOP mRNA translation but not global protein synthesis in liver. | 3 of 11 | 0.0039 |
| [PMID:30975297](https://www.ncbi.nlm.nih.gov/pubmed/30975297) | (2019) Cardioprotective Effect of the Mitochondrial Unfolded Protein Response During Chronic Pressure Overload. | 3 of 12 | 0.0043 |
| [PMID:30453691](https://www.ncbi.nlm.nih.gov/pubmed/30453691) | (2018) A Proteomic Approach for Understanding the Mechanisms of Delayed Corneal Wound Healing in Diabetic Keratopathy Using Diabetic Model Rat. | 3 of 12 | 0.0043 |
| [PMID:27835895](https://www.ncbi.nlm.nih.gov/pubmed/27835895) | (2016) Enhancement of 5-FU sensitivity by the proapoptotic rpL3 gene in p53 null colon cancer cells through combined polymer nanoparticles. | 3 of 12 | 0.0043 |
| [PMID:27016735](https://www.ncbi.nlm.nih.gov/pubmed/27016735) | (2016) Promoter architecture and transcriptional regulation of Abf1-dependent ribosomal protein genes in Saccharomyces cerevisiae. | 3 of 12 | 0.0043 |
| [PMID:25298750](https://www.ncbi.nlm.nih.gov/pubmed/25298750) | (2014) Human umbilical cord mesenchymal stem cells promote carcinoma growth and lymph node metastasis when co-injected with esophageal carcinoma cells in nude mice. | 3 of 12 | 0.0043 |
| [PMID:24828841](https://www.ncbi.nlm.nih.gov/pubmed/24828841) | (2014) Porcine endogenous retroviruses in xenotransplantation--molecular aspects. | 3 of 12 | 0.0043 |
| [PMID:22065581](https://www.ncbi.nlm.nih.gov/pubmed/22065581) | (2011) Human mutation within Per-Arnt-Sim (PAS) domain-containing protein kinase (PASK) causes basal insulin hypersecretion. | 3 of 12 | 0.0043 |
| [PMID:27078027](https://www.ncbi.nlm.nih.gov/pubmed/27078027) | (2016) Hypoxia Induces Autophagy through Translational Up-Regulation of Lysosomal Proteins in Human Colon Cancer Cells. | 4 of 51 | 0.0046 |
| [PMID:30708974](https://www.ncbi.nlm.nih.gov/pubmed/30708974) | (2019) TUDCA-Treated Mesenchymal Stem Cells Protect against ER Stress in the Hippocampus of a Murine Chronic Kidney Disease Model. | 3 of 13 | 0.0048 |
| [PMID:30135222](https://www.ncbi.nlm.nih.gov/pubmed/30135222) | (2018) Spleen Tyrosine Kinase Inhibitor TAK-659 Prevents Splenomegaly and Tumor Development in a Murine Model of Epstein-Barr Virus-Associated Lymphoma. | 3 of 13 | 0.0048 |
| [PMID:29384874](https://www.ncbi.nlm.nih.gov/pubmed/29384874) | (2018) BMP2 and VEGF165 transfection to bone marrow stromal stem cells regulate osteogenic potential in vitro. | 3 of 13 | 0.0048 |
| [PMID:27237224](https://www.ncbi.nlm.nih.gov/pubmed/27237224) | (2016) Adaptations to chronic rapamycin in mice. | 3 of 13 | 0.0048 |
| [PMID:23691483](https://www.ncbi.nlm.nih.gov/pubmed/23691483) | (2012) GNAI1 Suppresses Tumor Cell Migration and Invasion and is Post-Transcriptionally Regulated by Mir-320acd in Hepatocellular Carcinoma. | 3 of 13 | 0.0048 |
| [PMID:20126274](https://www.ncbi.nlm.nih.gov/pubmed/20126274) | (2010) An inhibitory role of the G-protein regulator AGS3 in mTOR-dependent macroautophagy. | 3 of 13 | 0.0048 |
| [PMID:30260431](https://www.ncbi.nlm.nih.gov/pubmed/30260431) | (2018) Protein Syndesmos is a novel RNA-binding protein that regulates primary cilia formation. | 4 of 54 | 0.0053 |
| [PMID:29499948](https://www.ncbi.nlm.nih.gov/pubmed/29499948) | (2018) Endogenous Cellular MicroRNAs Mediate Antiviral Defense against Influenza A Virus. | 3 of 14 | 0.0054 |
| [PMID:29054531](https://www.ncbi.nlm.nih.gov/pubmed/29054531) | (2018) The ammonia transporter RhCG modulates urinary acidification by interacting with the vacuolar proton-ATPases in renal intercalated cells. | 3 of 14 | 0.0054 |
| [PMID:27941876](https://www.ncbi.nlm.nih.gov/pubmed/27941876) | (2017) Inhibition of KPNA4 attenuates prostate cancer metastasis. | 3 of 14 | 0.0054 |
| [PMID:27001958](https://www.ncbi.nlm.nih.gov/pubmed/27001958) | (2016) Cutting Edge: Foxp1 Controls Naive CD8+ T Cell Quiescence by Simultaneously Repressing Key Pathways in Cellular Metabolism and Cell Cycle Progression. | 3 of 14 | 0.0054 |

**KEGG Pathways**

| *pathway* | *description* | *count in gene set* | *false discovery rate* |
| --- | --- | --- | --- |
| [mmu04966](https://www.kegg.jp/kegg-bin/show_pathway?mmu04966) | Collecting duct acid secretion | 8 of 27 | 1.06e-13 |
| [mmu04721](https://www.kegg.jp/kegg-bin/show_pathway?mmu04721) | Synaptic vesicle cycle | 8 of 62 | 2.03e-11 |
| [mmu04145](https://www.kegg.jp/kegg-bin/show_pathway?mmu04145) | Phagosome | 10 of 165 | 2.03e-11 |
| [mmu05323](https://www.kegg.jp/kegg-bin/show_pathway?mmu05323) | Rheumatoid arthritis | 8 of 81 | 7.42e-11 |
| [mmu00190](https://www.kegg.jp/kegg-bin/show_pathway?mmu00190) | Oxidative phosphorylation | 8 of 129 | 1.98e-09 |
| [mmu04150](https://www.kegg.jp/kegg-bin/show_pathway?mmu04150) | mTOR signaling pathway | 6 of 152 | 5.78e-06 |
| [mmu04611](https://www.kegg.jp/kegg-bin/show_pathway?mmu04611) | Platelet activation | 5 of 122 | 4.33e-05 |
| [mmu01100](https://www.kegg.jp/kegg-bin/show_pathway?mmu01100) | Metabolic pathways | 10 of 1296 | 0.00088 |
| [mmu03010](https://www.kegg.jp/kegg-bin/show_pathway?mmu03010) | Ribosome | 4 of 128 | 0.0010 |
| [mmu04730](https://www.kegg.jp/kegg-bin/show_pathway?mmu04730) | Long-term depression | 3 of 60 | 0.0020 |
| [mmu05152](https://www.kegg.jp/kegg-bin/show_pathway?mmu05152) | Tuberculosis | 4 of 172 | 0.0025 |
| [mmu05133](https://www.kegg.jp/kegg-bin/show_pathway?mmu05133) | Pertussis | 3 of 74 | 0.0028 |
| [mmu04971](https://www.kegg.jp/kegg-bin/show_pathway?mmu04971) | Gastric acid secretion | 3 of 72 | 0.0028 |
| [mmu04670](https://www.kegg.jp/kegg-bin/show_pathway?mmu04670) | Leukocyte transendothelial migration | 3 of 115 | 0.0089 |
| [mmu04142](https://www.kegg.jp/kegg-bin/show_pathway?mmu04142) | Lysosome | 3 of 123 | 0.0101 |
| [mmu04371](https://www.kegg.jp/kegg-bin/show_pathway?mmu04371) | Apelin signaling pathway | 3 of 134 | 0.0120 |
| [mmu05012](https://www.kegg.jp/kegg-bin/show_pathway?mmu05012) | Parkinson's disease | 3 of 138 | 0.0123 |
| [mmu04921](https://www.kegg.jp/kegg-bin/show_pathway?mmu04921) | Oxytocin signaling pathway | 3 of 149 | 0.0143 |
| [mmu05030](https://www.kegg.jp/kegg-bin/show_pathway?mmu05030) | Cocaine addiction | 2 of 48 | 0.0197 |
| [mmu04062](https://www.kegg.jp/kegg-bin/show_pathway?mmu04062) | Chemokine signaling pathway | 3 of 179 | 0.0214 |
| [mmu04923](https://www.kegg.jp/kegg-bin/show_pathway?mmu04923) | Regulation of lipolysis in adipocytes | 2 of 55 | 0.0230 |
| [mmu05134](https://www.kegg.jp/kegg-bin/show_pathway?mmu05134) | Legionellosis | 2 of 57 | 0.0235 |
| [mmu05205](https://www.kegg.jp/kegg-bin/show_pathway?mmu05205) | Proteoglycans in cancer | 3 of 199 | 0.0249 |
| [mmu04015](https://www.kegg.jp/kegg-bin/show_pathway?mmu04015) | Rap1 signaling pathway | 3 of 207 | 0.0266 |
| [mmu04924](https://www.kegg.jp/kegg-bin/show_pathway?mmu04924) | Renin secretion | 2 of 69 | 0.0296 |
| [mmu03008](https://www.kegg.jp/kegg-bin/show_pathway?mmu03008) | Ribosome biogenesis in eukaryotes | 2 of 76 | 0.0342 |
| [mmu05132](https://www.kegg.jp/kegg-bin/show_pathway?mmu05132) | Salmonella infection | 2 of 78 | 0.0346 |
| [mmu04540](https://www.kegg.jp/kegg-bin/show_pathway?mmu04540) | Gap junction | 2 of 85 | 0.0391 |
| [mmu04727](https://www.kegg.jp/kegg-bin/show_pathway?mmu04727) | GABAergic synapse | 2 of 87 | 0.0395 |
| [mmu05032](https://www.kegg.jp/kegg-bin/show_pathway?mmu05032) | Morphine addiction | 2 of 91 | 0.0407 |
| [mmu04914](https://www.kegg.jp/kegg-bin/show_pathway?mmu04914) | Progesterone-mediated oocyte maturation | 2 of 90 | 0.0407 |
| [mmu04713](https://www.kegg.jp/kegg-bin/show_pathway?mmu04713) | Circadian entrainment | 2 of 95 | 0.0407 |
| [mmu04640](https://www.kegg.jp/kegg-bin/show_pathway?mmu04640) | Hematopoietic cell lineage | 2 of 90 | 0.0407 |
| [mmu04064](https://www.kegg.jp/kegg-bin/show_pathway?mmu04064) | NF-kappa B signaling pathway | 2 of 93 | 0.0407 |
| [mmu04916](https://www.kegg.jp/kegg-bin/show_pathway?mmu04916) | Melanogenesis | 2 of 98 | 0.0408 |
| [mmu05142](https://www.kegg.jp/kegg-bin/show_pathway?mmu05142) | Chagas disease (American trypanosomiasis) | 2 of 101 | 0.0420 |
| [mmu05145](https://www.kegg.jp/kegg-bin/show_pathway?mmu05145) | Toxoplasmosis | 2 of 107 | 0.0455 |
| [mmu04725](https://www.kegg.jp/kegg-bin/show_pathway?mmu04725) | Cholinergic synapse | 2 of 112 | 0.0482 |
| [mmu04724](https://www.kegg.jp/kegg-bin/show_pathway?mmu04724) | Glutamatergic synapse | 2 of 113 | 0.0482 |

**Reactome Pathways**

| *pathway* | *description* | *count in gene set* | *false discovery rate* |
| --- | --- | --- | --- |
| [MMU-77387](https://reactome.org/content/detail/R-MMU-77387) | Insulin receptor recycling | 8 of 27 | 1.67e-13 |
| [MMU-917977](https://reactome.org/content/detail/R-MMU-917977) | Transferrin endocytosis and recycling | 8 of 30 | 1.72e-13 |
| [MMU-1222556](https://reactome.org/content/detail/R-MMU-1222556) | ROS, RNS production in phagocytes | 8 of 32 | 1.80e-13 |
| [MMU-917937](https://reactome.org/content/detail/R-MMU-917937) | Iron uptake and transport | 8 of 50 | 3.30e-12 |
| [MMU-74752](https://reactome.org/content/detail/R-MMU-74752) | Signaling by Insulin receptor | 8 of 66 | 2.03e-11 |
| [MMU-983712](https://reactome.org/content/detail/R-MMU-983712) | Ion channel transport | 9 of 159 | 3.33e-10 |
| [MMU-168249](https://reactome.org/content/detail/R-MMU-168249) | Innate Immune System | 14 of 879 | 3.95e-09 |
| [MMU-9006934](https://reactome.org/content/detail/R-MMU-9006934) | Signaling by Receptor Tyrosine Kinases | 10 of 360 | 1.33e-08 |
| [MMU-168256](https://reactome.org/content/detail/R-MMU-168256) | Immune System | 16 of 1523 | 4.30e-08 |
| [MMU-382551](https://reactome.org/content/detail/R-MMU-382551) | Transport of small molecules | 9 of 624 | 2.02e-05 |
| [MMU-6798695](https://reactome.org/content/detail/R-MMU-6798695) | Neutrophil degranulation | 7 of 476 | 0.00029 |
| [MMU-162582](https://reactome.org/content/detail/R-MMU-162582) | Signal Transduction | 13 of 2430 | 0.0025 |
| [MMU-997269](https://reactome.org/content/detail/R-MMU-997269) | Inhibition of adenylate cyclase pathway | 2 of 12 | 0.0035 |
| [MMU-170670](https://reactome.org/content/detail/R-MMU-170670) | Adenylate cyclase inhibitory pathway | 2 of 12 | 0.0035 |
| [MMU-392170](https://reactome.org/content/detail/R-MMU-392170) | ADP signalling through P2Y purinoceptor 12 | 2 of 22 | 0.0091 |
| [MMU-202040](https://reactome.org/content/detail/R-MMU-202040) | G-protein activation | 2 of 27 | 0.0125 |
| [MMU-991365](https://reactome.org/content/detail/R-MMU-991365) | Activation of GABAB receptors | 2 of 35 | 0.0162 |
| [MMU-977444](https://reactome.org/content/detail/R-MMU-977444) | GABA B receptor activation | 2 of 35 | 0.0162 |
| [MMU-5674135](https://reactome.org/content/detail/R-MMU-5674135) | MAP2K and MAPK activation | 2 of 36 | 0.0162 |
| [MMU-3928662](https://reactome.org/content/detail/R-MMU-3928662) | EPHB-mediated forward signaling | 2 of 34 | 0.0162 |
| [MMU-392518](https://reactome.org/content/detail/R-MMU-392518) | Signal amplification | 2 of 32 | 0.0162 |
| [MMU-112043](https://reactome.org/content/detail/R-MMU-112043) | PLC beta mediated events | 2 of 32 | 0.0162 |
| [MMU-112040](https://reactome.org/content/detail/R-MMU-112040) | G-protein mediated events | 2 of 33 | 0.0162 |
| [MMU-6814122](https://reactome.org/content/detail/R-MMU-6814122) | Cooperation of PDCL (PhLP1) and TRiC/CCT in G-protein beta folding | 2 of 39 | 0.0166 |
| [MMU-391251](https://reactome.org/content/detail/R-MMU-391251) | Protein folding | 2 of 40 | 0.0167 |
| [MMU-390466](https://reactome.org/content/detail/R-MMU-390466) | Chaperonin-mediated protein folding | 2 of 40 | 0.0167 |
| [MMU-5250924](https://reactome.org/content/detail/R-MMU-5250924) | B-WICH complex positively regulates rRNA expression | 2 of 48 | 0.0218 |
| [MMU-5250913](https://reactome.org/content/detail/R-MMU-5250913) | Positive epigenetic regulation of rRNA expression | 2 of 48 | 0.0218 |
| [MMU-977443](https://reactome.org/content/detail/R-MMU-977443) | GABA receptor activation | 2 of 51 | 0.0228 |
| [MMU-2682334](https://reactome.org/content/detail/R-MMU-2682334) | EPH-Ephrin signaling | 2 of 63 | 0.0328 |
| [MMU-212165](https://reactome.org/content/detail/R-MMU-212165) | Epigenetic regulation of gene expression | 2 of 65 | 0.0328 |
| [MMU-111885](https://reactome.org/content/detail/R-MMU-111885) | Opioid Signalling | 2 of 64 | 0.0328 |
| [MMU-2029480](https://reactome.org/content/detail/R-MMU-2029480) | Fcgamma receptor (FCGR) dependent phagocytosis | 2 of 74 | 0.0404 |
| [MMU-76002](https://reactome.org/content/detail/R-MMU-76002) | Platelet activation, signaling and aggregation | 3 of 242 | 0.0451 |

**UniProt Keywords**

| keyword | description | count in gene set | false discovery rate |
| --- | --- | --- | --- |
| [KW-0375](https://www.uniprot.org/keywords/KW-0375) | Hydrogen ion transport | 8 of 48 | 6.16e-12 |
| [KW-0832](https://www.uniprot.org/keywords/KW-0832) | Ubl conjugation | 15 of 2091 | 4.72e-05 |
| [KW-0449](https://www.uniprot.org/keywords/KW-0449) | Lipoprotein | 10 of 790 | 4.72e-05 |
| [KW-0564](https://www.uniprot.org/keywords/KW-0564) | Palmitate | 6 of 326 | 0.00050 |
| [KW-1017](https://www.uniprot.org/keywords/KW-1017) | Isopeptide bond | 11 of 1442 | 0.00051 |
| [KW-0687](https://www.uniprot.org/keywords/KW-0687) | Ribonucleoprotein | 5 of 281 | 0.0020 |
| [KW-0597](https://www.uniprot.org/keywords/KW-0597) | Phosphoprotein | 25 of 7545 | 0.0020 |
| [KW-0399](https://www.uniprot.org/keywords/KW-0399) | Innate immunity | 5 of 287 | 0.0020 |
| [KW-0391](https://www.uniprot.org/keywords/KW-0391) | Immunity | 6 of 453 | 0.0020 |
| [KW-0164](https://www.uniprot.org/keywords/KW-0164) | Citrullination | 3 of 60 | 0.0020 |
| [KW-0689](https://www.uniprot.org/keywords/KW-0689) | Ribosomal protein | 4 of 182 | 0.0028 |
| [KW-0007](https://www.uniprot.org/keywords/KW-0007) | Acetylation | 14 of 3060 | 0.0039 |
| [KW-0547](https://www.uniprot.org/keywords/KW-0547) | Nucleotide-binding | 10 of 1738 | 0.0053 |
| [KW-0813](https://www.uniprot.org/keywords/KW-0813) | Transport | 10 of 1912 | 0.0103 |
| [KW-0336](https://www.uniprot.org/keywords/KW-0336) | GPI-anchor | 3 of 138 | 0.0129 |
| [KW-0342](https://www.uniprot.org/keywords/KW-0342) | GTP-binding | 4 of 324 | 0.0161 |
| [KW-0395](https://www.uniprot.org/keywords/KW-0395) | Inflammatory response | 3 of 158 | 0.0167 |
| [KW-0519](https://www.uniprot.org/keywords/KW-0519) | Myristate | 3 of 174 | 0.0207 |

**PFAM Protein Domains**

| *domain* | *description* | *count in gene set* | *false discovery rate* |
| --- | --- | --- | --- |
| [PF02874](https://pfam.xfam.org/family/PF02874) | ATP synthase alpha/beta family, beta-barrel domain | 2 of 5 | 0.0030 |
| [PF01480](https://pfam.xfam.org/family/PF01480) | PWI domain | 2 of 5 | 0.0030 |
| [PF00006](https://pfam.xfam.org/family/PF00006) | ATP synthase alpha/beta family, nucleotide-binding domain | 2 of 5 | 0.0030 |
| [PF00503](https://pfam.xfam.org/family/PF00503) | G-protein alpha subunit | 2 of 16 | 0.0055 |
| [PF00025](https://pfam.xfam.org/family/PF00025) | ADP-ribosylation factor family | 3 of 163 | 0.0300 |

**INTERPRO Protein Domains and Features**

| *domain* | *description* | *count in gene set* | *false discovery rate* |
| --- | --- | --- | --- |
| [IPR036483](https://www.ebi.ac.uk/interpro/entry/IPR036483) | PWI domain superfamily | 2 of 3 | 0.0032 |
| [IPR020003](https://www.ebi.ac.uk/interpro/entry/IPR020003) | ATPase, alpha/beta subunit, nucleotide-binding domain, active site | 2 of 5 | 0.0033 |
| [IPR004100](https://www.ebi.ac.uk/interpro/entry/IPR004100) | ATPase, F1/V1/A1 complex, alpha/beta subunit, N-terminal domain | 2 of 5 | 0.0033 |
| [IPR002483](https://www.ebi.ac.uk/interpro/entry/IPR002483) | PWI domain | 2 of 5 | 0.0033 |
| [IPR001408](https://www.ebi.ac.uk/interpro/entry/IPR001408) | G-protein alpha subunit, group I | 2 of 8 | 0.0033 |
| [IPR000194](https://www.ebi.ac.uk/interpro/entry/IPR000194) | ATPase, F1/V1/A1 complex, alpha/beta subunit, nucleotide-binding domain | 2 of 5 | 0.0033 |
| [IPR011025](https://www.ebi.ac.uk/interpro/entry/IPR011025) | G protein alpha subunit, helical insertion | 2 of 16 | 0.0068 |
| [IPR001019](https://www.ebi.ac.uk/interpro/entry/IPR001019) | Guanine nucleotide binding protein (G-protein), alpha subunit | 2 of 16 | 0.0068 |
| [IPR027417](https://www.ebi.ac.uk/interpro/entry/IPR027417) | P-loop containing nucleoside triphosphate hydrolase | 7 of 877 | 0.0094 |
| [IPR009000](https://www.ebi.ac.uk/interpro/entry/IPR009000) | Translation protein, beta-barrel domain superfamily | 2 of 30 | 0.0153 |
