# Supplementary material for: TBC1D10C is a cytoskeletal functional linker that modulates cell spreading and phagocytosis in macrophages
Source: Sci Rep. 2021 Oct 22;11:20946. doi: 10.1038/s41598-021-00450-z (PMC8536695; doi:10.1038/s41598-021-00450-z)
Supplement: Supplementary file 2 — Supplementary Information 2. [file 41598_2021_450_MOESM2_ESM.docx]

| number of nodes: 28  number of edges: 90  average node degree: 6.43  avg. local clustering coefficient: 0.729 |
| --- |

| expected number of edges: 32  PPI enrichment p-value: < 1.0e-16 |
| --- |

**Network Stats**

**Supplementary Table 1.** Functional enrichments in TBC1D10C-interacting protein network

**Biological Process (GO)**

| *GO-term* | *description* | *count in gene set* | *false discovery rate* |
| --- | --- | --- | --- |
| [GO:0022613](http://amigo.geneontology.org/amigo/term/GO:0022613) | ribonucleoprotein complex biogenesis | 11 of 380 | 9.07e-10 |
| [GO:0042254](http://amigo.geneontology.org/amigo/term/GO:0042254) | ribosome biogenesis | 9 of 252 | 1.32e-08 |
| [GO:0044085](http://amigo.geneontology.org/amigo/term/GO:0044085) | cellular component biogenesis | 17 of 2213 | 3.19e-08 |
| [GO:0071840](http://amigo.geneontology.org/amigo/term/GO:0071840) | cellular component organization or biogenesis | 21 of 4730 | 6.67e-07 |
| [GO:0006412](http://amigo.geneontology.org/amigo/term/GO:0006412) | translation | 8 of 313 | 1.05e-06 |
| [GO:0006518](http://amigo.geneontology.org/amigo/term/GO:0006518) | peptide metabolic process | 8 of 440 | 8.70e-06 |
| [GO:0022618](http://amigo.geneontology.org/amigo/term/GO:0022618) | ribonucleoprotein complex assembly | 6 of 192 | 2.03e-05 |
| [GO:0034641](http://amigo.geneontology.org/amigo/term/GO:0034641) | cellular nitrogen compound metabolic process | 18 of 4247 | 2.59e-05 |
| [GO:1901566](http://amigo.geneontology.org/amigo/term/GO:1901566) | organonitrogen compound biosynthetic process | 10 of 1122 | 6.70e-05 |
| [GO:0042255](http://amigo.geneontology.org/amigo/term/GO:0042255) | ribosome assembly | 4 of 59 | 9.90e-05 |
| [GO:0034622](http://amigo.geneontology.org/amigo/term/GO:0034622) | cellular protein-containing complex assembly | 8 of 687 | 0.00013 |
| [GO:0042273](http://amigo.geneontology.org/amigo/term/GO:0042273) | ribosomal large subunit biogenesis | 4 of 68 | 0.00015 |
| [GO:0006996](http://amigo.geneontology.org/amigo/term/GO:0006996) | organelle organization | 14 of 2805 | 0.00015 |
| [GO:0022607](http://amigo.geneontology.org/amigo/term/GO:0022607) | cellular component assembly | 12 of 2015 | 0.00017 |
| [GO:0009987](http://amigo.geneontology.org/amigo/term/GO:0009987) | cellular process | 27 of 12459 | 0.00017 |
| [GO:0006364](http://amigo.geneontology.org/amigo/term/GO:0006364) | rRNA processing | 5 of 176 | 0.00017 |
| [GO:0016043](http://amigo.geneontology.org/amigo/term/GO:0016043) | cellular component organization | 17 of 4560 | 0.00026 |
| [GO:0015991](http://amigo.geneontology.org/amigo/term/GO:0015991) | ATP hydrolysis coupled proton transport | 3 of 25 | 0.00030 |
| [GO:0000154](http://amigo.geneontology.org/amigo/term/GO:0000154) | rRNA modification | 3 of 29 | 0.00042 |
| [GO:0000027](http://amigo.geneontology.org/amigo/term/GO:0000027) | ribosomal large subunit assembly | 3 of 29 | 0.00042 |
| [GO:0070887](http://amigo.geneontology.org/amigo/term/GO:0070887) | cellular response to chemical stimulus | 12 of 2287 | 0.00043 |
| [GO:1901564](http://amigo.geneontology.org/amigo/term/GO:1901564) | organonitrogen compound metabolic process | 16 of 4480 | 0.00088 |
| [GO:0065003](http://amigo.geneontology.org/amigo/term/GO:0065003) | protein-containing complex assembly | 9 of 1292 | 0.00088 |
| [GO:0048254](http://amigo.geneontology.org/amigo/term/GO:0048254) | snoRNA localization | 2 of 4 | 0.00091 |
| [GO:0044267](http://amigo.geneontology.org/amigo/term/GO:0044267) | cellular protein metabolic process | 13 of 2995 | 0.0010 |
| [GO:0042221](http://amigo.geneontology.org/amigo/term/GO:0042221) | response to chemical | 14 of 3532 | 0.0011 |
| [GO:0010467](http://amigo.geneontology.org/amigo/term/GO:0010467) | gene expression | 13 of 3013 | 0.0011 |
| [GO:1990090](http://amigo.geneontology.org/amigo/term/GO:1990090) | cellular response to nerve growth factor stimulus | 3 of 47 | 0.0012 |
| [GO:0046034](http://amigo.geneontology.org/amigo/term/GO:0046034) | ATP metabolic process | 4 of 162 | 0.0017 |
| [GO:0070925](http://amigo.geneontology.org/amigo/term/GO:0070925) | organelle assembly | 6 of 576 | 0.0021 |
| [GO:0044271](http://amigo.geneontology.org/amigo/term/GO:0044271) | cellular nitrogen compound biosynthetic process | 12 of 2781 | 0.0021 |
| [GO:0006807](http://amigo.geneontology.org/amigo/term/GO:0006807) | nitrogen compound metabolic process | 19 of 6983 | 0.0025 |
| [GO:0009167](http://amigo.geneontology.org/amigo/term/GO:0009167) | purine ribonucleoside monophosphate metabolic process | 4 of 196 | 0.0028 |
| [GO:0036295](http://amigo.geneontology.org/amigo/term/GO:0036295) | cellular response to increased oxygen levels | 2 of 12 | 0.0032 |
| [GO:0044237](http://amigo.geneontology.org/amigo/term/GO:0044237) | cellular metabolic process | 19 of 7348 | 0.0042 |
| [GO:0044238](http://amigo.geneontology.org/amigo/term/GO:0044238) | primary metabolic process | 19 of 7426 | 0.0048 |
| [GO:0006396](http://amigo.geneontology.org/amigo/term/GO:0006396) | RNA processing | 6 of 715 | 0.0049 |
| [GO:0006139](http://amigo.geneontology.org/amigo/term/GO:0006139) | nucleobase-containing compound metabolic process | 13 of 3702 | 0.0051 |
| [GO:0071310](http://amigo.geneontology.org/amigo/term/GO:0071310) | cellular response to organic substance | 9 of 1858 | 0.0067 |
| [GO:0017144](http://amigo.geneontology.org/amigo/term/GO:0017144) | drug metabolic process | 5 of 494 | 0.0071 |
| [GO:0071704](http://amigo.geneontology.org/amigo/term/GO:0071704) | organic substance metabolic process | 19 of 7733 | 0.0078 |
| [GO:0060359](http://amigo.geneontology.org/amigo/term/GO:0060359) | response to ammonium ion | 3 of 122 | 0.0092 |
| [GO:0006417](http://amigo.geneontology.org/amigo/term/GO:0006417) | regulation of translation | 4 of 307 | 0.0105 |
| [GO:0071353](http://amigo.geneontology.org/amigo/term/GO:0071353) | cellular response to interleukin-4 | 2 of 27 | 0.0106 |
| [GO:0006414](http://amigo.geneontology.org/amigo/term/GO:0006414) | translational elongation | 2 of 27 | 0.0106 |
| [GO:0010033](http://amigo.geneontology.org/amigo/term/GO:0010033) | response to organic substance | 10 of 2553 | 0.0125 |
| [GO:1901576](http://amigo.geneontology.org/amigo/term/GO:1901576) | organic substance biosynthetic process | 12 of 3651 | 0.0145 |
| [GO:0006757](http://amigo.geneontology.org/amigo/term/GO:0006757) | ATP generation from ADP | 2 of 37 | 0.0163 |
| [GO:0006096](http://amigo.geneontology.org/amigo/term/GO:0006096) | glycolytic process | 2 of 37 | 0.0163 |
| [GO:0043170](http://amigo.geneontology.org/amigo/term/GO:0043170) | macromolecule metabolic process | 16 of 6225 | 0.0177 |
| [GO:0042866](http://amigo.geneontology.org/amigo/term/GO:0042866) | pyruvate biosynthetic process | 2 of 41 | 0.0184 |
| [GO:0071495](http://amigo.geneontology.org/amigo/term/GO:0071495) | cellular response to endogenous stimulus | 6 of 997 | 0.0186 |
| [GO:0034645](http://amigo.geneontology.org/amigo/term/GO:0034645) | cellular macromolecule biosynthetic process | 10 of 2778 | 0.0201 |
| [GO:2000377](http://amigo.geneontology.org/amigo/term/GO:2000377) | regulation of reactive oxygen species metabolic process | 3 of 184 | 0.0215 |
| [GO:0051402](http://amigo.geneontology.org/amigo/term/GO:0051402) | neuron apoptotic process | 2 of 48 | 0.0228 |
| [GO:0009628](http://amigo.geneontology.org/amigo/term/GO:0009628) | response to abiotic stimulus | 6 of 1063 | 0.0236 |
| [GO:0071103](http://amigo.geneontology.org/amigo/term/GO:0071103) | DNA conformation change | 3 of 198 | 0.0251 |
| [GO:0071363](http://amigo.geneontology.org/amigo/term/GO:0071363) | cellular response to growth factor stimulus | 4 of 437 | 0.0266 |
| [GO:0044260](http://amigo.geneontology.org/amigo/term/GO:0044260) | cellular macromolecule metabolic process | 14 of 5233 | 0.0268 |
| [GO:0019359](http://amigo.geneontology.org/amigo/term/GO:0019359) | nicotinamide nucleotide biosynthetic process | 2 of 54 | 0.0268 |
| [GO:0032268](http://amigo.geneontology.org/amigo/term/GO:0032268) | regulation of cellular protein metabolic process | 9 of 2443 | 0.0278 |
| [GO:0006879](http://amigo.geneontology.org/amigo/term/GO:0006879) | cellular iron ion homeostasis | 2 of 56 | 0.0278 |
| [GO:0006090](http://amigo.geneontology.org/amigo/term/GO:0006090) | pyruvate metabolic process | 2 of 62 | 0.0315 |
| [GO:0042274](http://amigo.geneontology.org/amigo/term/GO:0042274) | ribosomal small subunit biogenesis | 2 of 70 | 0.0384 |
| [GO:0010941](http://amigo.geneontology.org/amigo/term/GO:0010941) | regulation of cell death | 7 of 1640 | 0.0384 |
| [GO:0006754](http://amigo.geneontology.org/amigo/term/GO:0006754) | ATP biosynthetic process | 2 of 70 | 0.0384 |
| [GO:0033365](http://amigo.geneontology.org/amigo/term/GO:0033365) | protein localization to organelle | 4 of 515 | 0.0393 |
| [GO:0051276](http://amigo.geneontology.org/amigo/term/GO:0051276) | chromosome organization | 5 of 883 | 0.0463 |
| [GO:0043066](http://amigo.geneontology.org/amigo/term/GO:0043066) | negative regulation of apoptotic process | 5 of 884 | 0.0463 |
| [GO:0071214](http://amigo.geneontology.org/amigo/term/GO:0071214) | cellular response to abiotic stimulus | 3 of 272 | 0.0465 |
| [GO:0006334](http://amigo.geneontology.org/amigo/term/GO:0006334) | nucleosome assembly | 2 of 80 | 0.0465 |
| [GO:0007010](http://amigo.geneontology.org/amigo/term/GO:0007010) | cytoskeleton organization | 5 of 916 | 0.0490 |
| [GO:0006986](http://amigo.geneontology.org/amigo/term/GO:0006986) | response to unfolded protein | 2 of 86 | 0.0490 |
| [GO:1903426](http://amigo.geneontology.org/amigo/term/GO:1903426) | regulation of reactive oxygen species biosynthetic process | 2 of 87 | 0.0494 |

**Molecular Function (GO)**

| *GO-term* | *description* | *count in gene set* | *false discovery rate* |
| --- | --- | --- | --- |
| [GO:1901363](http://amigo.geneontology.org/amigo/term/GO:1901363) | heterocyclic compound binding | 25 of 4748 | 9.26e-12 |
| [GO:0097159](http://amigo.geneontology.org/amigo/term/GO:0097159) | organic cyclic compound binding | 25 of 4818 | 9.26e-12 |
| [GO:0003723](http://amigo.geneontology.org/amigo/term/GO:0003723) | RNA binding | 13 of 986 | 5.18e-09 |
| [GO:0017111](http://amigo.geneontology.org/amigo/term/GO:0017111) | nucleoside-triphosphatase activity | 11 of 714 | 3.63e-08 |
| [GO:0005488](http://amigo.geneontology.org/amigo/term/GO:0005488) | binding | 28 of 10884 | 8.39e-08 |
| [GO:0003924](http://amigo.geneontology.org/amigo/term/GO:0003924) | GTPase activity | 7 of 255 | 8.23e-07 |
| [GO:0000166](http://amigo.geneontology.org/amigo/term/GO:0000166) | nucleotide binding | 14 of 2006 | 8.23e-07 |
| [GO:0035639](http://amigo.geneontology.org/amigo/term/GO:0035639) | purine ribonucleoside triphosphate binding | 13 of 1697 | 9.03e-07 |
| [GO:0032555](http://amigo.geneontology.org/amigo/term/GO:0032555) | purine ribonucleotide binding | 13 of 1766 | 1.33e-06 |
| [GO:0005525](http://amigo.geneontology.org/amigo/term/GO:0005525) | GTP binding | 7 of 338 | 3.02e-06 |
| [GO:0005198](http://amigo.geneontology.org/amigo/term/GO:0005198) | structural molecule activity | 8 of 546 | 3.31e-06 |
| [GO:0003676](http://amigo.geneontology.org/amigo/term/GO:0003676) | nucleic acid binding | 15 of 2868 | 3.74e-06 |
| [GO:0016787](http://amigo.geneontology.org/amigo/term/GO:0016787) | hydrolase activity | 13 of 2259 | 1.11e-05 |
| [GO:0005200](http://amigo.geneontology.org/amigo/term/GO:0005200) | structural constituent of cytoskeleton | 4 of 67 | 1.71e-05 |
| [GO:0019899](http://amigo.geneontology.org/amigo/term/GO:0019899) | enzyme binding | 12 of 2175 | 4.80e-05 |
| [GO:0003729](http://amigo.geneontology.org/amigo/term/GO:0003729) | mRNA binding | 5 of 202 | 4.80e-05 |
| [GO:0003824](http://amigo.geneontology.org/amigo/term/GO:0003824) | catalytic activity | 17 of 5239 | 0.00026 |
| [GO:0003735](http://amigo.geneontology.org/amigo/term/GO:0003735) | structural constituent of ribosome | 4 of 153 | 0.00034 |
| [GO:0044877](http://amigo.geneontology.org/amigo/term/GO:0044877) | protein-containing complex binding | 8 of 1094 | 0.00036 |
| [GO:0001094](http://amigo.geneontology.org/amigo/term/GO:0001094) | TFIID-class transcription factor complex binding | 2 of 9 | 0.00061 |
| [GO:0019843](http://amigo.geneontology.org/amigo/term/GO:0019843) | rRNA binding | 3 of 67 | 0.00066 |
| [GO:0016887](http://amigo.geneontology.org/amigo/term/GO:0016887) | ATPase activity | 5 of 372 | 0.00069 |
| [GO:0005515](http://amigo.geneontology.org/amigo/term/GO:0005515) | protein binding | 18 of 6454 | 0.00087 |
| [GO:0008097](http://amigo.geneontology.org/amigo/term/GO:0008097) | 5S rRNA binding | 2 of 13 | 0.0010 |
| [GO:0003746](http://amigo.geneontology.org/amigo/term/GO:0003746) | translation elongation factor activity | 2 of 15 | 0.0013 |
| [GO:0046961](http://amigo.geneontology.org/amigo/term/GO:0046961) | proton-transporting ATPase activity, rotational mechanism | 2 of 18 | 0.0018 |
| [GO:0045182](http://amigo.geneontology.org/amigo/term/GO:0045182) | translation regulator activity | 3 of 117 | 0.0027 |
| [GO:0043021](http://amigo.geneontology.org/amigo/term/GO:0043021) | ribonucleoprotein complex binding | 3 of 136 | 0.0039 |
| [GO:0030515](http://amigo.geneontology.org/amigo/term/GO:0030515) | snoRNA binding | 2 of 29 | 0.0039 |
| [GO:0008144](http://amigo.geneontology.org/amigo/term/GO:0008144) | drug binding | 8 of 1630 | 0.0039 |
| [GO:0097718](http://amigo.geneontology.org/amigo/term/GO:0097718) | disordered domain specific binding | 2 of 34 | 0.0049 |
| [GO:0019901](http://amigo.geneontology.org/amigo/term/GO:0019901) | protein kinase binding | 5 of 659 | 0.0068 |
| [GO:0005524](http://amigo.geneontology.org/amigo/term/GO:0005524) | ATP binding | 7 of 1389 | 0.0071 |
| [GO:0000049](http://amigo.geneontology.org/amigo/term/GO:0000049) | tRNA binding | 2 of 54 | 0.0100 |
| [GO:0043167](http://amigo.geneontology.org/amigo/term/GO:0043167) | ion binding | 14 of 5302 | 0.0109 |
| [GO:0043022](http://amigo.geneontology.org/amigo/term/GO:0043022) | ribosome binding | 2 of 57 | 0.0109 |
| [GO:0019904](http://amigo.geneontology.org/amigo/term/GO:0019904) | protein domain specific binding | 5 of 775 | 0.0112 |
| [GO:0051082](http://amigo.geneontology.org/amigo/term/GO:0051082) | unfolded protein binding | 2 of 77 | 0.0175 |
| [GO:0008092](http://amigo.geneontology.org/amigo/term/GO:0008092) | cytoskeletal protein binding | 5 of 877 | 0.0175 |
| [GO:0051117](http://amigo.geneontology.org/amigo/term/GO:0051117) | ATPase binding | 2 of 91 | 0.0230 |
| [GO:0042623](http://amigo.geneontology.org/amigo/term/GO:0042623) | ATPase activity, coupled | 3 of 301 | 0.0237 |
| [GO:0140098](http://amigo.geneontology.org/amigo/term/GO:0140098) | catalytic activity, acting on RNA | 3 of 317 | 0.0263 |
| [GO:0031625](http://amigo.geneontology.org/amigo/term/GO:0031625) | ubiquitin protein ligase binding | 3 of 316 | 0.0263 |
| [GO:0008134](http://amigo.geneontology.org/amigo/term/GO:0008134) | transcription factor binding | 4 of 644 | 0.0286 |
| [GO:0001085](http://amigo.geneontology.org/amigo/term/GO:0001085) | RNA polymerase II transcription factor binding | 2 of 130 | 0.0383 |

**Cellular Component (GO)**

| *GO-term* | *description* | *count in gene set* | *false discovery rate* |
| --- | --- | --- | --- |
| [GO:1990904](http://amigo.geneontology.org/amigo/term/GO:1990904) | ribonucleoprotein complex | 14 of 765 | 3.04e-11 |
| [GO:0032991](http://amigo.geneontology.org/amigo/term/GO:0032991) | protein-containing complex | 23 of 4701 | 1.73e-09 |
| [GO:0005829](http://amigo.geneontology.org/amigo/term/GO:0005829) | cytosol | 20 of 3326 | 3.40e-09 |
| [GO:0043209](http://amigo.geneontology.org/amigo/term/GO:0043209) | myelin sheath | 8 of 212 | 1.63e-08 |
| [GO:0043232](http://amigo.geneontology.org/amigo/term/GO:0043232) | intracellular non-membrane-bounded organelle | 19 of 3809 | 2.69e-07 |
| [GO:0044444](http://amigo.geneontology.org/amigo/term/GO:0044444) | cytoplasmic part | 24 of 7673 | 1.70e-06 |
| [GO:0044424](http://amigo.geneontology.org/amigo/term/GO:0044424) | intracellular part | 28 of 12219 | 2.45e-06 |
| [GO:0044428](http://amigo.geneontology.org/amigo/term/GO:0044428) | nuclear part | 16 of 3798 | 6.55e-05 |
| [GO:0098805](http://amigo.geneontology.org/amigo/term/GO:0098805) | whole membrane | 10 of 1300 | 6.76e-05 |
| [GO:0044422](http://amigo.geneontology.org/amigo/term/GO:0044422) | organelle part | 22 of 7665 | 6.76e-05 |
| [GO:0036464](http://amigo.geneontology.org/amigo/term/GO:0036464) | cytoplasmic ribonucleoprotein granule | 5 of 182 | 7.00e-05 |
| [GO:0033176](http://amigo.geneontology.org/amigo/term/GO:0033176) | proton-transporting V-type ATPase complex | 3 of 22 | 7.48e-05 |
| [GO:0005732](http://amigo.geneontology.org/amigo/term/GO:0005732) | small nucleolar ribonucleoprotein complex | 3 of 22 | 7.48e-05 |
| [GO:0043229](http://amigo.geneontology.org/amigo/term/GO:0043229) | intracellular organelle | 25 of 10645 | 9.71e-05 |
| [GO:0045121](http://amigo.geneontology.org/amigo/term/GO:0045121) | membrane raft | 6 of 374 | 9.91e-05 |
| [GO:0005840](http://amigo.geneontology.org/amigo/term/GO:0005840) | ribosome | 5 of 215 | 0.00010 |
| [GO:0044446](http://amigo.geneontology.org/amigo/term/GO:0044446) | intracellular organelle part | 21 of 7416 | 0.00011 |
| [GO:0044445](http://amigo.geneontology.org/amigo/term/GO:0044445) | cytosolic part | 5 of 228 | 0.00011 |
| [GO:0022626](http://amigo.geneontology.org/amigo/term/GO:0022626) | cytosolic ribosome | 4 of 107 | 0.00011 |
| [GO:0005730](http://amigo.geneontology.org/amigo/term/GO:0005730) | nucleolus | 8 of 878 | 0.00011 |
| [GO:0005634](http://amigo.geneontology.org/amigo/term/GO:0005634) | nucleus | 19 of 6086 | 0.00011 |
| [GO:0070013](http://amigo.geneontology.org/amigo/term/GO:0070013) | intracellular organelle lumen | 15 of 3882 | 0.00017 |
| [GO:0120025](http://amigo.geneontology.org/amigo/term/GO:0120025) | plasma membrane bounded cell projection | 11 of 2172 | 0.00031 |
| [GO:0015030](http://amigo.geneontology.org/amigo/term/GO:0015030) | Cajal body | 3 of 51 | 0.00035 |
| [GO:0043227](http://amigo.geneontology.org/amigo/term/GO:0043227) | membrane-bounded organelle | 23 of 9775 | 0.00037 |
| [GO:0098590](http://amigo.geneontology.org/amigo/term/GO:0098590) | plasma membrane region | 8 of 1115 | 0.00041 |
| [GO:0033180](http://amigo.geneontology.org/amigo/term/GO:0033180) | proton-transporting V-type ATPase, V1 domain | 2 of 7 | 0.00041 |
| [GO:0043231](http://amigo.geneontology.org/amigo/term/GO:0043231) | intracellular membrane-bounded organelle | 22 of 9088 | 0.00046 |
| [GO:0022625](http://amigo.geneontology.org/amigo/term/GO:0022625) | cytosolic large ribosomal subunit | 3 of 59 | 0.00046 |
| [GO:0031428](http://amigo.geneontology.org/amigo/term/GO:0031428) | box C/D snoRNP complex | 2 of 8 | 0.00047 |
| [GO:0044391](http://amigo.geneontology.org/amigo/term/GO:0044391) | ribosomal subunit | 4 of 181 | 0.00055 |
| [GO:0031981](http://amigo.geneontology.org/amigo/term/GO:0031981) | nuclear lumen | 13 of 3386 | 0.00065 |
| [GO:0005844](http://amigo.geneontology.org/amigo/term/GO:0005844) | polysome | 3 of 71 | 0.00070 |
| [GO:0030684](http://amigo.geneontology.org/amigo/term/GO:0030684) | preribosome | 3 of 72 | 0.00072 |
| [GO:0005886](http://amigo.geneontology.org/amigo/term/GO:0005886) | plasma membrane | 14 of 4328 | 0.0018 |
| [GO:0042470](http://amigo.geneontology.org/amigo/term/GO:0042470) | melanosome | 3 of 103 | 0.0019 |
| [GO:0120038](http://amigo.geneontology.org/amigo/term/GO:0120038) | plasma membrane bounded cell projection part | 8 of 1532 | 0.0026 |
| [GO:0031253](http://amigo.geneontology.org/amigo/term/GO:0031253) | cell projection membrane | 4 of 316 | 0.0035 |
| [GO:0044448](http://amigo.geneontology.org/amigo/term/GO:0044448) | cell cortex part | 3 of 136 | 0.0036 |
| [GO:0042788](http://amigo.geneontology.org/amigo/term/GO:0042788) | polysomal ribosome | 2 of 30 | 0.0038 |
| [GO:0032040](http://amigo.geneontology.org/amigo/term/GO:0032040) | small-subunit processome | 2 of 33 | 0.0043 |
| [GO:0016324](http://amigo.geneontology.org/amigo/term/GO:0016324) | apical plasma membrane | 4 of 339 | 0.0043 |
| [GO:0001726](http://amigo.geneontology.org/amigo/term/GO:0001726) | ruffle | 3 of 168 | 0.0062 |
| [GO:0044452](http://amigo.geneontology.org/amigo/term/GO:0044452) | nucleolar part | 3 of 175 | 0.0068 |
| [GO:0044451](http://amigo.geneontology.org/amigo/term/GO:0044451) | nucleoplasm part | 6 of 1023 | 0.0072 |
| [GO:0016604](http://amigo.geneontology.org/amigo/term/GO:0016604) | nuclear body | 5 of 709 | 0.0084 |
| [GO:0044459](http://amigo.geneontology.org/amigo/term/GO:0044459) | plasma membrane part | 9 of 2362 | 0.0086 |
| [GO:0044425](http://amigo.geneontology.org/amigo/term/GO:0044425) | membrane part | 15 of 5857 | 0.0095 |
| [GO:0005856](http://amigo.geneontology.org/amigo/term/GO:0005856) | cytoskeleton | 8 of 1933 | 0.0095 |
| [GO:0098858](http://amigo.geneontology.org/amigo/term/GO:0098858) | actin-based cell projection | 3 of 216 | 0.0110 |
| [GO:0016020](http://amigo.geneontology.org/amigo/term/GO:0016020) | membrane | 17 of 7460 | 0.0135 |
| [GO:0000786](http://amigo.geneontology.org/amigo/term/GO:0000786) | nucleosome | 2 of 67 | 0.0141 |
| [GO:0005654](http://amigo.geneontology.org/amigo/term/GO:0005654) | nucleoplasm | 9 of 2648 | 0.0168 |
| [GO:1902494](http://amigo.geneontology.org/amigo/term/GO:1902494) | catalytic complex | 6 of 1266 | 0.0176 |
| [GO:0097458](http://amigo.geneontology.org/amigo/term/GO:0097458) | neuron part | 7 of 1732 | 0.0192 |
| [GO:0032587](http://amigo.geneontology.org/amigo/term/GO:0032587) | ruffle membrane | 2 of 85 | 0.0204 |
| [GO:0030863](http://amigo.geneontology.org/amigo/term/GO:0030863) | cortical cytoskeleton | 2 of 85 | 0.0204 |
| [GO:0005902](http://amigo.geneontology.org/amigo/term/GO:0005902) | microvillus | 2 of 95 | 0.0246 |
| [GO:0043005](http://amigo.geneontology.org/amigo/term/GO:0043005) | neuron projection | 6 of 1429 | 0.0296 |
| [GO:0044430](http://amigo.geneontology.org/amigo/term/GO:0044430) | cytoskeletal part | 6 of 1460 | 0.0323 |
| [GO:0150034](http://amigo.geneontology.org/amigo/term/GO:0150034) | distal axon | 3 of 378 | 0.0416 |
| [GO:0030424](http://amigo.geneontology.org/amigo/term/GO:0030424) | axon | 4 of 712 | 0.0416 |
| [GO:0015630](http://amigo.geneontology.org/amigo/term/GO:0015630) | microtubule cytoskeleton | 5 of 1106 | 0.0416 |
| [GO:0012505](http://amigo.geneontology.org/amigo/term/GO:0012505) | endomembrane system | 10 of 3670 | 0.0416 |
| [GO:0001650](http://amigo.geneontology.org/amigo/term/GO:0001650) | fibrillar center | 2 of 128 | 0.0416 |
| [GO:0005903](http://amigo.geneontology.org/amigo/term/GO:0005903) | brush border | 2 of 133 | 0.0422 |

**Reference publications**

| *publication* | *(year) title* | *count in gene set* | *false discovery rate* |
| --- | --- | --- | --- |
| [PMID:28736500](https://www.ncbi.nlm.nih.gov/pubmed/28736500) | (2017) Comparative Proteomic Analysis of Three Chinese Hamster Ovary (CHO) Host Cells. | 7 of 50 | 1.19e-07 |
| [PMID:23696868](https://www.ncbi.nlm.nih.gov/pubmed/23696868) | (2013) Enhanced translation of mRNAs encoding proteins involved in mRNA translation during recovery from heat shock. | 6 of 28 | 2.96e-07 |
| [PMID:16822315](https://www.ncbi.nlm.nih.gov/pubmed/16822315) | (2006) Gene identification and analysis of transcripts differentially regulated in fracture healing by EST sequencing in the domestic sheep. | 7 of 64 | 2.96e-07 |
| [PMID:29986939](https://www.ncbi.nlm.nih.gov/pubmed/29986939) | (2018) MSC exosome works through a protein-based mechanism of action. | 6 of 33 | 3.74e-07 |
| [PMID:26399832](https://www.ncbi.nlm.nih.gov/pubmed/26399832) | (2015) Cell-fate determination by ubiquitin-dependent regulation of translation. | 6 of 44 | 1.44e-06 |
| [PMID:25993305](https://www.ncbi.nlm.nih.gov/pubmed/25993305) | (2015) MALDI-Mass Spectrometric Imaging Revealing Hypoxia-Driven Lipids and Proteins in a Breast Tumor Model. | 6 of 44 | 1.44e-06 |
| [PMID:31151297](https://www.ncbi.nlm.nih.gov/pubmed/31151297) | (2019) Identification of Proteins Differentially Expressed by Adipose-derived Mesenchymal Stem Cells Isolated from Immunodeficient Mice. | 8 of 180 | 1.84e-06 |
| [PMID:31033440](https://www.ncbi.nlm.nih.gov/pubmed/31033440) | (2019) HRI coordinates translation necessary for protein homeostasis and mitochondrial function in erythropoiesis. | 6 of 53 | 2.36e-06 |
| [PMID:30260431](https://www.ncbi.nlm.nih.gov/pubmed/30260431) | (2018) Protein Syndesmos is a novel RNA-binding protein that regulates primary cilia formation. | 6 of 54 | 2.36e-06 |
| [PMID:20701774](https://www.ncbi.nlm.nih.gov/pubmed/20701774) | (2010) A novel function for vimentin: the potential biomarker for predicting melanoma hematogenous metastasis. | 5 of 19 | 2.36e-06 |
| [PMID:24809507](https://www.ncbi.nlm.nih.gov/pubmed/24809507) | (2014) Multi-faceted proteomic characterization of host protein complement of Rift Valley fever virus virions and identification of specific heat shock proteins, including HSP90, as important viral host factors. | 6 of 59 | 3.36e-06 |
| [PMID:31698554](https://www.ncbi.nlm.nih.gov/pubmed/31698554) | (2019) LGR5 and Downstream Intracellular Signaling Proteins Play Critical Roles in the Cell Proliferation of Neuroblastoma, Meningioma and Pituitary Adenoma. | 5 of 24 | 4.38e-06 |
| [PMID:31460127](https://www.ncbi.nlm.nih.gov/pubmed/31460127) | (2019) Targeting Translation Activity at the Ribosome Interface with UV-Active Small Molecules. | 5 of 24 | 4.38e-06 |
| [PMID:26544960](https://www.ncbi.nlm.nih.gov/pubmed/26544960) | (2015) Lysine Methylation of the Valosin-Containing Protein (VCP) Is Dispensable for Development and Survival of Mice. | 5 of 24 | 4.38e-06 |
| [PMID:24113185](https://www.ncbi.nlm.nih.gov/pubmed/24113185) | (2013) Translational control in the stress adaptive response of cancer cells: a novel role for the heat shock protein TRAP1. | 5 of 24 | 4.38e-06 |
| [PMID:28611246](https://www.ncbi.nlm.nih.gov/pubmed/28611246) | (2017) Inhibition of Avian Influenza A Virus Replication in Human Cells by Host Restriction Factor TUFM Is Correlated with Autophagy. | 5 of 27 | 5.56e-06 |
| [PMID:23690912](https://www.ncbi.nlm.nih.gov/pubmed/23690912) | (2013) New model of action for mood stabilizers: phosphoproteome from rat pre-frontal cortex synaptoneurosomal preparations. | 5 of 27 | 5.56e-06 |
| [PMID:30891164](https://www.ncbi.nlm.nih.gov/pubmed/30891164) | (2019) Extracellular vesicles derived from natural killer cells use multiple cytotoxic proteins and killing mechanisms to target cancer cells. | 5 of 28 | 5.82e-06 |
| [PMID:30295850](https://www.ncbi.nlm.nih.gov/pubmed/30295850) | (2018) RNA-binding protein DDX1 is responsible for fatty acid-mediated repression of insulin translation. | 5 of 28 | 5.82e-06 |
| [PMID:23555724](https://www.ncbi.nlm.nih.gov/pubmed/23555724) | (2013) Oxidative stress induces monocyte necrosis with enrichment of cell-bound albumin and overexpression of endoplasmic reticulum and mitochondrial chaperones. | 5 of 29 | 5.82e-06 |
| [PMID:22809209](https://www.ncbi.nlm.nih.gov/pubmed/22809209) | (2012) Insights into the virulence of oral biofilms: discoveries from proteomics. | 5 of 28 | 5.82e-06 |
| [PMID:20529248](https://www.ncbi.nlm.nih.gov/pubmed/20529248) | (2010) Proteomic analysis of primary duck hepatocytes infected with duck hepatitis B virus. | 5 of 28 | 5.82e-06 |
| [PMID:28747643](https://www.ncbi.nlm.nih.gov/pubmed/28747643) | (2017) Generation of ribosome imprinted polymers for sensitive detection of translational responses. | 4 of 7 | 5.93e-06 |
| [PMID:22483619](https://www.ncbi.nlm.nih.gov/pubmed/22483619) | (2012) mRNA decapping factors and the exonuclease Xrn2 function in widespread premature termination of RNA polymerase II transcription. | 5 of 30 | 5.96e-06 |
| [PMID:26340096](https://www.ncbi.nlm.nih.gov/pubmed/26340096) | (2015) Ribosomal Biogenesis and Translational Flux Inhibition by the Selective Inhibitor of Nuclear Export (SINE) XPO1 Antagonist KPT-185. | 5 of 34 | 1.01e-05 |
| [PMID:26630129](https://www.ncbi.nlm.nih.gov/pubmed/26630129) | (2015) Transcriptional Profiling Identifies Location-Specific and Breed-Specific Differentially Expressed Genes in Embryonic Myogenesis in Anas Platyrhynchos. | 5 of 35 | 1.11e-05 |
| [PMID:27512079](https://www.ncbi.nlm.nih.gov/pubmed/27512079) | (2016) Quantitative Non-canonical Amino Acid Tagging (QuaNCAT) Proteomics Identifies Distinct Patterns of Protein Synthesis Rapidly Induced by Hypertrophic Agents in Cardiomyocytes, Revealing New Aspects of Metabolic Remodeling. | 5 of 37 | 1.38e-05 |
| [PMID:30862090](https://www.ncbi.nlm.nih.gov/pubmed/30862090) | (2019) Emerging Role of Eukaryote Ribosomes in Translational Control. | 5 of 38 | 1.47e-05 |
| [PMID:22537006](https://www.ncbi.nlm.nih.gov/pubmed/22537006) | (2012) PIntron: a fast method for detecting the gene structure due to alternative splicing via maximal pairings of a pattern and a text. | 4 of 10 | 1.47e-05 |
| [PMID:30302013](https://www.ncbi.nlm.nih.gov/pubmed/30302013) | (2018) Porcine Reproductive and Respiratory Syndrome Virus strains with Higher Virulence Cause Marked Protein Profile Changes in MARC-145 Cells. | 7 of 179 | 1.73e-05 |
| [PMID:24278208](https://www.ncbi.nlm.nih.gov/pubmed/24278208) | (2013) Differential phosphoproteome regulation of nucleus accumbens in environmentally enriched and isolated rats in response to acute stress. | 5 of 40 | 1.73e-05 |
| [PMID:20140087](https://www.ncbi.nlm.nih.gov/pubmed/20140087) | (2010) Comprehensive identification and modified-site mapping of S-nitrosylated targets in prostate epithelial cells. | 6 of 94 | 1.73e-05 |
| [PMID:28884116](https://www.ncbi.nlm.nih.gov/pubmed/28884116) | (2017) The Role of Pontin and Reptin in Cellular Physiology and Cancer Etiology. | 6 of 99 | 2.03e-05 |
| [PMID:23861804](https://www.ncbi.nlm.nih.gov/pubmed/23861804) | (2013) Proteomic profiling of rabbit embryonic stem cells derived from parthenotes and fertilized embryos. | 5 of 42 | 2.03e-05 |
| [PMID:19811410](https://www.ncbi.nlm.nih.gov/pubmed/19811410) | (2009) Proteomic analyses of the effects of drugs of abuse on monocyte-derived mature dendritic cells. | 5 of 42 | 2.03e-05 |
| [PMID:26939752](https://www.ncbi.nlm.nih.gov/pubmed/26939752) | (2016) Long noncoding RNAs (lncRNAs) dynamics evidence immunomodulation during ISAV-Infected Atlantic salmon (Salmo salar). | 4 of 12 | 2.07e-05 |
| [PMID:25307283](https://www.ncbi.nlm.nih.gov/pubmed/25307283) | (2015) The effect of sulforaphane on histone deacetylase activity in keratinocytes: Differences between in vitro and in vivo analyses. | 4 of 12 | 2.07e-05 |
| [PMID:20000738](https://www.ncbi.nlm.nih.gov/pubmed/20000738) | (2010) Proteomic dissection of cell type-specific H2AX-interacting protein complex associated with hepatocellular carcinoma. | 5 of 44 | 2.07e-05 |
| [PMID:19427311](https://www.ncbi.nlm.nih.gov/pubmed/19427311) | (2009) Molecular mechanisms of pancreatic dysfunction induced by protein malnutrition. | 4 of 12 | 2.07e-05 |
| [PMID:18302751](https://www.ncbi.nlm.nih.gov/pubmed/18302751) | (2008) Characterization of human mesenchymal stem cell secretome at early steps of adipocyte and osteoblast differentiation. | 5 of 43 | 2.07e-05 |
| [PMID:27152104](https://www.ncbi.nlm.nih.gov/pubmed/27152104) | (2016) Clinical proteomics of enervated neurons. | 5 of 45 | 2.25e-05 |
| [PMID:30852271](https://www.ncbi.nlm.nih.gov/pubmed/30852271) | (2019) N-Linked glycosylation of the membrane protein ectodomain regulates infectious bronchitis virus-induced ER stress response, apoptosis and pathogenesis. | 4 of 13 | 2.33e-05 |
| [PMID:29850612](https://www.ncbi.nlm.nih.gov/pubmed/29850612) | (2018) Proteomic Analysis of Hippocampus and Cortex in Streptozotocin-Induced Diabetic Model Mice Showing Dementia. | 5 of 46 | 2.33e-05 |
| [PMID:29337352](https://www.ncbi.nlm.nih.gov/pubmed/29337352) | (2018) Genetic removal of eIF2Alfa kinase PERK in mice enables hippocampal L-LTP independent of mTORC1 activity. | 4 of 13 | 2.33e-05 |
| [PMID:18596936](https://www.ncbi.nlm.nih.gov/pubmed/18596936) | (2008) Sex-specific expression of the X-linked histone demethylase gene Jarid1c in brain. | 4 of 13 | 2.33e-05 |
| [PMID:25923296](https://www.ncbi.nlm.nih.gov/pubmed/25923296) | (2015) Use of both cumulus cells' transcriptomic markers and zona pellucida birefringence to select developmentally competent oocytes in human assisted reproductive technologies. | 5 of 47 | 2.45e-05 |
| [PMID:25407680](https://www.ncbi.nlm.nih.gov/pubmed/25407680) | (2014) The dyskerin ribonucleoprotein complex as an OCT4SOX2 coactivator in embryonic stem cells. | 5 of 47 | 2.45e-05 |
| [PMID:29499948](https://www.ncbi.nlm.nih.gov/pubmed/29499948) | (2018) Endogenous Cellular MicroRNAs Mediate Antiviral Defense against Influenza A Virus. | 4 of 14 | 2.62e-05 |
| [PMID:26304123](https://www.ncbi.nlm.nih.gov/pubmed/26304123) | (2015) mRNA Targeting to Endoplasmic Reticulum Precedes Ago Protein Interaction and MicroRNA (miRNA)-mediated Translation Repression in Mammalian Cells. | 4 of 14 | 2.62e-05 |
| [PMID:24213167](https://www.ncbi.nlm.nih.gov/pubmed/24213167) | (2014) Parallel measurement of dynamic changes in translation rates in single cells. | 4 of 14 | 2.62e-05 |
| [PMID:21986946](https://www.ncbi.nlm.nih.gov/pubmed/21986946) | (2012) Small nucleolar RNA 42 acts as an oncogene in lung tumorigenesis. | 4 of 14 | 2.62e-05 |
| [PMID:28536652](https://www.ncbi.nlm.nih.gov/pubmed/28536652) | (2017) Mesencephalic astrocyte-derived neurotrophic factor reduces cell apoptosis via upregulating HSP70 in SHSY-5Y cells. | 4 of 15 | 3.06e-05 |
| [PMID:25579851](https://www.ncbi.nlm.nih.gov/pubmed/25579851) | (2016) Moderate Alcohol Drinking and the Amygdala Proteome: Identification and Validation of CalciumCalmodulin Dependent Kinase II and AMPA Receptor Activity as Novel Molecular Mechanisms of the Positive Reinforcing Effects of Alcohol. | 4 of 15 | 3.06e-05 |
| [PMID:25664316](https://www.ncbi.nlm.nih.gov/pubmed/25664316) | (2015) Isoform composition and gene expression of thick and thin filament proteins in striated muscles of mice after 30-day space flight. | 4 of 15 | 3.06e-05 |
| [PMID:23407963](https://www.ncbi.nlm.nih.gov/pubmed/23407963) | (2013) A novel role of cytosolic protein synthesis inhibition in aminoglycoside ototoxicity. | 4 of 15 | 3.06e-05 |
| [PMID:31987042](https://www.ncbi.nlm.nih.gov/pubmed/31987042) | (2020) The role of the redoxmiR-6855-3pPRDX5A axis in reversing SLUG-mediated BRCA2 silencing in breast cancer cells. | 4 of 16 | 3.53e-05 |
| [PMID:27694897](https://www.ncbi.nlm.nih.gov/pubmed/27694897) | (2017) Systematic and functional characterization of novel androgen receptor variants arising from alternative splicing in the ligand-binding domain. | 4 of 16 | 3.53e-05 |
| [PMID:27110132](https://www.ncbi.nlm.nih.gov/pubmed/27110132) | (2016) Identification of HSPA8 as a candidate biomarker for endometrial carcinoma by using iTRAQ-based proteomic analysis. | 4 of 16 | 3.53e-05 |
| [PMID:26102067](https://www.ncbi.nlm.nih.gov/pubmed/26102067) | (2015) Comparative Label-Free Mass Spectrometric Analysis of Mildly versus Severely Affected mdx Mouse Skeletal Muscles Identifies Annexin, Lamin, and Vimentin as Universal Dystrophic Markers. | 5 of 53 | 3.53e-05 |
| [PMID:23874968](https://www.ncbi.nlm.nih.gov/pubmed/23874968) | (2013) Inhibition of inducible heat shock protein-70 (hsp72) enhances bortezomib-induced cell death in human bladder cancer cells. | 4 of 16 | 3.53e-05 |
| [PMID:21480387](https://www.ncbi.nlm.nih.gov/pubmed/21480387) | (2011) Dyskerin is required for tumor cell growth through mechanisms that are independent of its role in telomerase and only partially related to its function in precursor rRNA processing. | 4 of 16 | 3.53e-05 |
| [PMID:20537126](https://www.ncbi.nlm.nih.gov/pubmed/20537126) | (2010) Epigallocatechin-3-gallate suppresses the expression of HSP70 and HSP90 and exhibits anti-tumor activity in vitro and in vivo. | 4 of 16 | 3.53e-05 |
| [PMID:20423331](https://www.ncbi.nlm.nih.gov/pubmed/20423331) | (2010) SnoRNA microarray analysis reveals changes in HACA and CD RNA levels caused by dyskerin ablation in mouse liver. | 4 of 16 | 3.53e-05 |
| [PMID:16526958](https://www.ncbi.nlm.nih.gov/pubmed/16526958) | (2006) Molecular phenotype of zebrafish ovarian follicle by serial analysis of gene expression and proteomic profiling, and comparison with the transcriptomes of other animals. | 5 of 54 | 3.53e-05 |
| [PMID:30890939](https://www.ncbi.nlm.nih.gov/pubmed/30890939) | (2019) Biochemical Pathways Triggered by Antipsychotics in Human [corrected] Oligodendrocytes: Potential of Discovering New Treatment Targets. | 5 of 55 | 3.62e-05 |
| [PMID:30894069](https://www.ncbi.nlm.nih.gov/pubmed/30894069) | (2019) Impaired TFEB-mediated lysosomal biogenesis promotes the development of pancreatitis in mice and is associated with human pancreatitis. | 4 of 17 | 3.72e-05 |
| [PMID:30805011](https://www.ncbi.nlm.nih.gov/pubmed/30805011) | (2019) A Liquid Chromatography with Tandem Mass Spectrometry-Based Proteomic Analysis of Primary Cultured Cells and Subcultured Cells Using Mouse Adipose-Derived Mesenchymal Stem Cells. | 6 of 124 | 3.72e-05 |
| [PMID:28704482](https://www.ncbi.nlm.nih.gov/pubmed/28704482) | (2017) Proteomic identification of proteins differentially expressed following overexpression of hTERT (human telomerase reverse transcriptase) in cancer cells. | 4 of 17 | 3.72e-05 |
| [PMID:28630480](https://www.ncbi.nlm.nih.gov/pubmed/28630480) | (2017) Pro-invasive stimuli and the interacting protein Hsp70 favour the route of alpha-enolase to the cell surface. | 4 of 17 | 3.72e-05 |
| [PMID:27852045](https://www.ncbi.nlm.nih.gov/pubmed/27852045) | (2016) Different BCRAbl protein suppression patterns as a converging trait of chronic myeloid leukemia cell adaptation to energy restriction. | 4 of 17 | 3.72e-05 |
| [PMID:26647757](https://www.ncbi.nlm.nih.gov/pubmed/26647757) | (2016) Valproate attenuates diabetic nephropathy through inhibition of endoplasmic reticulum stress-induced apoptosis. | 4 of 17 | 3.72e-05 |
| [PMID:26871690](https://www.ncbi.nlm.nih.gov/pubmed/26871690) | (2016) Effect of Dietary Restriction and Subsequent Re-Alimentation on the Transcriptional Profile of Bovine Skeletal Muscle. | 5 of 57 | 3.87e-05 |
| [PMID:32102213](https://www.ncbi.nlm.nih.gov/pubmed/32102213) | (2020) Augmenting Vacuolar H+-ATPase Function Prevents Cardiomyocytes from Lipid-Overload Induced Dysfunction. | 4 of 18 | 4.10e-05 |
| [PMID:31363116](https://www.ncbi.nlm.nih.gov/pubmed/31363116) | (2019) Serum anti-EIF3A autoantibody as a potential diagnostic marker for hepatocellular carcinoma. | 4 of 18 | 4.10e-05 |
| [PMID:28333953](https://www.ncbi.nlm.nih.gov/pubmed/28333953) | (2017) Involvement of Beta- and Gamma-actin isoforms in actin cytoskeleton organization and migration abilities of bleb-forming human colon cancer cells. | 4 of 18 | 4.10e-05 |
| [PMID:28057907](https://www.ncbi.nlm.nih.gov/pubmed/28057907) | (2017) Molecular cloning and characterization of porcine ribosomal protein L21. | 4 of 18 | 4.10e-05 |
| [PMID:24816817](https://www.ncbi.nlm.nih.gov/pubmed/24816817) | (2014) Massively parallel sequencing of human urinary exosomemicrovesicle RNA reveals a predominance of non-coding RNA. | 4 of 18 | 4.10e-05 |
| [PMID:23690862](https://www.ncbi.nlm.nih.gov/pubmed/23690862) | (2013) Melatonin Suppresses the Expression of 45S Preribosomal RNA and Upstream Binding Factor and Enhances the Antitumor Activity of Puromycin in MDA-MB-231 Breast Cancer Cells. | 4 of 18 | 4.10e-05 |
| [PMID:20137074](https://www.ncbi.nlm.nih.gov/pubmed/20137074) | (2010) Identification of arginine- and lysine-methylation in the proteome of Saccharomyces cerevisiae and its functional implications. | 4 of 18 | 4.10e-05 |
| [PMID:19271284](https://www.ncbi.nlm.nih.gov/pubmed/19271284) | (2009) Hop proanthocyanidins induce apoptosis, protein carbonylation, and cytoskeleton disorganization in human colorectal adenocarcinoma cells via reactive oxygen species. | 4 of 18 | 4.10e-05 |
| [PMID:18644987](https://www.ncbi.nlm.nih.gov/pubmed/18644987) | (2008) Destabilization of ERBB2 transcripts by targeting 3' untranslated region messenger RNA associated HuR and histone deacetylase-6. | 4 of 18 | 4.10e-05 |
| [PMID:29066441](https://www.ncbi.nlm.nih.gov/pubmed/29066441) | (2017) Endoplasmic Reticulum Stress Is Associated With Autophagy and Cardiomyocyte Remodeling in Experimental and Human Atrial Fibrillation. | 4 of 19 | 4.42e-05 |
| [PMID:28275690](https://www.ncbi.nlm.nih.gov/pubmed/28275690) | (2017) Intestinal Epithelial-Specific mTORC1 Activation Enhances Intestinal Adaptation After Small Bowel Resection. | 4 of 19 | 4.42e-05 |
| [PMID:25152840](https://www.ncbi.nlm.nih.gov/pubmed/25152840) | (2014) Induction of Apoptosis in Pancreatic Cancer Cells by CDDO-Me Involves Repression of Telomerase through Epigenetic Pathways. | 4 of 19 | 4.42e-05 |
| [PMID:24651535](https://www.ncbi.nlm.nih.gov/pubmed/24651535) | (2014) Towards decrypting cryptobiosis--analyzing anhydrobiosis in the tardigrade Milnesium tardigradum using transcriptome sequencing. | 4 of 19 | 4.42e-05 |
| [PMID:20931991](https://www.ncbi.nlm.nih.gov/pubmed/20931991) | (2010) Paraoxon-induced protein expression changes to SH-SY5Y cells. | 4 of 19 | 4.42e-05 |
| [PMID:20667471](https://www.ncbi.nlm.nih.gov/pubmed/20667471) | (2010) Acute hyperglycemia rapidly stimulates VEGF mRNA translation in the kidney. Role of angiotensin type 2 receptor (AT2). | 4 of 19 | 4.42e-05 |
| [PMID:20219969](https://www.ncbi.nlm.nih.gov/pubmed/20219969) | (2010) Analysis of human small nucleolar RNAs (snoRNA) and the development of snoRNA modulator of gene expression vectors. | 4 of 19 | 4.42e-05 |
| [PMID:32267851](https://www.ncbi.nlm.nih.gov/pubmed/32267851) | (2020) Proteomic analysis of the processes leading to Madurella mycetomatis grain formation in Galleria mellonella larvae. | 5 of 62 | 4.65e-05 |
| [PMID:29396395](https://www.ncbi.nlm.nih.gov/pubmed/29396395) | (2018) Replication confers Beta cell immaturity. | 5 of 62 | 4.65e-05 |
| [PMID:32194992](https://www.ncbi.nlm.nih.gov/pubmed/32194992) | (2020) TFEB-mediated lysosomal biogenesis and lysosomal drug sequestration confer resistance to MEK inhibition in pancreatic cancer. | 4 of 20 | 4.77e-05 |
| [PMID:30951670](https://www.ncbi.nlm.nih.gov/pubmed/30951670) | (2019) Reassessment of Exosome Composition. | 5 of 63 | 4.77e-05 |
| [PMID:27158675](https://www.ncbi.nlm.nih.gov/pubmed/27158675) | (2016) Eosinophilic esophagitis-linked calpain 14 is an IL-13-induced protease that mediates esophageal epithelial barrier impairment. | 4 of 20 | 4.77e-05 |
| [PMID:26554841](https://www.ncbi.nlm.nih.gov/pubmed/26554841) | (2015) Proteome Differences in Placenta and Endometrium between Normal and Intrauterine Growth Restricted Pig Fetuses. | 5 of 63 | 4.77e-05 |
| [PMID:25723318](https://www.ncbi.nlm.nih.gov/pubmed/25723318) | (2014) Proteomic analysis of the regenerating liver following 23 partial hepatectomy in rats. | 5 of 63 | 4.77e-05 |
| [PMID:21208456](https://www.ncbi.nlm.nih.gov/pubmed/21208456) | (2011) Induction of HSPA4 and HSPA14 by NBS1 overexpression contributes to NBS1-induced in vitro metastatic and transformation activity. | 4 of 20 | 4.77e-05 |
| [PMID:21364676](https://www.ncbi.nlm.nih.gov/pubmed/21364676) | (2010) Androgens modulate autophagy and cell death via regulation of the endoplasmic reticulum chaperone glucose-regulated protein 78BiP in prostate cancer cells. | 4 of 20 | 4.77e-05 |
| [PMID:19480393](https://www.ncbi.nlm.nih.gov/pubmed/19480393) | (2009) Proteomic analysis of DNA-protein cross-linking by antitumor nitrogen mustards. | 4 of 20 | 4.77e-05 |
| [PMID:31828325](https://www.ncbi.nlm.nih.gov/pubmed/31828325) | (2020) Functional diversity of small nucleolar RNAs. | 5 of 64 | 4.86e-05 |
| [PMID:31026227](https://www.ncbi.nlm.nih.gov/pubmed/31026227) | (2019) Impaired ribosome biogenesis: mechanisms and relevance to cancer and aging. | 5 of 64 | 4.86e-05 |

**KEGG Pathways**

| *pathway* | *description* | *count in gene set* | *false discovery rate* |
| --- | --- | --- | --- |
| [mmu04145](https://www.kegg.jp/kegg-bin/show_pathway?mmu04145) | Phagosome | 6 of 165 | 6.02e-06 |
| [mmu03008](https://www.kegg.jp/kegg-bin/show_pathway?mmu03008) | Ribosome biogenesis in eukaryotes | 4 of 76 | 0.00014 |
| [mmu04966](https://www.kegg.jp/kegg-bin/show_pathway?mmu04966) | Collecting duct acid secretion | 3 of 27 | 0.00022 |
| [mmu04721](https://www.kegg.jp/kegg-bin/show_pathway?mmu04721) | Synaptic vesicle cycle | 3 of 62 | 0.0018 |
| [mmu05323](https://www.kegg.jp/kegg-bin/show_pathway?mmu05323) | Rheumatoid arthritis | 3 of 81 | 0.0030 |
| [mmu04540](https://www.kegg.jp/kegg-bin/show_pathway?mmu04540) | Gap junction | 3 of 85 | 0.0030 |
| [mmu04066](https://www.kegg.jp/kegg-bin/show_pathway?mmu04066) | HIF-1 signaling pathway | 3 of 102 | 0.0042 |
| [mmu03010](https://www.kegg.jp/kegg-bin/show_pathway?mmu03010) | Ribosome | 3 of 128 | 0.0070 |
| [mmu00190](https://www.kegg.jp/kegg-bin/show_pathway?mmu00190) | Oxidative phosphorylation | 3 of 129 | 0.0070 |
| [mmu04921](https://www.kegg.jp/kegg-bin/show_pathway?mmu04921) | Oxytocin signaling pathway | 3 of 149 | 0.0086 |
| [mmu04150](https://www.kegg.jp/kegg-bin/show_pathway?mmu04150) | mTOR signaling pathway | 3 of 152 | 0.0086 |
| [mmu05203](https://www.kegg.jp/kegg-bin/show_pathway?mmu05203) | Viral carcinogenesis | 3 of 199 | 0.0162 |
| [mmu00010](https://www.kegg.jp/kegg-bin/show_pathway?mmu00010) | Glycolysis / Gluconeogenesis | 2 of 65 | 0.0233 |
| [mmu04971](https://www.kegg.jp/kegg-bin/show_pathway?mmu04971) | Gastric acid secretion | 2 of 72 | 0.0263 |
| [mmu01230](https://www.kegg.jp/kegg-bin/show_pathway?mmu01230) | Biosynthesis of amino acids | 2 of 75 | 0.0265 |
| [mmu04612](https://www.kegg.jp/kegg-bin/show_pathway?mmu04612) | Antigen processing and presentation | 2 of 78 | 0.0268 |
| [mmu04914](https://www.kegg.jp/kegg-bin/show_pathway?mmu04914) | Progesterone-mediated oocyte maturation | 2 of 90 | 0.0331 |
| [mmu04670](https://www.kegg.jp/kegg-bin/show_pathway?mmu04670) | Leukocyte transendothelial migration | 2 of 115 | 0.0497 |
| [mmu01200](https://www.kegg.jp/kegg-bin/show_pathway?mmu01200) | Carbon metabolism | 2 of 118 | 0.0497 |

**Reactome Pathways**

| *pathway* | *description* | *count in gene set* | *false discovery rate* |
| --- | --- | --- | --- |
| [MMU-168256](https://reactome.org/content/detail/R-MMU-168256) | Immune System | 11 of 1523 | 0.00016 |
| [MMU-168249](https://reactome.org/content/detail/R-MMU-168249) | Innate Immune System | 9 of 879 | 0.00016 |
| [MMU-917977](https://reactome.org/content/detail/R-MMU-917977) | Transferrin endocytosis and recycling | 3 of 30 | 0.00043 |
| [MMU-77387](https://reactome.org/content/detail/R-MMU-77387) | Insulin receptor recycling | 3 of 27 | 0.00043 |
| [MMU-1222556](https://reactome.org/content/detail/R-MMU-1222556) | ROS, RNS production in phagocytes | 3 of 32 | 0.00043 |
| [MMU-917937](https://reactome.org/content/detail/R-MMU-917937) | Iron uptake and transport | 3 of 50 | 0.0012 |
| [MMU-9006934](https://reactome.org/content/detail/R-MMU-9006934) | Signaling by Receptor Tyrosine Kinases | 5 of 360 | 0.0022 |
| [MMU-74752](https://reactome.org/content/detail/R-MMU-74752) | Signaling by Insulin receptor | 3 of 66 | 0.0022 |
| [MMU-380320](https://reactome.org/content/detail/R-MMU-380320) | Recruitment of NuMA to mitotic centrosomes | 3 of 82 | 0.0033 |
| [MMU-8868773](https://reactome.org/content/detail/R-MMU-8868773) | rRNA processing in the nucleus and cytosol | 3 of 102 | 0.0056 |
| [MMU-72312](https://reactome.org/content/detail/R-MMU-72312) | rRNA processing | 3 of 102 | 0.0056 |
| [MMU-6798695](https://reactome.org/content/detail/R-MMU-6798695) | Neutrophil degranulation | 5 of 476 | 0.0056 |
| [MMU-6791226](https://reactome.org/content/detail/R-MMU-6791226) | Major pathway of rRNA processing in the nucleolus and cytosol | 3 of 102 | 0.0056 |
| [MMU-5626467](https://reactome.org/content/detail/R-MMU-5626467) | RHO GTPases activate IQGAPs | 2 of 25 | 0.0068 |
| [MMU-8854214](https://reactome.org/content/detail/R-MMU-8854214) | TBC/RABGAPs | 2 of 29 | 0.0084 |
| [MMU-437239](https://reactome.org/content/detail/R-MMU-437239) | Recycling pathway of L1 | 2 of 33 | 0.0101 |
| [MMU-3928662](https://reactome.org/content/detail/R-MMU-3928662) | EPHB-mediated forward signaling | 2 of 34 | 0.0101 |
| [MMU-983712](https://reactome.org/content/detail/R-MMU-983712) | Ion channel transport | 3 of 159 | 0.0110 |
| [MMU-190828](https://reactome.org/content/detail/R-MMU-190828) | Gap junction trafficking | 2 of 37 | 0.0110 |
| [MMU-157858](https://reactome.org/content/detail/R-MMU-157858) | Gap junction trafficking and regulation | 2 of 39 | 0.0110 |
| [MMU-69275](https://reactome.org/content/detail/R-MMU-69275) | G2/M Transition | 3 of 169 | 0.0112 |
| [MMU-5617833](https://reactome.org/content/detail/R-MMU-5617833) | Cilium Assembly | 3 of 174 | 0.0112 |
| [MMU-453274](https://reactome.org/content/detail/R-MMU-453274) | Mitotic G2-G2/M phases | 3 of 171 | 0.0112 |
| [MMU-68877](https://reactome.org/content/detail/R-MMU-68877) | Mitotic Prometaphase | 3 of 179 | 0.0116 |
| [MMU-3371497](https://reactome.org/content/detail/R-MMU-3371497) | HSP90 chaperone cycle for steroid hormone receptors (SHR) | 2 of 48 | 0.0131 |
| [MMU-1852241](https://reactome.org/content/detail/R-MMU-1852241) | Organelle biogenesis and maintenance | 3 of 199 | 0.0144 |
| [MMU-2029482](https://reactome.org/content/detail/R-MMU-2029482) | Regulation of actin dynamics for phagocytic cup formation | 2 of 54 | 0.0152 |
| [MMU-8953854](https://reactome.org/content/detail/R-MMU-8953854) | Metabolism of RNA | 4 of 448 | 0.0154 |
| [MMU-8854518](https://reactome.org/content/detail/R-MMU-8854518) | AURKA Activation by TPX2 | 2 of 67 | 0.0190 |
| [MMU-8852276](https://reactome.org/content/detail/R-MMU-8852276) | The role of GTSE1 in G2/M progression after G2 checkpoint | 2 of 68 | 0.0190 |
| [MMU-446728](https://reactome.org/content/detail/R-MMU-446728) | Cell junction organization | 2 of 63 | 0.0190 |
| [MMU-380284](https://reactome.org/content/detail/R-MMU-380284) | Loss of proteins required for interphase microtubule organization from the centrosome | 2 of 64 | 0.0190 |
| [MMU-380259](https://reactome.org/content/detail/R-MMU-380259) | Loss of Nlp from mitotic centrosomes | 2 of 64 | 0.0190 |
| [MMU-373760](https://reactome.org/content/detail/R-MMU-373760) | L1CAM interactions | 2 of 64 | 0.0190 |
| [MMU-2682334](https://reactome.org/content/detail/R-MMU-2682334) | EPH-Ephrin signaling | 2 of 63 | 0.0190 |
| [MMU-422475](https://reactome.org/content/detail/R-MMU-422475) | Axon guidance | 3 of 248 | 0.0192 |
| [MMU-380287](https://reactome.org/content/detail/R-MMU-380287) | Centrosome maturation | 2 of 73 | 0.0197 |
| [MMU-380270](https://reactome.org/content/detail/R-MMU-380270) | Recruitment of mitotic centrosome proteins and complexes | 2 of 73 | 0.0197 |
| [MMU-2029480](https://reactome.org/content/detail/R-MMU-2029480) | Fcgamma receptor (FCGR) dependent phagocytosis | 2 of 74 | 0.0197 |
| [MMU-199991](https://reactome.org/content/detail/R-MMU-199991) | Membrane Trafficking | 4 of 523 | 0.0197 |
| [MMU-3371556](https://reactome.org/content/detail/R-MMU-3371556) | Cellular response to heat stress | 2 of 78 | 0.0201 |
| [MMU-2565942](https://reactome.org/content/detail/R-MMU-2565942) | Regulation of PLK1 Activity at G2/M Transition | 2 of 80 | 0.0206 |
| [MMU-5653656](https://reactome.org/content/detail/R-MMU-5653656) | Vesicle-mediated transport | 4 of 553 | 0.0211 |
| [MMU-1500931](https://reactome.org/content/detail/R-MMU-1500931) | Cell-Cell communication | 2 of 84 | 0.0216 |
| [MMU-5620912](https://reactome.org/content/detail/R-MMU-5620912) | Anchoring of the basal body to the plasma membrane | 2 of 88 | 0.0226 |
| [MMU-4420097](https://reactome.org/content/detail/R-MMU-4420097) | VEGFA-VEGFR2 Pathway | 2 of 87 | 0.0226 |
| [MMU-194138](https://reactome.org/content/detail/R-MMU-194138) | Signaling by VEGF | 2 of 94 | 0.0250 |
| [MMU-9007101](https://reactome.org/content/detail/R-MMU-9007101) | Rab regulation of trafficking | 2 of 101 | 0.0281 |
| [MMU-68886](https://reactome.org/content/detail/R-MMU-68886) | M Phase | 3 of 317 | 0.0281 |
| [MMU-2262752](https://reactome.org/content/detail/R-MMU-2262752) | Cellular responses to stress | 3 of 327 | 0.0294 |
| [MMU-392499](https://reactome.org/content/detail/R-MMU-392499) | Metabolism of proteins | 6 of 1497 | 0.0356 |
| [MMU-5663220](https://reactome.org/content/detail/R-MMU-5663220) | RHO GTPases Activate Formins | 2 of 125 | 0.0387 |
| [MMU-8953897](https://reactome.org/content/detail/R-MMU-8953897) | Cellular responses to external stimuli | 3 of 382 | 0.0422 |

**UniProt Keywords**

| *keyword* | *description* | *count in gene set* | *false discovery rate* |
| --- | --- | --- | --- |
| [KW-0832](https://www.uniprot.org/keywords/KW-0832) | Ubl conjugation | 16 of 2091 | 3.62e-08 |
| [KW-0488](https://www.uniprot.org/keywords/KW-0488) | Methylation | 12 of 922 | 3.62e-08 |
| [KW-1017](https://www.uniprot.org/keywords/KW-1017) | Isopeptide bond | 13 of 1442 | 1.67e-07 |
| [KW-0687](https://www.uniprot.org/keywords/KW-0687) | Ribonucleoprotein | 7 of 281 | 1.13e-06 |
| [KW-0007](https://www.uniprot.org/keywords/KW-0007) | Acetylation | 16 of 3060 | 1.80e-06 |
| [KW-0547](https://www.uniprot.org/keywords/KW-0547) | Nucleotide-binding | 12 of 1738 | 7.33e-06 |
| [KW-0597](https://www.uniprot.org/keywords/KW-0597) | Phosphoprotein | 22 of 7545 | 2.35e-05 |
| [KW-0690](https://www.uniprot.org/keywords/KW-0690) | Ribosome biogenesis | 4 of 74 | 2.80e-05 |
| [KW-0342](https://www.uniprot.org/keywords/KW-0342) | GTP-binding | 6 of 324 | 2.80e-05 |
| [KW-0963](https://www.uniprot.org/keywords/KW-0963) | Cytoplasm | 16 of 4690 | 0.00030 |
| [KW-0375](https://www.uniprot.org/keywords/KW-0375) | Hydrogen ion transport | 3 of 48 | 0.00030 |
| [KW-0251](https://www.uniprot.org/keywords/KW-0251) | Elongation factor | 2 of 15 | 0.0014 |
| [KW-0324](https://www.uniprot.org/keywords/KW-0324) | Glycolysis | 2 of 32 | 0.0053 |
| [KW-0944](https://www.uniprot.org/keywords/KW-0944) | Nitration | 2 of 42 | 0.0082 |
| [KW-0689](https://www.uniprot.org/keywords/KW-0689) | Ribosomal protein | 3 of 182 | 0.0088 |
| [KW-0702](https://www.uniprot.org/keywords/KW-0702) | S-nitrosylation | 2 of 52 | 0.0108 |
| [KW-0206](https://www.uniprot.org/keywords/KW-0206) | Cytoskeleton | 6 of 1159 | 0.0141 |
| [KW-0698](https://www.uniprot.org/keywords/KW-0698) | rRNA processing | 2 of 84 | 0.0239 |
| [KW-0067](https://www.uniprot.org/keywords/KW-0067) | ATP-binding | 6 of 1352 | 0.0267 |
| [KW-0539](https://www.uniprot.org/keywords/KW-0539) | Nucleus | 12 of 4624 | 0.0301 |
| [KW-0810](https://www.uniprot.org/keywords/KW-0810) | Translation regulation | 2 of 110 | 0.0337 |
| [KW-0694](https://www.uniprot.org/keywords/KW-0694) | RNA-binding | 4 of 647 | 0.0337 |
| [KW-0379](https://www.uniprot.org/keywords/KW-0379) | Hydroxylation | 2 of 120 | 0.0367 |

**PFAM Protein Domains**

| *domain* | *description* | *count in gene set* | *false discovery rate* |
| --- | --- | --- | --- |
| [PF02874](https://pfam.xfam.org/family/PF02874) | ATP synthase alpha/beta family, beta-barrel domain | 2 of 5 | 0.0016 |
| [PF00006](https://pfam.xfam.org/family/PF00006) | ATP synthase alpha/beta family, nucleotide-binding domain | 2 of 5 | 0.0016 |
| [PF03144](https://pfam.xfam.org/family/PF03144) | Elongation factor Tu domain 2 | 2 of 14 | 0.0030 |
| [PF03953](https://pfam.xfam.org/family/PF03953) | Tubulin C-terminal domain | 2 of 20 | 0.0043 |
| [PF00091](https://pfam.xfam.org/family/PF00091) | Tubulin/FtsZ family, GTPase domain | 2 of 22 | 0.0043 |
| [PF00009](https://pfam.xfam.org/family/PF00009) | Elongation factor Tu GTP binding domain | 2 of 23 | 0.0043 |

**INTERPRO Protein Domains and Features**

| *domain* | *description* | *count in gene set* | *false discovery rate* |
| --- | --- | --- | --- |
| [IPR009000](https://www.ebi.ac.uk/interpro/entry/IPR009000) | Translation protein, beta-barrel domain superfamily | 3 of 30 | 0.0010 |
| [IPR031157](https://www.ebi.ac.uk/interpro/entry/IPR031157) | Tr-type G domain, conserved site | 2 of 9 | 0.0018 |
| [IPR027417](https://www.ebi.ac.uk/interpro/entry/IPR027417) | P-loop containing nucleoside triphosphate hydrolase | 7 of 877 | 0.0018 |
| [IPR020003](https://www.ebi.ac.uk/interpro/entry/IPR020003) | ATPase, alpha/beta subunit, nucleotide-binding domain, active site | 2 of 5 | 0.0018 |
| [IPR004100](https://www.ebi.ac.uk/interpro/entry/IPR004100) | ATPase, F1/V1/A1 complex, alpha/beta subunit, N-terminal domain | 2 of 5 | 0.0018 |
| [IPR000194](https://www.ebi.ac.uk/interpro/entry/IPR000194) | ATPase, F1/V1/A1 complex, alpha/beta subunit, nucleotide-binding domain | 2 of 5 | 0.0018 |
| [IPR004161](https://www.ebi.ac.uk/interpro/entry/IPR004161) | Translation elongation factor EFTu-like, domain 2 | 2 of 14 | 0.0028 |
| [IPR037103](https://www.ebi.ac.uk/interpro/entry/IPR037103) | Tubulin/FtsZ, C-terminal domain superfamily | 2 of 17 | 0.0035 |
| [IPR036525](https://www.ebi.ac.uk/interpro/entry/IPR036525) | Tubulin/FtsZ, GTPase domain superfamily | 2 of 20 | 0.0035 |
| [IPR023123](https://www.ebi.ac.uk/interpro/entry/IPR023123) | Tubulin, C-terminal | 2 of 19 | 0.0035 |
| [IPR018316](https://www.ebi.ac.uk/interpro/entry/IPR018316) | Tubulin/FtsZ, 2-layer sandwich domain | 2 of 18 | 0.0035 |
| [IPR017975](https://www.ebi.ac.uk/interpro/entry/IPR017975) | Tubulin, conserved site | 2 of 19 | 0.0035 |
| [IPR008280](https://www.ebi.ac.uk/interpro/entry/IPR008280) | Tubulin/FtsZ, C-terminal | 2 of 19 | 0.0035 |
| [IPR003008](https://www.ebi.ac.uk/interpro/entry/IPR003008) | Tubulin/FtsZ, GTPase domain | 2 of 19 | 0.0035 |
| [IPR000795](https://www.ebi.ac.uk/interpro/entry/IPR000795) | Transcription factor, GTP-binding domain | 2 of 19 | 0.0035 |
| [IPR000217](https://www.ebi.ac.uk/interpro/entry/IPR000217) | Tubulin | 2 of 19 | 0.0035 |
| [IPR020568](https://www.ebi.ac.uk/interpro/entry/IPR020568) | Ribosomal protein S5 domain 2-type fold | 2 of 34 | 0.0060 |
| [IPR005225](https://www.ebi.ac.uk/interpro/entry/IPR005225) | Small GTP-binding protein domain | 3 of 171 | 0.0082 |
