# Supplementary material for: TBC1D10C is a cytoskeletal functional linker that modulates cell spreading and phagocytosis in macrophages
Source: Sci Rep. 2021 Oct 22;11:20946. doi: 10.1038/s41598-021-00450-z (PMC8536695; doi:10.1038/s41598-021-00450-z)
Supplement: Supplementary file 3 — Supplementary Information 3. [file 41598_2021_450_MOESM3_ESM.docx]

**Suppl Table 2.** List of Rab35-interacting proteins in samples from Raw 264.7 macrophages.

| UniProt ID | Protein name | Description | MW (kDa) | Scores  (Mascot) | #Peptides | SC (%) |
| --- | --- | --- | --- | --- | --- | --- |
| P25911 | LYN_MOUSE | Tyrosine-protein kinase Lyn | 58.8 | 285.0 | 5 | 10.7 |
| Q9DAJ4 | WDR83_MOUSE | WD repeat domain-containing protein 83 | 34.4 | 244.0 | 3 | 11.7 |
| Q9DC51 | GNAI3_MOUSE | Guanine nucleotide-binding protein G(k) subunit alpha | 40.5 | 241.7 | 7 | 17.5 |
| Q8BG79 | C19L2_MOUSE | CWF19-like protein 2 | 103.1 | 173.0 | 6 | 8.3 |
| Q9Z1G4 | VPP1_MOUSE | V-type proton ATPase 116 kDa subunit a isoform 1 | 96.4 | 143.8 | 3 | 4.2 |
| P58242 | ASM3B_MOUSE | Acid sphingomyelinase-like phosphodiesterase 3b | 51.6 | 133.7 | 3 | 9.6 |
| Q8R2Q8 | BST2_MOUSE | Bone marrow stromal antigen 2 | 19.1 | 132.8 | 3 | 16.9 |
| P60335 | PCBP1_MOUSE | Poly(rC)-binding protein 1 | 37.5 | 112.5 | 3 | 11.8 |
| Q52KI8 | SRRM1_MOUSE | Serine/arginine repetitive matrix protein 1 | 106.8 | 104.2 | 2 | 3.0 |
| O08602 | RAE1A_MOUSE | Retinoic acid early-inducible protein 1-alpha | 28.6 | 102.1 | 2 | 7.5 |
| Q5SUA5 | MYO1G_MOUSE | Unconventional myosin-Ig | 117.2 | 99.8 | 3 | 3.5 |
| O09044 | SNP23_MOUSE | Synaptosomal-associated protein 23 | 23.2 | 85.4 | 3 | 12.9 |
| P15379 | CD44_MOUSE | CD44 antigen | 85.6 | 80.4 | 2 | 2.4 |
| P27659 | RL3_MOUSE | 60S ribosomal protein L3 | 46.1 | 240.3 | 8 | 18.9 |
| P62754 | RS6_MOUSE | 40S ribosomal protein S6 | 28.7 | 187.4 | 3 | 14.1 |
| P10126 | EF1A1_MOUSE | Elongation factor 1-alpha 1 | 50.1 | 166.5 | 4 | 8.7 |
| Q9D8E6 | RL4_MOUSE | 60S ribosomal protein L4 | 47.1 | 98.7 | 2 | 6.7 |
| P84099 | RL19_MOUSE | 60S ribosomal protein L19 | 23.5 | 95.0 | 2 | 8.7 |
| B2RY56 | RBM25_MOUSE | RNA-binding protein 25 | 99.5 | 395.5 | 7 | 11.2 |
| Q6NSQ7 | LTV1_MOUSE | Protein LTV1 homolog | 54.0 | 237.1 | 4 | 11.5 |
| O08784 | TCOF_MOUSE | Treacle protein | 134.9 | 224.4 | 7 | 6.7 |
| Q9CQW9 | IFM3_MOUSE | Interferon-induced transmembrane protein 3 | 14.9 | 93.8 | 1 | 14.6 |
| P50516 | VATA_MOUSE | V-type proton ATPase catalytic subunit A | 68.3 | 705.6 | 15 | 27.6 |
| P10810 | CD14_MOUSE | Monocyte differentiation antigen CD14 | 39.2 | 553.6 | 9 | 31.7 |
| P62814 | VATB2_MOUSE | V-type proton ATPase subunit B, brain isoform | 56.5 | 541.5 | 10 | 27.0 |
| P08752 | GNAI2_MOUSE | Guanine nucleotide-binding protein G(i) subunit alpha-2 | 40.5 | 516.3 | 9 | 30.7 |
| P51863 | VA0D1_MOUSE | V-type proton ATPase subunit d 1 | 40.3 | 512.8 | 9 | 33.6 |
| P60710 | ACTB_MOUSE | Actin, cytoplasmic 1 | 41.7 | 237.4 | 5 | 17.3 |
| P54116 | STOM_MOUSE | Erythrocyte band 7 integral membrane protein | 31.4 | 198.7 | 4 | 18.3 |
| P50518 | VATE1_MOUSE | V-type proton ATPase subunit E 1 | 26.1 | 197.0 | 5 | 22.1 |
| P63082 | VATL_MOUSE | V-type proton ATPase 16 kDa proteolipid subunit | 15.8 | 171.8 | 3 | 11.6 |
| Q9JIY5 | HTRA2_MOUSE | Serine protease HTRA2, mitochondrial | 49.3 | 141.6 | 3 | 8.5 |
| Q9Z1G3 | VATC1_MOUSE | V-type proton ATPase subunit C 1 | 43.9 | 125.3 | 2 | 6.8 |
| P57746 | VATD_MOUSE | V-type proton ATPase subunit D | 28.4 | 107.5 | 2 | 11.3 |
| P62806 | H4_MOUSE | Histone H4 | 11.4 | 105.4 | 3 | 26.2 |
| Q922Q2 | RIOK1_MOUSE | Serine/threonine-protein kinase RIO1 | 64.9 | 101.7 | 2 | 3.5 |
| Q78WZ7 | RPA43_MOUSE | DNA-directed RNA polymerase I subunit RPA43 | 36.7 | 96.4 | 2 | 7.9 |
| P45376 | ALDR_MOUSE | Aldose reductase | 35.7 | 93.3 | 2 | 7.0 |
| P62880 | GBB2_MOUSE | Guanine nucleotide-binding protein G(I)/G(S)/G(T) subunit beta-2 | 37.3 | 92.6 | 2 | 6.2 |
| P20029 | GRP78_MOUSE | 78 kDa glucose-regulated protein | 72.4 | 309.9 | 6 | 11.3 |

Conserved proteins identified between GFP-tag control vector and GFP-tag Rab35 DN were discarded (results from 4 independent experiments). *SC: Sequence coverage. Rab35 was identified but not included in the table.
