# Supplementary material for: TBC1D10C is a cytoskeletal functional linker that modulates cell spreading and phagocytosis in macrophages
Source: Sci Rep. 2021 Oct 22;11:20946. doi: 10.1038/s41598-021-00450-z (PMC8536695; doi:10.1038/s41598-021-00450-z)
Supplement: Supplementary file 4 — Supplementary Information 4. [file 41598_2021_450_MOESM4_ESM.docx]

**Supplementary Table 3.** Functional enrichments in Rab35 (DN)-interacting protein network

**Biological Process (GO)**

| *GO-term* | *description* | *count in gene set* | *false discovery rate* |
| --- | --- | --- | --- |
| [GO:0015991](http://amigo.geneontology.org/amigo/term/GO:0015991) | ATP hydrolysis coupled proton transport | 7 of 25 | 1.49e-10 |
| [GO:0070887](http://amigo.geneontology.org/amigo/term/GO:0070887) | cellular response to chemical stimulus | 15 of 2287 | 0.00084 |
| [GO:0007035](http://amigo.geneontology.org/amigo/term/GO:0007035) | vacuolar acidification | 3 of 18 | 0.0011 |
| [GO:0006812](http://amigo.geneontology.org/amigo/term/GO:0006812) | cation transport | 9 of 795 | 0.0013 |
| [GO:0071310](http://amigo.geneontology.org/amigo/term/GO:0071310) | cellular response to organic substance | 13 of 1858 | 0.0015 |
| [GO:0006810](http://amigo.geneontology.org/amigo/term/GO:0006810) | transport | 17 of 3187 | 0.0015 |
| [GO:0006811](http://amigo.geneontology.org/amigo/term/GO:0006811) | ion transport | 10 of 1156 | 0.0025 |
| [GO:0034641](http://amigo.geneontology.org/amigo/term/GO:0034641) | cellular nitrogen compound metabolic process | 19 of 4247 | 0.0029 |
| [GO:0051179](http://amigo.geneontology.org/amigo/term/GO:0051179) | localization | 19 of 4315 | 0.0034 |
| [GO:0009987](http://amigo.geneontology.org/amigo/term/GO:0009987) | cellular process | 34 of 12459 | 0.0034 |
| [GO:0035456](http://amigo.geneontology.org/amigo/term/GO:0035456) | response to interferon-beta | 3 of 37 | 0.0035 |
| [GO:0034097](http://amigo.geneontology.org/amigo/term/GO:0034097) | response to cytokine | 8 of 792 | 0.0044 |
| [GO:0033864](http://amigo.geneontology.org/amigo/term/GO:0033864) | positive regulation of NAD(P)H oxidase activity | 2 of 5 | 0.0044 |
| [GO:0010033](http://amigo.geneontology.org/amigo/term/GO:0010033) | response to organic substance | 14 of 2553 | 0.0044 |
| [GO:0002553](http://amigo.geneontology.org/amigo/term/GO:0002553) | histamine secretion by mast cell | 2 of 5 | 0.0044 |
| [GO:0002431](http://amigo.geneontology.org/amigo/term/GO:0002431) | Fc receptor mediated stimulatory signaling pathway | 2 of 5 | 0.0044 |
| [GO:0002252](http://amigo.geneontology.org/amigo/term/GO:0002252) | immune effector process | 6 of 395 | 0.0044 |
| [GO:0043603](http://amigo.geneontology.org/amigo/term/GO:0043603) | cellular amide metabolic process | 7 of 644 | 0.0060 |
| [GO:0071840](http://amigo.geneontology.org/amigo/term/GO:0071840) | cellular component organization or biogenesis | 19 of 4730 | 0.0069 |
| [GO:0042221](http://amigo.geneontology.org/amigo/term/GO:0042221) | response to chemical | 16 of 3532 | 0.0075 |
| [GO:0006955](http://amigo.geneontology.org/amigo/term/GO:0006955) | immune response | 8 of 914 | 0.0075 |
| [GO:0006412](http://amigo.geneontology.org/amigo/term/GO:0006412) | translation | 5 of 313 | 0.0094 |
| [GO:0036295](http://amigo.geneontology.org/amigo/term/GO:0036295) | cellular response to increased oxygen levels | 2 of 12 | 0.0106 |
| [GO:0045069](http://amigo.geneontology.org/amigo/term/GO:0045069) | regulation of viral genome replication | 3 of 72 | 0.0110 |
| [GO:0044085](http://amigo.geneontology.org/amigo/term/GO:0044085) | cellular component biogenesis | 12 of 2213 | 0.0110 |
| [GO:0042274](http://amigo.geneontology.org/amigo/term/GO:0042274) | ribosomal small subunit biogenesis | 3 of 70 | 0.0110 |
| [GO:0009205](http://amigo.geneontology.org/amigo/term/GO:0009205) | purine ribonucleoside triphosphate metabolic process | 4 of 187 | 0.0116 |
| [GO:0006807](http://amigo.geneontology.org/amigo/term/GO:0006807) | nitrogen compound metabolic process | 23 of 6983 | 0.0125 |
| [GO:0010941](http://amigo.geneontology.org/amigo/term/GO:0010941) | regulation of cell death | 10 of 1640 | 0.0133 |
| [GO:0009628](http://amigo.geneontology.org/amigo/term/GO:0009628) | response to abiotic stimulus | 8 of 1063 | 0.0139 |
| [GO:0055085](http://amigo.geneontology.org/amigo/term/GO:0055085) | transmembrane transport | 8 of 1073 | 0.0145 |
| [GO:0032930](http://amigo.geneontology.org/amigo/term/GO:0032930) | positive regulation of superoxide anion generation | 2 of 19 | 0.0160 |
| [GO:0009408](http://amigo.geneontology.org/amigo/term/GO:0009408) | response to heat | 3 of 94 | 0.0166 |
| [GO:0002902](http://amigo.geneontology.org/amigo/term/GO:0002902) | regulation of B cell apoptotic process | 2 of 20 | 0.0167 |
| [GO:0016241](http://amigo.geneontology.org/amigo/term/GO:0016241) | regulation of macroautophagy | 3 of 97 | 0.0175 |
| [GO:1901701](http://amigo.geneontology.org/amigo/term/GO:1901701) | cellular response to oxygen-containing compound | 7 of 870 | 0.0176 |
| [GO:0050776](http://amigo.geneontology.org/amigo/term/GO:0050776) | regulation of immune response | 6 of 635 | 0.0184 |
| [GO:0048661](http://amigo.geneontology.org/amigo/term/GO:0048661) | positive regulation of smooth muscle cell proliferation | 3 of 105 | 0.0189 |
| [GO:0044237](http://amigo.geneontology.org/amigo/term/GO:0044237) | cellular metabolic process | 23 of 7348 | 0.0189 |
| [GO:0042981](http://amigo.geneontology.org/amigo/term/GO:0042981) | regulation of apoptotic process | 9 of 1476 | 0.0189 |
| [GO:0035455](http://amigo.geneontology.org/amigo/term/GO:0035455) | response to interferon-alpha | 2 of 23 | 0.0189 |
| [GO:0002683](http://amigo.geneontology.org/amigo/term/GO:0002683) | negative regulation of immune system process | 5 of 421 | 0.0189 |
| [GO:0071363](http://amigo.geneontology.org/amigo/term/GO:0071363) | cellular response to growth factor stimulus | 5 of 437 | 0.0200 |
| [GO:0042254](http://amigo.geneontology.org/amigo/term/GO:0042254) | ribosome biogenesis | 4 of 252 | 0.0200 |
| [GO:0035809](http://amigo.geneontology.org/amigo/term/GO:0035809) | regulation of urine volume | 2 of 25 | 0.0200 |
| [GO:0006518](http://amigo.geneontology.org/amigo/term/GO:0006518) | peptide metabolic process | 5 of 440 | 0.0200 |
| [GO:0071345](http://amigo.geneontology.org/amigo/term/GO:0071345) | cellular response to cytokine stimulus | 6 of 676 | 0.0211 |
| [GO:0071353](http://amigo.geneontology.org/amigo/term/GO:0071353) | cellular response to interleukin-4 | 2 of 27 | 0.0213 |
| [GO:0006954](http://amigo.geneontology.org/amigo/term/GO:0006954) | inflammatory response | 5 of 454 | 0.0214 |
| [GO:0006360](http://amigo.geneontology.org/amigo/term/GO:0006360) | transcription by RNA polymerase I | 2 of 28 | 0.0223 |
| [GO:0009636](http://amigo.geneontology.org/amigo/term/GO:0009636) | response to toxic substance | 5 of 471 | 0.0238 |
| [GO:0051716](http://amigo.geneontology.org/amigo/term/GO:0051716) | cellular response to stimulus | 18 of 5142 | 0.0239 |
| [GO:0010467](http://amigo.geneontology.org/amigo/term/GO:0010467) | gene expression | 13 of 3013 | 0.0239 |
| [GO:0006396](http://amigo.geneontology.org/amigo/term/GO:0006396) | RNA processing | 6 of 715 | 0.0245 |
| [GO:0050896](http://amigo.geneontology.org/amigo/term/GO:0050896) | response to stimulus | 21 of 6616 | 0.0255 |
| [GO:1904707](http://amigo.geneontology.org/amigo/term/GO:1904707) | positive regulation of vascular smooth muscle cell proliferation | 2 of 32 | 0.0256 |
| [GO:0071495](http://amigo.geneontology.org/amigo/term/GO:0071495) | cellular response to endogenous stimulus | 7 of 997 | 0.0256 |
| [GO:0034122](http://amigo.geneontology.org/amigo/term/GO:0034122) | negative regulation of toll-like receptor signaling pathway | 2 of 32 | 0.0256 |
| [GO:0031663](http://amigo.geneontology.org/amigo/term/GO:0031663) | lipopolysaccharide-mediated signaling pathway | 2 of 32 | 0.0256 |
| [GO:0009719](http://amigo.geneontology.org/amigo/term/GO:0009719) | response to endogenous stimulus | 8 of 1289 | 0.0256 |
| [GO:0060548](http://amigo.geneontology.org/amigo/term/GO:0060548) | negative regulation of cell death | 7 of 1004 | 0.0258 |
| [GO:2000108](http://amigo.geneontology.org/amigo/term/GO:2000108) | positive regulation of leukocyte apoptotic process | 2 of 34 | 0.0268 |
| [GO:0016192](http://amigo.geneontology.org/amigo/term/GO:0016192) | vesicle-mediated transport | 7 of 1020 | 0.0276 |
| [GO:0071704](http://amigo.geneontology.org/amigo/term/GO:0071704) | organic substance metabolic process | 23 of 7733 | 0.0282 |
| [GO:1903707](http://amigo.geneontology.org/amigo/term/GO:1903707) | negative regulation of hemopoiesis | 3 of 143 | 0.0307 |
| [GO:0002366](http://amigo.geneontology.org/amigo/term/GO:0002366) | leukocyte activation involved in immune response | 3 of 147 | 0.0328 |
| [GO:0045087](http://amigo.geneontology.org/amigo/term/GO:0045087) | innate immune response | 5 of 534 | 0.0336 |
| [GO:0002376](http://amigo.geneontology.org/amigo/term/GO:0002376) | immune system process | 9 of 1703 | 0.0336 |
| [GO:1901564](http://amigo.geneontology.org/amigo/term/GO:1901564) | organonitrogen compound metabolic process | 16 of 4480 | 0.0346 |
| [GO:1901998](http://amigo.geneontology.org/amigo/term/GO:1901998) | toxin transport | 2 of 42 | 0.0348 |
| [GO:1901360](http://amigo.geneontology.org/amigo/term/GO:1901360) | organic cyclic compound metabolic process | 15 of 4057 | 0.0348 |
| [GO:0045071](http://amigo.geneontology.org/amigo/term/GO:0045071) | negative regulation of viral genome replication | 2 of 41 | 0.0348 |
| [GO:0035690](http://amigo.geneontology.org/amigo/term/GO:0035690) | cellular response to drug | 4 of 325 | 0.0348 |
| [GO:0006952](http://amigo.geneontology.org/amigo/term/GO:0006952) | defense response | 7 of 1079 | 0.0348 |
| [GO:0010976](http://amigo.geneontology.org/amigo/term/GO:0010976) | positive regulation of neuron projection development | 4 of 333 | 0.0355 |
| [GO:0002443](http://amigo.geneontology.org/amigo/term/GO:0002443) | leukocyte mediated immunity | 3 of 158 | 0.0355 |
| [GO:0065007](http://amigo.geneontology.org/amigo/term/GO:0065007) | biological regulation | 27 of 10168 | 0.0363 |
| [GO:0046034](http://amigo.geneontology.org/amigo/term/GO:0046034) | ATP metabolic process | 3 of 162 | 0.0363 |
| [GO:0044238](http://amigo.geneontology.org/amigo/term/GO:0044238) | primary metabolic process | 22 of 7426 | 0.0363 |
| [GO:0034605](http://amigo.geneontology.org/amigo/term/GO:0034605) | cellular response to heat | 2 of 44 | 0.0363 |
| [GO:0016043](http://amigo.geneontology.org/amigo/term/GO:0016043) | cellular component organization | 16 of 4560 | 0.0366 |
| [GO:1901566](http://amigo.geneontology.org/amigo/term/GO:1901566) | organonitrogen compound biosynthetic process | 7 of 1122 | 0.0379 |
| [GO:1990090](http://amigo.geneontology.org/amigo/term/GO:1990090) | cellular response to nerve growth factor stimulus | 2 of 47 | 0.0388 |
| [GO:0002757](http://amigo.geneontology.org/amigo/term/GO:0002757) | immune response-activating signal transduction | 3 of 168 | 0.0388 |
| [GO:0009150](http://amigo.geneontology.org/amigo/term/GO:0009150) | purine ribonucleotide metabolic process | 4 of 356 | 0.0408 |
| [GO:0007399](http://amigo.geneontology.org/amigo/term/GO:0007399) | nervous system development | 10 of 2181 | 0.0427 |
| [GO:0051602](http://amigo.geneontology.org/amigo/term/GO:0051602) | response to electrical stimulus | 2 of 51 | 0.0430 |
| [GO:0002682](http://amigo.geneontology.org/amigo/term/GO:0002682) | regulation of immune system process | 7 of 1165 | 0.0434 |
| [GO:0071216](http://amigo.geneontology.org/amigo/term/GO:0071216) | cellular response to biotic stimulus | 3 of 180 | 0.0437 |
| [GO:2000377](http://amigo.geneontology.org/amigo/term/GO:2000377) | regulation of reactive oxygen species metabolic process | 3 of 184 | 0.0457 |
| [GO:1901135](http://amigo.geneontology.org/amigo/term/GO:1901135) | carbohydrate derivative metabolic process | 6 of 882 | 0.0457 |
| [GO:0043066](http://amigo.geneontology.org/amigo/term/GO:0043066) | negative regulation of apoptotic process | 6 of 884 | 0.0457 |
| [GO:0017157](http://amigo.geneontology.org/amigo/term/GO:0017157) | regulation of exocytosis | 3 of 184 | 0.0457 |
| [GO:0002181](http://amigo.geneontology.org/amigo/term/GO:0002181) | cytoplasmic translation | 2 of 55 | 0.0460 |
| [GO:0006879](http://amigo.geneontology.org/amigo/term/GO:0006879) | cellular iron ion homeostasis | 2 of 56 | 0.0466 |
| [GO:0006397](http://amigo.geneontology.org/amigo/term/GO:0006397) | mRNA processing | 4 of 384 | 0.0466 |
| [GO:0071300](http://amigo.geneontology.org/amigo/term/GO:0071300) | cellular response to retinoic acid | 2 of 57 | 0.0473 |
| [GO:0052548](http://amigo.geneontology.org/amigo/term/GO:0052548) | regulation of endopeptidase activity | 4 of 387 | 0.0473 |
| [GO:0048699](http://amigo.geneontology.org/amigo/term/GO:0048699) | generation of neurons | 8 of 1538 | 0.0473 |
| [GO:0006725](http://amigo.geneontology.org/amigo/term/GO:0006725) | cellular aromatic compound metabolic process | 14 of 3879 | 0.0473 |
| [GO:0016072](http://amigo.geneontology.org/amigo/term/GO:0016072) | rRNA metabolic process | 3 of 196 | 0.0487 |
| [GO:0009167](http://amigo.geneontology.org/amigo/term/GO:0009167) | purine ribonucleoside monophosphate metabolic process | 3 of 196 | 0.0487 |

**Molecular Function (GO)**

| *GO-term* | *description* | *count in gene set* | *false discovery rate* |
| --- | --- | --- | --- |
| [GO:0046961](http://amigo.geneontology.org/amigo/term/GO:0046961) | proton-transporting ATPase activity, rotational mechanism | 6 of 18 | 5.43e-10 |
| [GO:0017111](http://amigo.geneontology.org/amigo/term/GO:0017111) | nucleoside-triphosphatase activity | 13 of 714 | 9.00e-09 |
| [GO:0042626](http://amigo.geneontology.org/amigo/term/GO:0042626) | ATPase activity, coupled to transmembrane movement of substances | 7 of 105 | 2.45e-08 |
| [GO:0008553](http://amigo.geneontology.org/amigo/term/GO:0008553) | proton-exporting ATPase activity, phosphorylative mechanism | 4 of 14 | 3.39e-07 |
| [GO:0016787](http://amigo.geneontology.org/amigo/term/GO:0016787) | hydrolase activity | 17 of 2259 | 1.04e-06 |
| [GO:0016887](http://amigo.geneontology.org/amigo/term/GO:0016887) | ATPase activity | 8 of 372 | 3.12e-06 |
| [GO:0097367](http://amigo.geneontology.org/amigo/term/GO:0097367) | carbohydrate derivative binding | 13 of 2051 | 0.00028 |
| [GO:0035639](http://amigo.geneontology.org/amigo/term/GO:0035639) | purine ribonucleoside triphosphate binding | 11 of 1697 | 0.0011 |
| [GO:1901363](http://amigo.geneontology.org/amigo/term/GO:1901363) | heterocyclic compound binding | 19 of 4748 | 0.0013 |
| [GO:0003735](http://amigo.geneontology.org/amigo/term/GO:0003735) | structural constituent of ribosome | 4 of 153 | 0.0013 |
| [GO:0097159](http://amigo.geneontology.org/amigo/term/GO:0097159) | organic cyclic compound binding | 19 of 4818 | 0.0014 |
| [GO:0032555](http://amigo.geneontology.org/amigo/term/GO:0032555) | purine ribonucleotide binding | 11 of 1766 | 0.0014 |
| [GO:0003824](http://amigo.geneontology.org/amigo/term/GO:0003824) | catalytic activity | 20 of 5239 | 0.0014 |
| [GO:0019843](http://amigo.geneontology.org/amigo/term/GO:0019843) | rRNA binding | 3 of 67 | 0.0016 |
| [GO:0008097](http://amigo.geneontology.org/amigo/term/GO:0008097) | 5S rRNA binding | 2 of 13 | 0.0019 |
| [GO:0005488](http://amigo.geneontology.org/amigo/term/GO:0005488) | binding | 30 of 10884 | 0.0024 |
| [GO:0003729](http://amigo.geneontology.org/amigo/term/GO:0003729) | mRNA binding | 4 of 202 | 0.0027 |
| [GO:0036094](http://amigo.geneontology.org/amigo/term/GO:0036094) | small molecule binding | 12 of 2364 | 0.0029 |
| [GO:0031683](http://amigo.geneontology.org/amigo/term/GO:0031683) | G-protein beta/gamma-subunit complex binding | 2 of 25 | 0.0052 |
| [GO:0019899](http://amigo.geneontology.org/amigo/term/GO:0019899) | enzyme binding | 11 of 2175 | 0.0052 |
| [GO:0003924](http://amigo.geneontology.org/amigo/term/GO:0003924) | GTPase activity | 4 of 255 | 0.0052 |
| [GO:0043168](http://amigo.geneontology.org/amigo/term/GO:0043168) | anion binding | 12 of 2578 | 0.0056 |
| [GO:0003723](http://amigo.geneontology.org/amigo/term/GO:0003723) | RNA binding | 7 of 986 | 0.0070 |
| [GO:0005198](http://amigo.geneontology.org/amigo/term/GO:0005198) | structural molecule activity | 5 of 546 | 0.0111 |
| [GO:0005525](http://amigo.geneontology.org/amigo/term/GO:0005525) | GTP binding | 4 of 338 | 0.0121 |
| [GO:0070063](http://amigo.geneontology.org/amigo/term/GO:0070063) | RNA polymerase binding | 2 of 52 | 0.0144 |
| [GO:0019003](http://amigo.geneontology.org/amigo/term/GO:0019003) | GDP binding | 2 of 62 | 0.0198 |
| [GO:0005546](http://amigo.geneontology.org/amigo/term/GO:0005546) | phosphatidylinositol-4,5-bisphosphate binding | 2 of 68 | 0.0231 |
| [GO:0008144](http://amigo.geneontology.org/amigo/term/GO:0008144) | drug binding | 8 of 1630 | 0.0233 |
| [GO:0005524](http://amigo.geneontology.org/amigo/term/GO:0005524) | ATP binding | 7 of 1389 | 0.0338 |
| [GO:0051117](http://amigo.geneontology.org/amigo/term/GO:0051117) | ATPase binding | 2 of 91 | 0.0380 |
| [GO:0090079](http://amigo.geneontology.org/amigo/term/GO:0090079) | translation regulator activity, nucleic acid binding | 2 of 92 | 0.0382 |
| [GO:0051219](http://amigo.geneontology.org/amigo/term/GO:0051219) | phosphoprotein binding | 2 of 98 | 0.0405 |

**Cellular Component (GO)**

| *GO-term* | *description* | *count in gene set* | *false discovery rate* |
| --- | --- | --- | --- |
| [GO:0033176](http://amigo.geneontology.org/amigo/term/GO:0033176) | proton-transporting V-type ATPase complex | 7 of 22 | 1.24e-11 |
| [GO:0016469](http://amigo.geneontology.org/amigo/term/GO:0016469) | proton-transporting two-sector ATPase complex | 8 of 46 | 1.24e-11 |
| [GO:0032991](http://amigo.geneontology.org/amigo/term/GO:0032991) | protein-containing complex | 30 of 4701 | 1.99e-11 |
| [GO:0098796](http://amigo.geneontology.org/amigo/term/GO:0098796) | membrane protein complex | 15 of 1009 | 5.63e-09 |
| [GO:0098805](http://amigo.geneontology.org/amigo/term/GO:0098805) | whole membrane | 15 of 1300 | 1.44e-07 |
| [GO:0043229](http://amigo.geneontology.org/amigo/term/GO:0043229) | intracellular organelle | 36 of 10645 | 2.77e-07 |
| [GO:0005773](http://amigo.geneontology.org/amigo/term/GO:0005773) | vacuole | 10 of 519 | 6.97e-07 |
| [GO:0033178](http://amigo.geneontology.org/amigo/term/GO:0033178) | proton-transporting two-sector ATPase complex, catalytic domain | 4 of 14 | 7.24e-07 |
| [GO:0005902](http://amigo.geneontology.org/amigo/term/GO:0005902) | microvillus | 6 of 95 | 7.24e-07 |
| [GO:0044446](http://amigo.geneontology.org/amigo/term/GO:0044446) | intracellular organelle part | 30 of 7416 | 1.03e-06 |
| [GO:0016471](http://amigo.geneontology.org/amigo/term/GO:0016471) | vacuolar proton-transporting V-type ATPase complex | 4 of 16 | 1.03e-06 |
| [GO:0044424](http://amigo.geneontology.org/amigo/term/GO:0044424) | intracellular part | 37 of 12219 | 1.16e-06 |
| [GO:1990904](http://amigo.geneontology.org/amigo/term/GO:1990904) | ribonucleoprotein complex | 11 of 765 | 1.31e-06 |
| [GO:0044444](http://amigo.geneontology.org/amigo/term/GO:0044444) | cytoplasmic part | 30 of 7673 | 1.80e-06 |
| [GO:0005774](http://amigo.geneontology.org/amigo/term/GO:0005774) | vacuolar membrane | 7 of 239 | 3.53e-06 |
| [GO:0043227](http://amigo.geneontology.org/amigo/term/GO:0043227) | membrane-bounded organelle | 33 of 9775 | 3.83e-06 |
| [GO:0005886](http://amigo.geneontology.org/amigo/term/GO:0005886) | plasma membrane | 22 of 4328 | 5.92e-06 |
| [GO:0044464](http://amigo.geneontology.org/amigo/term/GO:0044464) | cell part | 38 of 14017 | 6.60e-06 |
| [GO:0033180](http://amigo.geneontology.org/amigo/term/GO:0033180) | proton-transporting V-type ATPase, V1 domain | 3 of 7 | 7.24e-06 |
| [GO:0005764](http://amigo.geneontology.org/amigo/term/GO:0005764) | lysosome | 8 of 422 | 7.97e-06 |
| [GO:0005829](http://amigo.geneontology.org/amigo/term/GO:0005829) | cytosol | 19 of 3326 | 8.29e-06 |
| [GO:0044425](http://amigo.geneontology.org/amigo/term/GO:0044425) | membrane part | 25 of 5857 | 1.02e-05 |
| [GO:0033179](http://amigo.geneontology.org/amigo/term/GO:0033179) | proton-transporting V-type ATPase, V0 domain | 3 of 9 | 1.06e-05 |
| [GO:0043231](http://amigo.geneontology.org/amigo/term/GO:0043231) | intracellular membrane-bounded organelle | 31 of 9088 | 1.17e-05 |
| [GO:0120025](http://amigo.geneontology.org/amigo/term/GO:0120025) | plasma membrane bounded cell projection | 15 of 2172 | 1.84e-05 |
| [GO:0043209](http://amigo.geneontology.org/amigo/term/GO:0043209) | myelin sheath | 6 of 212 | 1.87e-05 |
| [GO:0005737](http://amigo.geneontology.org/amigo/term/GO:0005737) | cytoplasm | 32 of 9909 | 1.87e-05 |
| [GO:0045121](http://amigo.geneontology.org/amigo/term/GO:0045121) | membrane raft | 7 of 374 | 3.19e-05 |
| [GO:0070013](http://amigo.geneontology.org/amigo/term/GO:0070013) | intracellular organelle lumen | 19 of 3882 | 6.20e-05 |
| [GO:0045177](http://amigo.geneontology.org/amigo/term/GO:0045177) | apical part of cell | 7 of 423 | 6.20e-05 |
| [GO:0042629](http://amigo.geneontology.org/amigo/term/GO:0042629) | mast cell granule | 3 of 23 | 8.39e-05 |
| [GO:0012505](http://amigo.geneontology.org/amigo/term/GO:0012505) | endomembrane system | 18 of 3670 | 0.00011 |
| [GO:0044428](http://amigo.geneontology.org/amigo/term/GO:0044428) | nuclear part | 18 of 3798 | 0.00016 |
| [GO:0031410](http://amigo.geneontology.org/amigo/term/GO:0031410) | cytoplasmic vesicle | 12 of 1710 | 0.00016 |
| [GO:0044459](http://amigo.geneontology.org/amigo/term/GO:0044459) | plasma membrane part | 14 of 2362 | 0.00017 |
| [GO:0042470](http://amigo.geneontology.org/amigo/term/GO:0042470) | melanosome | 4 of 103 | 0.00021 |
| [GO:0005634](http://amigo.geneontology.org/amigo/term/GO:0005634) | nucleus | 23 of 6086 | 0.00022 |
| [GO:0022626](http://amigo.geneontology.org/amigo/term/GO:0022626) | cytosolic ribosome | 4 of 107 | 0.00023 |
| [GO:0000220](http://amigo.geneontology.org/amigo/term/GO:0000220) | vacuolar proton-transporting V-type ATPase, V0 domain | 2 of 5 | 0.00033 |
| [GO:0031981](http://amigo.geneontology.org/amigo/term/GO:0031981) | nuclear lumen | 16 of 3386 | 0.00053 |
| [GO:0043232](http://amigo.geneontology.org/amigo/term/GO:0043232) | intracellular non-membrane-bounded organelle | 17 of 3809 | 0.00056 |
| [GO:0022625](http://amigo.geneontology.org/amigo/term/GO:0022625) | cytosolic large ribosomal subunit | 3 of 59 | 0.00084 |
| [GO:0030688](http://amigo.geneontology.org/amigo/term/GO:0030688) | preribosome, small subunit precursor | 2 of 10 | 0.00092 |
| [GO:0031225](http://amigo.geneontology.org/amigo/term/GO:0031225) | anchored component of membrane | 4 of 163 | 0.00096 |
| [GO:0098552](http://amigo.geneontology.org/amigo/term/GO:0098552) | side of membrane | 6 of 514 | 0.0013 |
| [GO:0009898](http://amigo.geneontology.org/amigo/term/GO:0009898) | cytoplasmic side of plasma membrane | 4 of 177 | 0.0013 |
| [GO:0044391](http://amigo.geneontology.org/amigo/term/GO:0044391) | ribosomal subunit | 4 of 181 | 0.0014 |
| [GO:0016324](http://amigo.geneontology.org/amigo/term/GO:0016324) | apical plasma membrane | 5 of 339 | 0.0014 |
| [GO:0005765](http://amigo.geneontology.org/amigo/term/GO:0005765) | lysosomal membrane | 4 of 186 | 0.0015 |
| [GO:0031234](http://amigo.geneontology.org/amigo/term/GO:0031234) | extrinsic component of cytoplasmic side of plasma membrane | 3 of 111 | 0.0042 |
| [GO:0098588](http://amigo.geneontology.org/amigo/term/GO:0098588) | bounding membrane of organelle | 9 of 1513 | 0.0043 |
| [GO:0120038](http://amigo.geneontology.org/amigo/term/GO:0120038) | plasma membrane bounded cell projection part | 9 of 1532 | 0.0045 |
| [GO:0098563](http://amigo.geneontology.org/amigo/term/GO:0098563) | intrinsic component of synaptic vesicle membrane | 2 of 28 | 0.0047 |
| [GO:0005834](http://amigo.geneontology.org/amigo/term/GO:0005834) | heterotrimeric G-protein complex | 2 of 31 | 0.0056 |
| [GO:0043235](http://amigo.geneontology.org/amigo/term/GO:0043235) | receptor complex | 4 of 302 | 0.0072 |
| [GO:0031090](http://amigo.geneontology.org/amigo/term/GO:0031090) | organelle membrane | 12 of 2740 | 0.0073 |
| [GO:0099503](http://amigo.geneontology.org/amigo/term/GO:0099503) | secretory vesicle | 5 of 525 | 0.0079 |
| [GO:0005768](http://amigo.geneontology.org/amigo/term/GO:0005768) | endosome | 6 of 779 | 0.0081 |
| [GO:0044456](http://amigo.geneontology.org/amigo/term/GO:0044456) | synapse part | 6 of 809 | 0.0096 |
| [GO:0031256](http://amigo.geneontology.org/amigo/term/GO:0031256) | leading edge membrane | 3 of 161 | 0.0101 |
| [GO:0098590](http://amigo.geneontology.org/amigo/term/GO:0098590) | plasma membrane region | 7 of 1115 | 0.0104 |
| [GO:0030496](http://amigo.geneontology.org/amigo/term/GO:0030496) | midbody | 3 of 165 | 0.0104 |
| [GO:0016607](http://amigo.geneontology.org/amigo/term/GO:0016607) | nuclear speck | 4 of 362 | 0.0123 |
| [GO:0005681](http://amigo.geneontology.org/amigo/term/GO:0005681) | spliceosomal complex | 3 of 177 | 0.0124 |
| [GO:0036464](http://amigo.geneontology.org/amigo/term/GO:0036464) | cytoplasmic ribonucleoprotein granule | 3 of 182 | 0.0132 |
| [GO:0008021](http://amigo.geneontology.org/amigo/term/GO:0008021) | synaptic vesicle | 3 of 183 | 0.0132 |
| [GO:0005730](http://amigo.geneontology.org/amigo/term/GO:0005730) | nucleolus | 6 of 878 | 0.0132 |
| [GO:0043679](http://amigo.geneontology.org/amigo/term/GO:0043679) | axon terminus | 3 of 188 | 0.0140 |
| [GO:0031252](http://amigo.geneontology.org/amigo/term/GO:0031252) | cell leading edge | 4 of 393 | 0.0154 |
| [GO:0005856](http://amigo.geneontology.org/amigo/term/GO:0005856) | cytoskeleton | 9 of 1933 | 0.0167 |
| [GO:1902494](http://amigo.geneontology.org/amigo/term/GO:1902494) | catalytic complex | 7 of 1266 | 0.0179 |
| [GO:0098793](http://amigo.geneontology.org/amigo/term/GO:0098793) | presynapse | 4 of 429 | 0.0194 |
| [GO:0005844](http://amigo.geneontology.org/amigo/term/GO:0005844) | polysome | 2 of 71 | 0.0204 |
| [GO:0005794](http://amigo.geneontology.org/amigo/term/GO:0005794) | Golgi apparatus | 7 of 1313 | 0.0206 |
| [GO:0005770](http://amigo.geneontology.org/amigo/term/GO:0005770) | late endosome | 3 of 225 | 0.0206 |
| [GO:0098797](http://amigo.geneontology.org/amigo/term/GO:0098797) | plasma membrane protein complex | 4 of 461 | 0.0237 |
| [GO:0097458](http://amigo.geneontology.org/amigo/term/GO:0097458) | neuron part | 8 of 1732 | 0.0260 |
| [GO:0005758](http://amigo.geneontology.org/amigo/term/GO:0005758) | mitochondrial intermembrane space | 2 of 83 | 0.0260 |
| [GO:0005913](http://amigo.geneontology.org/amigo/term/GO:0005913) | cell-cell adherens junction | 2 of 84 | 0.0261 |
| [GO:0030863](http://amigo.geneontology.org/amigo/term/GO:0030863) | cortical cytoskeleton | 2 of 85 | 0.0264 |
| [GO:0005813](http://amigo.geneontology.org/amigo/term/GO:0005813) | centrosome | 4 of 481 | 0.0264 |
| [GO:0009986](http://amigo.geneontology.org/amigo/term/GO:0009986) | cell surface | 5 of 796 | 0.0315 |
| [GO:0005912](http://amigo.geneontology.org/amigo/term/GO:0005912) | adherens junction | 3 of 276 | 0.0316 |
| [GO:0044430](http://amigo.geneontology.org/amigo/term/GO:0044430) | cytoskeletal part | 7 of 1460 | 0.0317 |
| [GO:0005654](http://amigo.geneontology.org/amigo/term/GO:0005654) | nucleoplasm | 10 of 2648 | 0.0352 |
| [GO:0031902](http://amigo.geneontology.org/amigo/term/GO:0031902) | late endosome membrane | 2 of 115 | 0.0420 |
| [GO:0031300](http://amigo.geneontology.org/amigo/term/GO:0031300) | intrinsic component of organelle membrane | 3 of 312 | 0.0420 |
| [GO:0043195](http://amigo.geneontology.org/amigo/term/GO:0043195) | terminal bouton | 2 of 116 | 0.0423 |
| [GO:0031253](http://amigo.geneontology.org/amigo/term/GO:0031253) | cell projection membrane | 3 of 316 | 0.0423 |

**Reference publications**

| *publication* | *(year) title* | *count in gene set* | *false discovery rate* |
| --- | --- | --- | --- |
| [PMID:32194992](https://www.ncbi.nlm.nih.gov/pubmed/32194992) | (2020) TFEB-mediated lysosomal biogenesis and lysosomal drug sequestration confer resistance to MEK inhibition in pancreatic cancer. | 5 of 20 | 9.58e-05 |
| [PMID:30395881](https://www.ncbi.nlm.nih.gov/pubmed/30395881) | (2019) The protein interaction networks of mucolipins and two-pore channels. | 7 of 105 | 9.58e-05 |
| [PMID:30555553](https://www.ncbi.nlm.nih.gov/pubmed/30555553) | (2018) V-ATPases and osteoclasts: ambiguous future of V-ATPases inhibitors in osteoporosis. | 6 of 50 | 9.58e-05 |
| [PMID:27519690](https://www.ncbi.nlm.nih.gov/pubmed/27519690) | (2016) Systematic identification of genes involved in metabolic acid stress resistance in yeast and their potential as cancer targets. | 5 of 22 | 9.58e-05 |
| [PMID:26442671](https://www.ncbi.nlm.nih.gov/pubmed/26442671) | (2015) Mapping the H(+) (V)-ATPase interactome: identification of proteins involved in trafficking, folding, assembly and phosphorylation. | 6 of 51 | 9.58e-05 |
| [PMID:25948753](https://www.ncbi.nlm.nih.gov/pubmed/25948753) | (2015) Regulation of lipid droplet dynamics in Saccharomyces cerevisiae depends on the Rab7-like Ypt7p, HOPS complex and V1-ATPase. | 5 of 29 | 9.58e-05 |
| [PMID:24575049](https://www.ncbi.nlm.nih.gov/pubmed/24575049) | (2014) Role of the bicarbonate-responsive soluble adenylyl cyclase in pH sensing and metabolic regulation. | 5 of 29 | 9.58e-05 |
| [PMID:29900055](https://www.ncbi.nlm.nih.gov/pubmed/29900055) | (2018) pH regulators to target the tumor immune microenvironment in human hepatocellular carcinoma. | 5 of 33 | 0.00012 |
| [PMID:29422602](https://www.ncbi.nlm.nih.gov/pubmed/29422602) | (2018) Acidic organelles mediate TGF-Beta1-induced cellular fibrosis via (pro)renin receptor and vacuolar ATPase trafficking in human peritoneal mesothelial cells. | 5 of 34 | 0.00013 |
| [PMID:20418956](https://www.ncbi.nlm.nih.gov/pubmed/20418956) | (2010) High-content, image-based screening for drug targets in yeast. | 4 of 10 | 0.00013 |
| [PMID:23967163](https://www.ncbi.nlm.nih.gov/pubmed/23967163) | (2013) Elucidation of how cancer cells avoid acidosis through comparative transcriptomic data analysis. | 5 of 39 | 0.00019 |
| [PMID:30478388](https://www.ncbi.nlm.nih.gov/pubmed/30478388) | (2018) The interferon-inducible isoform of NCOA7 inhibits endosome-mediated viral entry. | 5 of 43 | 0.00028 |
| [PMID:29473670](https://www.ncbi.nlm.nih.gov/pubmed/29473670) | (2018) Some assembly required: Contributions of Tom Stevens' lab to the V-ATPase field. | 4 of 15 | 0.00038 |
| [PMID:24155661](https://www.ncbi.nlm.nih.gov/pubmed/24155661) | (2013) Silencing of atp6v1c1 prevents breast cancer growth and bone metastasis. | 4 of 15 | 0.00038 |
| [PMID:25866880](https://www.ncbi.nlm.nih.gov/pubmed/25866880) | (2015) Translational control of the cytosolic stress response by mitochondrial ribosomal protein L18. | 4 of 16 | 0.00041 |
| [PMID:24314139](https://www.ncbi.nlm.nih.gov/pubmed/24314139) | (2013) Reprogramming of lysosomal gene expression by interleukin-4 and Stat6. | 6 of 105 | 0.00041 |
| [PMID:32102213](https://www.ncbi.nlm.nih.gov/pubmed/32102213) | (2020) Augmenting Vacuolar H+-ATPase Function Prevents Cardiomyocytes from Lipid-Overload Induced Dysfunction. | 4 of 18 | 0.00043 |
| [PMID:31033440](https://www.ncbi.nlm.nih.gov/pubmed/31033440) | (2019) HRI coordinates translation necessary for protein homeostasis and mitochondrial function in erythropoiesis. | 5 of 53 | 0.00043 |
| [PMID:30952843](https://www.ncbi.nlm.nih.gov/pubmed/30952843) | (2019) FoxK1 and FoxK2 in insulin regulation of cellular and mitochondrial metabolism. | 6 of 113 | 0.00043 |
| [PMID:30894069](https://www.ncbi.nlm.nih.gov/pubmed/30894069) | (2019) Impaired TFEB-mediated lysosomal biogenesis promotes the development of pancreatitis in mice and is associated with human pancreatitis. | 4 of 17 | 0.00043 |
| [PMID:30647105](https://www.ncbi.nlm.nih.gov/pubmed/30647105) | (2019) Comparative Genomic Screen in Two Yeasts Reveals Conserved Pathways in the Response Network to Phenol Stress. | 4 of 18 | 0.00043 |
| [PMID:28594408](https://www.ncbi.nlm.nih.gov/pubmed/28594408) | (2017) Autophagy blockade and lysosomal membrane permeabilization contribute to lead-induced nephrotoxicity in primary rat proximal tubular cells. | 4 of 17 | 0.00043 |
| [PMID:28290485](https://www.ncbi.nlm.nih.gov/pubmed/28290485) | (2017) Complementary transcriptomic and proteomic analyses reveal regulatory mechanisms of milk protein production in dairy cows consuming different forages. | 5 of 51 | 0.00043 |
| [PMID:28053225](https://www.ncbi.nlm.nih.gov/pubmed/28053225) | (2017) The V-ATPase is expressed in the choroid plexus and mediates cAMP-induced intracellular pH alterations. | 4 of 17 | 0.00043 |
| [PMID:26586472](https://www.ncbi.nlm.nih.gov/pubmed/26586472) | (2016) V-type ATPase proton pump expression during enamel formation. | 4 of 19 | 0.00043 |
| [PMID:24223829](https://www.ncbi.nlm.nih.gov/pubmed/24223829) | (2013) The role of individual domains and the significance of shedding of ATP6AP2(pro)renin receptor in vacuolar H(+)-ATPase biogenesis. | 4 of 17 | 0.00043 |
| [PMID:23469216](https://www.ncbi.nlm.nih.gov/pubmed/23469216) | (2013) Distinct signal transduction pathways downstream of the (P)RR revealed by microarray and ChIP-chip analyses. | 5 of 58 | 0.00043 |
| [PMID:22467241](https://www.ncbi.nlm.nih.gov/pubmed/22467241) | (2012) V-ATPase subunit ATP6AP1 (Ac45) regulates osteoclast differentiation, extracellular acidification, lysosomal trafficking, and protease exocytosis in osteoclast-mediated bone resorption. | 4 of 19 | 0.00043 |
| [PMID:21804531](https://www.ncbi.nlm.nih.gov/pubmed/21804531) | (2011) Regulation of TFEB and V-ATPases by mTORC1. | 4 of 19 | 0.00043 |
| [PMID:20137074](https://www.ncbi.nlm.nih.gov/pubmed/20137074) | (2010) Identification of arginine- and lysine-methylation in the proteome of Saccharomyces cerevisiae and its functional implications. | 4 of 18 | 0.00043 |
| [PMID:11922865](https://www.ncbi.nlm.nih.gov/pubmed/11922865) | (2002) Nerve growth factor selectively regulates expression of transcripts encoding ribosomal proteins. | 4 of 19 | 0.00043 |
| [PMID:25133973](https://www.ncbi.nlm.nih.gov/pubmed/25133973) | (2014) Nuclear cytoplasmic trafficking of proteins is a major response of human fibroblasts to oxidative stress. | 6 of 123 | 0.00048 |
| [PMID:23724114](https://www.ncbi.nlm.nih.gov/pubmed/23724114) | (2013) Functional analyse of GLUT1 and GLUT12 in glucose uptake in goat mammary gland epithelial cells. | 4 of 21 | 0.00048 |
| [PMID:30875964](https://www.ncbi.nlm.nih.gov/pubmed/30875964) | (2019) Niclosamide Triggers Non-Canonical LC3 Lipidation. | 4 of 22 | 0.00055 |
| [PMID:31523176](https://www.ncbi.nlm.nih.gov/pubmed/31523176) | (2019) Ribosomal Protein L15 is involved in Colon Carcinogenesis. | 4 of 23 | 0.00063 |
| [PMID:25332393](https://www.ncbi.nlm.nih.gov/pubmed/25332393) | (2014) Ribosomal stress activates eEF2K-eEF2 pathway causing translation elongation inhibition and recruitment of terminal oligopyrimidine (TOP) mRNAs on polysomes. | 4 of 23 | 0.00063 |
| [PMID:30307711](https://www.ncbi.nlm.nih.gov/pubmed/30307711) | (2018) Quantitative proteomic analysis of intracerebral hemorrhage in rats with a focus on brain energy metabolism. | 4 of 26 | 0.00092 |
| [PMID:22194971](https://www.ncbi.nlm.nih.gov/pubmed/22194971) | (2011) Protein profile changes during porcine oocyte aging and effects of caffeine on protein expression patterns. | 4 of 26 | 0.00092 |
| [PMID:26319900](https://www.ncbi.nlm.nih.gov/pubmed/26319900) | (2015) Suppression of the GTPase-activating protein RGS10 increases Rheb-GTP and mTOR signaling in ovarian cancer cells. | 4 of 27 | 0.0010 |
| [PMID:23690912](https://www.ncbi.nlm.nih.gov/pubmed/23690912) | (2013) New model of action for mood stabilizers: phosphoproteome from rat pre-frontal cortex synaptoneurosomal preparations. | 4 of 27 | 0.0010 |
| [PMID:22829766](https://www.ncbi.nlm.nih.gov/pubmed/22829766) | (2012) A cytotoxic type III secretion effector of Vibrio parahaemolyticus targets vacuolar H+-ATPase subunit c and ruptures host cell lysosomes. | 4 of 27 | 0.0010 |
| [PMID:31189779](https://www.ncbi.nlm.nih.gov/pubmed/31189779) | (2019) Vacuolar-type ATPase: A proton pump to lysosomal trafficking. | 4 of 28 | 0.0011 |
| [PMID:29980615](https://www.ncbi.nlm.nih.gov/pubmed/29980615) | (2018) Influenza A Virus Induces Autophagosomal Targeting of Ribosomal Proteins. | 4 of 29 | 0.0011 |
| [PMID:25951193](https://www.ncbi.nlm.nih.gov/pubmed/25951193) | (2015) The integral membrane protein ITM2A, a transcriptional target of PKA-CREB, regulates autophagic flux via interaction with the vacuolar ATPase. | 4 of 28 | 0.0011 |
| [PMID:23696868](https://www.ncbi.nlm.nih.gov/pubmed/23696868) | (2013) Enhanced translation of mRNAs encoding proteins involved in mRNA translation during recovery from heat shock. | 4 of 28 | 0.0011 |
| [PMID:23077530](https://www.ncbi.nlm.nih.gov/pubmed/23077530) | (2012) Suppressed RNA-polymerase 1 pathway is associated with benign multiple sclerosis. | 4 of 30 | 0.0013 |
| [PMID:18667600](https://www.ncbi.nlm.nih.gov/pubmed/18667600) | (2008) V-ATPase expression in the mouse olfactory epithelium. | 3 of 6 | 0.0014 |
| [PMID:31285595](https://www.ncbi.nlm.nih.gov/pubmed/31285595) | (2019) Covalent targeting of the vacuolar H+-ATPase activates autophagy via mTORC1 inhibition. | 4 of 32 | 0.0015 |
| [PMID:30804932](https://www.ncbi.nlm.nih.gov/pubmed/30804932) | (2019) New Insights for RANKL as a Proinflammatory Modulator in Modeled Inflammatory Arthritis. | 4 of 32 | 0.0015 |
| [PMID:26151086](https://www.ncbi.nlm.nih.gov/pubmed/26151086) | (2015) Compensatory Islet Response to Insulin Resistance Revealed by Quantitative Proteomics. | 5 of 85 | 0.0016 |
| [PMID:31201651](https://www.ncbi.nlm.nih.gov/pubmed/31201651) | (2019) Altered Levels of Proteins and Phosphoproteins, in the Absence of Early Causative Transcriptional Changes, Shape the Molecular Pathogenesis in the Brain of Young Presymptomatic Ki91 SCA3MJD Mouse. | 7 of 277 | 0.0018 |
| [PMID:28253842](https://www.ncbi.nlm.nih.gov/pubmed/28253842) | (2017) Salicylic acid-related cotton (Gossypium arboreum) ribosomal protein GaRPL18 contributes to resistance to Verticillium dahliae. | 3 of 7 | 0.0018 |
| [PMID:26901847](https://www.ncbi.nlm.nih.gov/pubmed/26901847) | (2016) Radiotherapy diagnostic biomarkers in radioresistant human H460 lung cancer stem-like cells. | 4 of 34 | 0.0018 |
| [PMID:28994389](https://www.ncbi.nlm.nih.gov/pubmed/28994389) | (2017) Molecular architecture underlying fluid absorption by the developing inner ear. | 5 of 93 | 0.0023 |
| [PMID:25885223](https://www.ncbi.nlm.nih.gov/pubmed/25885223) | (2015) Endoplasmic reticulum chaperone GRP78 is involved in autophagy activation induced by ischemic preconditioning in neural cells. | 3 of 8 | 0.0023 |
| [PMID:25496664](https://www.ncbi.nlm.nih.gov/pubmed/25496664) | (2014) Molecular signatures that correlate with induction of lens regeneration in newts: lessons from proteomic analysis. | 5 of 93 | 0.0023 |
| [PMID:24284395](https://www.ncbi.nlm.nih.gov/pubmed/24284395) | (2013) Combination BMSC and Niaspan treatment of stroke enhances white matter remodeling and synaptic protein expression in diabetic rats. | 3 of 8 | 0.0023 |
| [PMID:31920959](https://www.ncbi.nlm.nih.gov/pubmed/31920959) | (2019) Multiomics-Based Signaling Pathway Network Alterations in Human Non-functional Pituitary Adenomas. | 6 of 186 | 0.0027 |
| [PMID:26792401](https://www.ncbi.nlm.nih.gov/pubmed/26792401) | (2016) CNS uptake of bortezomib is enhanced by P-glycoprotein inhibition: implications for spinal muscular atrophy. | 3 of 9 | 0.0029 |
| [PMID:24899231](https://www.ncbi.nlm.nih.gov/pubmed/24899231) | (2014) Conditional disruption of interactions between GAlfai2 and regulator of G protein signaling (RGS) proteins protects the heart from ischemic injury. | 3 of 9 | 0.0029 |
| [PMID:30120234](https://www.ncbi.nlm.nih.gov/pubmed/30120234) | (2018) Galectin-9 suppresses B cell receptor signaling and is regulated by I-branching of N-glycans. | 4 of 41 | 0.0030 |
| [PMID:19302708](https://www.ncbi.nlm.nih.gov/pubmed/19302708) | (2009) Transcriptional signatures of BALBc mouse macrophages housing multiplying Leishmania amazonensis amastigotes. | 5 of 102 | 0.0031 |
| [PMID:31296888](https://www.ncbi.nlm.nih.gov/pubmed/31296888) | (2019) A Differential Hypofunctionality of GAlfai Proteins Occurs in Adolescent Idiopathic Scoliosis and Correlates with the Risk of Disease Progression. | 3 of 10 | 0.0035 |
| [PMID:31149036](https://www.ncbi.nlm.nih.gov/pubmed/31149036) | (2019) GRP78-targeted ferritin nanocaged ultra-high dose of doxorubicin for hepatocellular carcinoma therapy. | 3 of 10 | 0.0035 |
| [PMID:30201806](https://www.ncbi.nlm.nih.gov/pubmed/30201806) | (2018) RACK1 Specifically Regulates Translation through Its Binding to Ribosomes. | 3 of 10 | 0.0035 |
| [PMID:30189184](https://www.ncbi.nlm.nih.gov/pubmed/30189184) | (2018) Role of hippocampal 5-HT1A receptors in the antidepressant-like phenotype of mice expressing RGS-insensitive GAlfai2 protein. | 3 of 10 | 0.0035 |
| [PMID:25993305](https://www.ncbi.nlm.nih.gov/pubmed/25993305) | (2015) MALDI-Mass Spectrometric Imaging Revealing Hypoxia-Driven Lipids and Proteins in a Breast Tumor Model. | 4 of 44 | 0.0035 |
| [PMID:24858945](https://www.ncbi.nlm.nih.gov/pubmed/24858945) | (2014) GAlfai2- and GAlfai3-deficient mice display opposite severity of myocardial ischemia reperfusion injury. | 3 of 10 | 0.0035 |
| [PMID:23434374](https://www.ncbi.nlm.nih.gov/pubmed/23434374) | (2013) ZKSCAN3 is a master transcriptional repressor of autophagy. | 4 of 43 | 0.0035 |
| [PMID:21524776](https://www.ncbi.nlm.nih.gov/pubmed/21524776) | (2011) Subcellular location and topology of severe acute respiratory syndrome coronavirus envelope protein. | 3 of 10 | 0.0035 |
| [PMID:21276872](https://www.ncbi.nlm.nih.gov/pubmed/21276872) | (2011) Uncovering the global host cell requirements for influenza virus replication via RNAi screening. | 4 of 45 | 0.0036 |
| [PMID:31223620](https://www.ncbi.nlm.nih.gov/pubmed/31223620) | (2019) Validation of Reference Genes for Gene Expression Normalization in RAW264.7 Cells under Different Conditions. | 3 of 11 | 0.0039 |
| [PMID:30442709](https://www.ncbi.nlm.nih.gov/pubmed/30442709) | (2019) PPT1 Promotes Tumor Growth and Is the Molecular Target of Chloroquine Derivatives in Cancer. | 3 of 11 | 0.0039 |
| [PMID:30009162](https://www.ncbi.nlm.nih.gov/pubmed/30009162) | (2018) Topological Characterization of Human and Mouse m5C Epitranscriptome Revealed by Bisulfite Sequencing. | 4 of 46 | 0.0039 |
| [PMID:29203145](https://www.ncbi.nlm.nih.gov/pubmed/29203145) | (2018) Global gene expression analysis of macrophage response induced by nonporous and porous silica nanoparticles. | 3 of 11 | 0.0039 |
| [PMID:27790248](https://www.ncbi.nlm.nih.gov/pubmed/27790248) | (2016) Large Scale Gene Expression Meta-Analysis Reveals Tissue-Specific, Sex-Biased Gene Expression in Humans. | 4 of 47 | 0.0039 |
| [PMID:27278128](https://www.ncbi.nlm.nih.gov/pubmed/27278128) | (2016) Phorbol ester-mediated re-expression of endogenous LAT adapter in J.CaM2 cells: a model for dissecting drivers and blockers of LAT transcription. | 3 of 11 | 0.0039 |
| [PMID:26075749](https://www.ncbi.nlm.nih.gov/pubmed/26075749) | (2015) Metformin induces ER stress-dependent apoptosis through miR-708-5pNNAT pathway in prostate cancer. | 3 of 11 | 0.0039 |
| [PMID:25050110](https://www.ncbi.nlm.nih.gov/pubmed/25050110) | (2014) GRP78 inhibits macrophage adhesion via SR-A. | 3 of 11 | 0.0039 |
| [PMID:19008951](https://www.ncbi.nlm.nih.gov/pubmed/19008951) | (2008) Key role of splenic myeloid DCs in the IFN-alphabeta response to adenoviruses in vivo. | 4 of 46 | 0.0039 |
| [PMID:18638456](https://www.ncbi.nlm.nih.gov/pubmed/18638456) | (2008) AMPK represses TOP mRNA translation but not global protein synthesis in liver. | 3 of 11 | 0.0039 |
| [PMID:30975297](https://www.ncbi.nlm.nih.gov/pubmed/30975297) | (2019) Cardioprotective Effect of the Mitochondrial Unfolded Protein Response During Chronic Pressure Overload. | 3 of 12 | 0.0043 |
| [PMID:30453691](https://www.ncbi.nlm.nih.gov/pubmed/30453691) | (2018) A Proteomic Approach for Understanding the Mechanisms of Delayed Corneal Wound Healing in Diabetic Keratopathy Using Diabetic Model Rat. | 3 of 12 | 0.0043 |
| [PMID:27835895](https://www.ncbi.nlm.nih.gov/pubmed/27835895) | (2016) Enhancement of 5-FU sensitivity by the proapoptotic rpL3 gene in p53 null colon cancer cells through combined polymer nanoparticles. | 3 of 12 | 0.0043 |
| [PMID:27016735](https://www.ncbi.nlm.nih.gov/pubmed/27016735) | (2016) Promoter architecture and transcriptional regulation of Abf1-dependent ribosomal protein genes in Saccharomyces cerevisiae. | 3 of 12 | 0.0043 |
| [PMID:25298750](https://www.ncbi.nlm.nih.gov/pubmed/25298750) | (2014) Human umbilical cord mesenchymal stem cells promote carcinoma growth and lymph node metastasis when co-injected with esophageal carcinoma cells in nude mice. | 3 of 12 | 0.0043 |
| [PMID:24828841](https://www.ncbi.nlm.nih.gov/pubmed/24828841) | (2014) Porcine endogenous retroviruses in xenotransplantation--molecular aspects. | 3 of 12 | 0.0043 |
| [PMID:22065581](https://www.ncbi.nlm.nih.gov/pubmed/22065581) | (2011) Human mutation within Per-Arnt-Sim (PAS) domain-containing protein kinase (PASK) causes basal insulin hypersecretion. | 3 of 12 | 0.0043 |
| [PMID:27078027](https://www.ncbi.nlm.nih.gov/pubmed/27078027) | (2016) Hypoxia Induces Autophagy through Translational Up-Regulation of Lysosomal Proteins in Human Colon Cancer Cells. | 4 of 51 | 0.0046 |
| [PMID:30708974](https://www.ncbi.nlm.nih.gov/pubmed/30708974) | (2019) TUDCA-Treated Mesenchymal Stem Cells Protect against ER Stress in the Hippocampus of a Murine Chronic Kidney Disease Model. | 3 of 13 | 0.0048 |
| [PMID:30135222](https://www.ncbi.nlm.nih.gov/pubmed/30135222) | (2018) Spleen Tyrosine Kinase Inhibitor TAK-659 Prevents Splenomegaly and Tumor Development in a Murine Model of Epstein-Barr Virus-Associated Lymphoma. | 3 of 13 | 0.0048 |
| [PMID:29384874](https://www.ncbi.nlm.nih.gov/pubmed/29384874) | (2018) BMP2 and VEGF165 transfection to bone marrow stromal stem cells regulate osteogenic potential in vitro. | 3 of 13 | 0.0048 |
| [PMID:27237224](https://www.ncbi.nlm.nih.gov/pubmed/27237224) | (2016) Adaptations to chronic rapamycin in mice. | 3 of 13 | 0.0048 |
| [PMID:23691483](https://www.ncbi.nlm.nih.gov/pubmed/23691483) | (2012) GNAI1 Suppresses Tumor Cell Migration and Invasion and is Post-Transcriptionally Regulated by Mir-320acd in Hepatocellular Carcinoma. | 3 of 13 | 0.0048 |
| [PMID:20126274](https://www.ncbi.nlm.nih.gov/pubmed/20126274) | (2010) An inhibitory role of the G-protein regulator AGS3 in mTOR-dependent macroautophagy. | 3 of 13 | 0.0048 |
| [PMID:30260431](https://www.ncbi.nlm.nih.gov/pubmed/30260431) | (2018) Protein Syndesmos is a novel RNA-binding protein that regulates primary cilia formation. | 4 of 54 | 0.0053 |
| [PMID:29499948](https://www.ncbi.nlm.nih.gov/pubmed/29499948) | (2018) Endogenous Cellular MicroRNAs Mediate Antiviral Defense against Influenza A Virus. | 3 of 14 | 0.0054 |
| [PMID:29054531](https://www.ncbi.nlm.nih.gov/pubmed/29054531) | (2018) The ammonia transporter RhCG modulates urinary acidification by interacting with the vacuolar proton-ATPases in renal intercalated cells. | 3 of 14 | 0.0054 |
| [PMID:27941876](https://www.ncbi.nlm.nih.gov/pubmed/27941876) | (2017) Inhibition of KPNA4 attenuates prostate cancer metastasis. | 3 of 14 | 0.0054 |
| [PMID:27001958](https://www.ncbi.nlm.nih.gov/pubmed/27001958) | (2016) Cutting Edge: Foxp1 Controls Naive CD8+ T Cell Quiescence by Simultaneously Repressing Key Pathways in Cellular Metabolism and Cell Cycle Progression. | 3 of 14 | 0.0054 |

**KEGG Pathways**

| *pathway* | *description* | *count in gene set* | *false discovery rate* |
| --- | --- | --- | --- |
| [mmu04966](https://www.kegg.jp/kegg-bin/show_pathway?mmu04966) | Collecting duct acid secretion | 8 of 27 | 1.06e-13 |
| [mmu04721](https://www.kegg.jp/kegg-bin/show_pathway?mmu04721) | Synaptic vesicle cycle | 8 of 62 | 2.03e-11 |
| [mmu04145](https://www.kegg.jp/kegg-bin/show_pathway?mmu04145) | Phagosome | 10 of 165 | 2.03e-11 |
| [mmu05323](https://www.kegg.jp/kegg-bin/show_pathway?mmu05323) | Rheumatoid arthritis | 8 of 81 | 7.42e-11 |
| [mmu00190](https://www.kegg.jp/kegg-bin/show_pathway?mmu00190) | Oxidative phosphorylation | 8 of 129 | 1.98e-09 |
| [mmu04150](https://www.kegg.jp/kegg-bin/show_pathway?mmu04150) | mTOR signaling pathway | 6 of 152 | 5.78e-06 |
| [mmu04611](https://www.kegg.jp/kegg-bin/show_pathway?mmu04611) | Platelet activation | 5 of 122 | 4.33e-05 |
| [mmu01100](https://www.kegg.jp/kegg-bin/show_pathway?mmu01100) | Metabolic pathways | 10 of 1296 | 0.00088 |
| [mmu03010](https://www.kegg.jp/kegg-bin/show_pathway?mmu03010) | Ribosome | 4 of 128 | 0.0010 |
| [mmu04730](https://www.kegg.jp/kegg-bin/show_pathway?mmu04730) | Long-term depression | 3 of 60 | 0.0020 |
| [mmu05152](https://www.kegg.jp/kegg-bin/show_pathway?mmu05152) | Tuberculosis | 4 of 172 | 0.0025 |
| [mmu05133](https://www.kegg.jp/kegg-bin/show_pathway?mmu05133) | Pertussis | 3 of 74 | 0.0028 |
| [mmu04971](https://www.kegg.jp/kegg-bin/show_pathway?mmu04971) | Gastric acid secretion | 3 of 72 | 0.0028 |
| [mmu04670](https://www.kegg.jp/kegg-bin/show_pathway?mmu04670) | Leukocyte transendothelial migration | 3 of 115 | 0.0089 |
| [mmu04142](https://www.kegg.jp/kegg-bin/show_pathway?mmu04142) | Lysosome | 3 of 123 | 0.0101 |
| [mmu04371](https://www.kegg.jp/kegg-bin/show_pathway?mmu04371) | Apelin signaling pathway | 3 of 134 | 0.0120 |
| [mmu05012](https://www.kegg.jp/kegg-bin/show_pathway?mmu05012) | Parkinson's disease | 3 of 138 | 0.0123 |
| [mmu04921](https://www.kegg.jp/kegg-bin/show_pathway?mmu04921) | Oxytocin signaling pathway | 3 of 149 | 0.0143 |
| [mmu05030](https://www.kegg.jp/kegg-bin/show_pathway?mmu05030) | Cocaine addiction | 2 of 48 | 0.0197 |
| [mmu04062](https://www.kegg.jp/kegg-bin/show_pathway?mmu04062) | Chemokine signaling pathway | 3 of 179 | 0.0214 |
| [mmu04923](https://www.kegg.jp/kegg-bin/show_pathway?mmu04923) | Regulation of lipolysis in adipocytes | 2 of 55 | 0.0230 |
| [mmu05134](https://www.kegg.jp/kegg-bin/show_pathway?mmu05134) | Legionellosis | 2 of 57 | 0.0235 |
| [mmu05205](https://www.kegg.jp/kegg-bin/show_pathway?mmu05205) | Proteoglycans in cancer | 3 of 199 | 0.0249 |
| [mmu04015](https://www.kegg.jp/kegg-bin/show_pathway?mmu04015) | Rap1 signaling pathway | 3 of 207 | 0.0266 |
| [mmu04924](https://www.kegg.jp/kegg-bin/show_pathway?mmu04924) | Renin secretion | 2 of 69 | 0.0296 |
| [mmu03008](https://www.kegg.jp/kegg-bin/show_pathway?mmu03008) | Ribosome biogenesis in eukaryotes | 2 of 76 | 0.0342 |
| [mmu05132](https://www.kegg.jp/kegg-bin/show_pathway?mmu05132) | Salmonella infection | 2 of 78 | 0.0346 |
| [mmu04540](https://www.kegg.jp/kegg-bin/show_pathway?mmu04540) | Gap junction | 2 of 85 | 0.0391 |
| [mmu04727](https://www.kegg.jp/kegg-bin/show_pathway?mmu04727) | GABAergic synapse | 2 of 87 | 0.0395 |
| [mmu05032](https://www.kegg.jp/kegg-bin/show_pathway?mmu05032) | Morphine addiction | 2 of 91 | 0.0407 |
| [mmu04914](https://www.kegg.jp/kegg-bin/show_pathway?mmu04914) | Progesterone-mediated oocyte maturation | 2 of 90 | 0.0407 |
| [mmu04713](https://www.kegg.jp/kegg-bin/show_pathway?mmu04713) | Circadian entrainment | 2 of 95 | 0.0407 |
| [mmu04640](https://www.kegg.jp/kegg-bin/show_pathway?mmu04640) | Hematopoietic cell lineage | 2 of 90 | 0.0407 |
| [mmu04064](https://www.kegg.jp/kegg-bin/show_pathway?mmu04064) | NF-kappa B signaling pathway | 2 of 93 | 0.0407 |
| [mmu04916](https://www.kegg.jp/kegg-bin/show_pathway?mmu04916) | Melanogenesis | 2 of 98 | 0.0408 |
| [mmu05142](https://www.kegg.jp/kegg-bin/show_pathway?mmu05142) | Chagas disease (American trypanosomiasis) | 2 of 101 | 0.0420 |
| [mmu05145](https://www.kegg.jp/kegg-bin/show_pathway?mmu05145) | Toxoplasmosis | 2 of 107 | 0.0455 |
| [mmu04725](https://www.kegg.jp/kegg-bin/show_pathway?mmu04725) | Cholinergic synapse | 2 of 112 | 0.0482 |
| [mmu04724](https://www.kegg.jp/kegg-bin/show_pathway?mmu04724) | Glutamatergic synapse | 2 of 113 | 0.0482 |

**Reactome Pathways**

| *pathway* | *description* | *count in gene set* | *false discovery rate* |
| --- | --- | --- | --- |
| [MMU-77387](https://reactome.org/content/detail/R-MMU-77387) | Insulin receptor recycling | 8 of 27 | 1.67e-13 |
| [MMU-917977](https://reactome.org/content/detail/R-MMU-917977) | Transferrin endocytosis and recycling | 8 of 30 | 1.72e-13 |
| [MMU-1222556](https://reactome.org/content/detail/R-MMU-1222556) | ROS, RNS production in phagocytes | 8 of 32 | 1.80e-13 |
| [MMU-917937](https://reactome.org/content/detail/R-MMU-917937) | Iron uptake and transport | 8 of 50 | 3.30e-12 |
| [MMU-74752](https://reactome.org/content/detail/R-MMU-74752) | Signaling by Insulin receptor | 8 of 66 | 2.03e-11 |
| [MMU-983712](https://reactome.org/content/detail/R-MMU-983712) | Ion channel transport | 9 of 159 | 3.33e-10 |
| [MMU-168249](https://reactome.org/content/detail/R-MMU-168249) | Innate Immune System | 14 of 879 | 3.95e-09 |
| [MMU-9006934](https://reactome.org/content/detail/R-MMU-9006934) | Signaling by Receptor Tyrosine Kinases | 10 of 360 | 1.33e-08 |
| [MMU-168256](https://reactome.org/content/detail/R-MMU-168256) | Immune System | 16 of 1523 | 4.30e-08 |
| [MMU-382551](https://reactome.org/content/detail/R-MMU-382551) | Transport of small molecules | 9 of 624 | 2.02e-05 |
| [MMU-6798695](https://reactome.org/content/detail/R-MMU-6798695) | Neutrophil degranulation | 7 of 476 | 0.00029 |
| [MMU-162582](https://reactome.org/content/detail/R-MMU-162582) | Signal Transduction | 13 of 2430 | 0.0025 |
| [MMU-997269](https://reactome.org/content/detail/R-MMU-997269) | Inhibition of adenylate cyclase pathway | 2 of 12 | 0.0035 |
| [MMU-170670](https://reactome.org/content/detail/R-MMU-170670) | Adenylate cyclase inhibitory pathway | 2 of 12 | 0.0035 |
| [MMU-392170](https://reactome.org/content/detail/R-MMU-392170) | ADP signalling through P2Y purinoceptor 12 | 2 of 22 | 0.0091 |
| [MMU-202040](https://reactome.org/content/detail/R-MMU-202040) | G-protein activation | 2 of 27 | 0.0125 |
| [MMU-991365](https://reactome.org/content/detail/R-MMU-991365) | Activation of GABAB receptors | 2 of 35 | 0.0162 |
| [MMU-977444](https://reactome.org/content/detail/R-MMU-977444) | GABA B receptor activation | 2 of 35 | 0.0162 |
| [MMU-5674135](https://reactome.org/content/detail/R-MMU-5674135) | MAP2K and MAPK activation | 2 of 36 | 0.0162 |
| [MMU-3928662](https://reactome.org/content/detail/R-MMU-3928662) | EPHB-mediated forward signaling | 2 of 34 | 0.0162 |
| [MMU-392518](https://reactome.org/content/detail/R-MMU-392518) | Signal amplification | 2 of 32 | 0.0162 |
| [MMU-112043](https://reactome.org/content/detail/R-MMU-112043) | PLC beta mediated events | 2 of 32 | 0.0162 |
| [MMU-112040](https://reactome.org/content/detail/R-MMU-112040) | G-protein mediated events | 2 of 33 | 0.0162 |
| [MMU-6814122](https://reactome.org/content/detail/R-MMU-6814122) | Cooperation of PDCL (PhLP1) and TRiC/CCT in G-protein beta folding | 2 of 39 | 0.0166 |
| [MMU-391251](https://reactome.org/content/detail/R-MMU-391251) | Protein folding | 2 of 40 | 0.0167 |
| [MMU-390466](https://reactome.org/content/detail/R-MMU-390466) | Chaperonin-mediated protein folding | 2 of 40 | 0.0167 |
| [MMU-5250924](https://reactome.org/content/detail/R-MMU-5250924) | B-WICH complex positively regulates rRNA expression | 2 of 48 | 0.0218 |
| [MMU-5250913](https://reactome.org/content/detail/R-MMU-5250913) | Positive epigenetic regulation of rRNA expression | 2 of 48 | 0.0218 |
| [MMU-977443](https://reactome.org/content/detail/R-MMU-977443) | GABA receptor activation | 2 of 51 | 0.0228 |
| [MMU-2682334](https://reactome.org/content/detail/R-MMU-2682334) | EPH-Ephrin signaling | 2 of 63 | 0.0328 |
| [MMU-212165](https://reactome.org/content/detail/R-MMU-212165) | Epigenetic regulation of gene expression | 2 of 65 | 0.0328 |
| [MMU-111885](https://reactome.org/content/detail/R-MMU-111885) | Opioid Signalling | 2 of 64 | 0.0328 |
| [MMU-2029480](https://reactome.org/content/detail/R-MMU-2029480) | Fcgamma receptor (FCGR) dependent phagocytosis | 2 of 74 | 0.0404 |
| [MMU-76002](https://reactome.org/content/detail/R-MMU-76002) | Platelet activation, signaling and aggregation | 3 of 242 | 0.0451 |

**UniProt Keywords**

| *keyword* | *description* | *count in gene set* | *false discovery rate* |
| --- | --- | --- | --- |
| [KW-0375](https://www.uniprot.org/keywords/KW-0375) | Hydrogen ion transport | 8 of 48 | 6.16e-12 |
| [KW-0832](https://www.uniprot.org/keywords/KW-0832) | Ubl conjugation | 15 of 2091 | 4.72e-05 |
| [KW-0449](https://www.uniprot.org/keywords/KW-0449) | Lipoprotein | 10 of 790 | 4.72e-05 |
| [KW-0564](https://www.uniprot.org/keywords/KW-0564) | Palmitate | 6 of 326 | 0.00050 |
| [KW-1017](https://www.uniprot.org/keywords/KW-1017) | Isopeptide bond | 11 of 1442 | 0.00051 |
| [KW-0687](https://www.uniprot.org/keywords/KW-0687) | Ribonucleoprotein | 5 of 281 | 0.0020 |
| [KW-0597](https://www.uniprot.org/keywords/KW-0597) | Phosphoprotein | 25 of 7545 | 0.0020 |
| [KW-0399](https://www.uniprot.org/keywords/KW-0399) | Innate immunity | 5 of 287 | 0.0020 |
| [KW-0391](https://www.uniprot.org/keywords/KW-0391) | Immunity | 6 of 453 | 0.0020 |
| [KW-0164](https://www.uniprot.org/keywords/KW-0164) | Citrullination | 3 of 60 | 0.0020 |
| [KW-0689](https://www.uniprot.org/keywords/KW-0689) | Ribosomal protein | 4 of 182 | 0.0028 |
| [KW-0007](https://www.uniprot.org/keywords/KW-0007) | Acetylation | 14 of 3060 | 0.0039 |
| [KW-0547](https://www.uniprot.org/keywords/KW-0547) | Nucleotide-binding | 10 of 1738 | 0.0053 |
| [KW-0813](https://www.uniprot.org/keywords/KW-0813) | Transport | 10 of 1912 | 0.0103 |
| [KW-0336](https://www.uniprot.org/keywords/KW-0336) | GPI-anchor | 3 of 138 | 0.0129 |
| [KW-0342](https://www.uniprot.org/keywords/KW-0342) | GTP-binding | 4 of 324 | 0.0161 |
| [KW-0395](https://www.uniprot.org/keywords/KW-0395) | Inflammatory response | 3 of 158 | 0.0167 |
| [KW-0519](https://www.uniprot.org/keywords/KW-0519) | Myristate | 3 of 174 | 0.0207 |

**PFAM Protein Domains**

| *domain* | *description* | *count in gene set* | *false discovery rate* |
| --- | --- | --- | --- |
| [PF02874](https://pfam.xfam.org/family/PF02874) | ATP synthase alpha/beta family, beta-barrel domain | 2 of 5 | 0.0030 |
| [PF01480](https://pfam.xfam.org/family/PF01480) | PWI domain | 2 of 5 | 0.0030 |
| [PF00006](https://pfam.xfam.org/family/PF00006) | ATP synthase alpha/beta family, nucleotide-binding domain | 2 of 5 | 0.0030 |
| [PF00503](https://pfam.xfam.org/family/PF00503) | G-protein alpha subunit | 2 of 16 | 0.0055 |
| [PF00025](https://pfam.xfam.org/family/PF00025) | ADP-ribosylation factor family | 3 of 163 | 0.0300 |

**INTERPRO Protein Domains and Features**

| *domain* | *description* | *count in gene set* | *false discovery rate* |
| --- | --- | --- | --- |
| [IPR036483](https://www.ebi.ac.uk/interpro/entry/IPR036483) | PWI domain superfamily | 2 of 3 | 0.0032 |
| [IPR020003](https://www.ebi.ac.uk/interpro/entry/IPR020003) | ATPase, alpha/beta subunit, nucleotide-binding domain, active site | 2 of 5 | 0.0033 |
| [IPR004100](https://www.ebi.ac.uk/interpro/entry/IPR004100) | ATPase, F1/V1/A1 complex, alpha/beta subunit, N-terminal domain | 2 of 5 | 0.0033 |
| [IPR002483](https://www.ebi.ac.uk/interpro/entry/IPR002483) | PWI domain | 2 of 5 | 0.0033 |
| [IPR001408](https://www.ebi.ac.uk/interpro/entry/IPR001408) | G-protein alpha subunit, group I | 2 of 8 | 0.0033 |
| [IPR000194](https://www.ebi.ac.uk/interpro/entry/IPR000194) | ATPase, F1/V1/A1 complex, alpha/beta subunit, nucleotide-binding domain | 2 of 5 | 0.0033 |
| [IPR011025](https://www.ebi.ac.uk/interpro/entry/IPR011025) | G protein alpha subunit, helical insertion | 2 of 16 | 0.0068 |
| [IPR001019](https://www.ebi.ac.uk/interpro/entry/IPR001019) | Guanine nucleotide binding protein (G-protein), alpha subunit | 2 of 16 | 0.0068 |
| [IPR027417](https://www.ebi.ac.uk/interpro/entry/IPR027417) | P-loop containing nucleoside triphosphate hydrolase | 7 of 877 | 0.0094 |
| [IPR009000](https://www.ebi.ac.uk/interpro/entry/IPR009000) | Translation protein, beta-barrel domain superfamily | 2 of 30 | 0.0153 |
